# FOXP3 promote the progression of glioblastoma via inhibiting ferroptosis mediated by linc00857/miR-1290/GPX4 axis

**DOI:** 10.1038/s41419-024-06619-4

**Published:** 2024-04-01

**Authors:** Wenpeng Cao, Ya He, Jinzhi Lan, Shipeng Luo, Baofei Sun, Chaolun Xiao, Wenfeng Yu, Zhirui Zeng, Shan Lei

**Affiliations:** 1 Department of Anatomy, School of Basic Medicine, Guizhou Medical University, Guiyang, 550009 Guizhou China; 2 Key Laboratory of Pathogenesis and Drug Research on Common Chronic Diseases, Guizhou Medical University, Guiyang, 550009 Guizhou China; 3 Key Laboratory of Endemic and Ethnic Diseases, Ministry of Education, School of Basic Medicine, Guizhou Medical University, Guiyang, 550009 Guizhou China; 4 Key Laboratory of Endemic and Ethnic Diseases, Ministry of Education, Guizhou Medical University, Guiyang, 550009 Guizhou China; 5 Key Laboratory of Medical Molecular Biology, School of Basic Medicine, Guizhou Medical University, Guiyang, 550009 China

**Keywords:** Transcriptional regulatory elements, CNS cancer, Cell proliferation

## Abstract

The oncogenic properties of members belonging to the forkhead box (FOX) family have been extensively documented in different types of cancers. In this study, our objective was to investigate the impact of FOXP3 on glioblastoma multiforme (GBM) cells. By conducting a screen using a small hairpin RNA (shRNA) library, we discovered a significant association between FOXP3 and ferroptosis in GBM cells. Furthermore, we observed elevated levels of FOXP3 in both GBM tissues and cell lines, which correlated with a poorer prognosis. FOXP3 was found to promote the proliferation of GBM cells by inhibiting cell ferroptosis in vitro and in vivo. Mechanistically, FOXP3 not only directly upregulated the transcription of GPX4, but also attenuated the degradation of GPX4 mRNA through the linc00857/miR-1290 axis, thereby suppressing ferroptosis and promoting proliferation. Additionally, the FOXP3 inhibitor epirubicin exhibited the ability to impede proliferation and induce ferroptosis in GBM cells both in vitro and in vivo. In summary, our study provided evidences that FOXP3 facilitates the progression of glioblastoma by inhibiting ferroptosis via the linc00857/miR-1290/GPX4 axis, highlighting FOXP3 as a potential therapeutic target for GBM.

## Introduction

Glioblastoma (GBM) is recognized as the most malignant neoplasm within the central nervous system, classified as a World Health Organization (WHO) grade IV tumor among all gliomas [1]. Despite the prevailing treatment approach of surgical intervention coupled with radiotherapy and chemotherapy for GBM, the median survival duration remains below 15 months, posing a significant risk to patient overall survival [2, 3]. Therefore, it is urgent to explore novel targets for GBM therapy.

Ferroptosis represents a recently discovered iron-dependent regulated cell death (RCD) mechanism that distinguishes itself from apoptosis, necrosis, autophagy, and other forms of cell death. The pivotal factors contributing to ferroptosis are iron accumulation and lipid peroxidation [4, 5]. Extensive research has demonstrated a notable correlation between iron metabolic imbalance, ferroptosis, and the malignant advancement of diverse tumor types. For instance, the ferroptosis inducer erastin has been shown to augment the susceptibility of glioblastoma multiforme (GBM) to temozolomide [6]. However, it has been observed that various defense mechanisms present in cancer cells can impede the process of ferroptosis, thereby promoting cancer progression. For instance, Li et al. [7] demonstrated that the cysteine protease inhibitor SN enhanced the stability of GPX4 and suppressed ferroptosis in gastric cancer cells, consequently facilitating cell metastasis. Similarly, Wang et al. [8] suggested that linc00336 hindered ferroptosis in lung cancer cells through a competing endogenous RNA (ceRNA) mechanism, leading to increased cell metastasis. Furthermore, our previous study [9] revealed that linc00976 upregulated the expression of GPX4, thereby inhibiting ferroptosis and increasing cholangiocarcinoma cell proliferation and metastasis. Hence, the recognition of defense mechanisms employed by cancer cells to resist ferroptosis was helpful for enhancing cancer therapy.

Forkhead box protein P3 (FOXP3) is a member of the forkhead box (FOX) family of transcription factors [10]. Dysregulation of FOXP3 has been observed in various cancer types. Studies have shown that FOXP3 is upregulated in non-small cell lung cancer tissues, leading to the activation of WNT/β-catenin signaling pathway, cell proliferation, and metastasis [11]. Furthermore, a higher presence of FOXP3+ regulatory T cells infiltrating tumors in breast cancer patients has been associated with a poorer prognosis [12]. FOXP3 can reduce the expression of PPARγ in thyroid cancer cells, thus suppressing cell apoptosis [13]. However, the understanding of the role of FOXP3 in GBM remains limited.

In this current study, we demonstrated that FOXP3 was associated with ferroptosis, elevated in GBM tissues, and indicative of a poor prognosis. Further investigations revealed that FOXP3 bound to the promoter and activated the transcription of GPX4 and linc00857. Additionally, linc00857 functioned as a ceRNA for miR-1290, resulting in increased stability of GPX4 mRNA. These alterations lead to an upregulation of GPX4 expression, a decrease in ferroptosis levels, and ultimately the promotion of GBM cell proliferation. Our research indicated that FOXP3 was a potential biomarker and target for GBM diagnosis and therapy.

## Results

### FOXP3 linked to ferroptosis regulation and elevated in GBM tissues

**Fig. 1:**
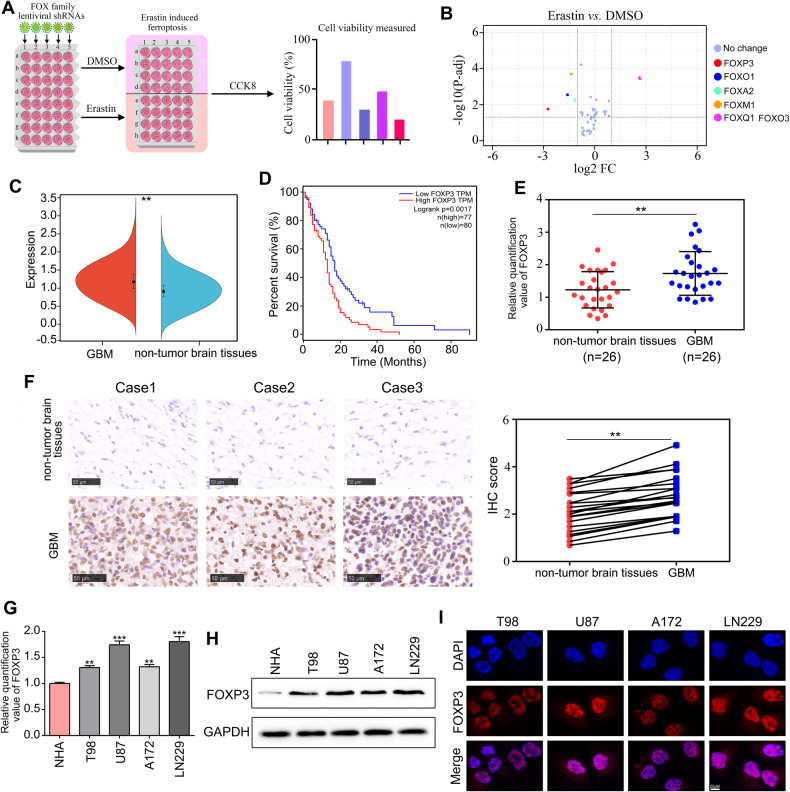
FOXP3 linked to ferroptosis regulation and elevated in GBM tissues. **A** An RNAi screen was performed using an shRNA library targeting 45 members of the FOX family to comprehensively identify specific FOXs that have the potential to regulate ferroptosis in GBM cells. **B** Volcano indicated that the depletion of FOXP3, FOXO1, FOXA2, and FOXM1 resulted in an increased sensitivity of U87 cells to erastin, whereas inhibition of FOXQ1 and FOXO3 led to a reduction in sensitivity. **C** High levels of FOXP3 was observed in GBM tissues from TCGA database. **D** High levels of FOXP3 predicted lower survival days in patients with GBM from TCGA database. **E** qRT-PCR was used to detect the expression of FOXP3 in GBM tissues and non-tumor brain tissues from research cohort. **F** IHC was used to detect the expression of FOXP3 in GBM tissues and non-tumor brain tissues from research cohort. **G**, **H** qRT-PCR and western blotting was used to detect the expression of FOXP3 in NHA, T98, U87, A172 and LN229 cell. **I** immunofluorescence analysis demonstrated that FOXP3 predominantly localized within the cell nucleus. **P < 0.01.

Previous studies had suggested that members of the FOX family possess the capability to regulate ferroptosis and influence the development of diseases [14, 15]. In order to comprehensively identify specific FOXs that have the potential to regulate ferroptosis in GBM cells, an RNAi screen was performed using an shRNA library targeting 45 members of the FOX family (Fig. S1A). Ferroptosis was induced in U87 cells with depletion of FOX using erastin, and the resulting cell viability in response to ferroptosis induction was assessed using the CCK-8 method (Fig. 1A). It was observed that the depletion of FOXP3, FOXO1, FOXA2, and FOXM1 resulted in an increased sensitivity of U87 cells to erastin, whereas inhibition of FOXQ1 and FOXO3 led to a reduction in sensitivity (Fig. 1B). Additionally, the expression levels of FOXP3, FOXO3, FOXO1, FOXA2, and FOXM1 were found to be elevated in GBM tissues from the TCGA database compared to non-tumor brain tissues, while the level of FOXQ1 was reduced (Fig. 1C, Fig. S2A). However, only FOXP3 was found to be positively associated with poor prognosis, while the other factors showed no significant relationship with the prognosis of GBM patients (Fig. 1D, Fig. S1B). Hence, we then assessed the clinical significance of FOXP3 in a research cohort. The findings from qRT-PCR and immunohistochemistry (IHC) analyses revealed a significant upregulation of both FOXP3 mRNA and protein levels in GBM tissues compared to non-tumor brain tissues (Fig. 1E, F). Similarly, elevated levels of FOXP3 mRNA and protein were observed in GBM cell lines (T98, U87, A172, and LN229) when compared to normal astrocyte NHA (Fig. 1G–F). Furthermore, immunofluorescence analysis demonstrated that FOXP3 predominantly localized within the cell nucleus (Fig. 1I). Taken together, the results indicated that FOXP3 linked to ferroptosis, and may play as oncogene in GBM.

### FOXP3 increased the proliferation of GBM cells via inhibiting ferroptosis

**Fig. 2:**
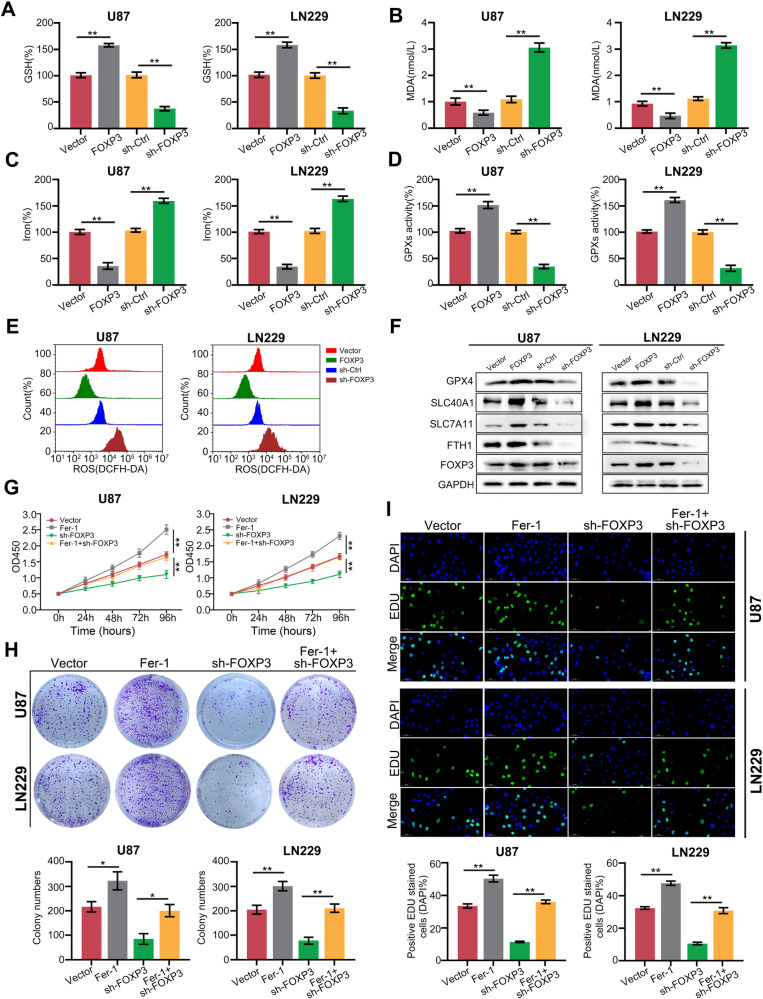
FOXP3 increased the proliferation of GBM cells via inhibiting ferroptosis. The GSH (**A**), MDA (**B**), Iron (**C**), GPX (**D**), and ROS level (**E**) was detected in U87 and LN229 cells with FOXP3-knockdown or FOXP3-overexpression. **F** Western blotting was used to detect the expression of FOXP3, GPX4, SLC40A1, SLC7A11, and FTH1 in U87 and LN229 cells with FOXP3-knockdown or FOXP3-overexpression. **G** CCK-8 indicated the effects of FOXP3-overexpression and FOXP3-knockdown on U87 and LN229 proliferation. **H** Colony formation indicated the effects of FOXP3-overexpression and FOXP3-knockdown on U87 and LN229 colony formation. **I** EDU assay indicated the change of EDU positive rate after FOXP3-overexpression and FOXP3-knockdown in U87 and LN229. *P < 0.05; **P < 0.01.

To further elucidate the biological roles of FOXP3, FOXP3-overexpression lentivirus and FOXP3 shRNAs targeting were employed to generate FOXP3-overexpression and FOXP3 knockdown GBM cells, respectively (Fig. S3A). The findings demonstrated that FOXP3-overexpression resulted in increased levels of GSH (Fig. 2A) and GPXs activity (Fig. 2D) in U87 and LN229 cells, respectively. Conversely, FOXP3 overexpression led to decreased levels of MDA (Fig. 2B) and iron (Fig. 2C). Knockdown of FOXP3 induced the opposite effect. After assessing the levels of reactive oxygen species (ROS) in cells, it was discovered that the overexpression of FOXP3 resulted in a significant reduction in ROS levels. Conversely, the knockdown of FOXP3 in GBM cells led to an elevation in ROS levels (Fig. 2E). Additionally, the impact of FOXP3 on the expression of various proteins, such as GPX4, SLC40A1, SLC7A11, and FTH1, which are known to negatively regulate ferroptosis, was investigated. Western blotting analysis revealed that the overexpression of FOXP3 increased the expression of all these proteins, while the suppression of FOXP3 had the opposite effect (Fig. 2F). These results indicated that FOXP3 had potential to inhibit the ferroptosis of GBM cells.

In addition, we employed CCK-8 (Fig. 2G) and colony formation assay (Fig. 2H) to demonstrate that the downregulation of FOXP3 resulted in decreased proliferation and colony formation in U87 and LN229 cells. Conversely, the using of ferroptosis inhibitor Fer-1 reversed the suppressive effects of FOXP3 knockdown on GBM cell proliferation and colony formation (Fig. 2G, H). Furthermore, the EDU assay revealed a significant reduction in the positive rate with decreased FOXP3 expression, which was attenuated by the administration of Fer-1 (Fig. 2I). Taken together, results indicated that FOXP3 regulated GBM cell proliferation via inhibiting ferroptosis in vitro.

### Knockdown of FOXP3 inhibited the proliferation of GBM cell via inducing ferroptosis in vivo

**Fig. 3:**
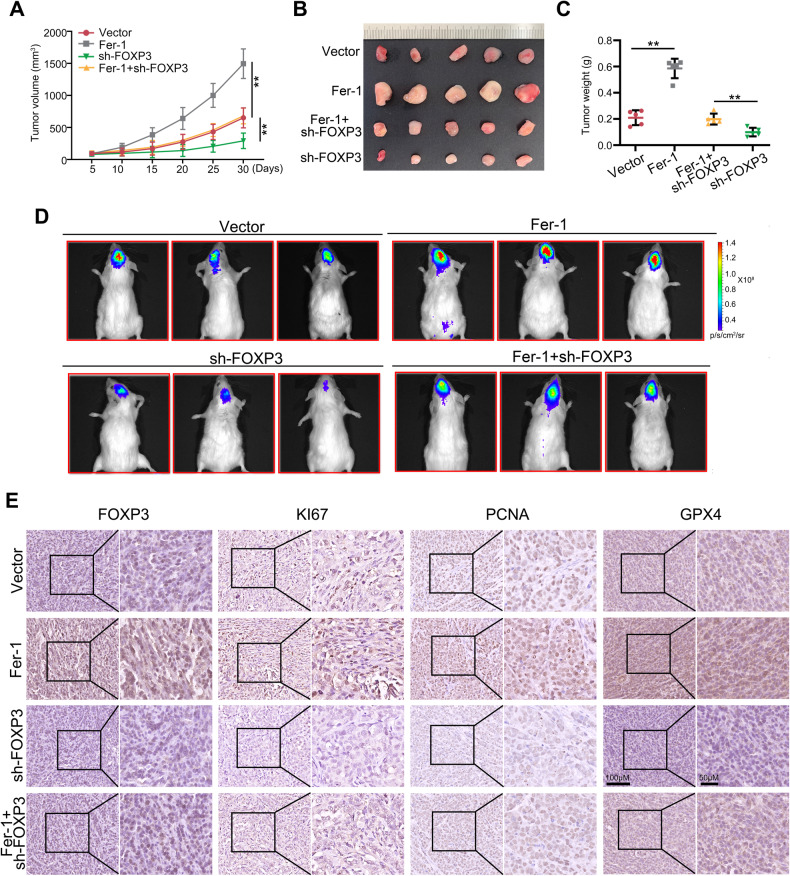
Knockdown of FOXP3 inhibited the proliferation of GBM cell via inducing ferroptosis in vivo. **A**, **B** Subcutaneous tumor formation assay was used to detect whether FOXP3 regulated U87 cell proliferation in vivo via a ferroptosis mechanism. **C** The weight of tumor tissues derived from U87 cells with FOXP3-knockdown or combined treatment with Fer-1. **D** The in situ tumor formation model was used to detect whether FOXP3 regulated U87 cell proliferation in vivo via a ferroptosis mechanism. **E** The expression of FOXP3, KI67, PCNA and GPX4 was detected in tumor tissues derived from U87 cells with FOXP3-knockdown or combined treatment with Fer-1 by IHC method. **P < 0.01.

The effects and mechanisms of FOXP3 on U87 cell proliferation in vivo were investigated using subcutaneous tumor formation and in situ tumor formation models. The results obtained from the subcutaneous tumor formation model demonstrated that knockdown of FOXP3 significantly decreased the proliferation rate (Fig. 3A, B) and weight (Fig. 3C) of tumor tissues derived from U87 cells in vivo. However, the application of Fer-1 reversed these effects. In a similar vein, in situ tumor formation models demonstrated that the downregulation of FOXP3 resulted in a notable decrease in the proliferation of U87 cells in situ. Conversely, the administration of Fer-1 in tissues with FOXP3 knockdown was found to reverse these effects (Fig. 3D). Subsequently, the extracted tissues underwent immunohistochemical analysis to assess the expression levels of KI67, PCNA, and GPX4. It was observed that the downregulation of FOXP3 led to a reduction in the expression of KI67, PCNA, and GPX4 in the tissues. However, the utilization of Fer-1 significantly mitigated the suppressive effects caused by FOXP3 knockdown (Fig. 3E). Taken together, results indicated that Knockdown of FOXP3 inhibited the proliferation of GBM cell via inducing ferroptosis in vivo.

### FOXP3 directly transcribed GPX4 and linc00857

**Fig. 4:**
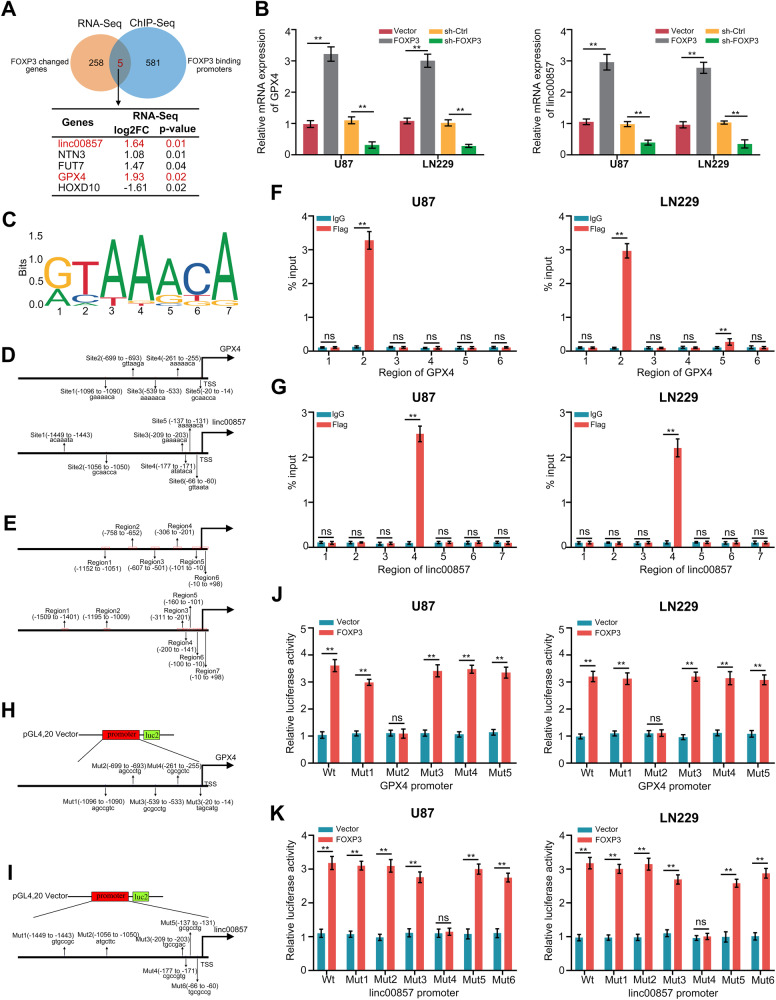
FOXP3 directly increased the transcription of GPX4 and linc00857. **A** Venn diagram indicated the genes directly regulated by FOXP3 and significantly increased in cells with FOXP3-overexpression detecting by RNA-Seq and ChIP-Seq. **B** qRT-PCR was used to detect the expression of GPX4 and linc00857 in U87 and LN229 cells with FOXP3-overexpression and FOXP3-knockdown. **C** FOXP3 binding motif predicted by JASPAR. **D** Schematic diagram of potential FOXP3 binding sites in the promoter region of GPX4 and linc00857. **E** Schematic diagram of primers designed for regions of GPX4 and linc00857 promoter. **F**, **G** ChIP-PCR analysis of enrichment of FOXP3 on GPX4 or linc00857 promoter. IgG was used as a negative control. **H**, **I** Schematic diagram of dual-luciferase reporter vector with GPX4 and linc00857 wildtype or mutation promoter. **J**, **K** The luciferase activity of the wild-type and mutant GPX4 or linc00857 promoters with indicated cells. **P < 0.01.

Transcription factors can bind to the promoter of target genes, and regulate their transcription, thus affecting biological process of cells. Therefore, to determine how FOXP3 regulate ferroptosis in GBM cells, RNA-seq and Chip-seq were performed in U87 cells. Results indicated that five members including linc00857, NTN3, FUT7, GPX4, HOXD10 were directly regulated by FOXP3 and their expression were significantly changed while FOXP3 was overexpressed in U87 cells (Fig. 4A). It is worth noting that, among them, linc00857 has been shown to be an oncogenic lncRNA in various cancers [16, 17], while GPX4 is one of the essential enzymes involved in ferroptosis. We therefore focused on these two FOXP3 downstream members. Using qRT-PCR, we discovered that FOXP3 overexpression significantly increased linc00857 and GPX4 transcription levels, whereas FOXP3 knockdown reduced linc00857 and GPX4 transcription levels in U87 and LN229 cells (Fig. 4B, Fig. S4A). In order to confirm the interaction between FOXP3 and the promoter regions of GPX4 and linc00857, the FOXP3 motif was obtained from the JASPAR database (Fig. 4C). A total of five binding sites in the GPX4 promoter and six binding sites in the linc00857 promoter were identified for FOXP3 (Fig. 4D). PCR primers were then designed to amplify an approximately 100-bp sequence that encompassed the potential binding sites and transcriptional start sites (Fig. 4E). The binding sites of FOXP3 in the GPX4 promoter (site 2, −699 to −693) and linc00857 promoter (site 4, −177 to −171) were confirmed through CHIP-qPCR analysis (Fig. 4F, G). In order to confirm the transcription activity of FOXP3 in the promoter regions of GPX4 and linc00857, we generated plasmids containing mutated promoters of GPX4 (Fig. 4H) and linc00857 (Fig. 4I). Through the utilization of a dual-luciferase reporter assay, we observed that the overexpression of FOXP3 significantly enhanced the fluorescence intensity in U87 and LN229 cells transfected with the wildtype GPX4 (Fig. 4J) and linc00857 (Fig. 4K) plasmids. Conversely, when site 2 in GPX4 and site 4 in linc00857 were mutated, the aforementioned effects were reversed (Fig. 4J, K). These results indicated that FOXP3 directly regulates transcription of GPX4 and linc00857.

### Linc00857 increased the expression of GPX4 via sponging miR-1290

**Fig. 5:**
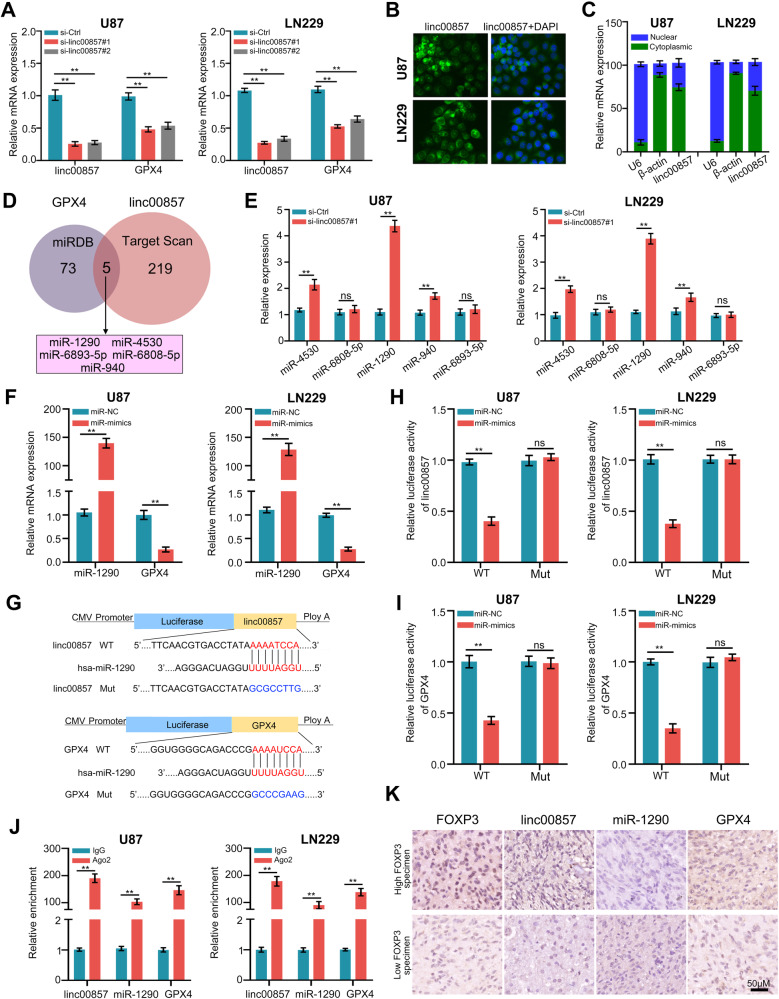
Linc00857 increased the expression of GPX4 via sponging miR-1290. **A** qRT-PCR was used to detect the mRNA levels of GPX4 after linc00857 knockdown. **B**, **C** Subcellular localization of linc00857 was measured using FISH or nucleocytoplasmic fractionation RT-qPCR. **D** Potential miRNAs regulated by linc00857 which can bind with GPX4 were predicted by indicated websites. **E** Expression of potential miRNAs regulated by linc00857 were detected by RT-qPCR in cells with linc00857 knockdown. **F** miR-1290 and GPX4 were detected with RT-qPCR in cells with miR-1290-overexpression. **G** Predicted binding sites between linc00857, GPX4, and miR-1290. **H**, **I** Luciferase activation was detected in cells co-transfected with miR-1290 and wildtype/mutation 3′UTR of linc00857/GPX4. **J** RIP assay was conducted to confirm the interaction between AGO2, linc00857, miR-1290 and GPX4. **K** The co-expression relationship between FOXP3, linc00857, miR-1290 and GPX4 using ISH and IHC method. **P < 0.01.

It is noteworthy that the downregulation of linc00857 resulted in a concomitant decrease in the expression of GPX4 (Fig. 5A). Consequently, our objective was to investigate the regulatory role of linc00857 on GPX4. The subcellular localization analysis revealed that linc00857 predominantly localized in the cytoplasm of U87 and LN229 cells (Fig. 5B, C), suggesting that linc00857 may exert its regulatory influence on GPX4 through a ceRNA mechanism. By employing miRDB and TargetScan for target miRNA prediction, we identified miR-1290, miR-4530, miR-6893-5p, miR-6808-5p, and miR-940 as potential target miRNAs of linc00857 which can regulate GPX4 (Fig. 5D). To further confirm whether linc00857 could directly bind to these miRNA candidates, a pull-down assay with a specific biotin-labeled probe against linc00857 was performed. The pull-down efficiency miR-1290 was significantly enriched by the biotinylated linc00857 probe in both U87 and LN229 cell lines (Fig. S5A). By utilizing qRT-PCR, it was observed that the expression of miR-1290 exhibited a significant increase in response to FOXP3 knockdown, while other miRNAs did not display any notable changes (Fig. 5E) in both U87 and LN229 cells. Furthermore, the overexpression of miR-1290 resulted in a reduction in GPX4 expression (Fig. 5F). Consequently, it can be inferred that there exists a linc00857/miR-1290/GPX4 axis in GBM cells. Plasmids containing linc00857 and GPX4, featuring either wildtype or mutation binding sites, were successfully constructed (Fig. 5G). The findings demonstrated that the overexpression of miR-1290 resulted in a decrease in fluorescence intensity in U87 and LN229 cells that were transfected with the wildtype binding site of linc00857 and GPX4. However, no significant effects were observed in cells transfected with the wildtype binding site of linc00857 and GPX4 (Fig. 5H, I). Immunoprecipitation assays were conducted using an anti-AGO2 antibody, and the results indicated that linc00857, miR-1290, and GPX4 all interacted with the AGO2 protein, suggesting a potential interaction between miR-1290, linc00857, and GPX4 in the RNA-induced silencing complex (Fig. 5J). Moreover, an anti-Ago2 RIP assay revealed that Ago2, linc00857, miR-1290 and GPX4 were all efficiently pulled down in the presence of anti-Ago2 antibodies, but not anti-IgG antibodies. Compared with the NC mimic group, linc00857, miR-1290 and GPX4 were significantly enriched in U87 and LN229 cells transfected with miR-3202 mimics (Fig. S5B). Additionally, our study revealed an elevation in the levels of GPX4 and linc00857 in GBM tissues, while the expression of miR-1290 was found to be decreased (Fig. S5C). Furthermore, we observed a positive correlation between FOXP3 expression and linc00857 and GPX4 in GBM tissues, whereas a negative relationship was observed between FOXP3 and miR-1290 expression (Fig. 5K; Fig. S6). These findings suggest that Linc00857 enhances the expression of GPX4 by acting as a sponge for miR-1290 in GBM.

### Linc00857/miR-1290/GPX4 axis was essential for FOXP3-mediated biological functions

**Fig. 6:**
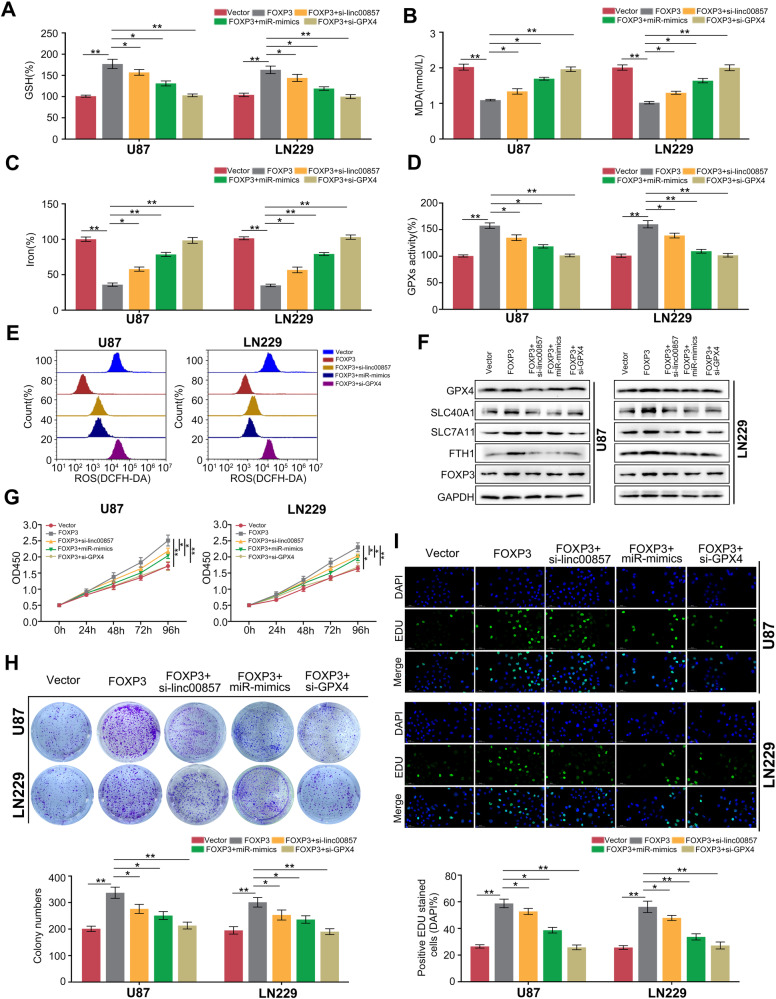
Linc00857/miR-1290/GPX4 axis was essential for FOXP3-mediated biological functions. Linc00857-knockdown, GPX4 knockdown, or miR-1290 overexpression were performed in GBM cells with FOXP3 overexpression. The GSH (**A**), MDA (**B**), Iron (**C**), GPXs activity (**D**), and ROS (**E**) in each group were detected. **F** Western blotting was used to detect the expression of GPX4, SLC40A1, SLC7A11, FTH1, and FOXP3 in group. **G** CCK-8 assays were used to detect the proliferation of cells in each group. **H** Colony formation assays were used to detect the colony formation of cells in each group. **I** The EDU positive rate in each group was detected. *P < 0.05; **P < 0.01.

In order to investigate the potential involvement of the Linc00857/miR-1290/GPX4 axis in the biological functions mediated by FOXP3 in GBM cells, we conducted experiments involving linc00857 knockdown, GPX4 knockdown, or miR-1290 overexpression in GBM cells that also had FOXP3 overexpression. The results demonstrated that these interventions significantly attenuated the elevation of GSH (Fig. 6A) and GPXs activity (Fig. 6D) induced by FOXP3. Similarly, linc00857-knockdown, GPX4 knockdown, or miR-1290 overexpression significantly increased the levels of MDA (Fig. 6B), iron (Fig. 6C), and ROS (Fig. 6E) in GBM tissues with FOXP3 overexpression. Furthermore, our study revealed that the upregulation of GPX4, SLC40A1, SLC7A11, and FTH1, which is mediated by FOXP3 overexpression, can be effectively reversed through linc00857-knockdown, GPX4 knockdown, or miR-1290 overexpression (Fig. 6F). These findings strongly suggest that the Linc00857/miR-1290/GPX4 axis plays a crucial role in FOXP3-mediated ferroptosis. Additionally, all of these interventions were able to reverse the enhanced cell proliferation (Fig. 6G) and colony formation (Fig. 6H) induced by FOXP3 overexpression. Furthermore, elevated EDU positive rate induced by FOXP3 overexpression was relieved by linc00857-knockdown, GPX4 knockdown or miR-1290 overexpression (Fig. 6I). These evidences indicated that linc00857/miR-1290/GPX4 axis was involved in FOXP3-mediated biological functions.

### FOXP3 inhibitor, epirubicin, inhibited the proliferation of GBM cell and induced ferroptosis

**Fig. 7:**
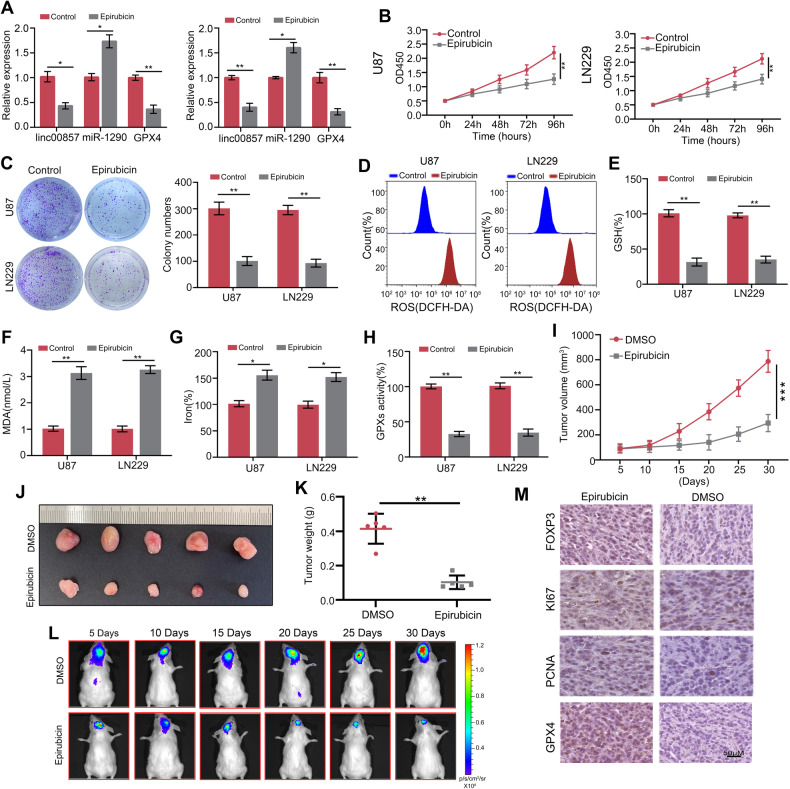
FOXP3 inhibitor, epirubicin, inhibited the proliferation of GBM cell and induced ferroptosis. **A** RT-qPCR were performed to detect the effects of epirubicin on U87 and LN229 cell the expression level of linc00857, miR-1290 and GPX4. **B** CCK-8 assays were performed to detect the effects of epirubicin on U87 and LN229 cell proliferation. **C** Colony formation assays were performed to detect the effects of epirubicin on U87 and LN229 cell colony formation. **D** The ROS levels in cells were detected in U87 and LN229 cells after treatment with epirubicin. **E**–**H** The GSH, MDA, iron, and GPXs activity levels were detected in U87 and LN229 cells after treatment with epirubicin. **I**, **J** Subcutaneous tumor formation assay was used to detect the proliferation of U87 cells in vivo after treatment with epirubicin. **K** Tumor weight of tumor tissues derived from U87 cells treatment with epirubicin. **L** The in situ tumor formation model was used to detect the proliferation of U87 cells in vivo after treatment with epirubicin. **M** Expression of FOXP3, KI67, PCNA and GPX4 in tissues derived from U87 cells treatment with epirubicin. **P < 0.01; ***P < 0.001.

After elucidating the function of FOXP3 in GBM proliferation and ferroptosis and clarifying its underlying mechanisms, we next evaluated the potential therapeutic effect of the FOXP3-specific inhibitor epirubicin in GBM models. The expression levels of linc00857 and GPX4 were found to be significantly decreased, and increased those of miR-1290 in U87 and LN229 cells following epirubicin treatment by RT-qPCR (Fig. 7A). CCK-8 and colony formation assays revealed a significant reduction in proliferation and colony formation in U87 and LN229 cells upon treatment with epirubicin (Fig. 7B, C). Additionally, the levels of reactive oxygen species (ROS) (Fig. 7D), MDA (Fig. 7F), and iron ions (Fig. 7G) were found to be significantly increased in U87 and LN229 cells following epirubicin treatment. Conversely, the levels of GSH (Fig. 7E) and (GPXs activity (Fig. 7H) were observed to be reduced. In a subcutaneous tumor model, it was observed that tumor tissues derived from U87 cells exhibited slower growth (Fig. 7I, J) following treatment with a combination of epirubicin and lower weight (Fig. 7K). Furthermore, findings from an in situ tumor model demonstrated that epirubicin effectively reduced the proliferation of U87-derived tumor tissues in situ (Fig. 7L), as well as downregulating the expression of KI67, PCNA, and GPX4 in the tissues (Fig. 7M). These results suggest that the FOXP3 inhibitor, epirubicin, exerts inhibitory effects on the proliferation of GBM cells and induces ferroptosis.

## Discussion

Ferroptosis, a form of RCD dependent on iron and controlled by GPX4, is primarily initiated by severe lipid peroxidation resulting from the generation of ROS and the availability of iron [18]. A growing body of evidence indicates the significant involvement of ferroptosis in GBM progression, with several drugs demonstrating anti-tumor effects and synergistic effects with temozolomide by targeting regulatory molecules associated with ferroptosis in glioma, especially in GBM [19, 20]. Therefore, the identification of these regulatory molecules holds potential for enhancing GBM therapy.

The dysregulation of FOX family members in GBM has been extensively investigated in multiple studies. One such study revealed that FOXO1 expression was elevated in GBM tissues, and the knockdown of FOXO1 in GBM cells resulted in increased sensitivity to chemotherapy [21]. Similarly, FOXM1 was identified as a potential therapeutic target for GBM, and the knockdown of FOXM1 was shown to reduce the resistance of GBM cells to temozolomide [22]. Additionally, decreased expression of FOXO3 was observed in GBM tissues, and enhanced FOXO3 activity significantly inhibited the proliferation of GBM-derived stem cells [23]. Furthermore, it has been observed that FOXO4 levels decrease in both GBM tissues and cells. Conversely, overexpression of FOXO4 has been shown to decrease cell proliferation and mobility [24]. In this present study, we have presented the initial evidences indicating a connection between FOXP3, a member of the FOX family, and ferroptosis. FOXP3 expression was found to be elevated in GBM tissues and positively correlated with a poor prognosis. Notably, knockdown of FOXP3 resulted in reduced proliferation of GBM cells through the induction of ferroptosis. Taken together, these findings suggested that FOXP3 had the potential to serve as a valuable prognostic indicator and target for therapeutic interventions in GBM.

The pivotal enzyme involved in the process of ferroptosis is GPX4. The accumulation of ROS and subsequent cell death occurs when GPX4 is either inhibited or its functionality is compromised [25, 26]. Notably, tumor cells heavily depend on enzymes such as GPX4 to mitigate oxidative stress and ensure their survival against therapeutic interventions. Consequently, the loss of GPX4 presents a potential avenue for selectively eliminating therapy-resistant tumor cells and averting relapse [27]. Herein, we found that FOXP3 not only directly regulated the transcription of GPX4, but also activated the transcription of linc00857, which can increased the mRNA stability of GPX4 via sponging miR-1290, thus inhibiting ferroptosis in GBM. These evidences provided a novel regulation mechanism of GPX4.

Epirubicin, also known as 4′-Epidoxorubicin, is a semi-synthetic derivative of doxorubicin that is derived from l-arabinose. It exerts its antitumor effects by inhibiting Topoisomerase, thereby impeding the synthesis of DNA and RNA [28, 29]. Epirubicin has been frequently employed in neoadjuvant therapy for breast cancer [30]. Previous studies indicated that FOXP3 was a target of epirubicin, while epirubicin cam inhibit the transcript activation of FOXP3 and regulate the proliferation of Tregs [31, 32]. In our present investigation, we have successfully demonstrated the substantial inhibitory impact of epirubicin on GBM cells, leading to the induction of ferroptosis. These findings provide valuable evidence that may contribute to the expansion of the clinical utility of epirubicin.

Taken together, our results indicated that FOXP3 act as an oncogene in GBM. FOXP3 bound to the promoter and activated the transcription of GPX4 and linc00857, thus leading to an upregulation of GPX4 expression, a decrease in ferroptosis levels, and ultimately the promotion of GBM cell proliferation. Targeting FOXP3 may be a strategy for GBM therapy.

## Materials and methods

### Cell culture and transfection

Normal neurogliocyte (NHA) and four GBM cell lines (U87, T98, A172 and LN229) were obtained from the American Type Culture Collection. All cells were maintained in RPMI-1640 medium (Gibco) supplemented with 10% fetal bovine serum (FBS; Gibco). All cells were cultured in a controlled environment with 5% CO_2_ at 37 °C. The authenticity of all cell lines was confirmed through STR profiling. The FOXP3-overexpression lentivirus, short hairpin RNAs targeting FOXs, and their corresponding control lentiviruses were obtained from Genechem (Shanghai, China). The transfection of lentivirus was conducted using polybrene. A total of 0.5 µg/mL puromycin was used to select cells with stable FOXP3-overexpression or FOXP3-knockdown after 48 h transfection. We obtained small interfering RNA (siRNA) mimics targeting GPX4 and Linc00857 from iGenebio (Beijing, China). Transfecting mimics and siRNAs was performed using Lipo2000 (Thermo Fisher Scientific, USA) according to the manufacturer’s instructions. Sequences of shRNAs, mimics and siRNAs were showed in Supplement Table 1.

### Human ethics and tissue collection

Through written agreements, informed consent of each patient was obtained prior to the acquisition and utilization of these clinical specimens, which were approved by Guizhou Medical University’s Ethics Committee A total of 26 pairs of GBM tissue samples and corresponding non-tumor brain tissues were obtained from patients who had undergone surgical procedures.

### Immunohistochemistry

Following the processes of fixing, embedding, sliding, and deparaffinizing, the GBM or non-tumor brain sections were subjected to blocking using solution containing 3% H_2_O_2_ and 5% BSA. Subsequently, the sections were incubated overnight at 4 °C with antibodies against FOXP3 (1:1000; Cat no. ab215206, Abcam, USA), KI67 (1:200; Cat no. A20018, Abconal, Wuhan, China), PCNA (1:200; Cat no. A0264, Abconal, Wuhan, China), and GPX4 (1:200; Cat no. A1933, Abconal, Wuhan, China). After PBS washing, the sections were treated with an immunohistochemical secondary antibody for 1 h at room temperature, followed by DAB staining, hematoxylin re-staining, and imaging. The evaluation of the results was performed in a blinded manner by two independent pathologists.

### qRT-PCR

TRIzol (Cat no. R0016; Beyotime, Jiangsu, China) was used to extract total RNA, and its concentration was determined using Nano Drop ND1000. Then, the PrimeScript RT Master Mix Kit (Takara, Cat No. RR047A) was used for cDNA synthesis. qPCR analysis was performed using TB Green® Premix Ex Taq™ (Takara, Cat No. RR420A). The relative expression was calculated using the 2^−ΔΔCT^ method, with GAPDH or U6 as the reference gene for internal control. The primer sequences used in this study are shown in Supplementary Table 1.

### Western blotting

Cells were collected and lysed in RIPA buffer (Beyotime, Cat no. P0013B, Jiangsu, China) containing protease and phosphatase inhibitors for a duration of 10 min on ice. Subsequently, the cellular debris was eliminated through centrifugation at 12,000 rpm for 15 min at a temperature of 4 °C. The protein concentrations were determined using the BCA Kit (Beyotime, Cat no. P0011). A total of 50 μg of protein was utilized for denaturing 10% SDS-PAGE, followed by transfer onto a membrane for subsequent blotting with specific antibodies. The specific antibodies employed were anti-FOXP3 (1:1000; Cat no. ab215206, Abcam, USA), anti-GPX4 (1:1000; Cat no. A1933, Abconal, Wuhan, China), anti-SLC40A1 (1:1000; Cat no. A14884, Abconal, Wuhan, China), anti-SLC7A11 (1:1000; Cat no. A2413, Abconal, Wuhan, China), anti-FTH1 (1:1000; Cat no. A19544, Abconal, Wuhan, China) and GAPDH (1:5000; Cat no. AC002, Abconal, Wuhan, China).

### Ferroptosis level detection

Glutathione Assay Kits (Cat no. K264, Biovision, USA) were employed in accordance with the manufacturer’s instructions to assess the glutathione (GSH) levels in U87 and LN229 cells. The levels of MDA were detected in U87 and LN229 cells using the Lipid Peroxidation (MDA) Assay Kit (Cat no. RK05818; Abconal, Wuhan, China), while the iron concentrations were analyzed utilizing an Iron Assay Kit (Cat no. ab83366, Abcam, USA). Glutathione peroxidase (GPX) activity detection kit (Cat no. BC1190, Solarbio, Beijing, China) was used to detect the total activity of GPX in U87 and LN229 cells.

### ROS detection

The cells were seeded in triplicate in 6-well plates and cultured until reaching 80% confluence. To quantify the levels of reactive oxygen species (ROS) in the entire cell population, the cells were cultured in fresh DMEM for 24 h and stained with 10 μM of 2′,7′-dichlorodihydrofluorescein diacetate (DCFH-DA; Invitrogen, USA). Flow cytometry was employed to assess the ROS levels, and the data were analyzed using FlowJo software (version: 7.4.1).

### In vitro biological function experiments

The cell proliferation and colony formation were detected using CCK-8, colony formation and EDU assay. In the CCK-8 assays, U87 and LN229 cells were seeded in triplicate in 96-well plates at a density of 5 × 10^3^ cells per well. After 48 h of culturing, 10 μL of CCK-8 solution was added to each well and incubated for 2 hours. Subsequently, the absorbance of each well was measured at 450 nm using a microplate reader. For the colony formation assays, U87 and LN229 cells were seeded in triplicate in 6-well plates at a density of 1 × 10^3^ cells per well. After 12 days of incubation, the culture medium was removed, and the cell colonies were fixed with paraformaldehyde and stained with crystal violet. The condition of the cell colonies in each well was documented and quantified. The EDU assays were performed using the BeyoClick™ EdU-488 Cell Proliferation Assay Kit (Cat no. C0071S) according to the instruction book provided by manufacturer.

### In vivo cell proliferation detection

The animal experiments conducted in this study were carried out in accordance with the protocols approved by the Ethics Committee of Guizhou Medical University. BALB/c-nude mice, aged 8 weeks, were procured from Animal Experimental Center of Guizhou Medical University and subsequently assigned randomly to each experimental group (*n* = 5). In order to establish the xenograft model, a total of 5 × 10^6^ cells were inoculated into the right flank of each mouse. The tumor volume was subsequently measured at 5-day intervals, and after a period of 30 days, the tumors were excised from the euthanized animals.

### Chip-seq assay

The ChIP assay was conducted using the ChIP kit ab500 (Abcam, USA). A total of 1 × 10^7^ U87 cells with FOXP3-overexpression were fixed with 1% formaldehyde and the reaction was stopped via using 0.1 M glycine. The chromatin was then fragmented into 200–1000 bp fragments using ultrasonication, and these fragments were precipitated using either anti-FOXP3 (1:25; Cat no. ab215206, Abcam, USA) or anti-IgG antibody. Finally, the co-precipitated DNA was extracted using phenol/chloroform for the ChIP-seq step or amplified through qPCR.

### RNA-seq assay

RNA-seq were conducted to compare the gene change between three U87 cell samples with FOXP3-overexpression and three control samples. The total RNA from each sample was extracted using TRIzol reagent and subjected to RNA-seq analyses using the Novaseq 6000 platform. DEGs were identified based on the criteria of |log2FC| > 1 and adjust *P* value < 0.05.

### Dual-luciferase reporter assay

The wild-type (WT) or mutant (MUT) sequences with binding sites for FOXP3 in linc00857 or GPX4 promoter regions were inserted into the pGL4.20 luciferase reporter vector. GBM cells were plated on 96-well culture plates with a density of five hundred cells per well and transfected with 200 ng of luciferase reporter plasmids coupled with WT/MUT sequences. Following transfection for 48 h, firefly luciferase activity was measured and normalized to that of Renilla.

WT or MUT sequences with binding sites for miR-1290 in linc00857 or GPX4 3′UTR were inserted into the pmirGLO vector. Cells were plated in 96-well culture plates at a density of 5 × 10^3^ cells/well for 24 h and transfected with 150 ng plasmids coupled with 50 nM mimics for 48 h. Following transfection for 48 hours, firefly luciferase activity was measured and normalized to that of Renilla.

### RNA immunoprecipitation (RIP)

As part of the RIP assay, GBM cells were lysed with RIP buffer and treated with magnetic beads conjugated to anti-Ago2 or anti-IgG antibodies. BersinBio’s RNA-Binding Protein Immunoprecipitation Kit (Cat no. Bes5101) was used. In three independent replicates, immunoprecipitated RNAs were extracted in accordance with the manufacturer’s instructions and quantified by real-time RT-qPCR.

### ISH

The expression levels of linc00857 and miR-1290 were evaluated in 26 frozen GBM tissue samples using biotin-labeled probes specifically designed by RiboBio. The frozen tissue sections on slides were subjected to proteinase K digestion at a concentration of 15 µg/mL, followed by fixation with 4% paraformaldehyde and dehydration with ethanol. Subsequently, the slides were incubated with the respective probes as per the manufacturer’s instructions. Finally, the visualization of linc00857 and miR-1290 signals was achieved using digoxin substrate, while the nucleus was stained with hematoxylin.

### Statistical analysis

The data analysis was conducted using SPSS 20.0, and the results are reported as mean ± standard deviation. Group comparisons were performed using the two-sided unpaired Student’s t-test. while multiple groups were evaluated using a one-way analysis of variance. The associations between FOXP3, LINC00857, miR-1290, and GPX4 were determined through Pearson correlation analysis. Statistical significance was defined as *P* < 0.05.

### Supplementary information

supplementary figure and table legends

Full and uncropped western blots

Figure S1

Figure S2

Figure S3

Figure S4

Figure S5

Figure S6

Table S1

## Data Availability

The datasets used and/or analyzed during the current study are available from the corresponding author on reasonable request.

## References

[1] Verdugo E, Puerto I, Medina MÁ (2022). An update on the molecular biology of glioblastoma, with clinical implications and progress in its treatment. Cancer Commun.

[2] Minniti G, Niyazi M, Alongi F, Navarria P, Belka C (2021). Current status and recent advances in reirradiation of glioblastoma. Radiat Oncol.

[3] Aldape K, Zadeh G, Mansouri S, Reifenberger G, von Deimling A (2015). Glioblastoma: pathology, molecular mechanisms and markers. Acta Neuropathol.

[4] Liang D, Minikes AM, Jiang X (2022). Ferroptosis at the intersection of lipid metabolism and cellular signaling. Mol Cell.

[5] Liu J, Kang R, Tang D (2022). Signaling pathways and defense mechanisms of ferroptosis. FEBS J.

[6] Chen L, Li X, Liu L, Yu B, Xue Y, Liu Y (2015). Erastin sensitizes glioblastoma cells to temozolomide by restraining xCT and cystathionine-γ-lyase function. Oncol Rep.

[7] Li D, Wang Y, Dong C, Chen T, Dong A, Ren J (2023). CST1 inhibits ferroptosis and promotes gastric cancer metastasis by regulating GPX4 protein stability via OTUB1. Oncogene.

[8] Wang M, Mao C, Ouyang L, Liu Y, Lai W, Liu N (2019). Long noncoding RNA LINC00336 inhibits ferroptosis in lung cancer by functioning as a competing endogenous RNA. Cell Death Differ.

[9] Lei S, Cao W, Zeng Z, Zhang Z, Jin B, Tian Q (2022). JUND/linc00976 promotes cholangiocarcinoma progression and metastasis, inhibits ferroptosis by regulating the miR-3202/GPX4 axis. Cell Death Dis.

[10] Jackson BC, Carpenter C, Nebert DW, Vasiliou V (2010). Update of human and mouse forkhead box (FOX) gene families. Hum Genomics.

[11] Yang S, Liu Y, Li MY, Ng CSH, Yang SL, Wang S (2017). FOXP3 promotes tumor growth and metastasis by activating Wnt/β-catenin signaling pathway and EMT in non-small cell lung cancer. Mol Cancer.

[12] Usman AN, Ahmad M, Sinrang AW, Natsir S, Takko AB, Ariyandy A (2023). FOXP3 regulatory T cells on prognosis of breast cancer. Breast Dis.

[13] Chu R, Liu SY, Vlantis AC, van Hasselt CA, Ng EK, Fan MD (2015). Inhibition of Foxp3 in cancer cells induces apoptosis of thyroid cancer cells. Mol Cell Endocrinol.

[14] Wang J, Deng M, Yang J, Zhou X, Yang P, Li Y (2023). FOXN4 affects myocardial ischemia-reperfusion injury through HIF-1α/MMP2-mediated ferroptosis of cardiomyocytes. Cell Mol Biol (Noisy-le-grand).

[15] Ding Y, Wu Q (2023). 1,25D/VDR inhibits pancreatic β cell ferroptosis by downregulating FOXO1 expression in diabetes mellitus. Cell Signal.

[16] Chen P, Zeng Z, Wang J, Cao W, Song C, Lei S (2022). Long noncoding RNA LINC00857 promotes pancreatic cancer proliferation and metastasis by regulating the miR-130b/RHOA axis. Cell Death Discov.

[17] Zhang W, Ji K, Min C, Zhang C, Yang L, Zhang Q (2023). Oncogenic LINC00857 recruits TFAP2C to elevate FAT1 expression in gastric cancer. Cancer Sci.

[18] Seibt TM, Proneth B, Conrad M (2019). Role of GPX4 in ferroptosis and its pharmacological implication. Free Radic Biol Med.

[19] Cao W, Chen X, Xiao C, Lin D, Li Y, Luo S (2023). Ar-turmerone inhibits the proliferation and mobility of glioma by downregulating cathepsin B. Aging.

[20] Cai J, Ye Z, Hu Y, Ye L, Gao L, Wang Y (2023). Fatostatin induces ferroptosis through inhibition of the AKT/mTORC1/GPX4 signaling pathway in glioblastoma. Cell Death Dis.

[21] Han J, Yu X, Wang S, Wang Y, Liu Q, Xu H (2022). IGF2BP2 Induces U251 glioblastoma cell chemoresistance by inhibiting FOXO1-mediated PID1 expression through stabilizing lncRNA DANCR. Front Cell Dev Biol.

[22] Zhang N, Wu X, Yang L, Xiao F, Zhang H, Zhou A (2012). FoxM1 inhibition sensitizes resistant glioblastoma cells to temozolomide by downregulating the expression of DNA-repair gene Rad51. Clin Cancer Res.

[23] Audesse AJ, Karashchuk G, Gardell ZA, Lakis NS, Maybury-Lewis SY, Brown AK (2021). FOXO3 regulates a common genomic program in aging and glioblastoma stem cells. Aging Cancer.

[24] Qi M, Sun LA, Jiang XC, Han YL, Wang L, Niu WH (2020). FOXO4 expression associates with glioblastoma development and FOXO4 expression inhibits cell malignant phenotypes in vitro and in vivo. Life Sci.

[25] Zhan S, Lu L, Pan SS, Wei XQ, Miao RR, Liu XH (2022). Targeting NQO1/GPX4-mediated ferroptosis by plumbagin suppresses in vitro and in vivo glioma growth. Br J Cancer.

[26] Sekhar KR, Hanna DN, Cyr S, Baechle JJ, Kuravi S, Balusu R (2022). Glutathione peroxidase 4 inhibition induces ferroptosis and mTOR pathway suppression in thyroid cancer. Sci Rep.

[27] Sui X, Zhang R, Liu S, Duan T, Zhai L, Zhang M (2018). RSL3 drives ferroptosis through GPX4 inactivation and ROS production in colorectal cancer. Front Pharmacol.

[28] Onrust SV, Wiseman LR, Goa KL (1999). Epirubicin: a review of its intravesical use in superficial bladder cancer. Drugs Aging.

[29] Kaklamani VG, Gradishar WJ (2003). Epirubicin versus doxorubicin: which is the anthracycline of choice for the treatment of breast cancer?. Clin Breast Cancer.

[30] Zhang J, Jiang H, Zhang J, Bao G, Zhang G, Wang H (2021). Effectiveness and safety of pegylated liposomal doxorubicin versus epirubicin as neoadjuvant or adjuvant chemotherapy for breast cancer: a real-world study. BMC Cancer.

[31] Kashima H, Momose F, Umehara H, Miyoshi N, Ogo N, Muraoka D (2016). Epirubicin, identified using a novel luciferase reporter assay for Foxp3 inhibitors, inhibits regulatory T cell activity. PLoS ONE.

[32] Oda N, Shimazu K, Naoi Y, Morimoto K, Shimomura A, Shimoda M (2012). Intratumoral regulatory T cells as an independent predictive factor for pathological complete response to neoadjuvant paclitaxel followed by 5-FU/epirubicin/cyclophosphamide in breast cancer patients. Breast Cancer Res Treat.

