# Supplementary material for: FOXP3 promote the progression of glioblastoma via inhibiting ferroptosis mediated by linc00857/miR-1290/GPX4 axis
Source: Cell Death Dis. 2024 Apr 1;15(4):239. doi: 10.1038/s41419-024-06619-4 (PMC10984987; doi:10.1038/s41419-024-06619-4)
Supplement: Supplementary file 1 — supplementary figure and table legends [file 41419_2024_6619_MOESM1_ESM.docx]

**Supplementary figure and table legends**

**Table S1: The primers used in the article.**

**Figure S1. Efficiency of shRNA for FOX family knockdown was detected by western blotting.**

**Figure S2. The expression and clinical value of FOXs in GBM from TCGA database.** (A) The expression of FOXO3, FOXO1, FOXA2, FOXM1, and FOXQ1 in GBM tissues and non-tumor brain tissues were analyzed in TCGA database. (B) The relationship between FOXO3, FOXO1, FOXA2, FOXM1, and FOXQ1 expression and survival days in GBM patients were analyzed in TCGA database. **, P<0.01.

**Figure S3. Efficiency of lentivirus for FOXP3-overexpression and FOXP3 knockdown was detected by qRT-PCR.**

**Figure S4. qRT-PCR was used to detect the expression of NTN3, FUT7 and HOXD10 in U87 and LN229 cells with FOXP3-overexpression and FOXP3-knockdown.**

**Figure S5. Linc00857 increased the expression of GPX4 via sponging miR-1290.** (A) The relative levels of five miRNA candidates in the GBM cell lysates were examined by RT-qPCR. (B) Anti-Ago2 RIP assay was conducted in GBM cells after transfection with miR-1290 mimics or NC mimics, followed by western blotting and RT-qPCR analyses to detect the expression levels of Ago2, linc00857, GPX4 and miR-1290. (C) Expression of linc00857, miR-1290 and GPX4 was detected using qRT-PCR in GBM tissues and non-tumor brain tissues using qRT-PCR.

**Figure S6. The co-expression relationship between FOXP3, linc00857, miR-1290 and GPX4 in GBM tissues was analyzed.**
