# Supplementary material for: FOXP3 promote the progression of glioblastoma via inhibiting ferroptosis mediated by linc00857/miR-1290/GPX4 axis
Source: Cell Death Dis. 2024 Apr 1;15(4):239. doi: 10.1038/s41419-024-06619-4 (PMC10984987; doi:10.1038/s41419-024-06619-4)

Figure S1C FOXA1

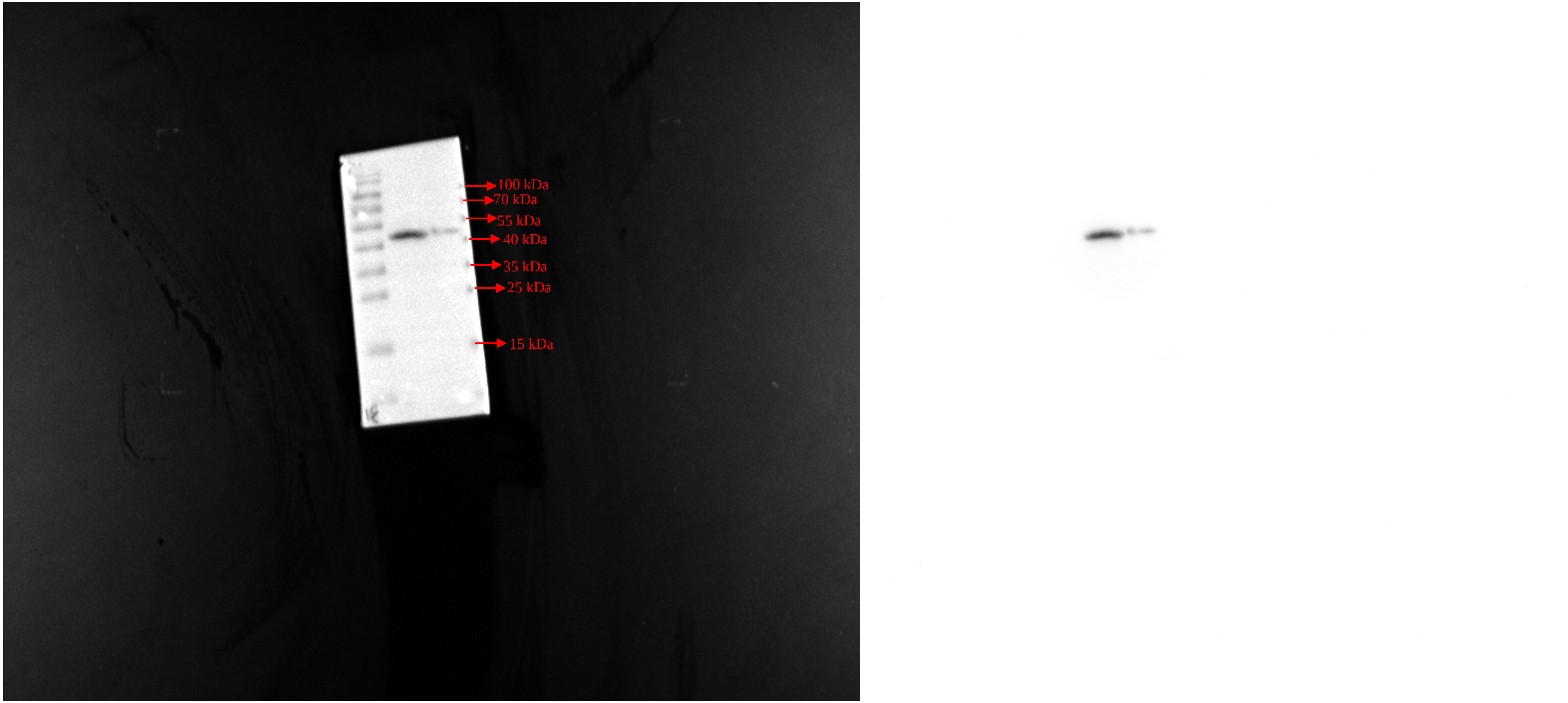

Figure S1C GAPDH

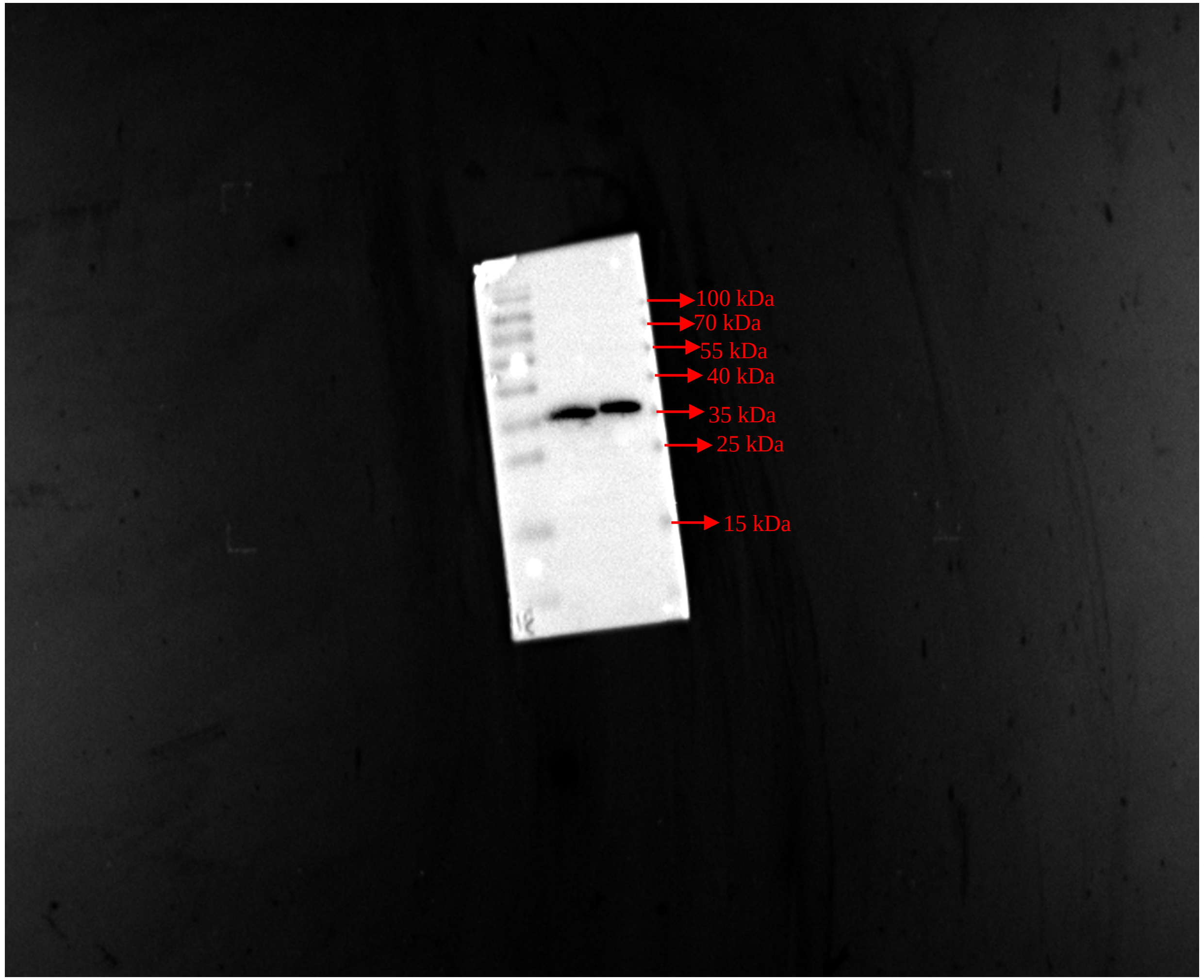

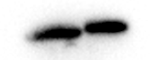

Figure S1C FOXA2

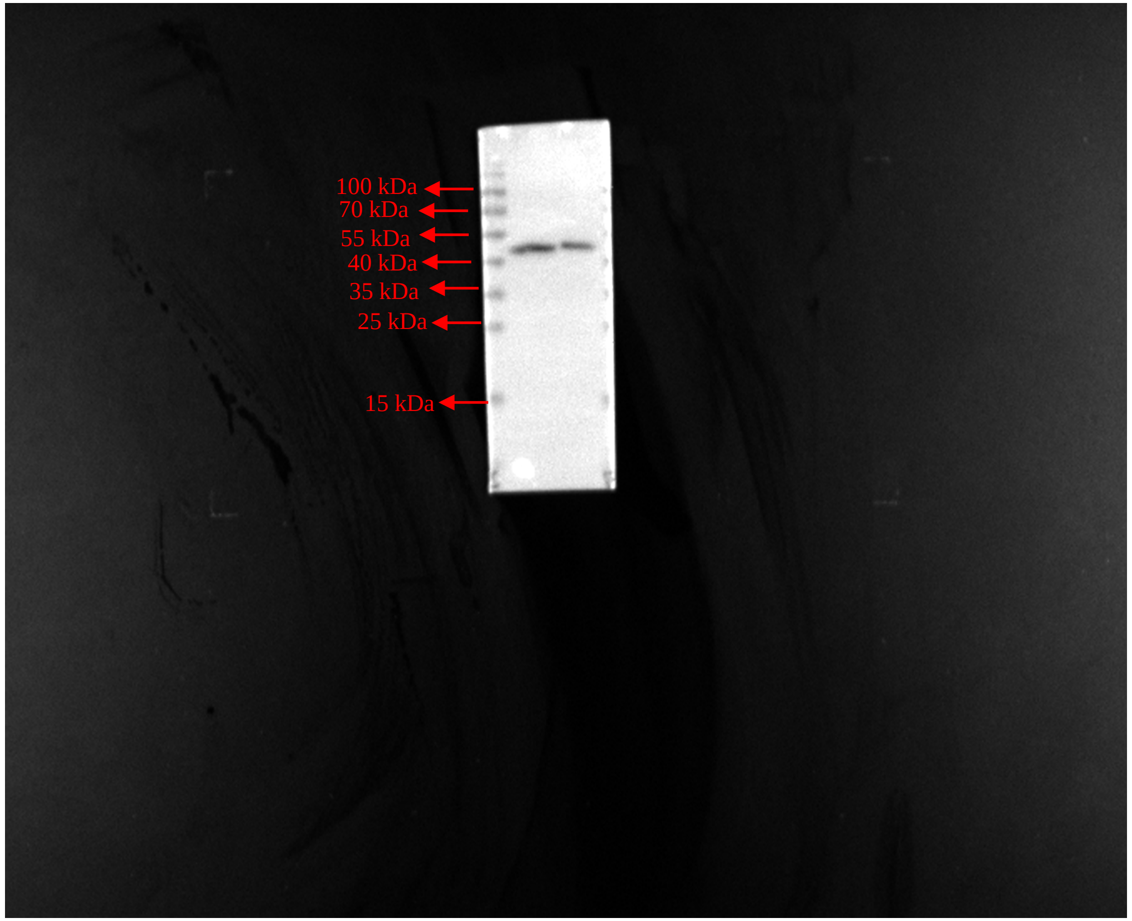

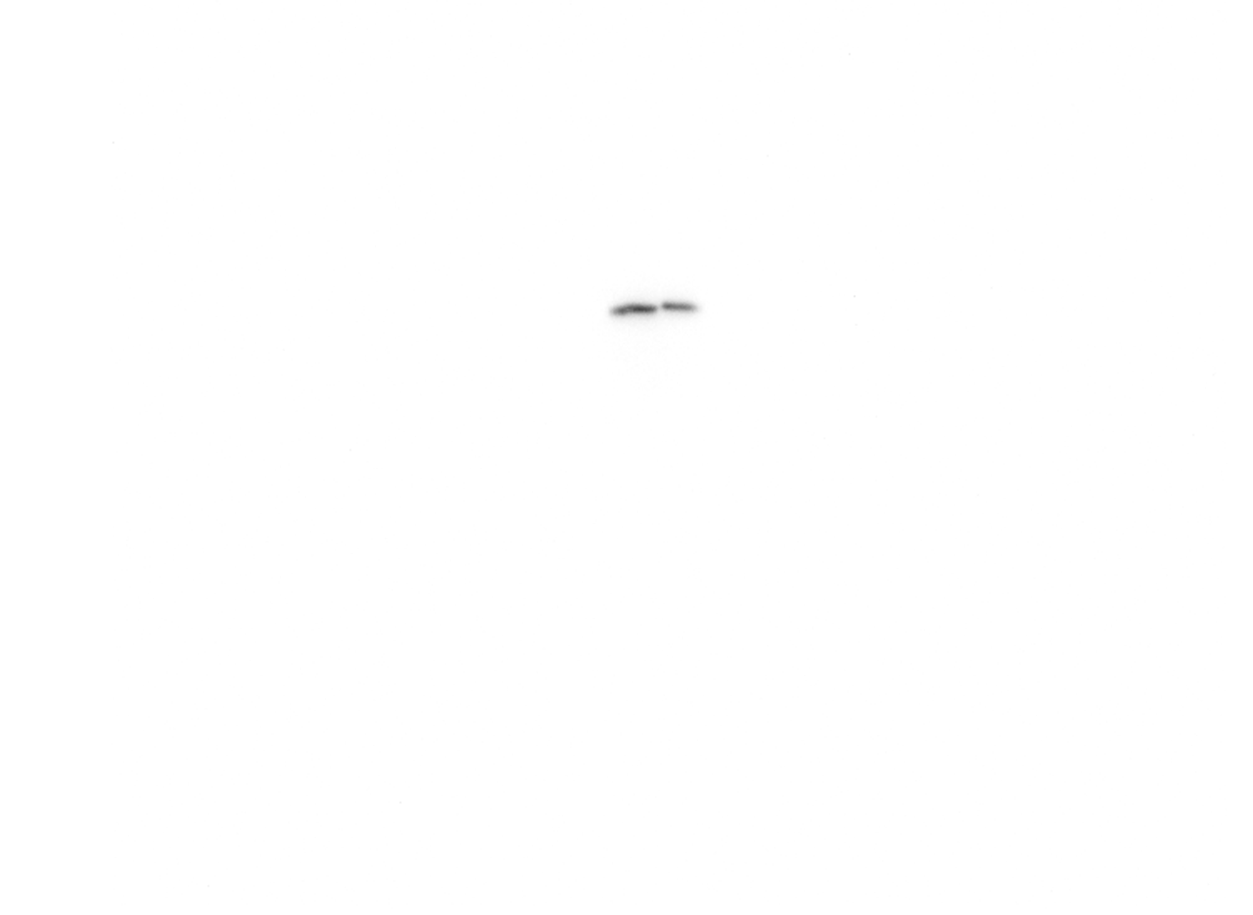

Figure S1C GAPDH

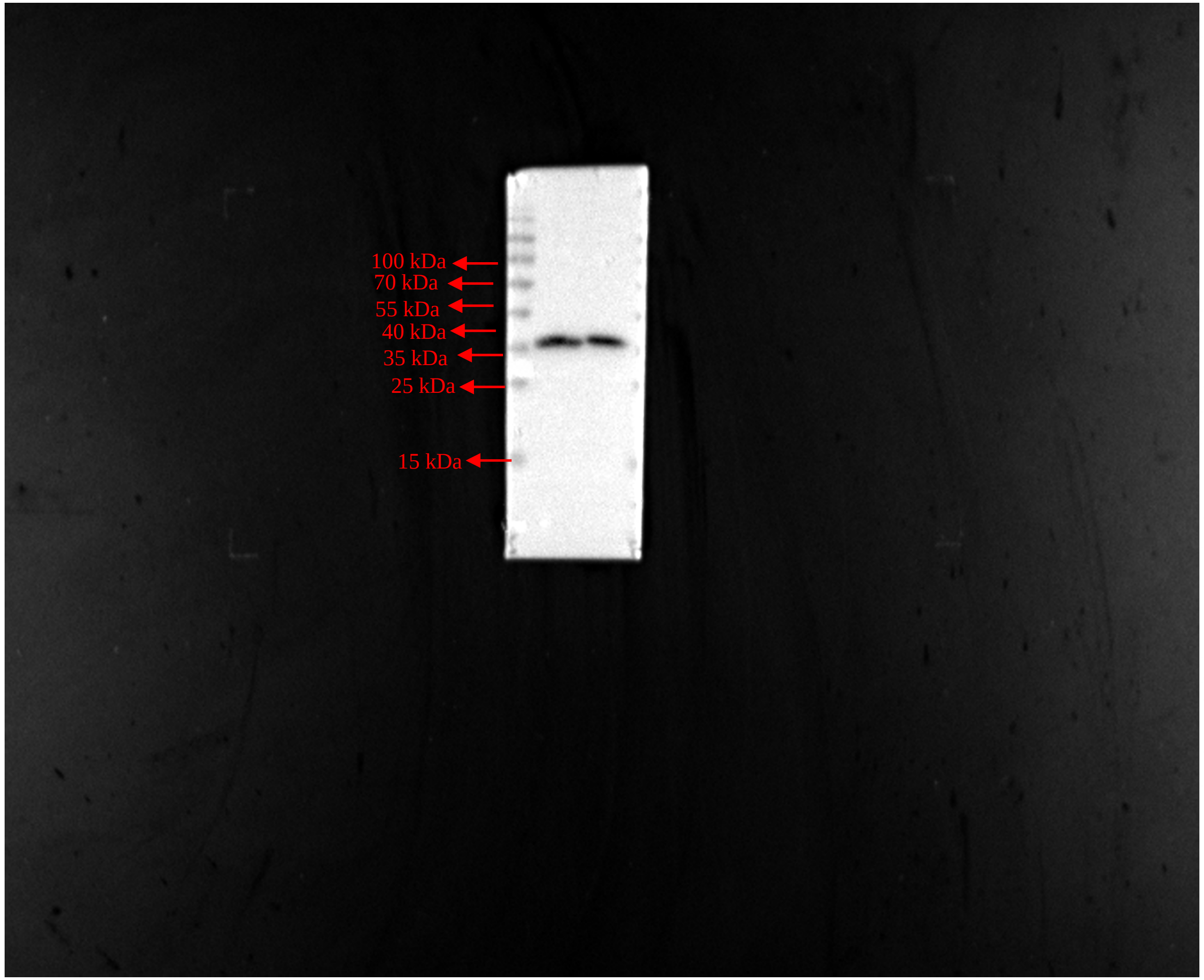

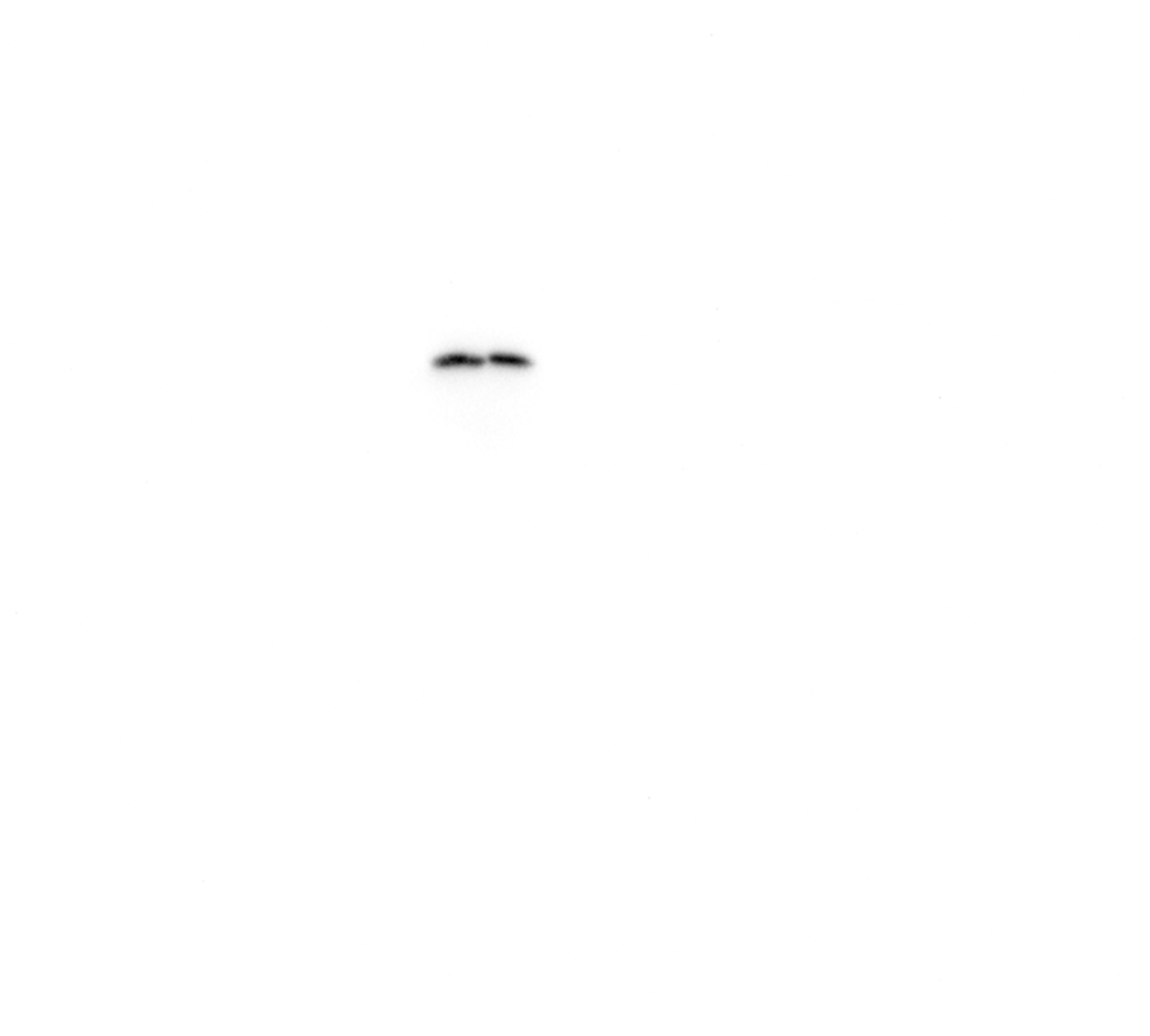

Figure S1C FOXA3

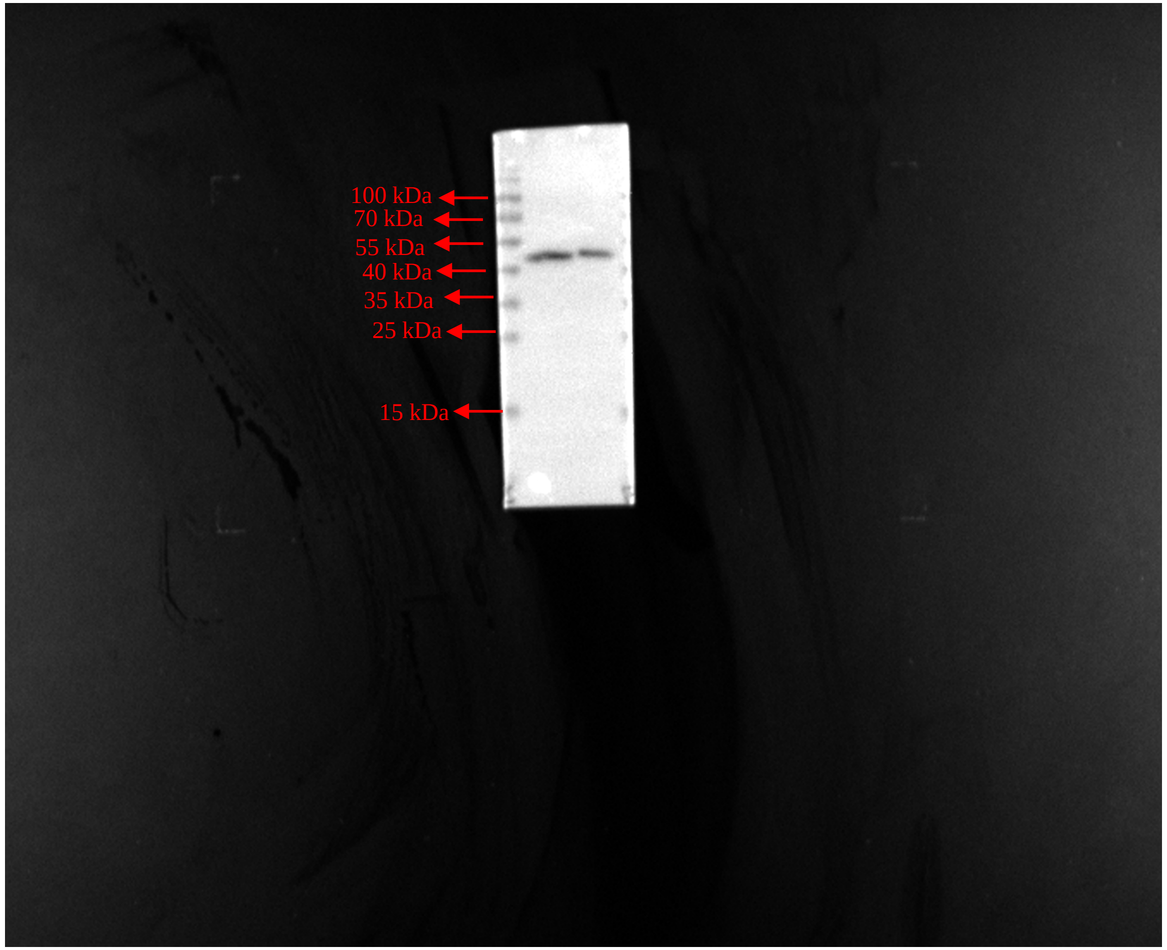

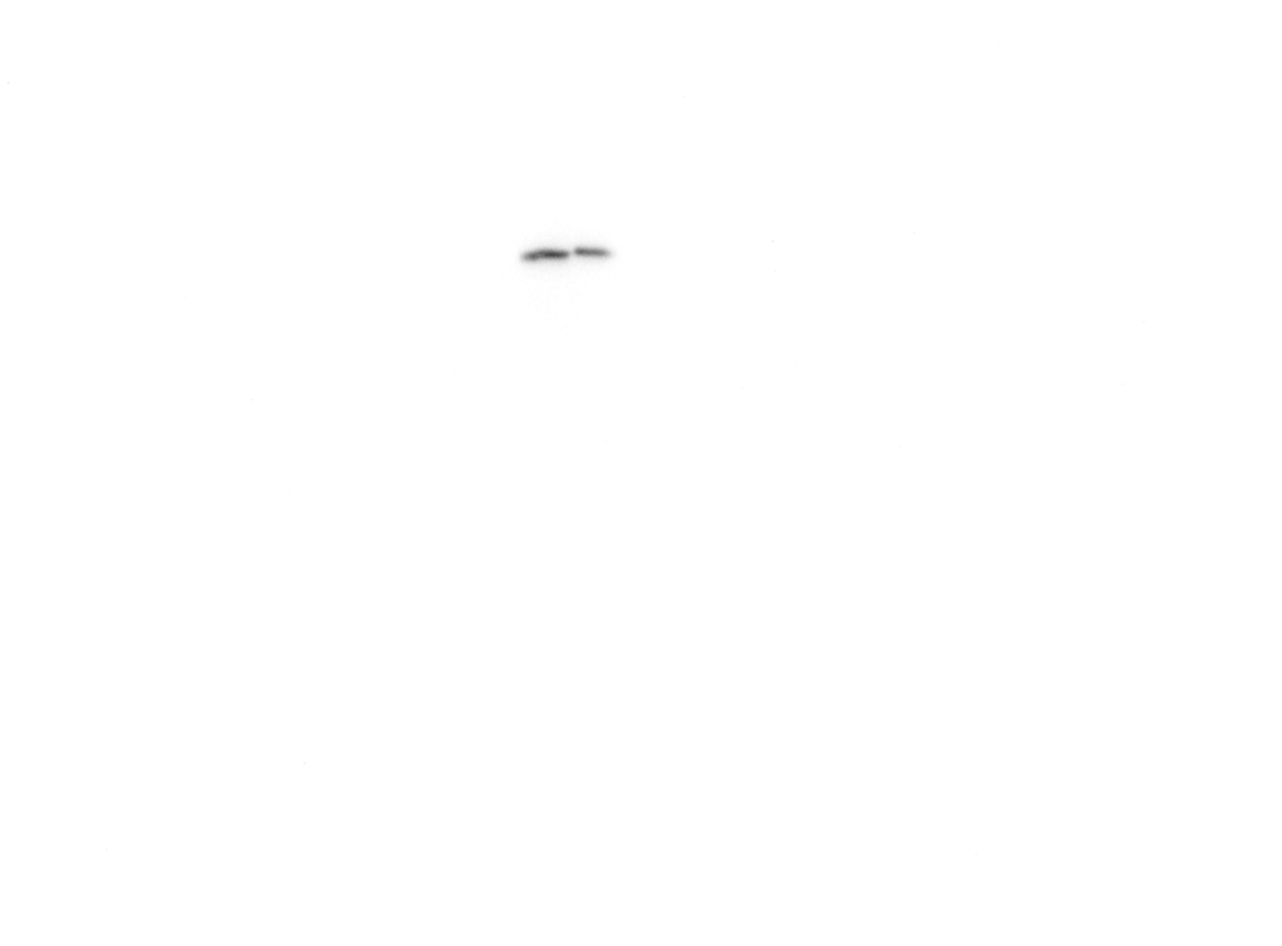

Figure S1C GAPDH

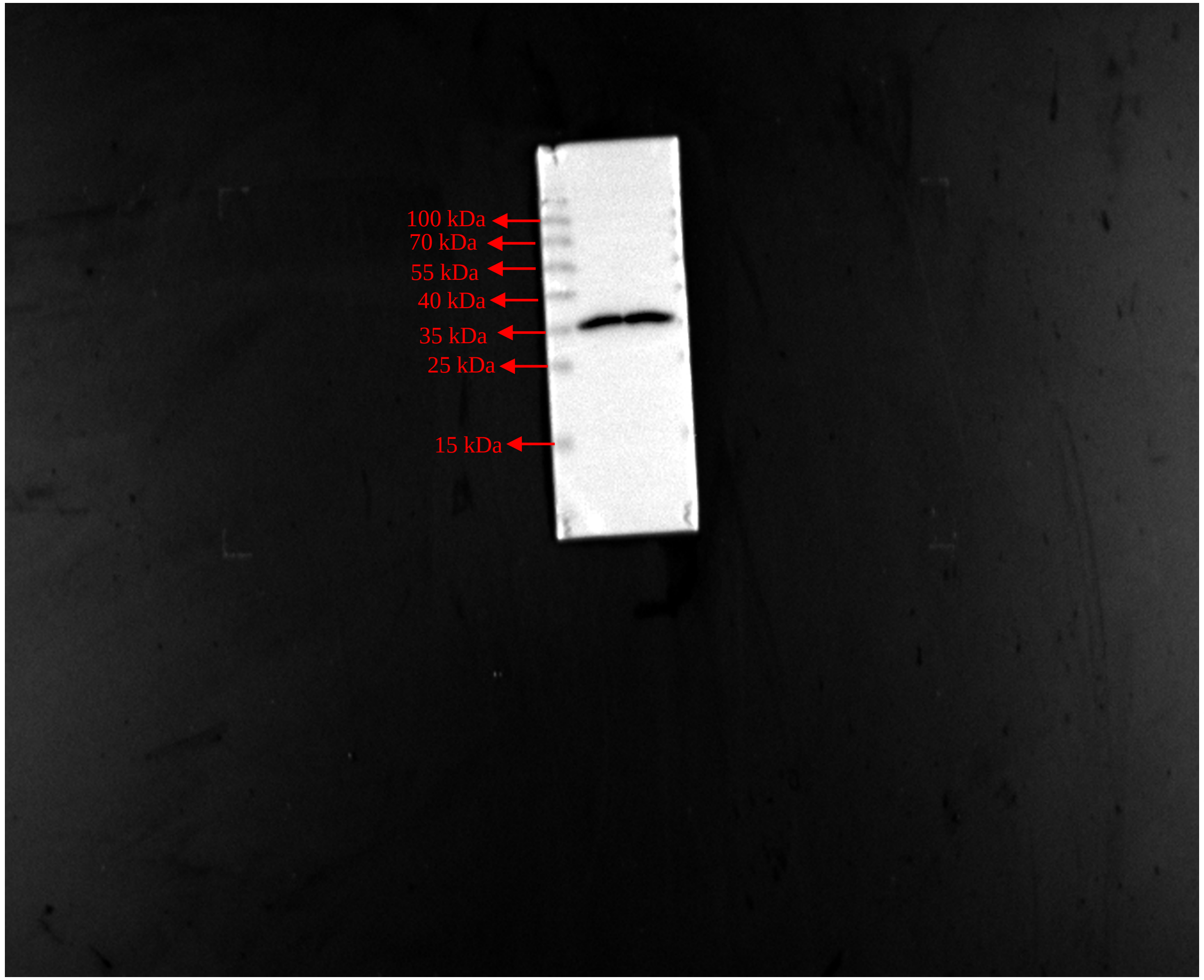

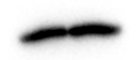

Figure S1C FOXB1

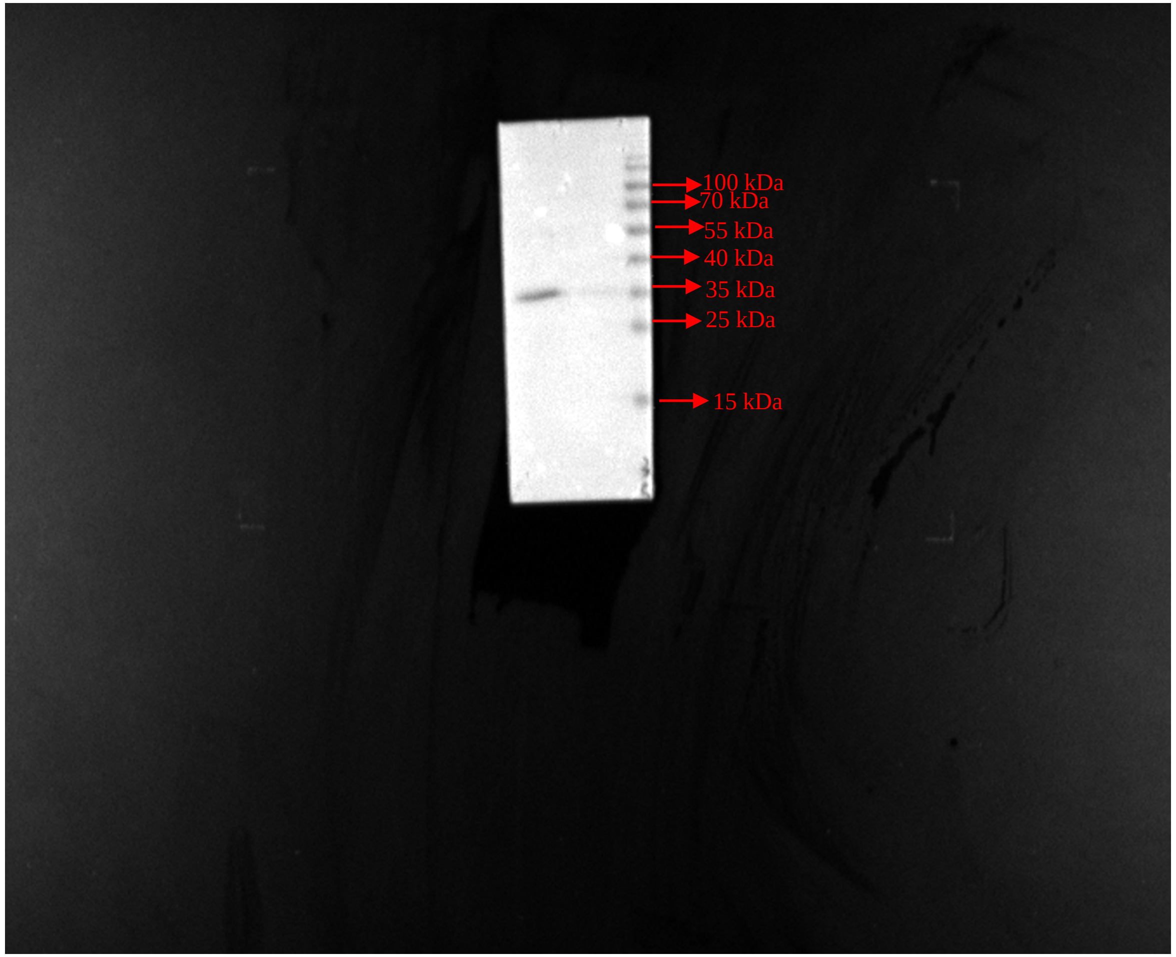

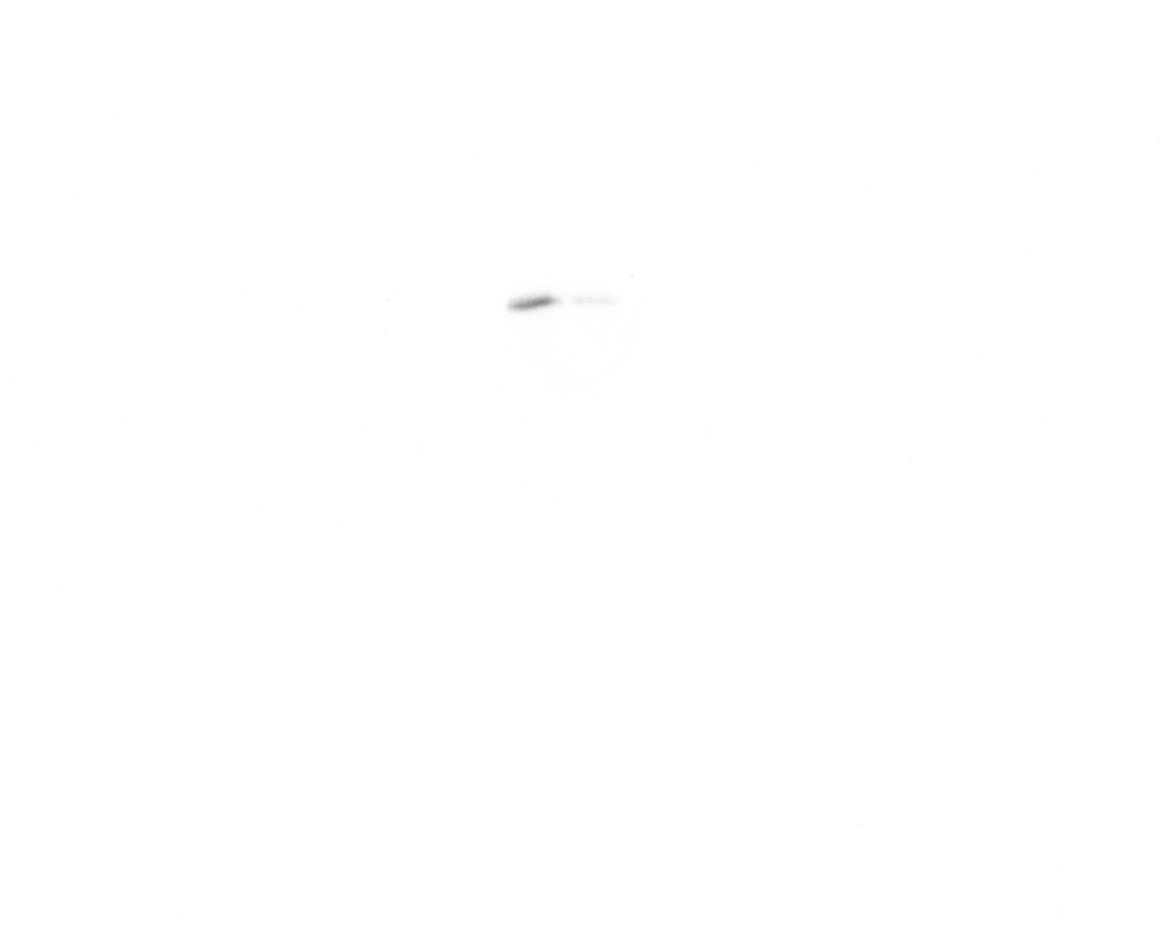

Figure S1C GAPDH

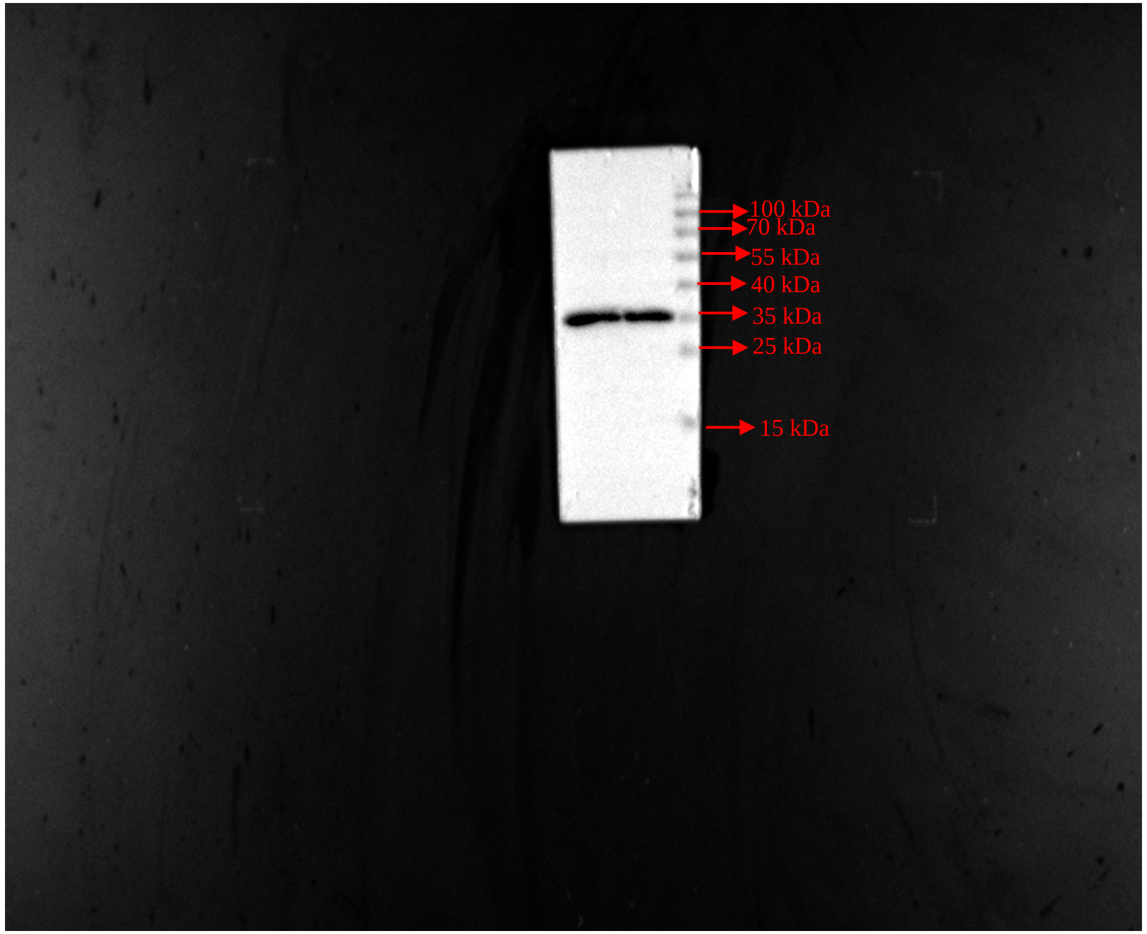

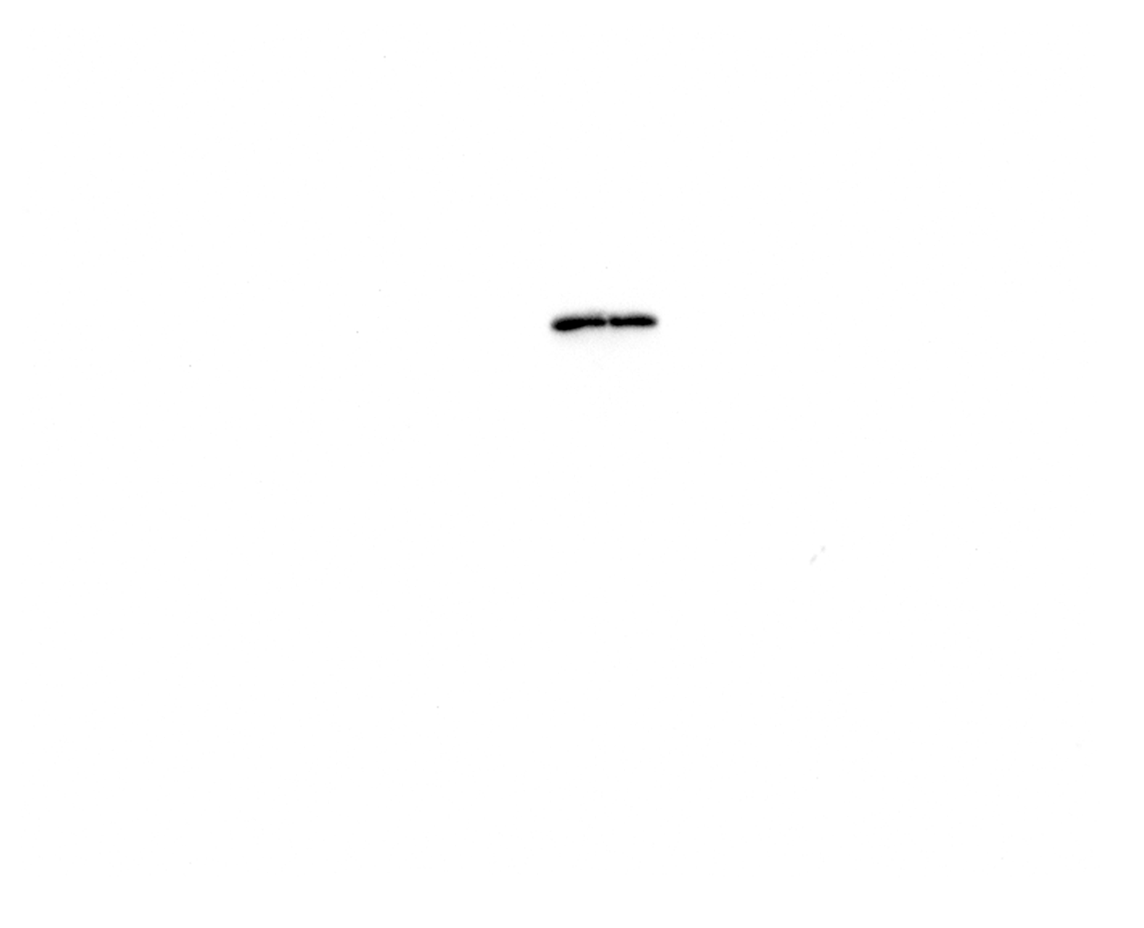

Figure S1C FOXB2

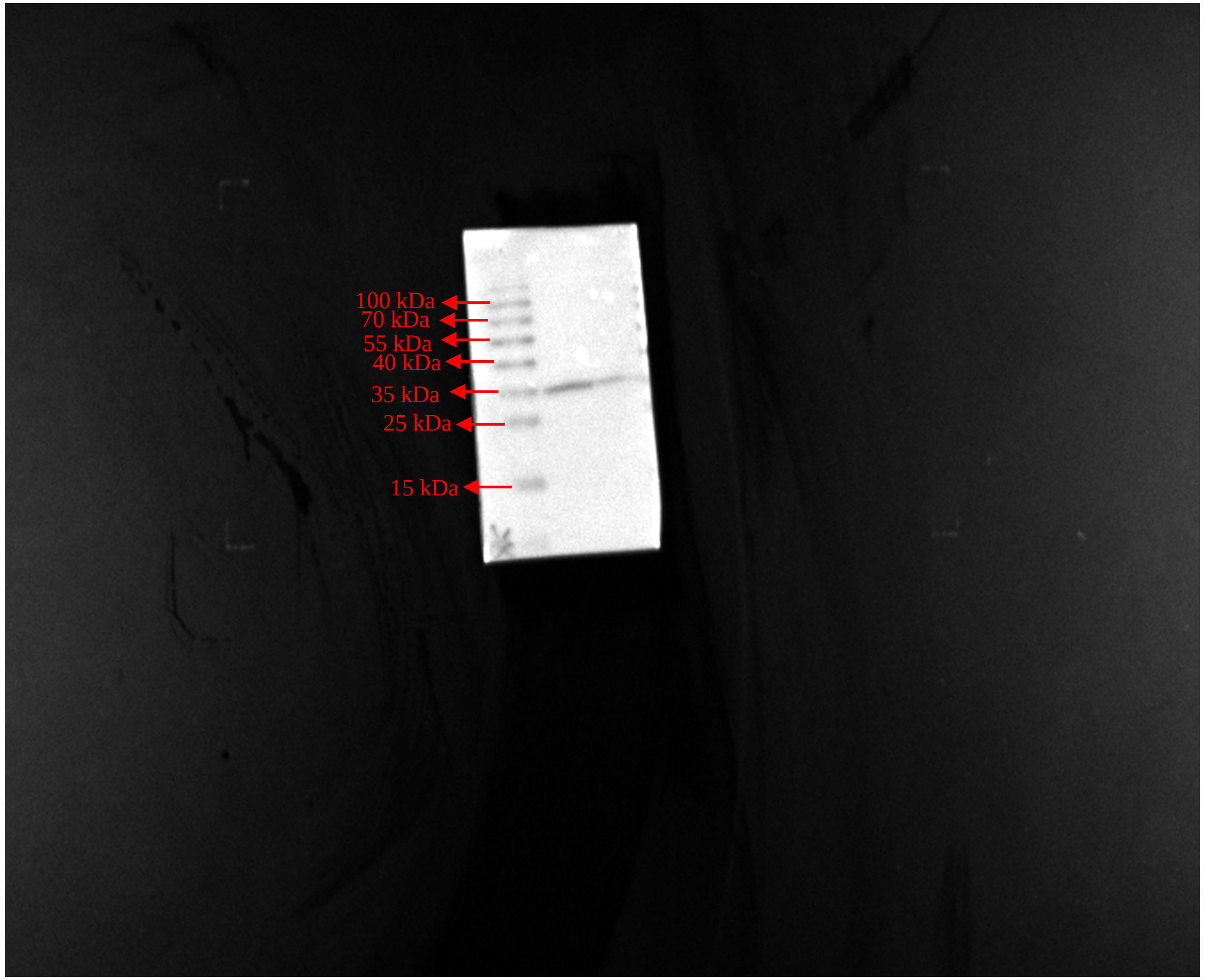

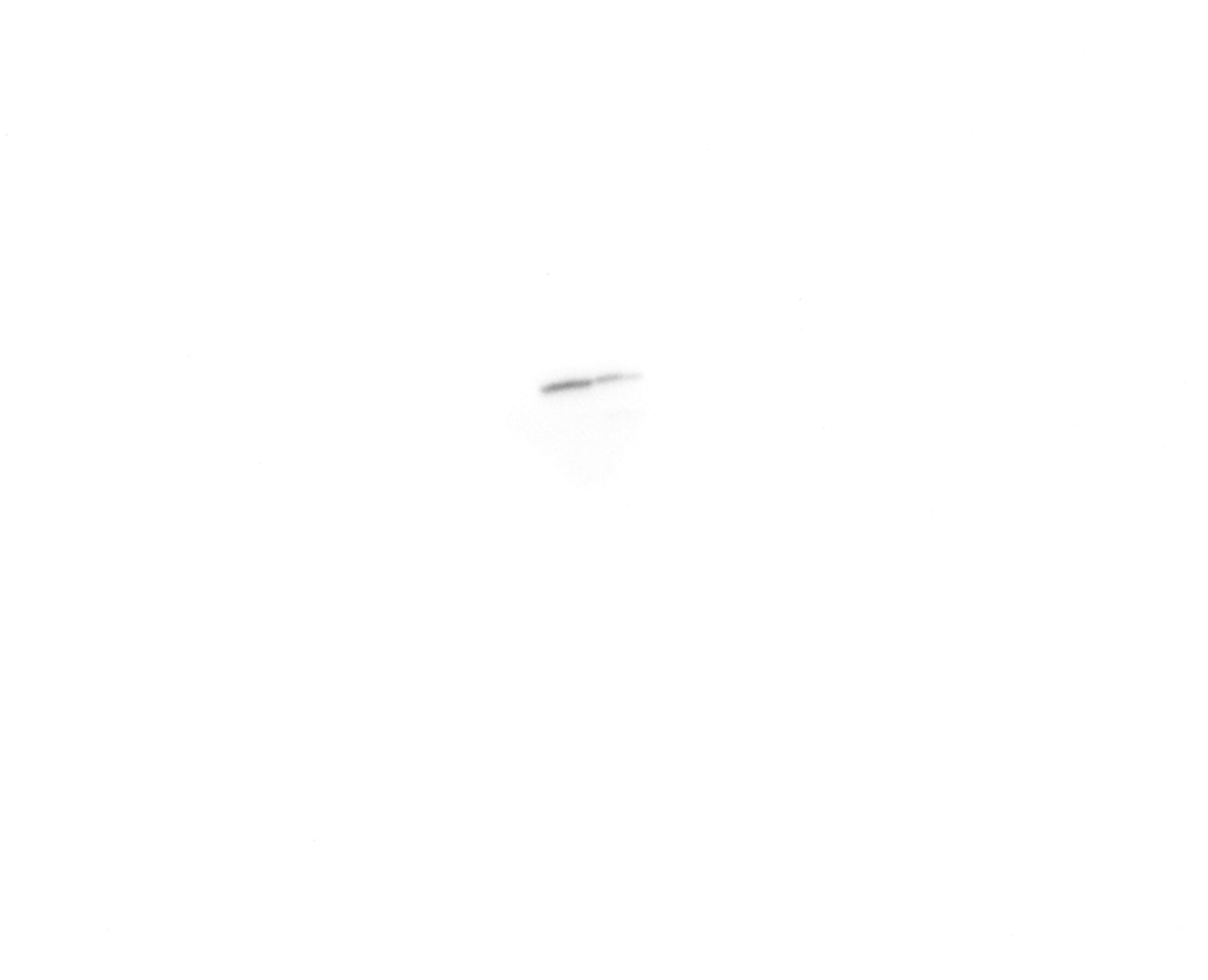

Figure S1C GAPDH

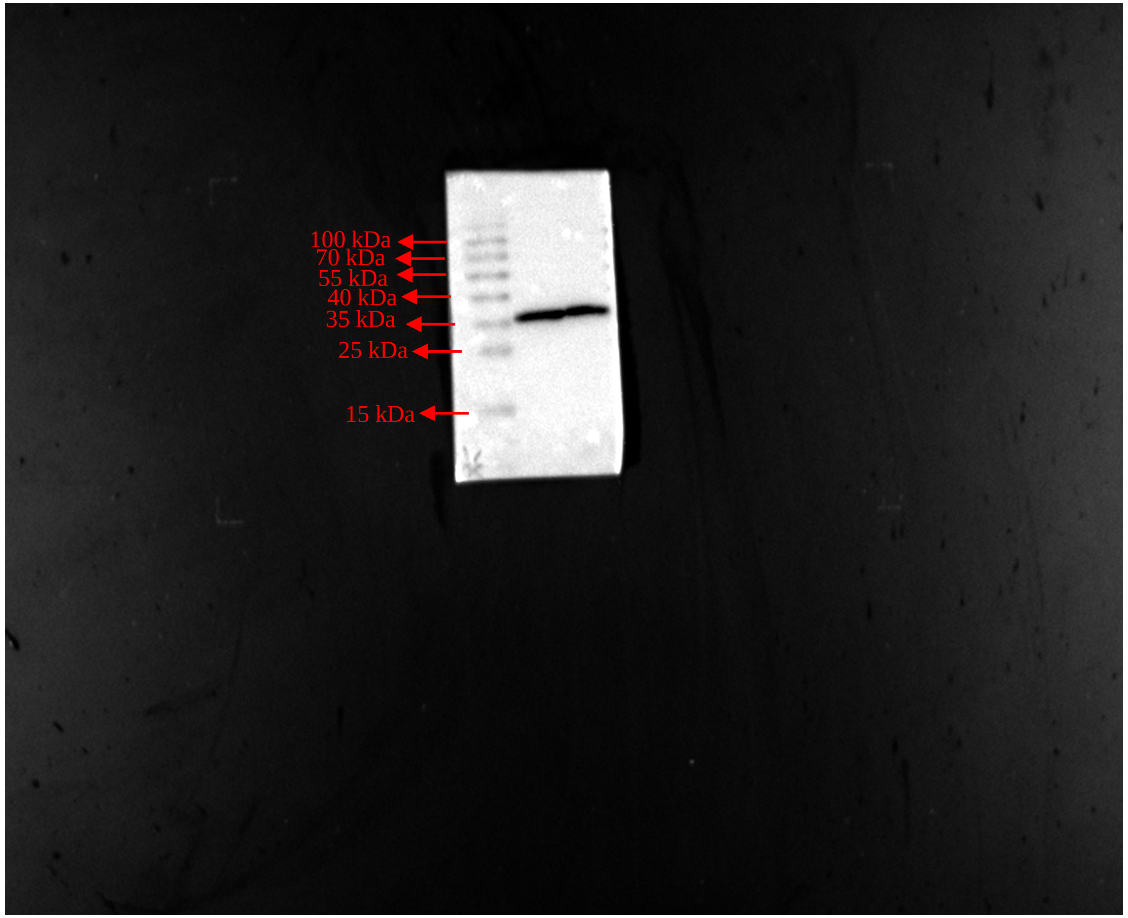

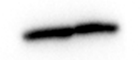

Figure S1C FOXD2

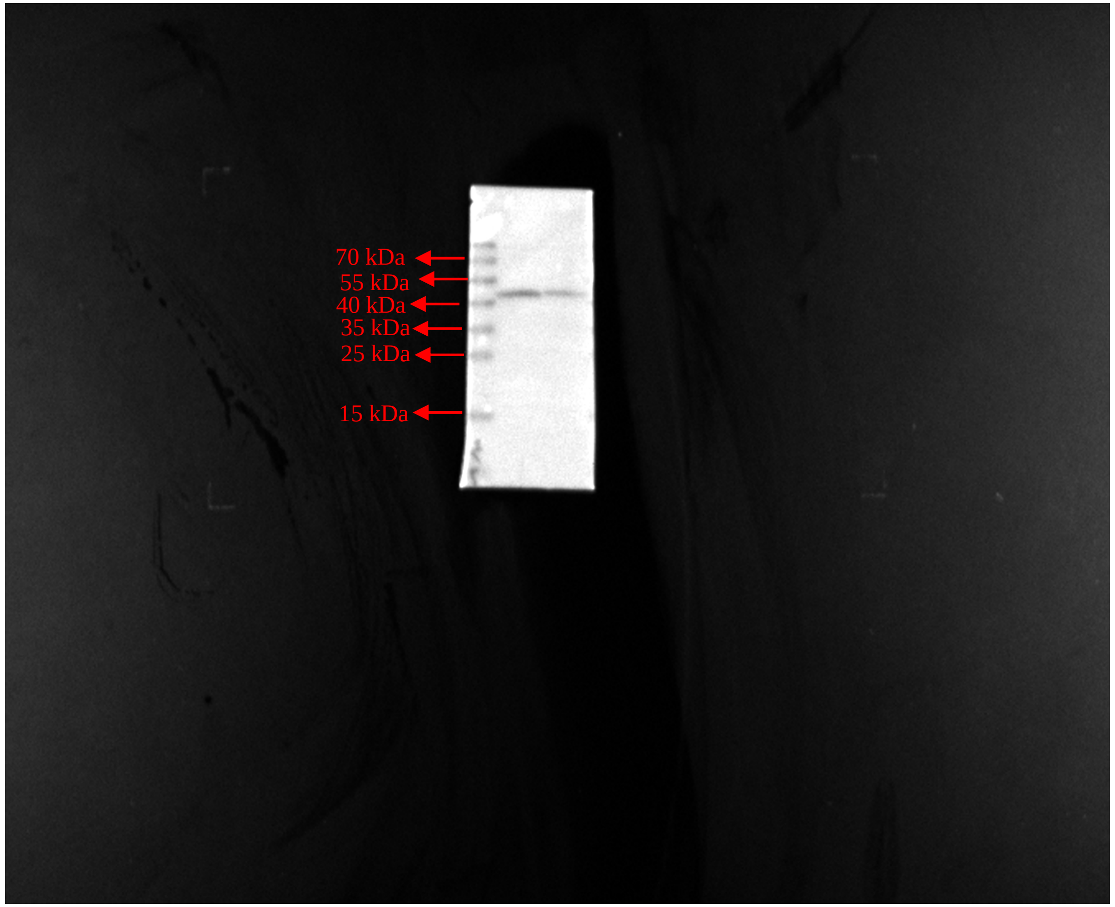

Figure S1C GAPDH

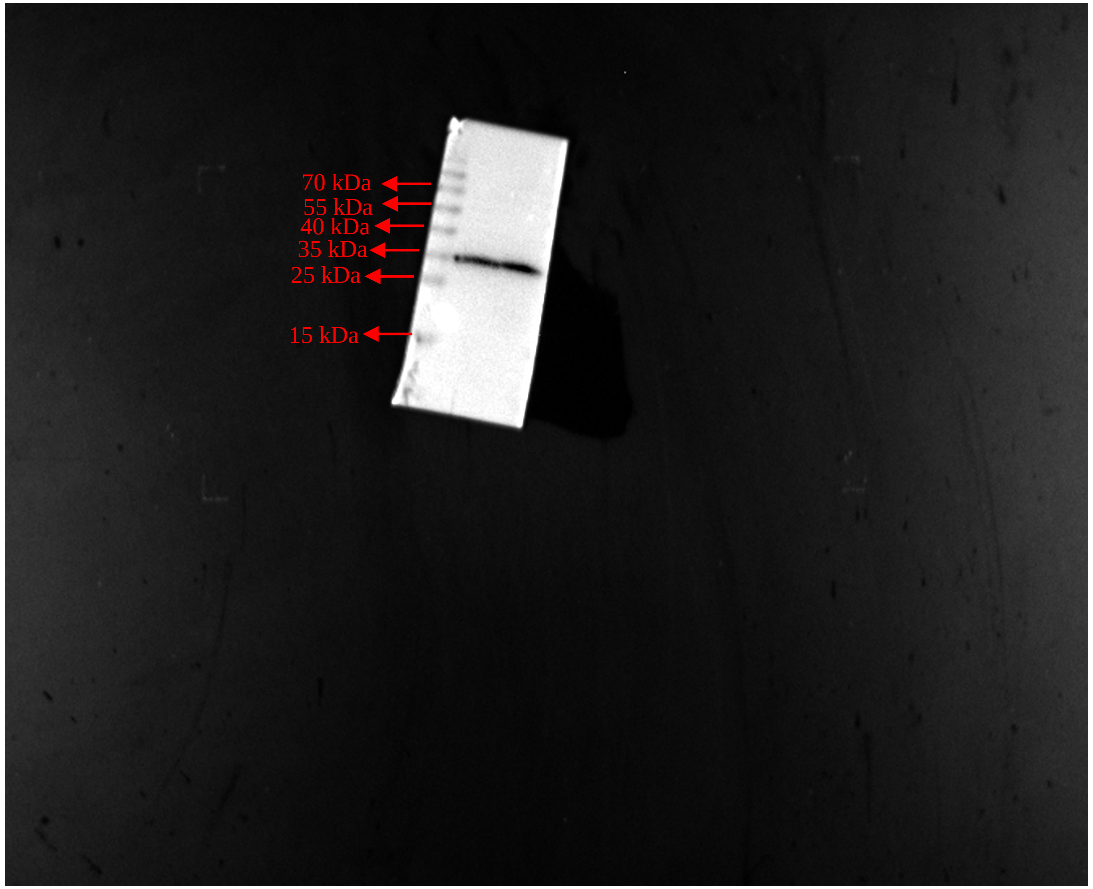

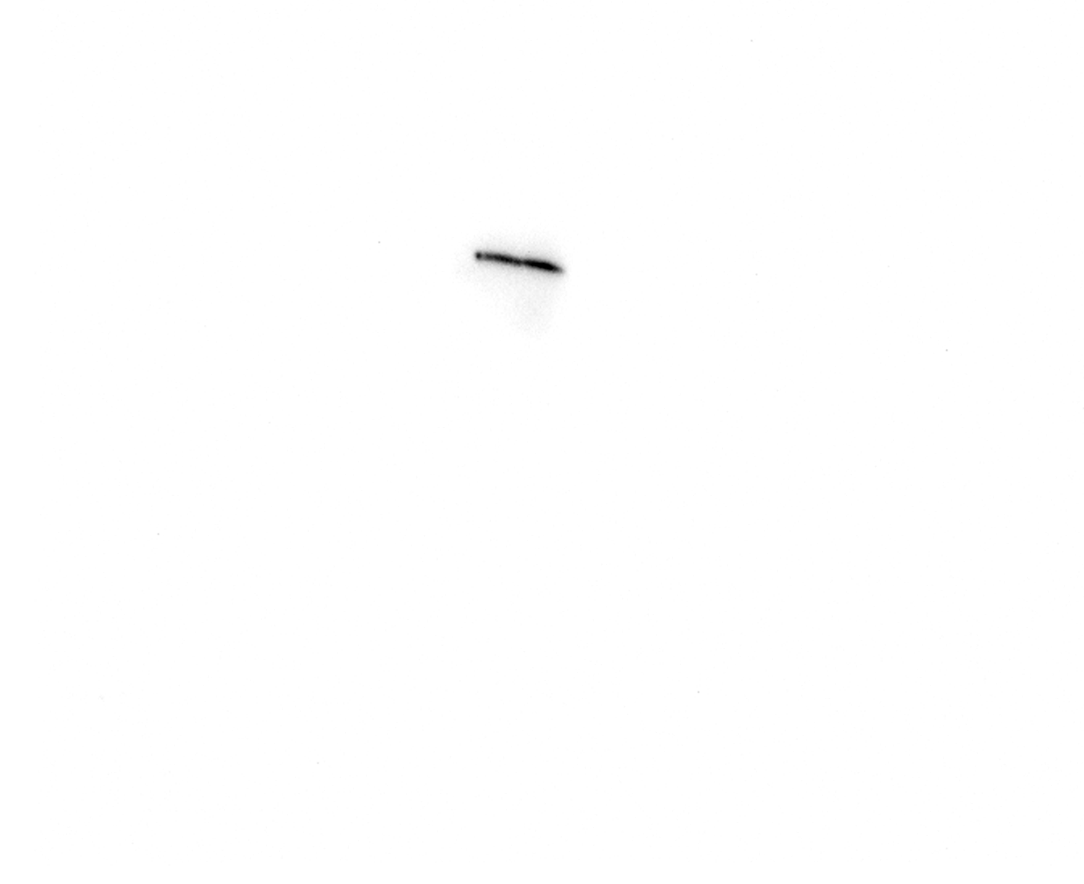

Figure S1C FOXE1

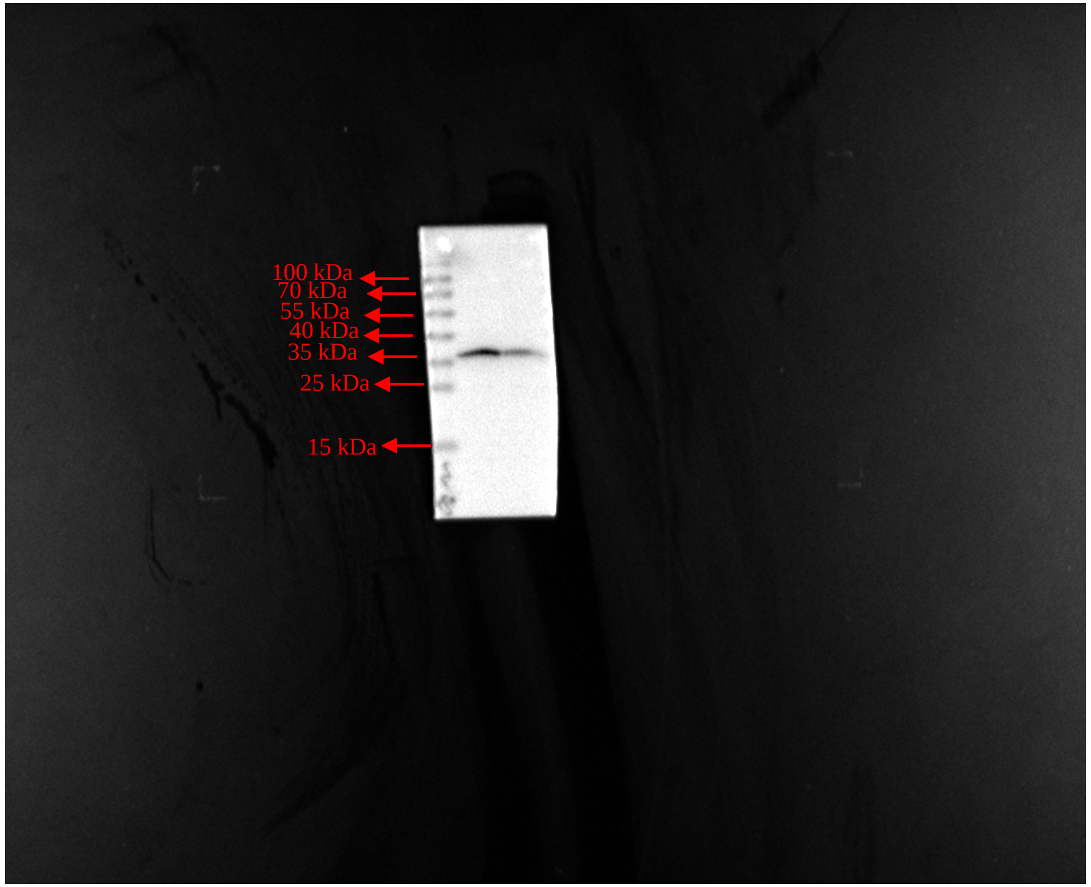

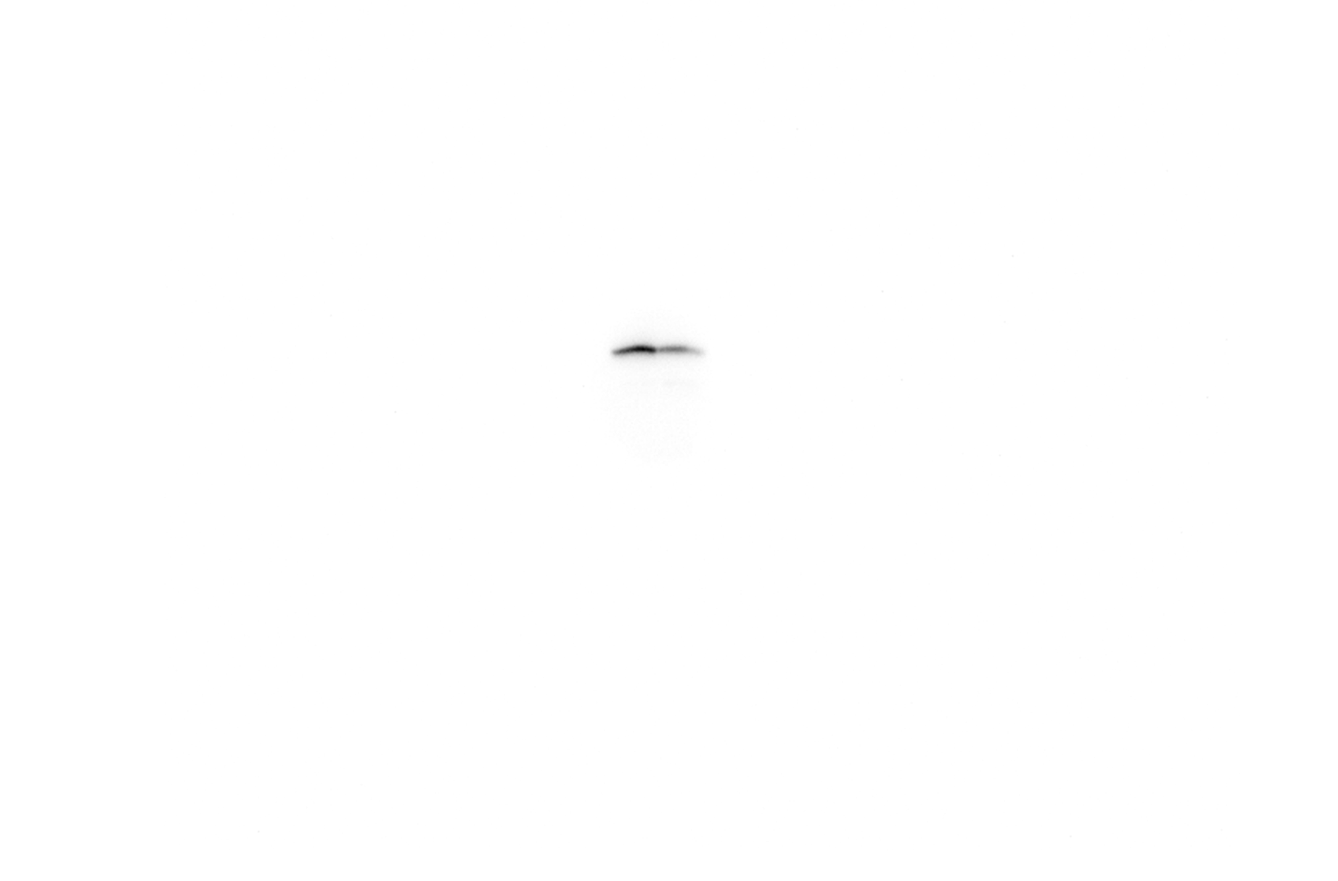

Figure S1C GAPDH

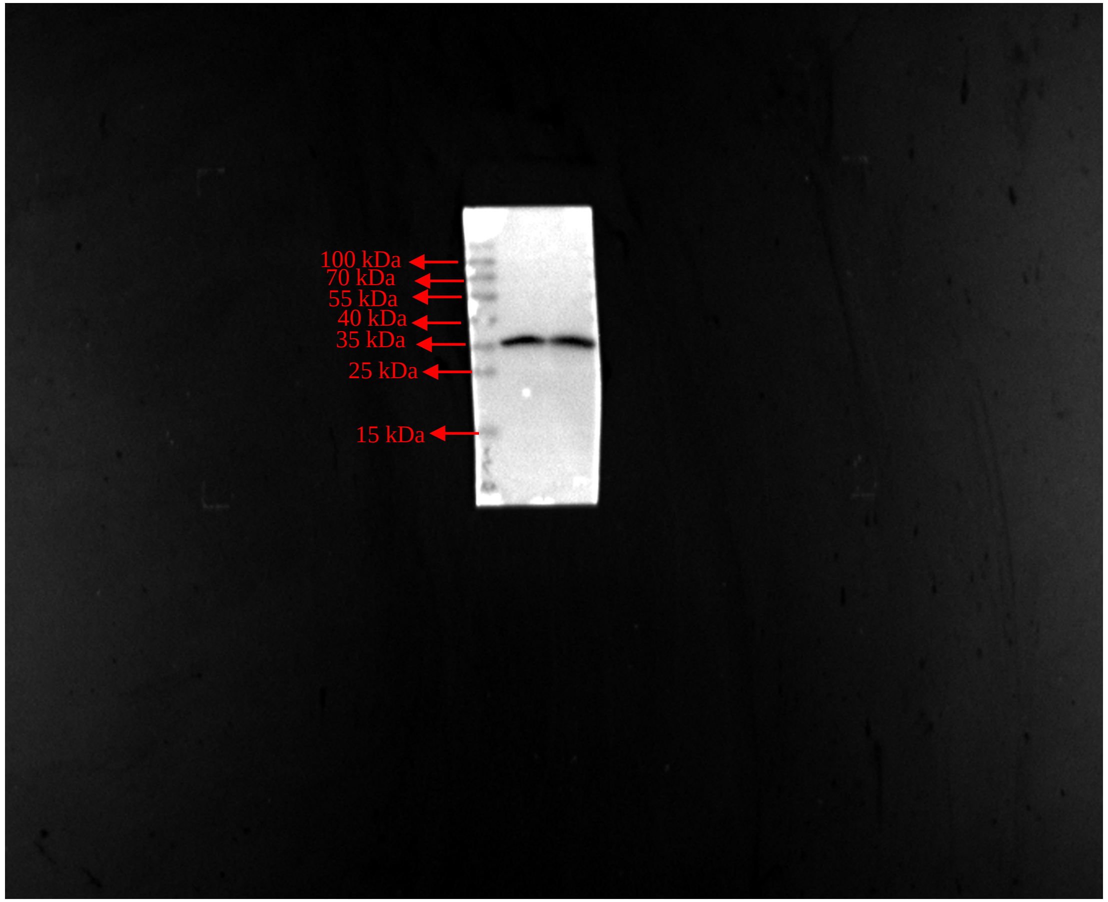

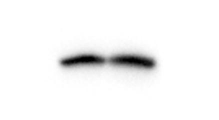

Figure S1C FOXE3

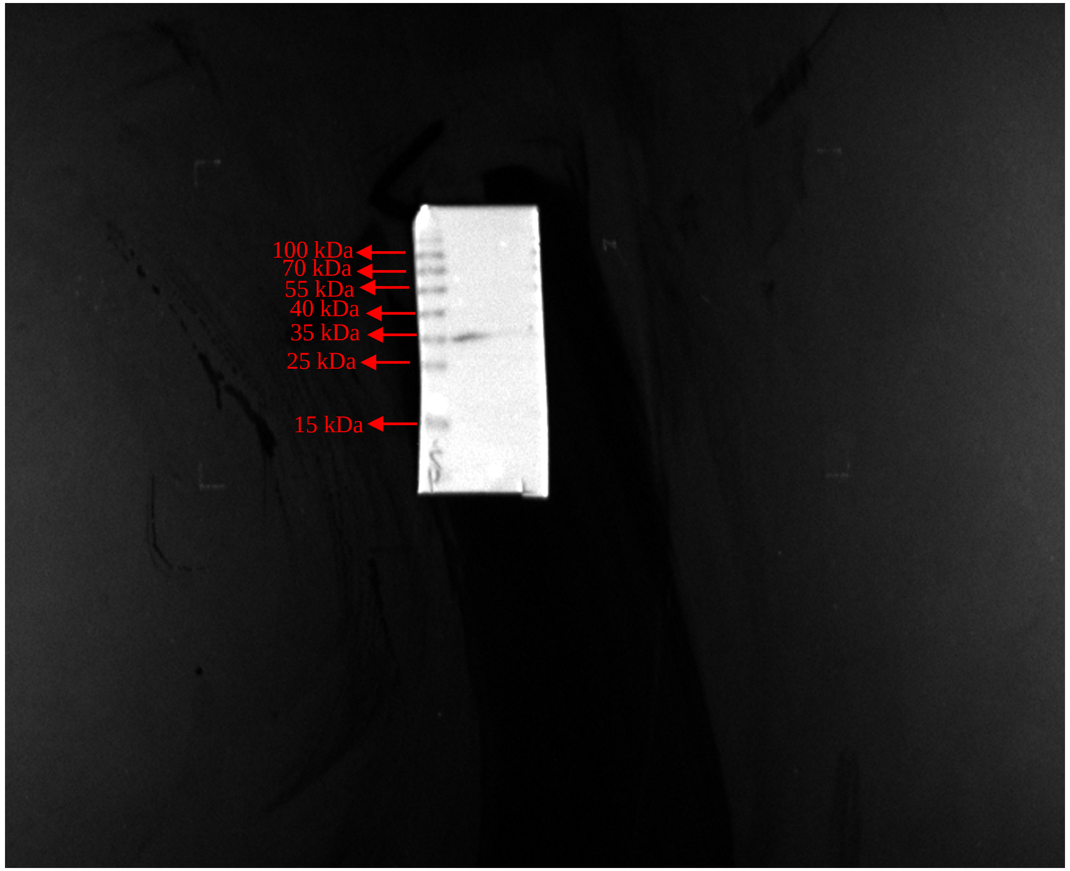

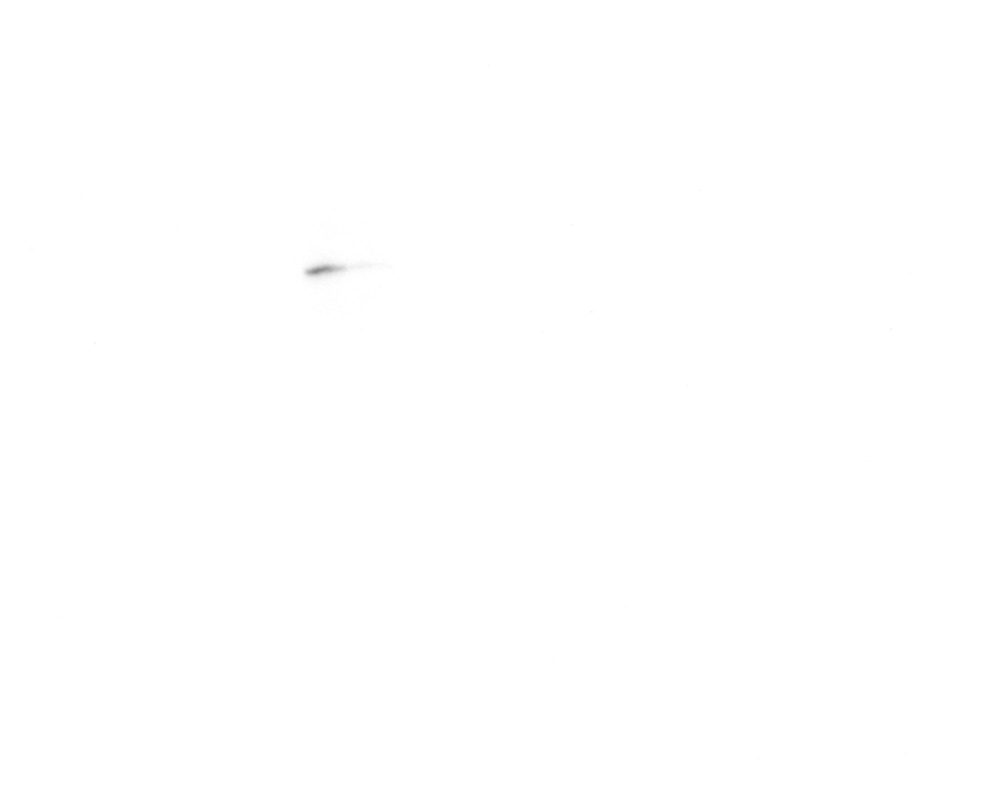

Figure S1C GAPDH

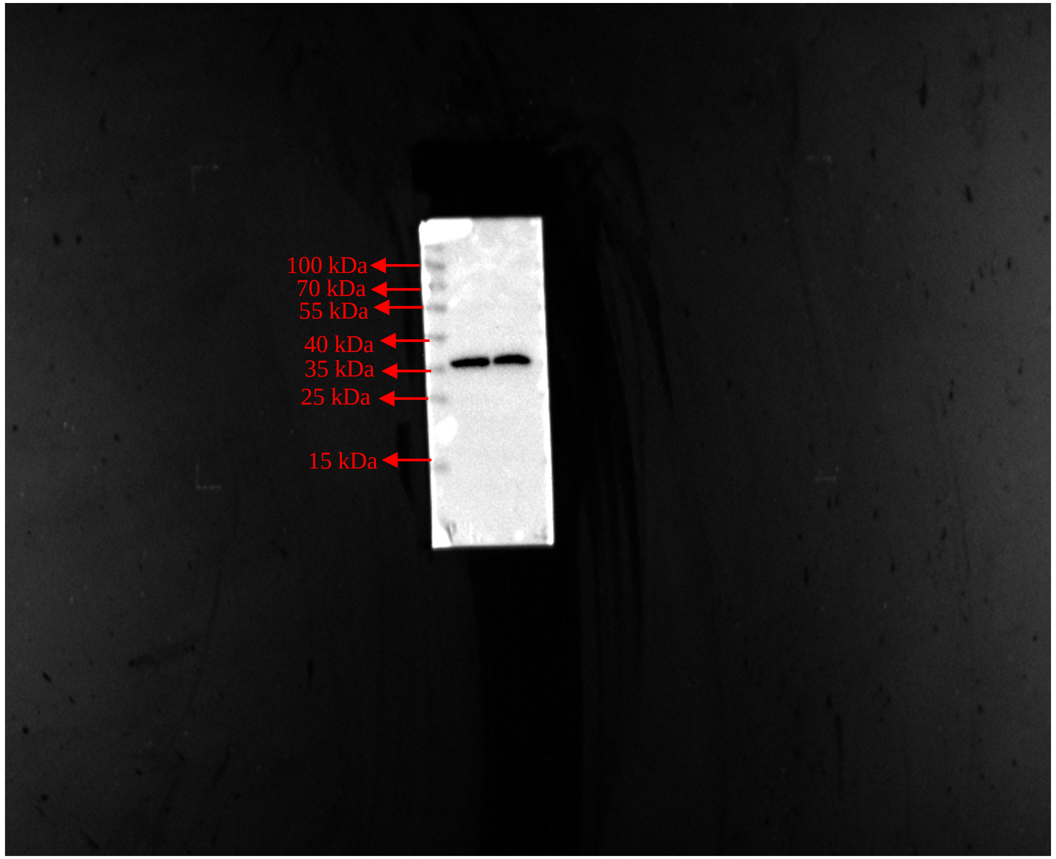

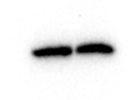

Figure S1C FOXF1

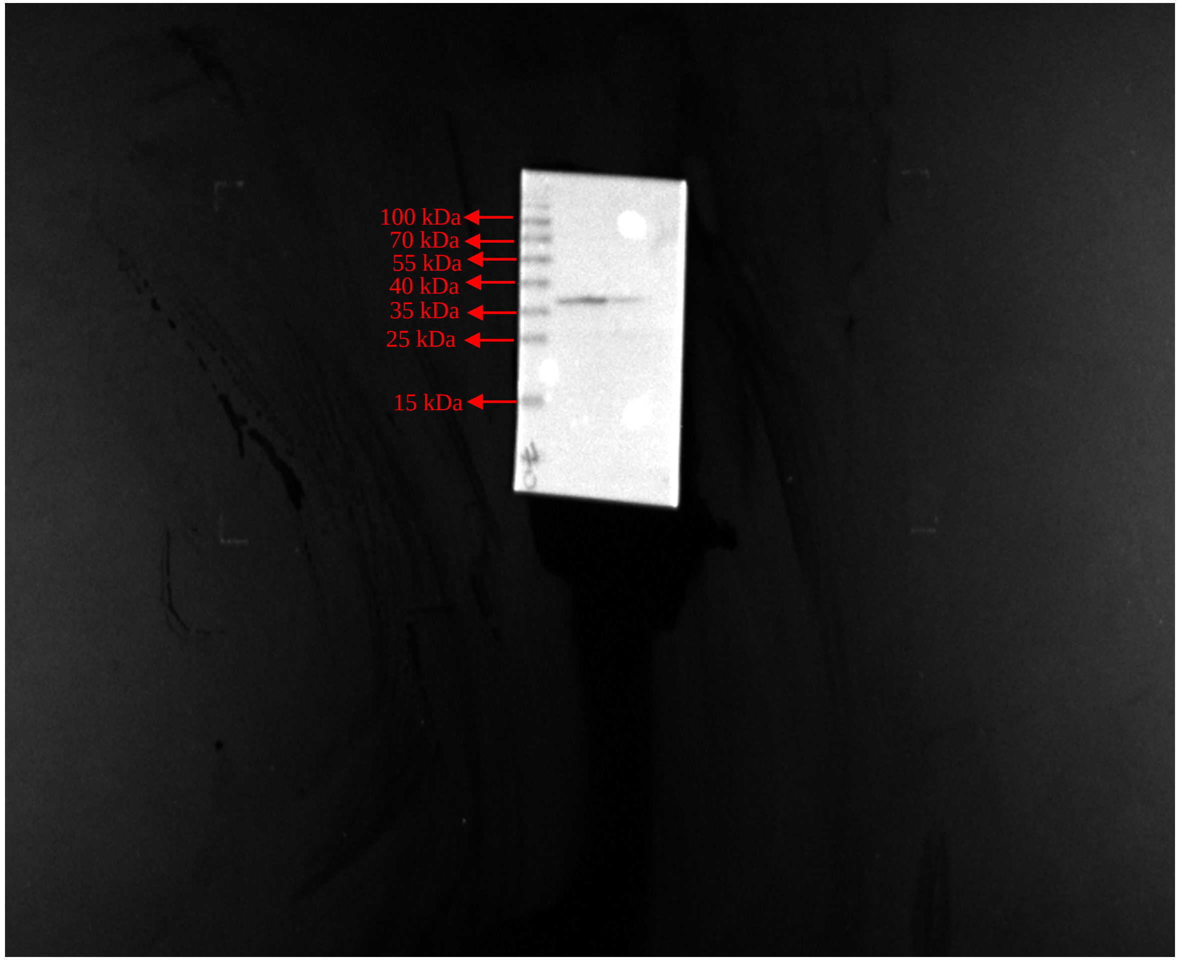

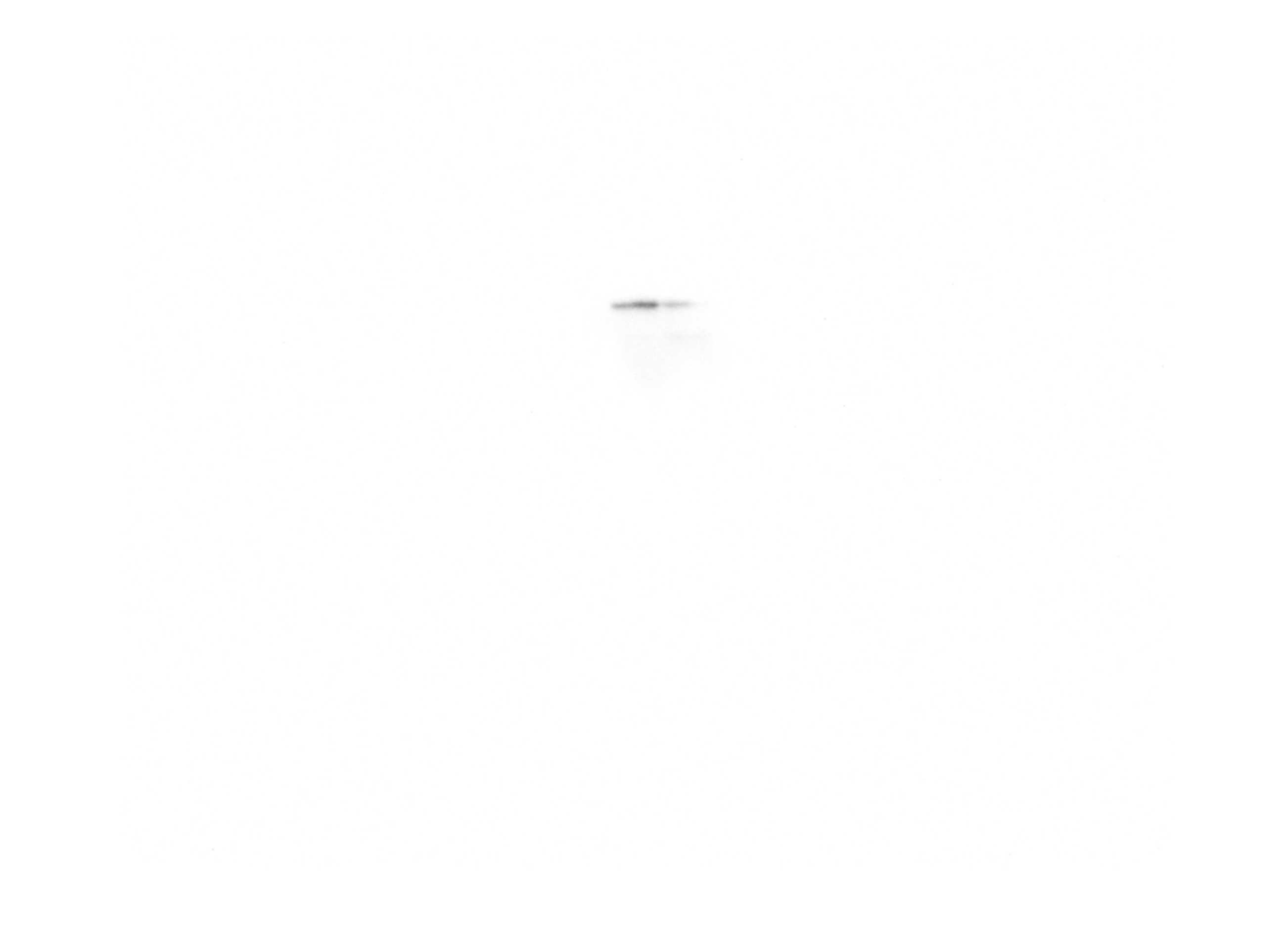

Figure S1C GAPDH

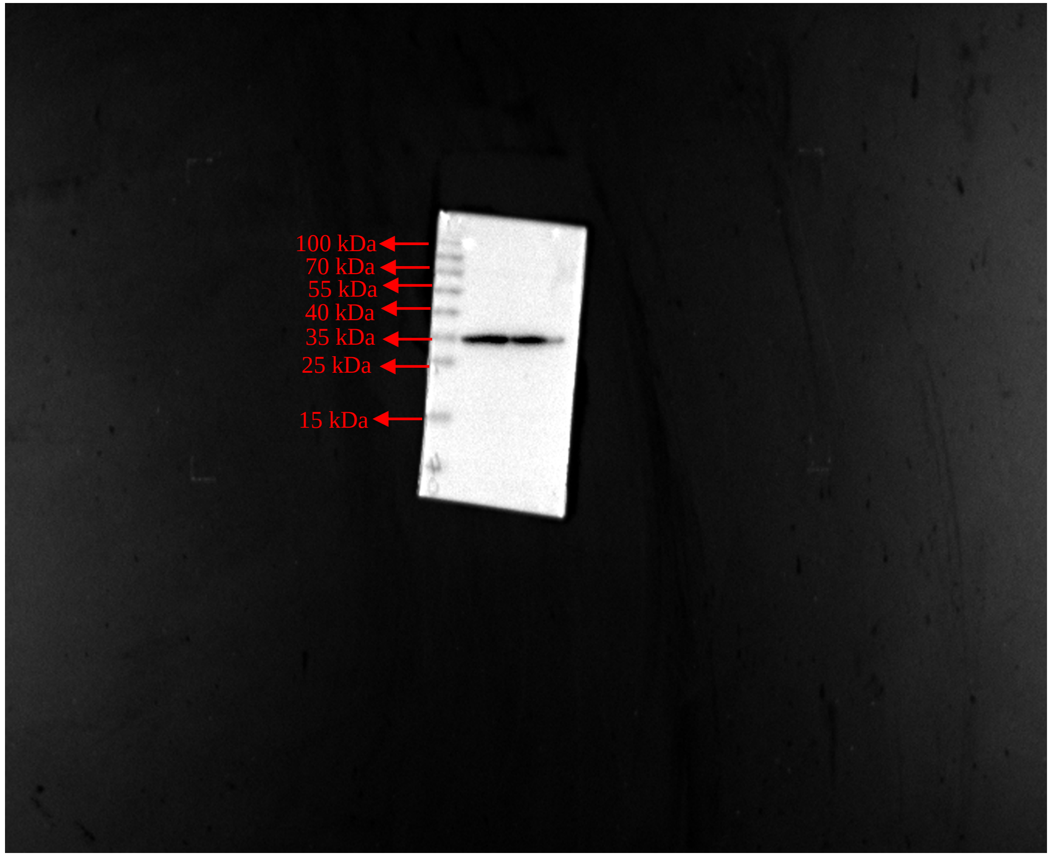

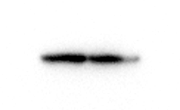

Figure S1C FOXH1

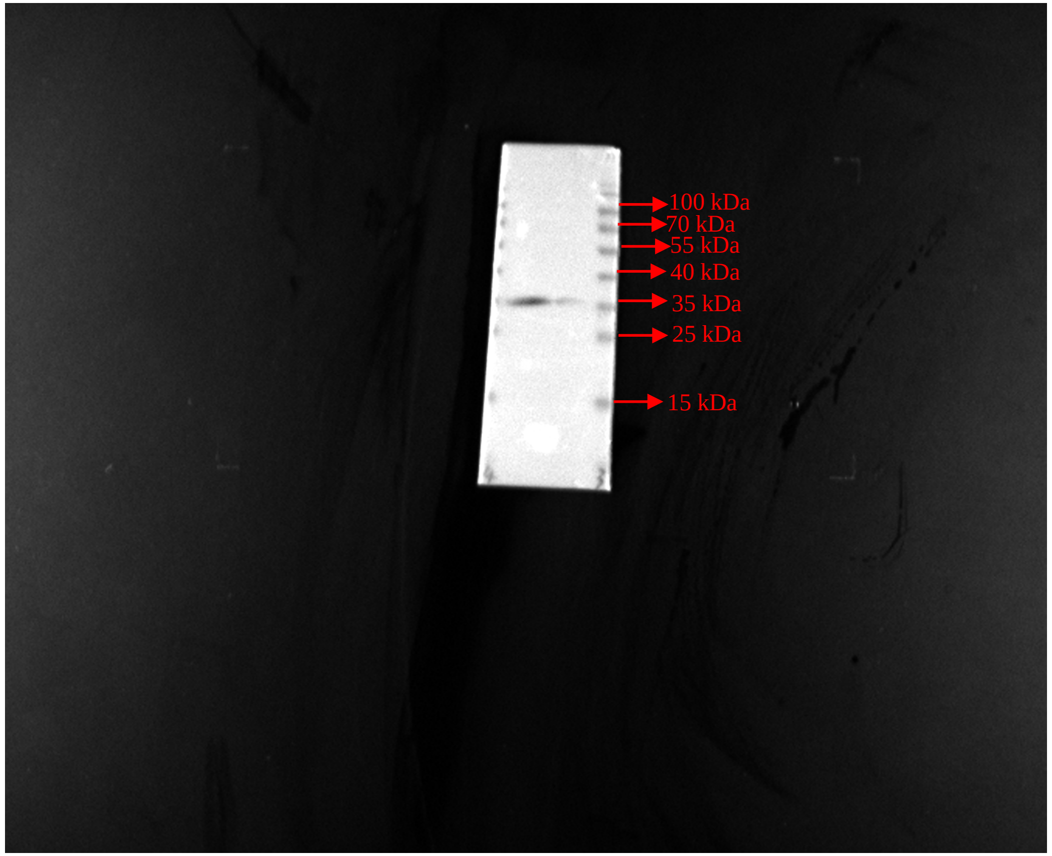

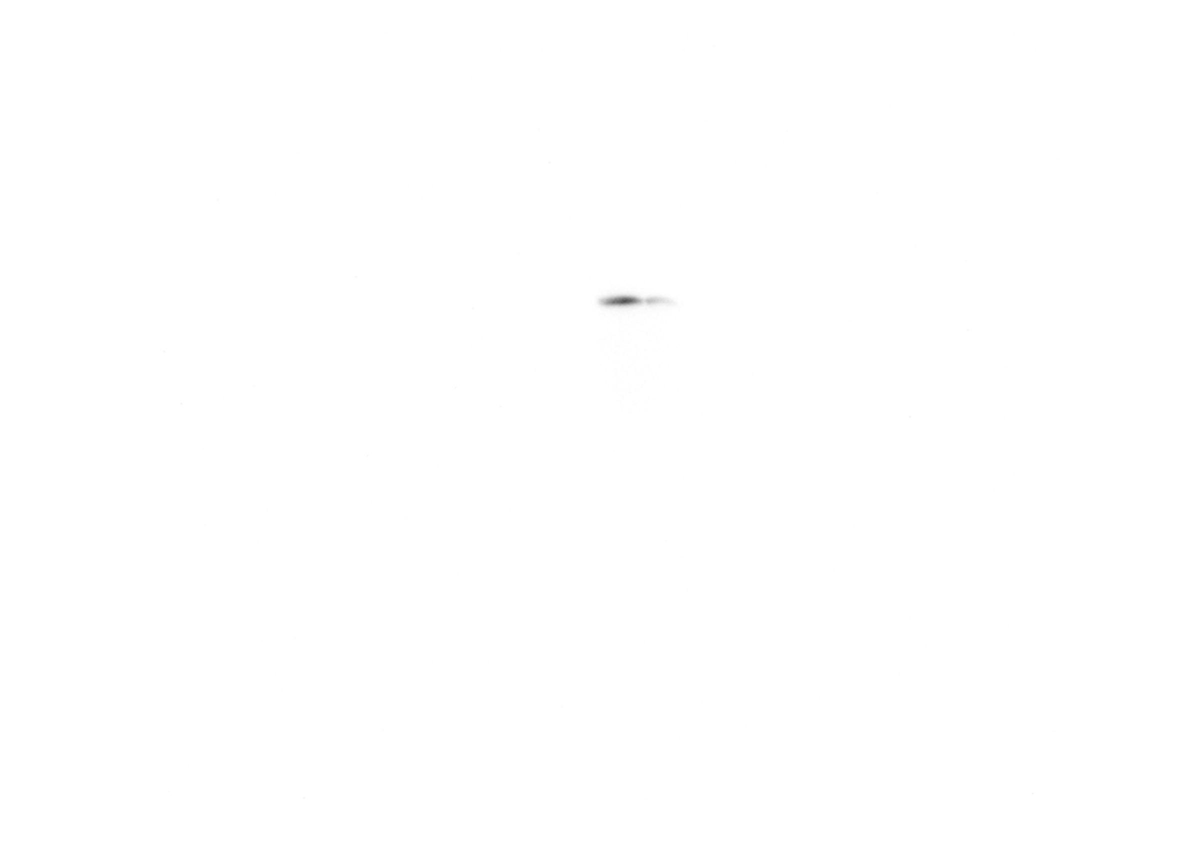

Figure S1C GAPDH

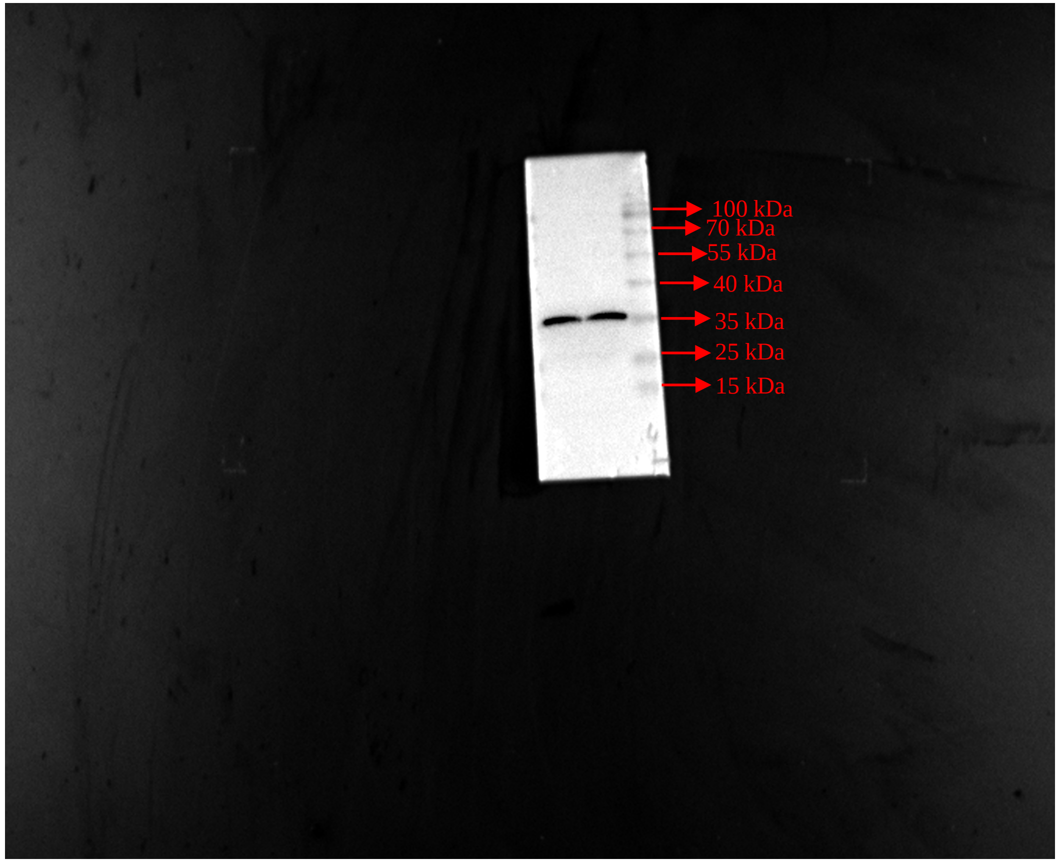

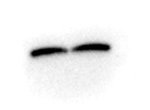

Figure S1C FOXS1

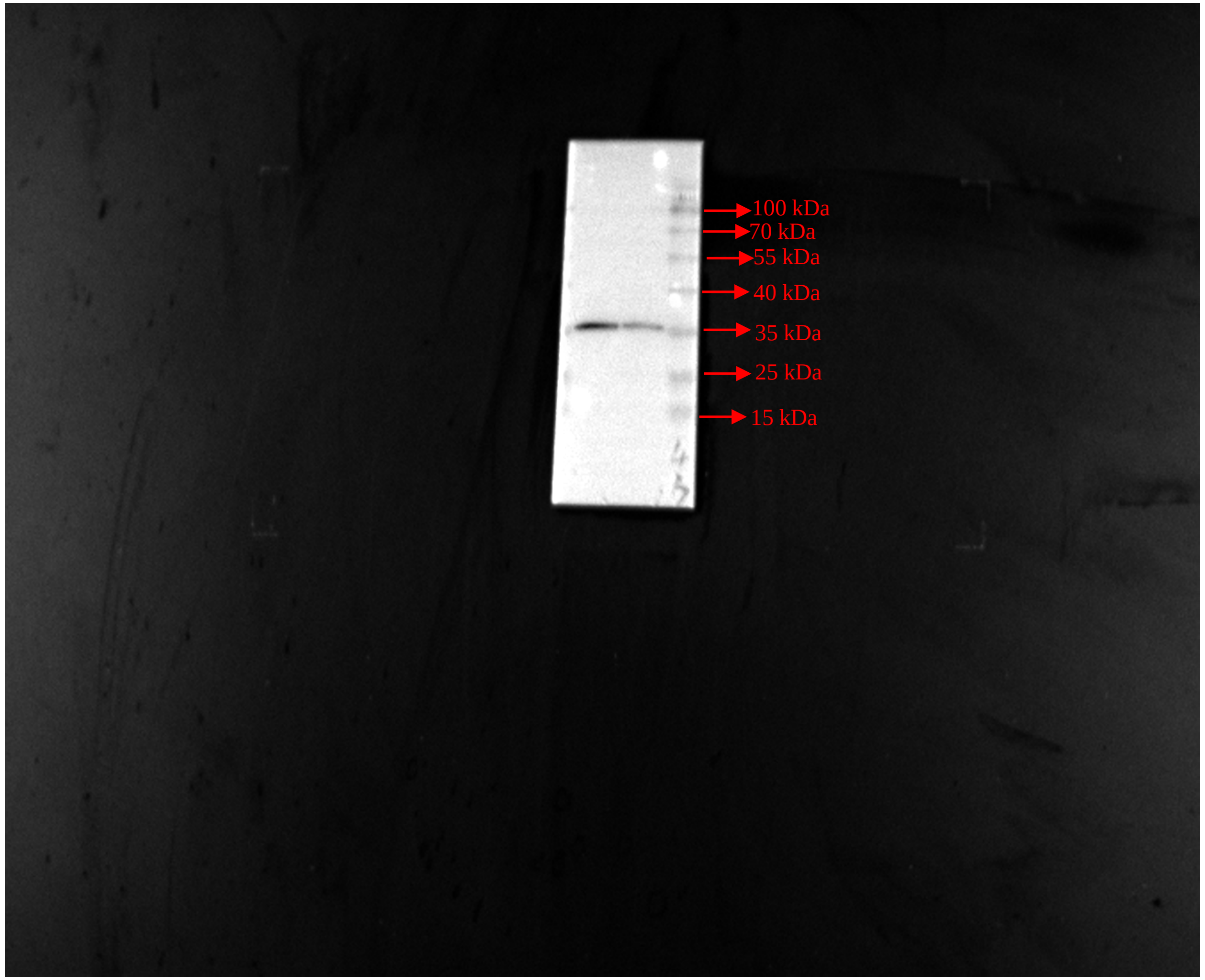

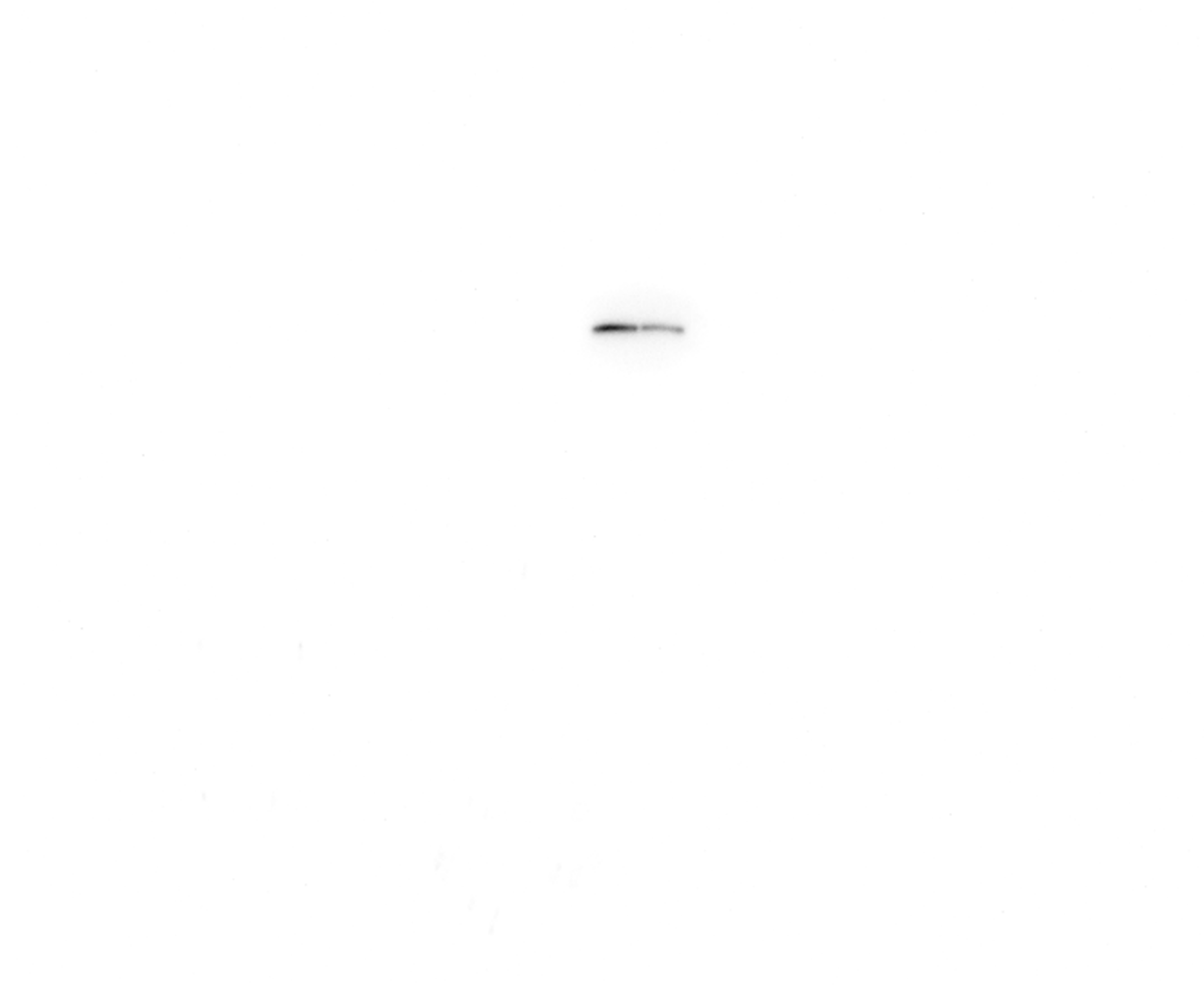

Figure S1C GAPDH

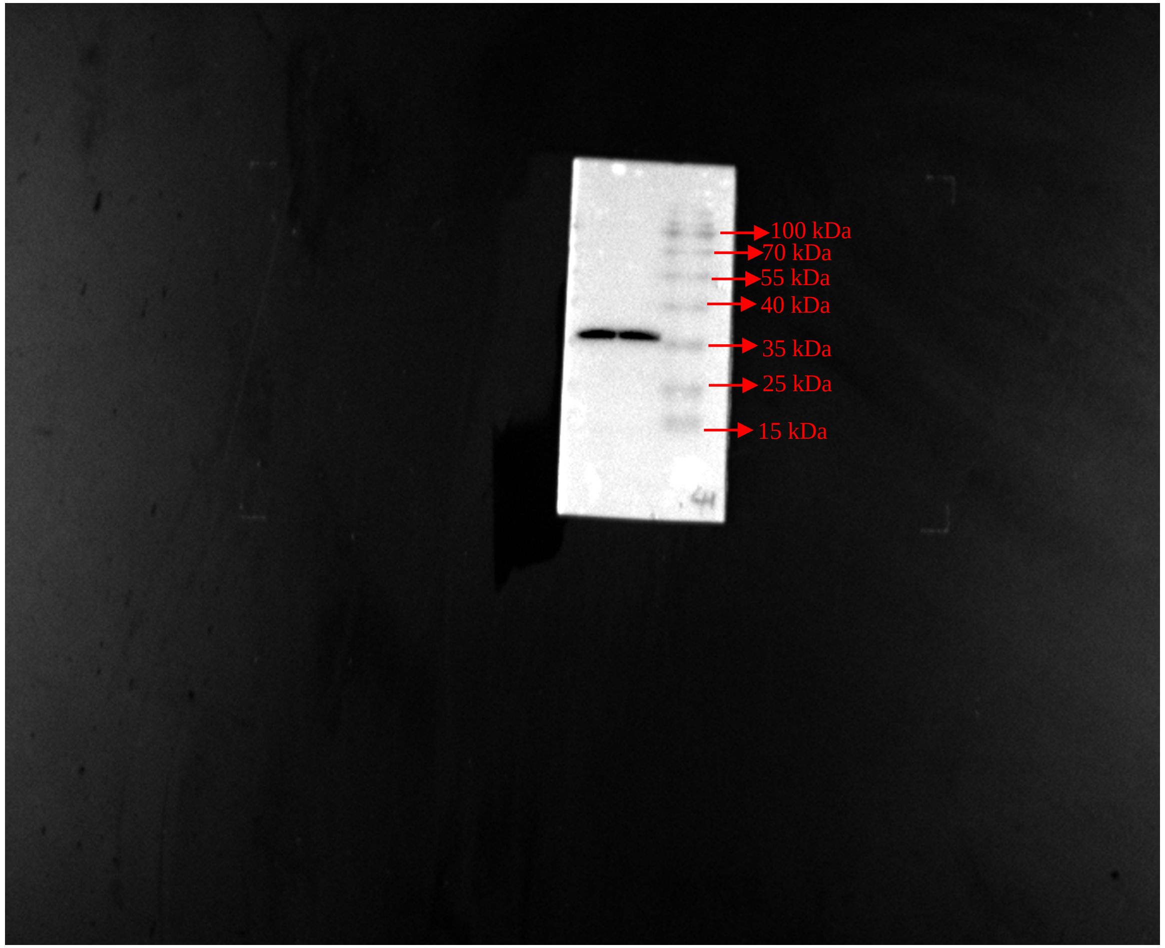

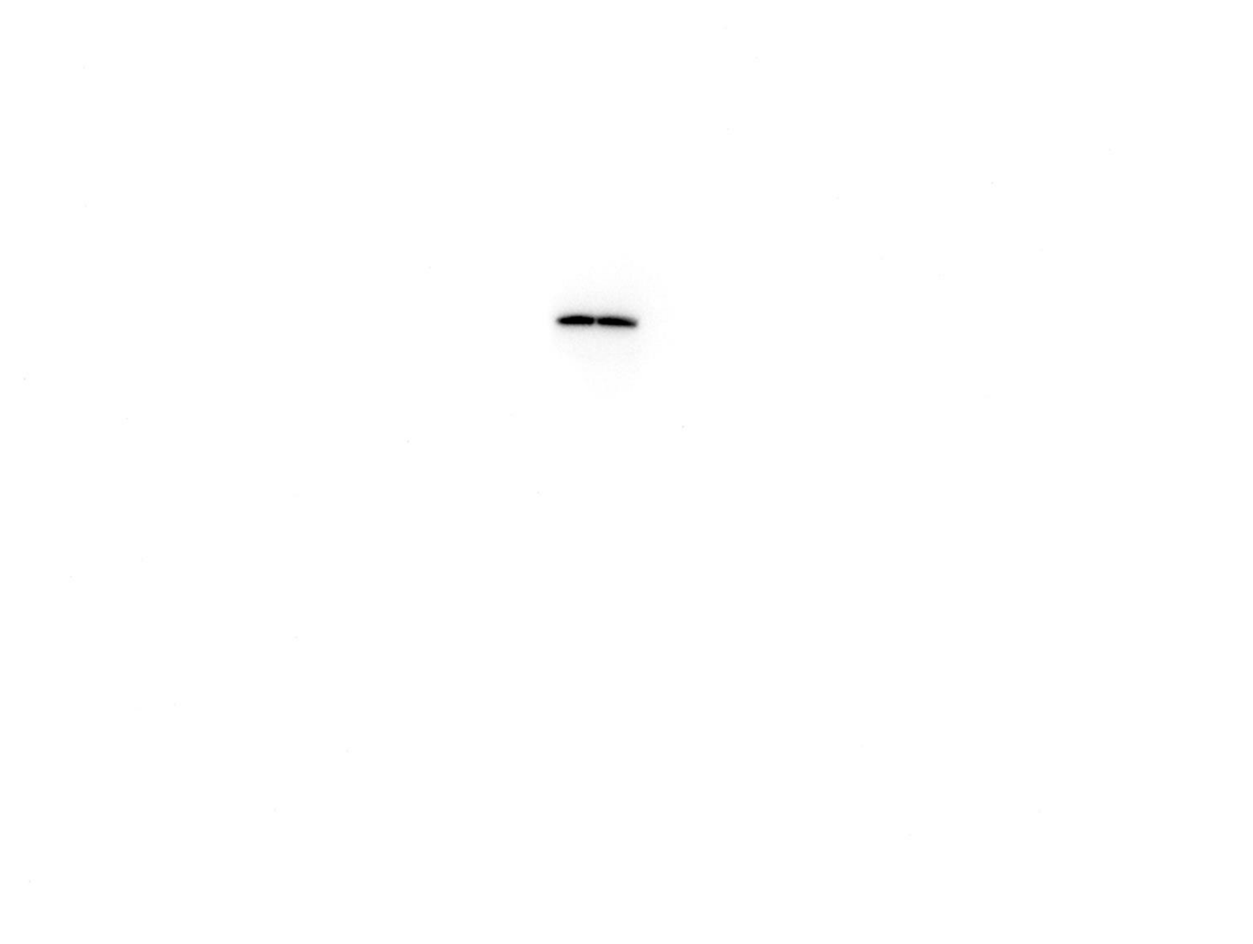

Figure S1C FOXI3

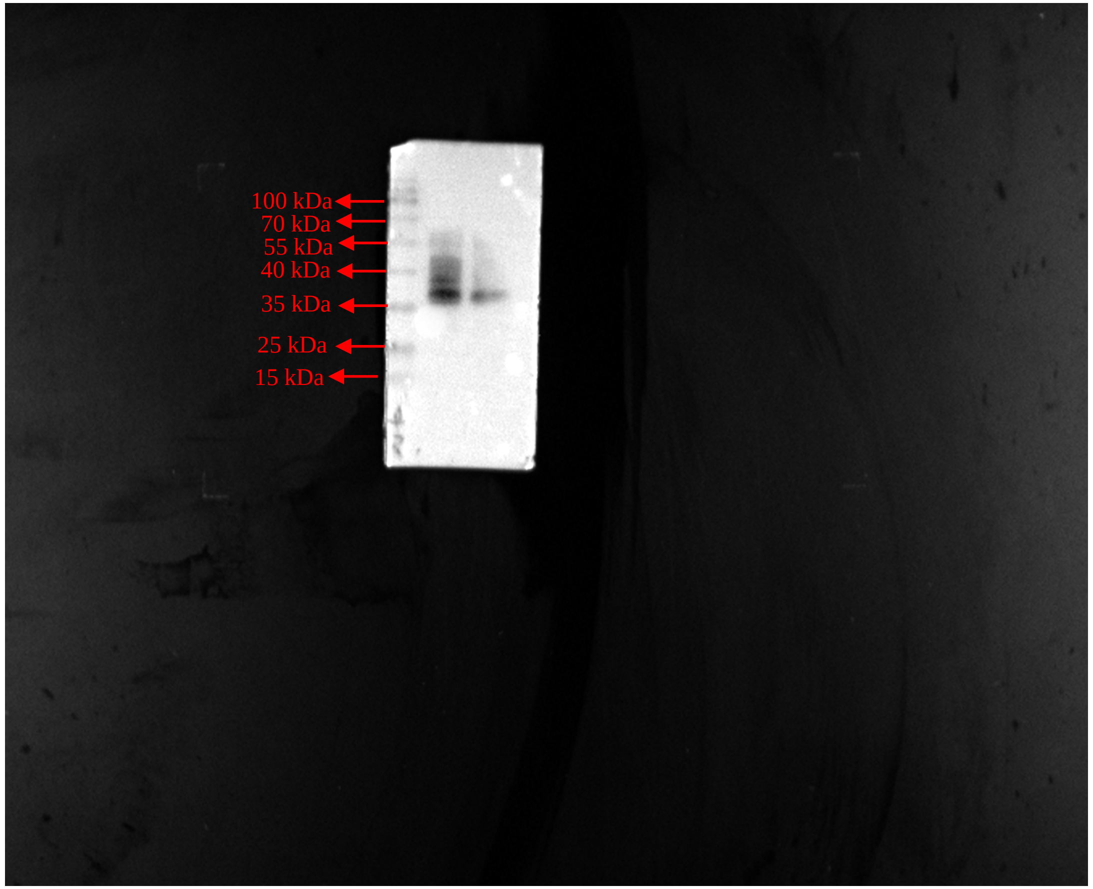

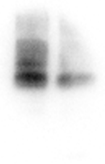

Figure S1C GAPDH

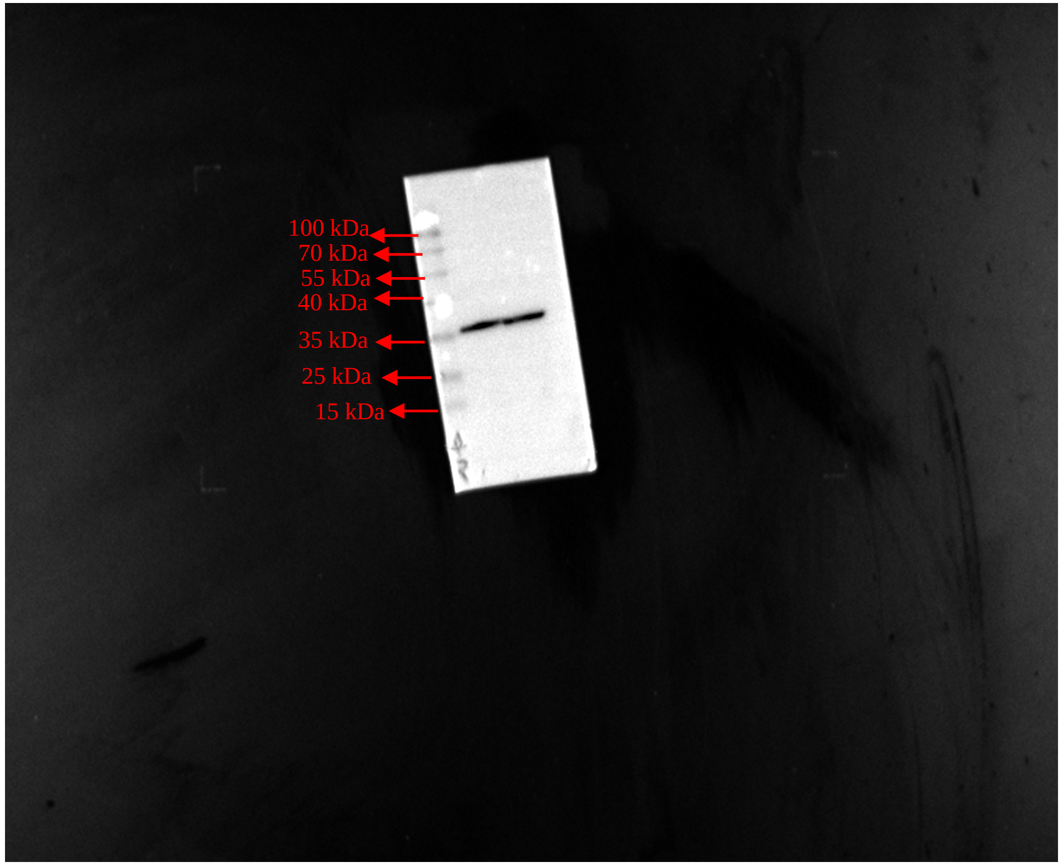

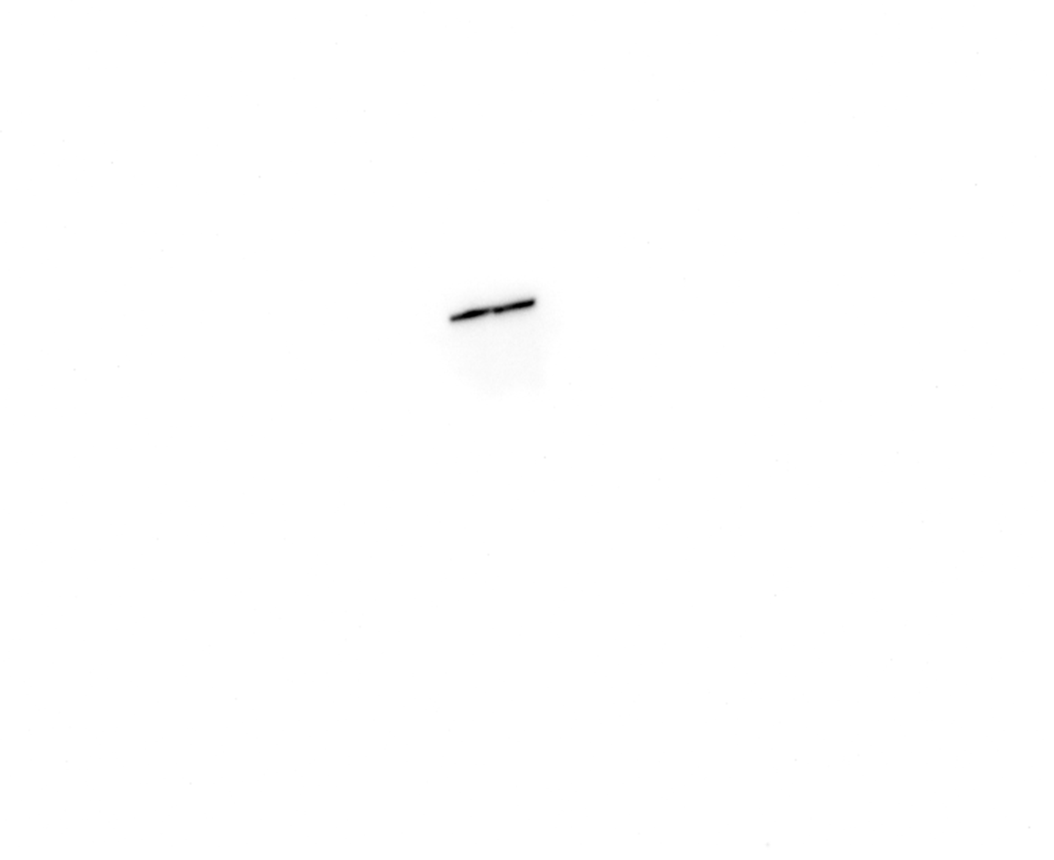

Figure S1C FOXL1

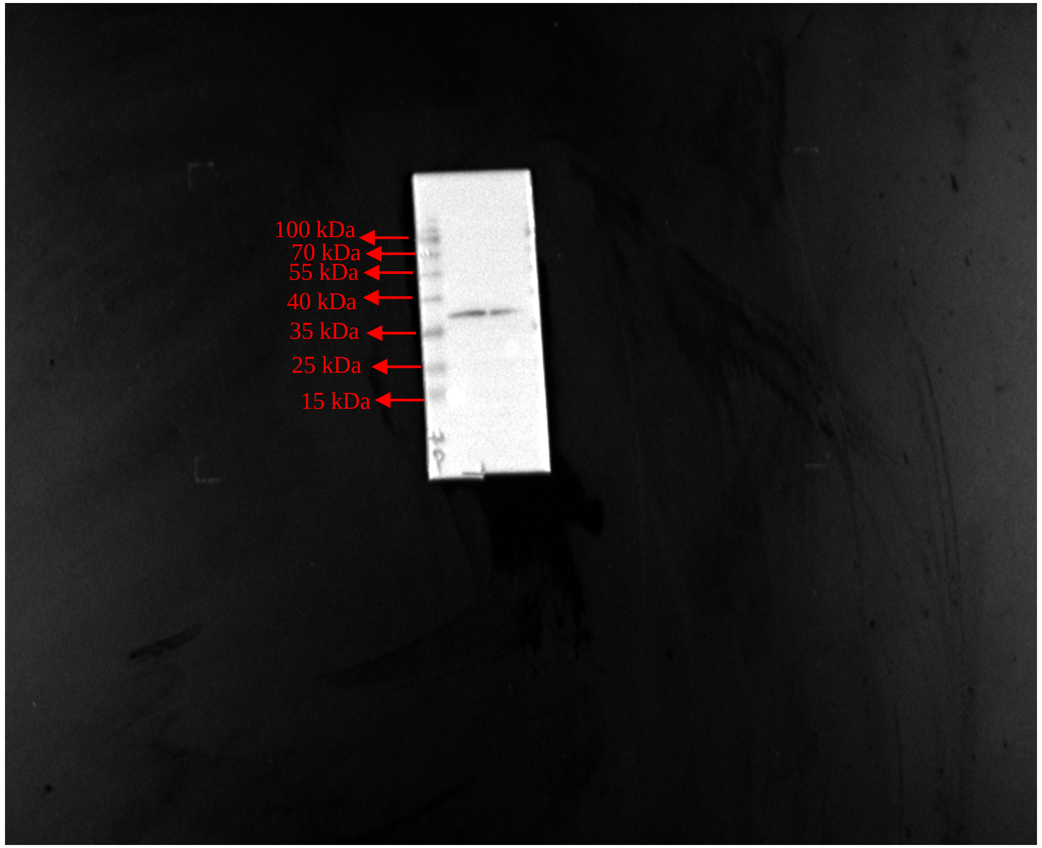

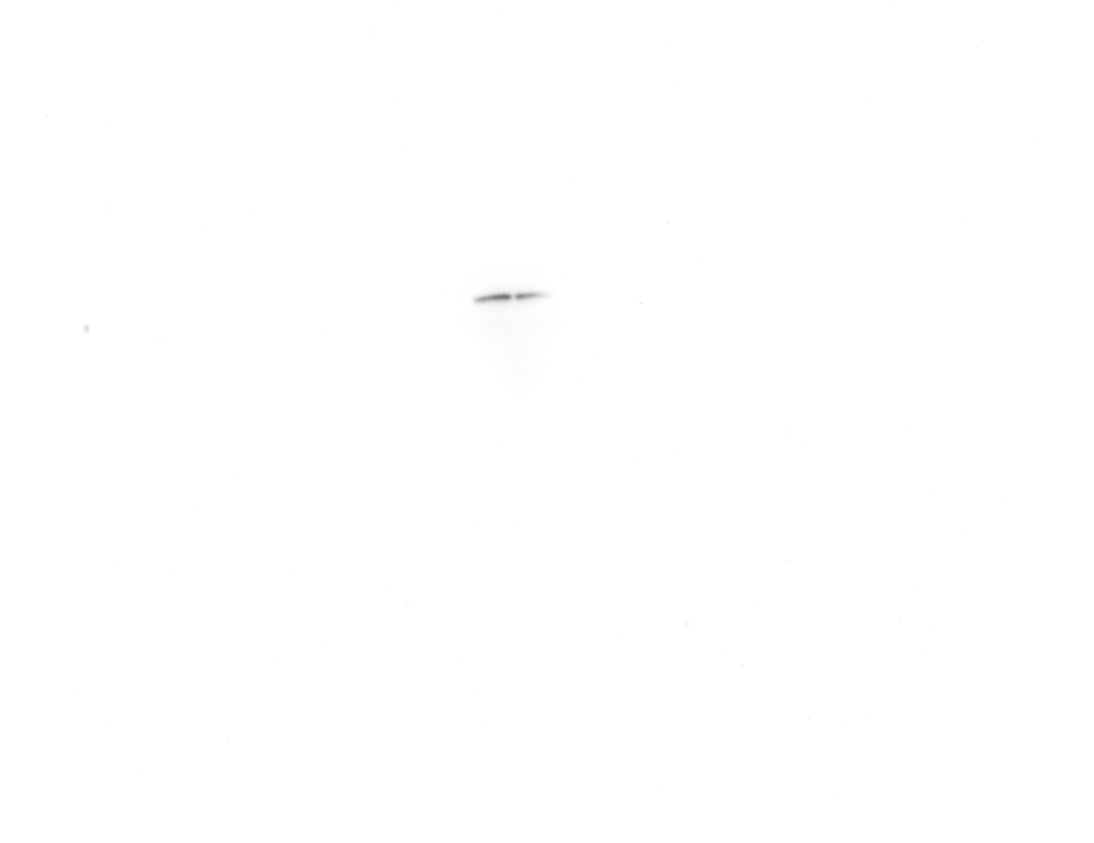

Figure S1C GAPDH

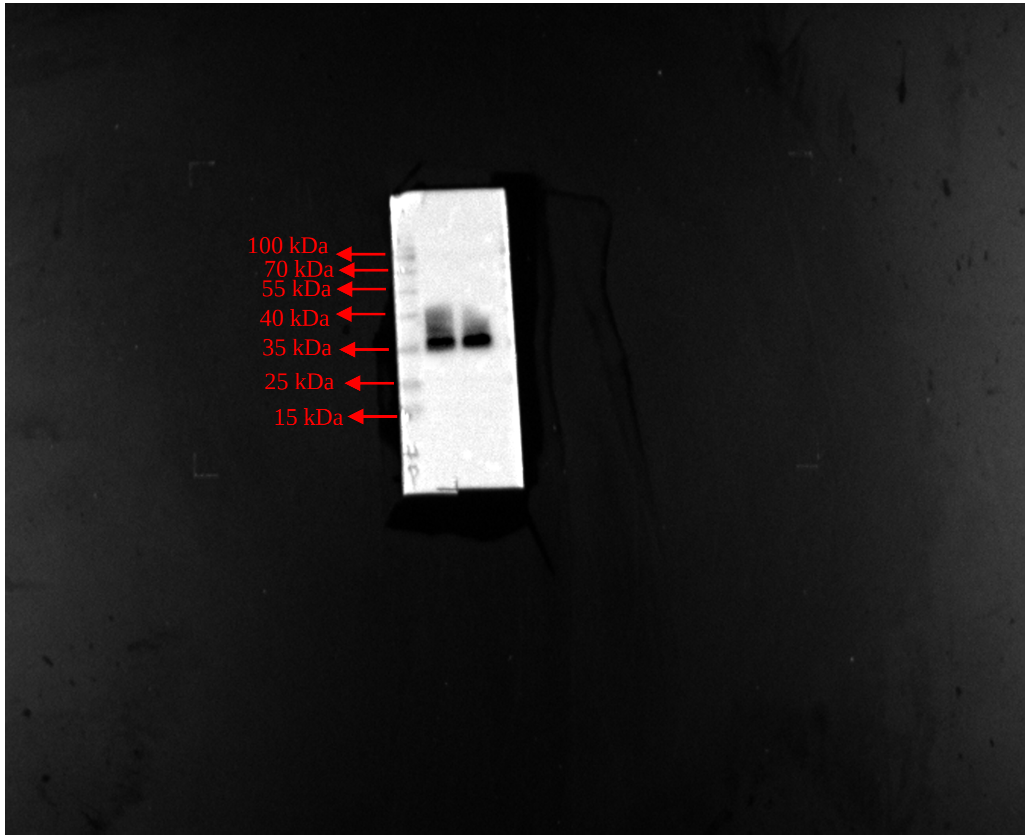

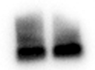

Figure S1C FOXL1

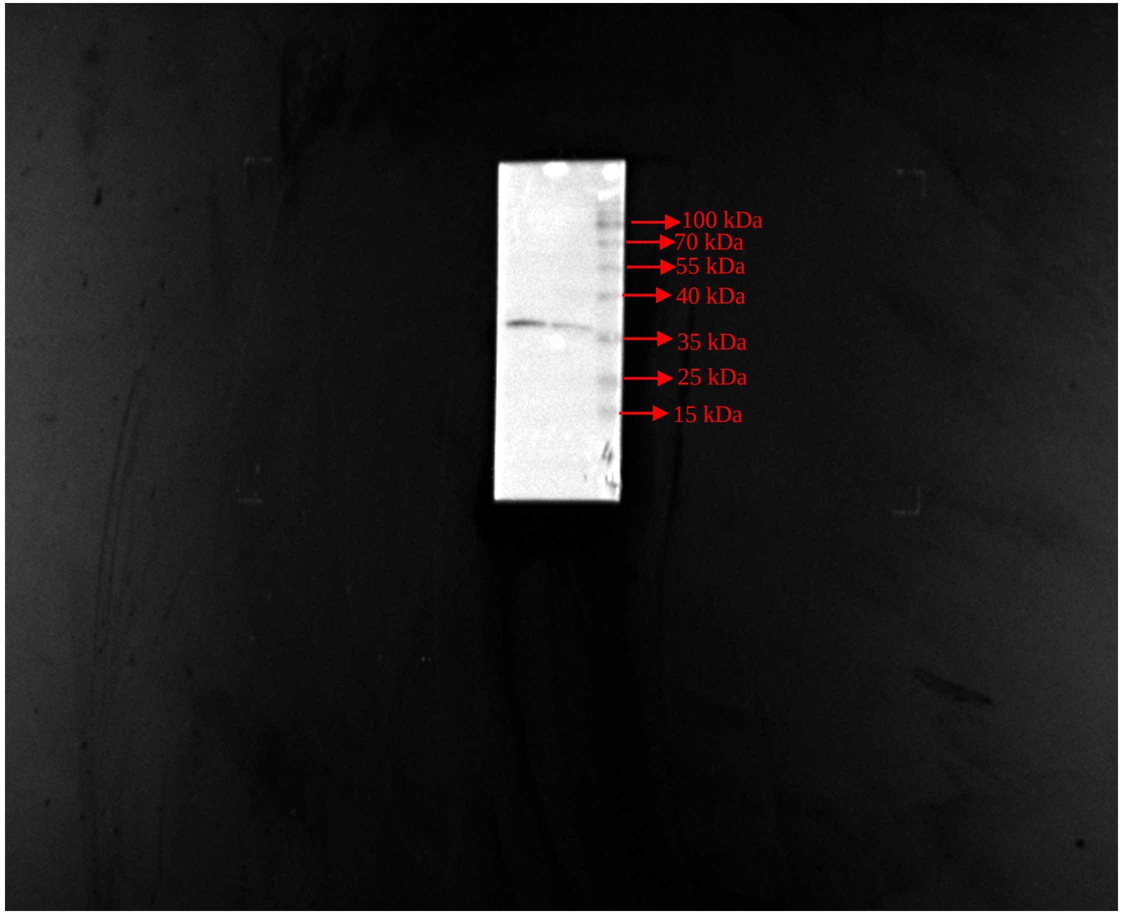

Figure S1C GAPDH

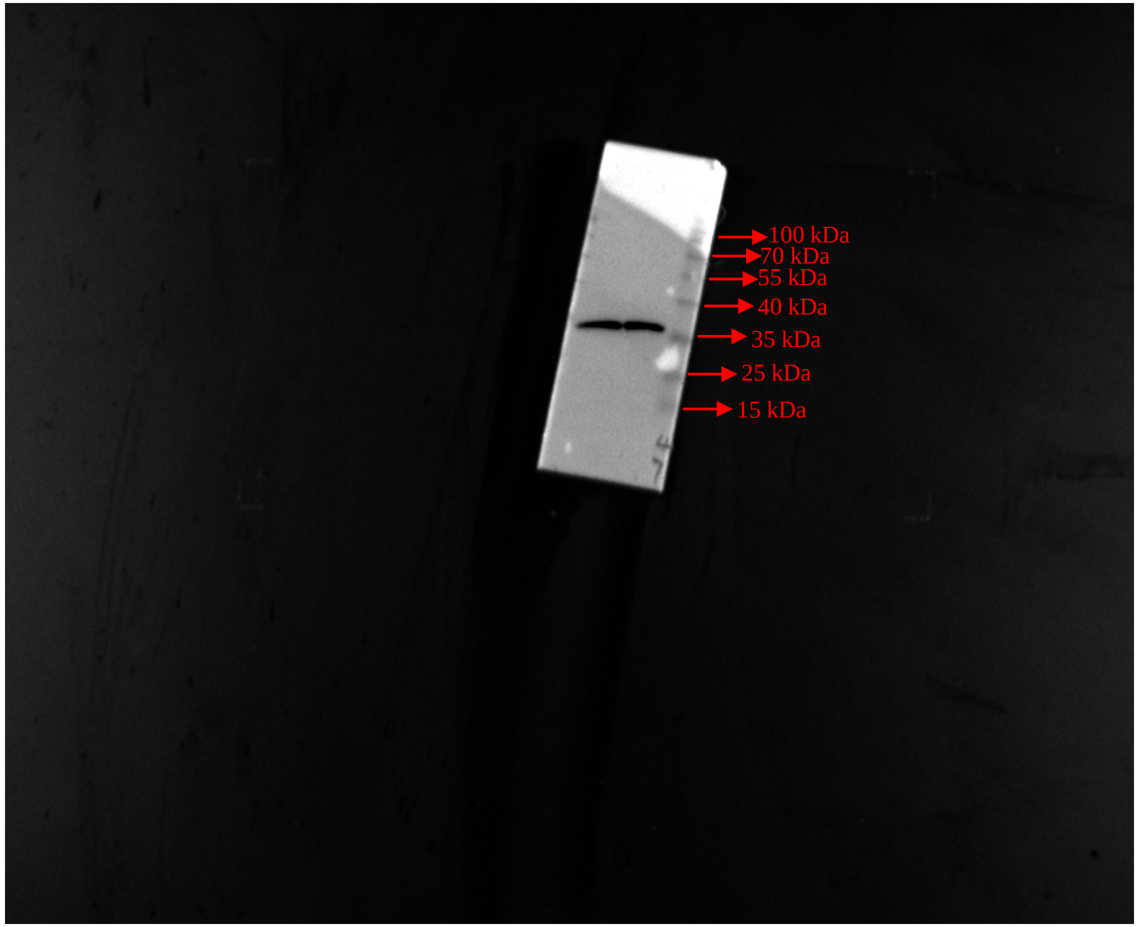

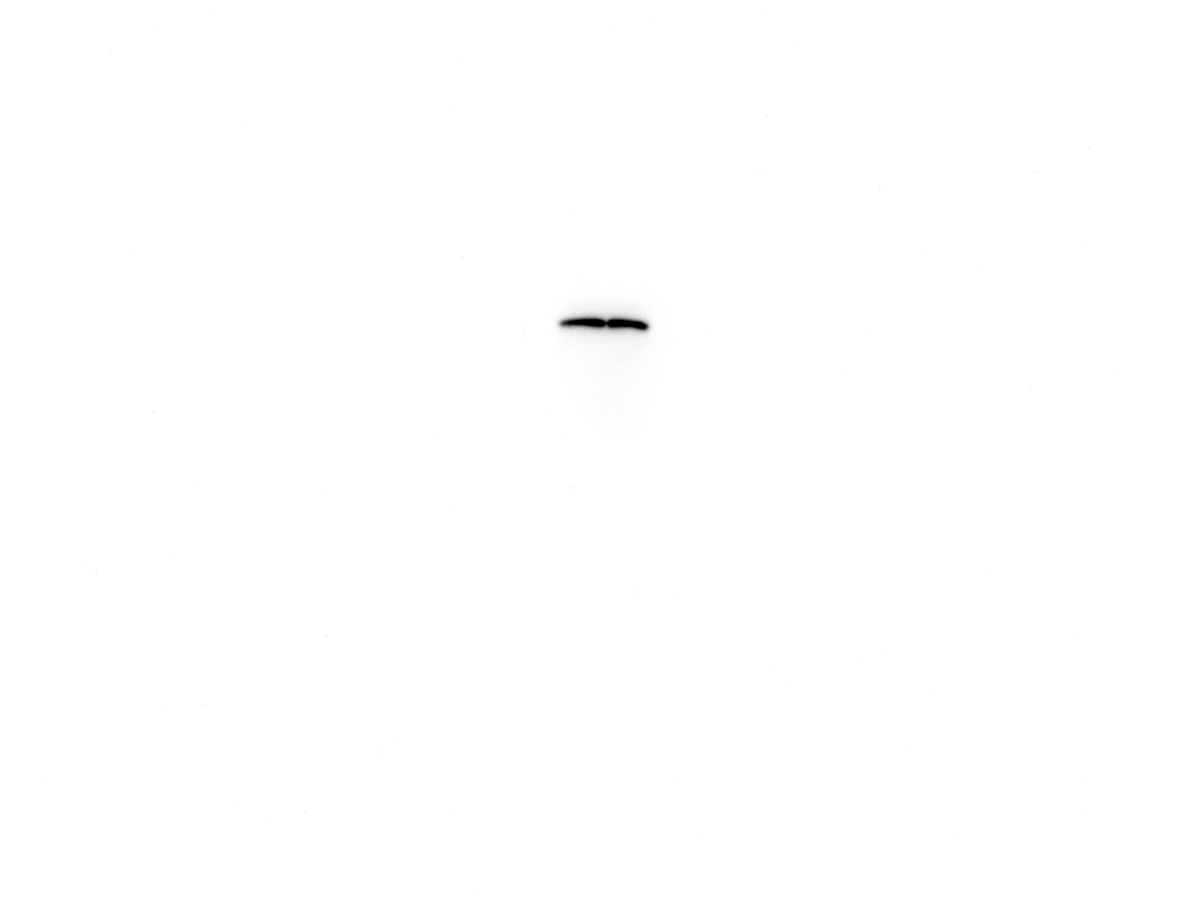

Figure S1C FOXR1

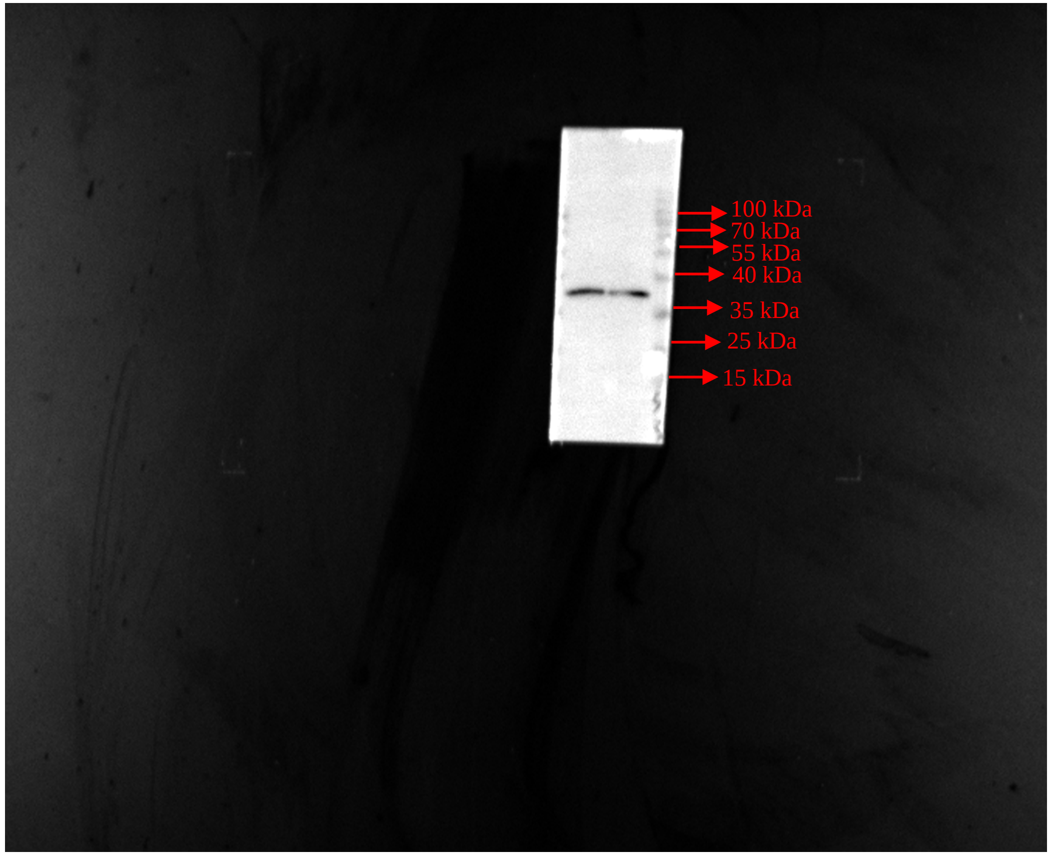

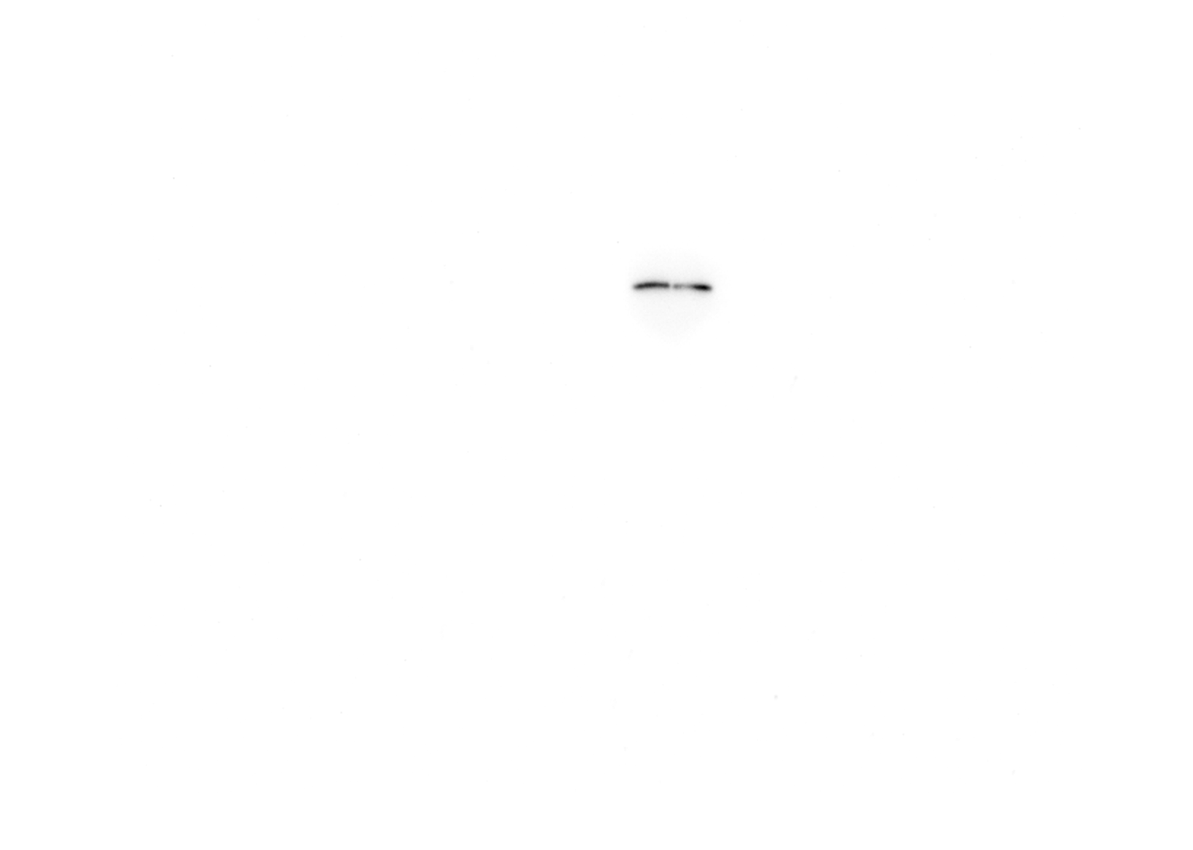

Figure S1C GAPDH

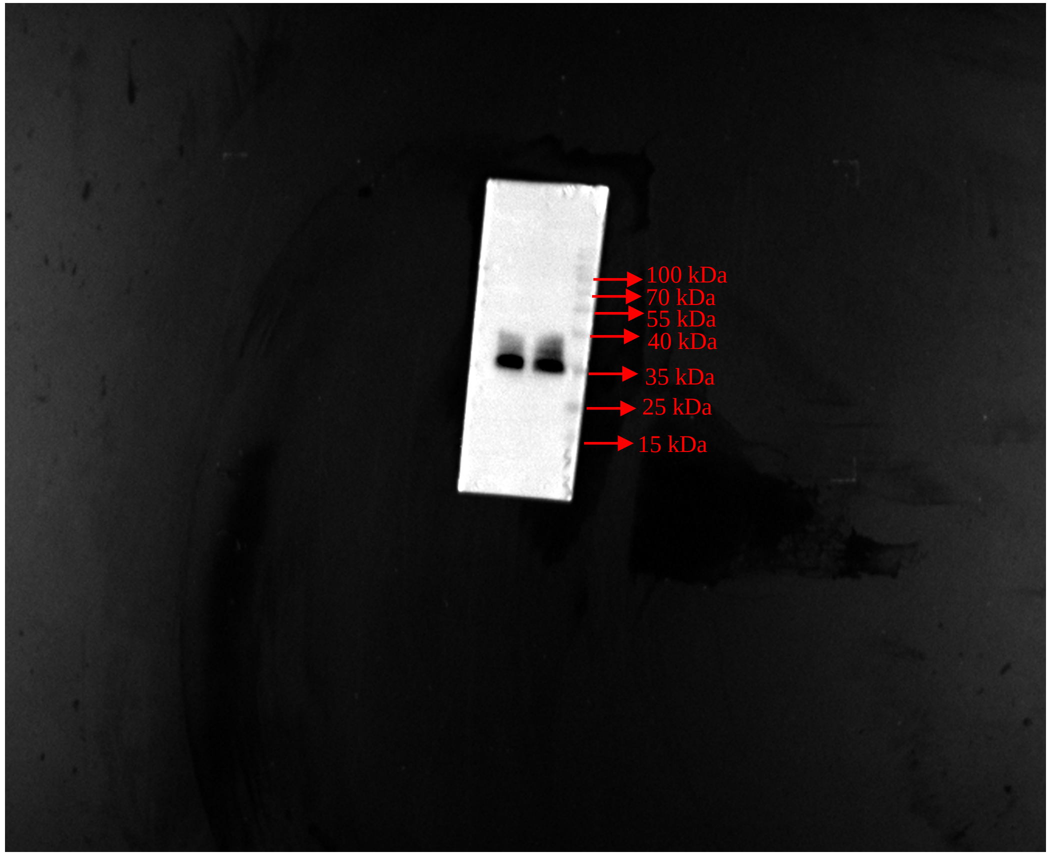

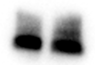

Figure S1C FOXQ1

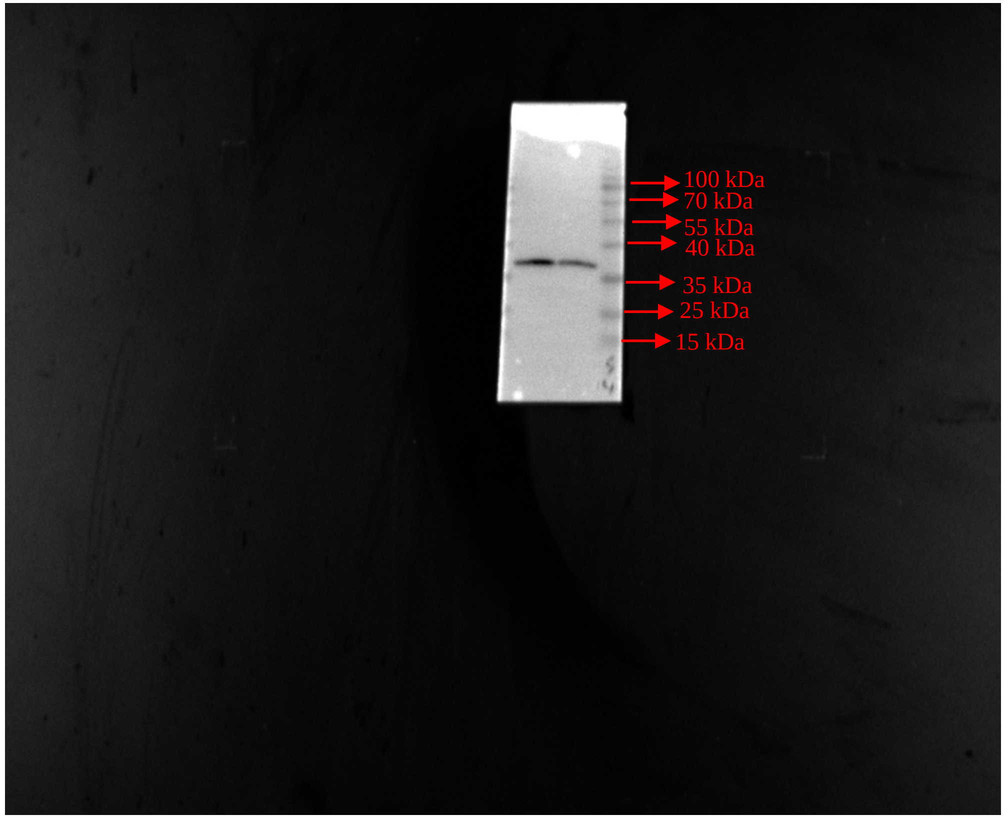

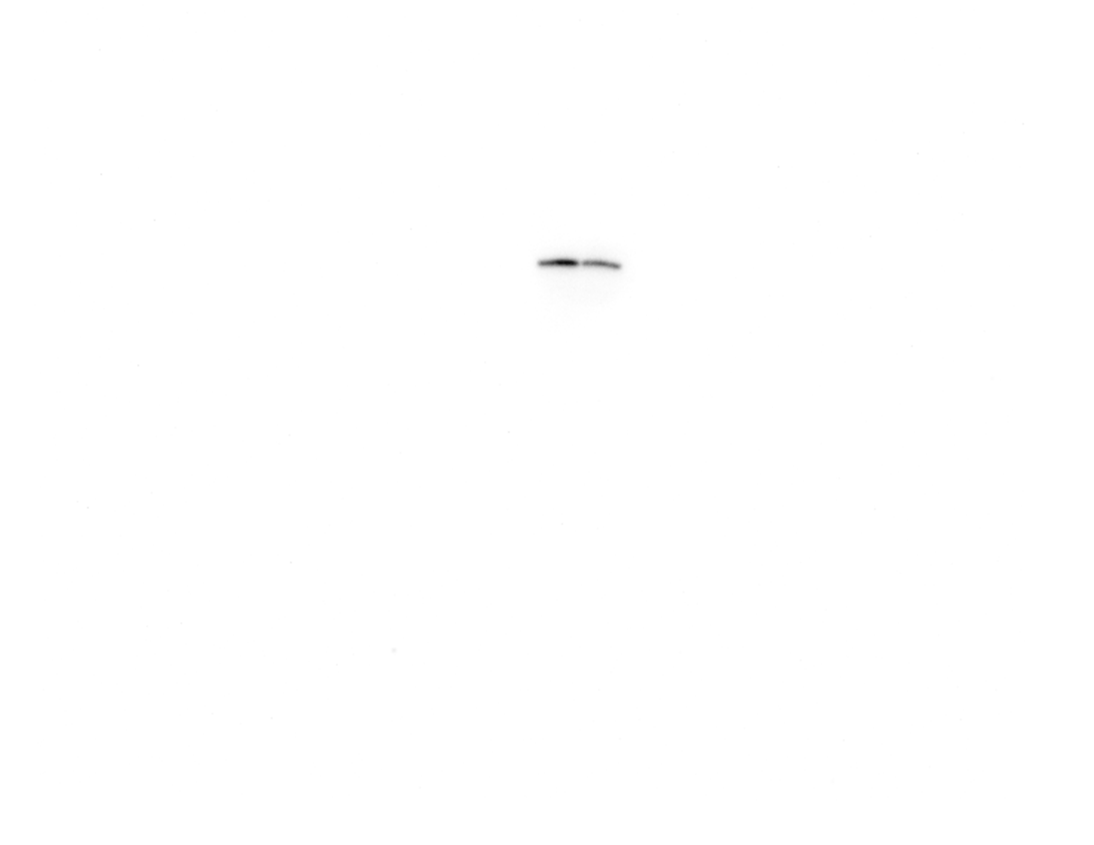

Figure S1C GAPDH

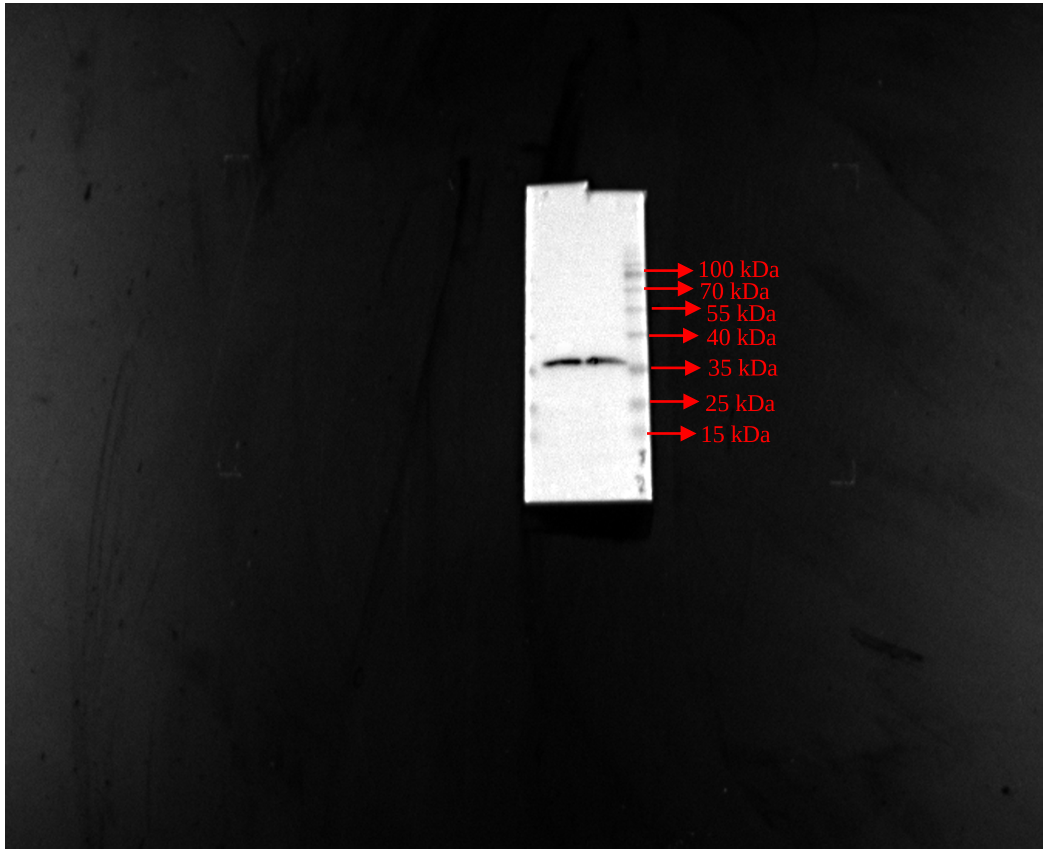

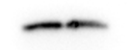

Figure S1C FOXR2

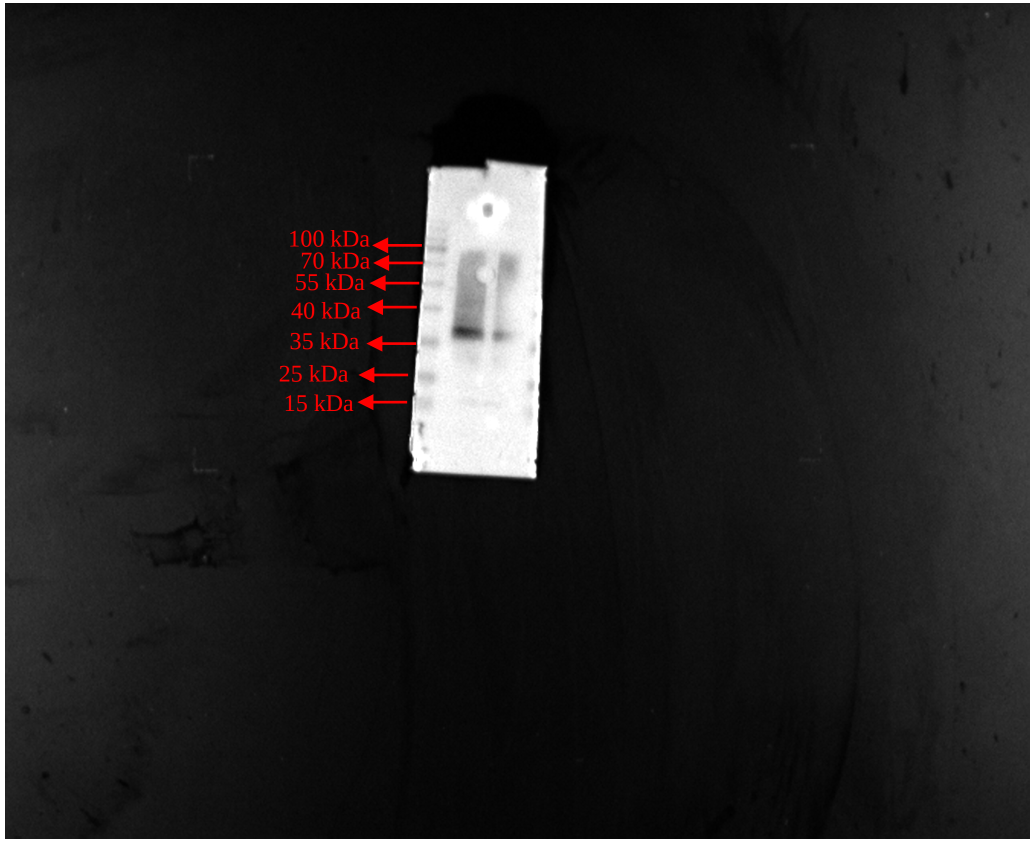

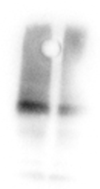

Figure S1C GAPDH

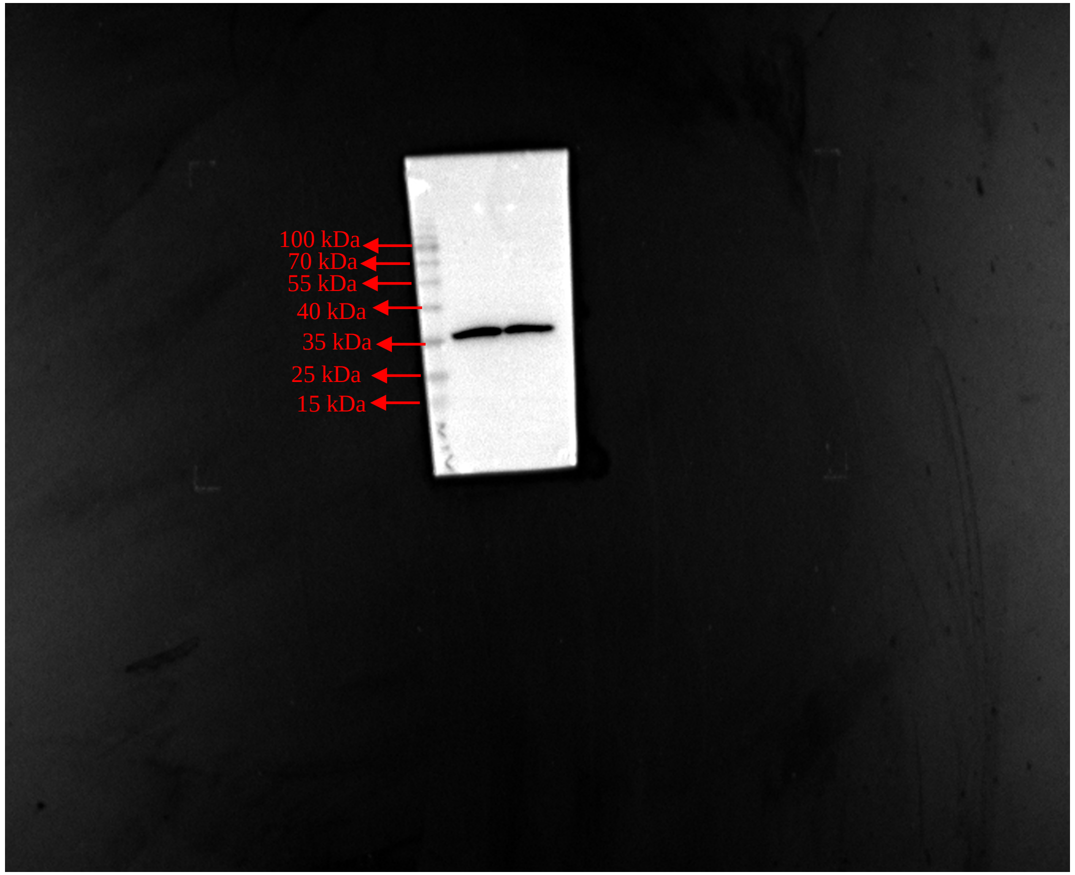

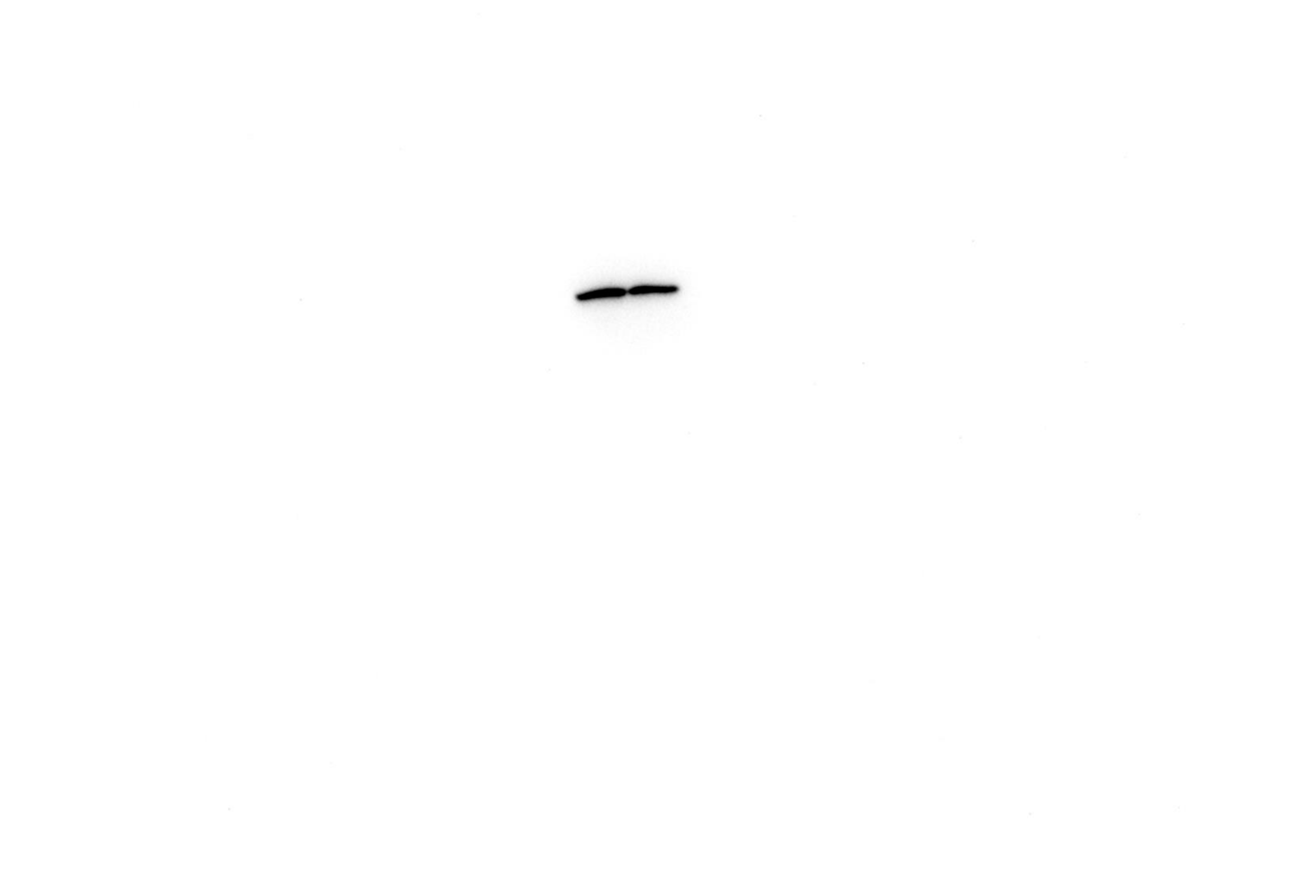

Figure S1C FOXC1

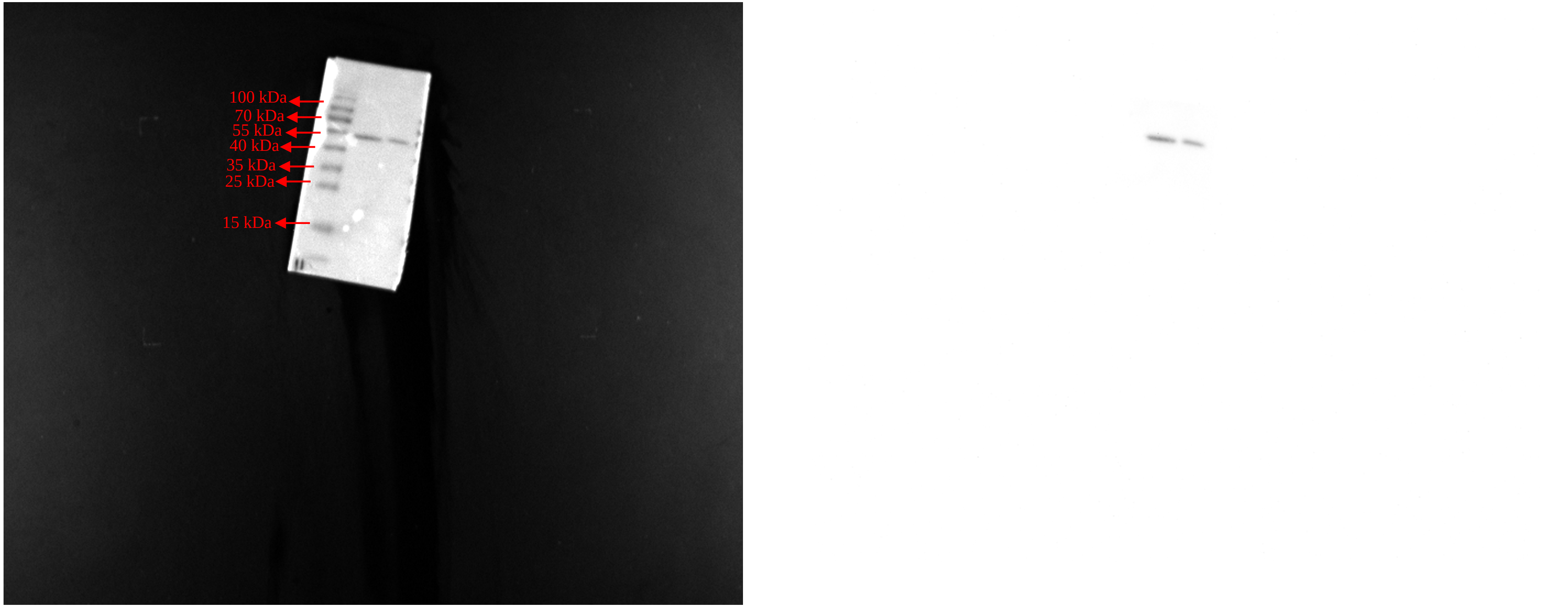

Figure S1C GAPDH

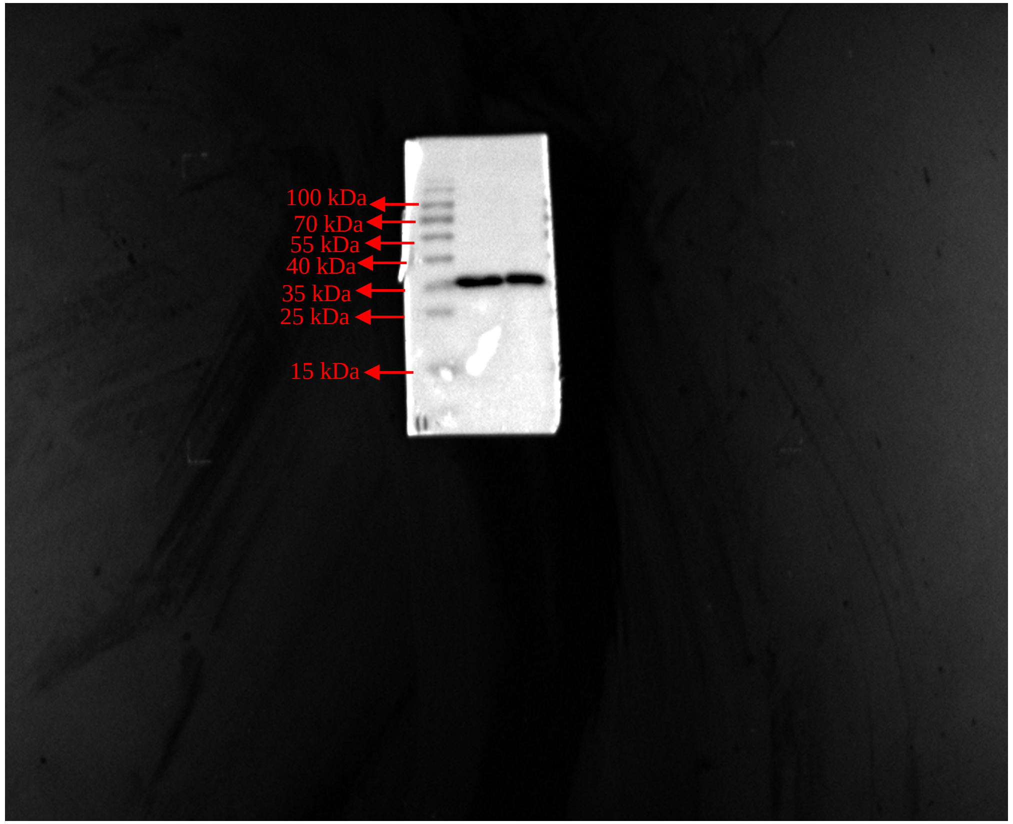

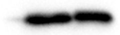

Figure S1C FOXD1

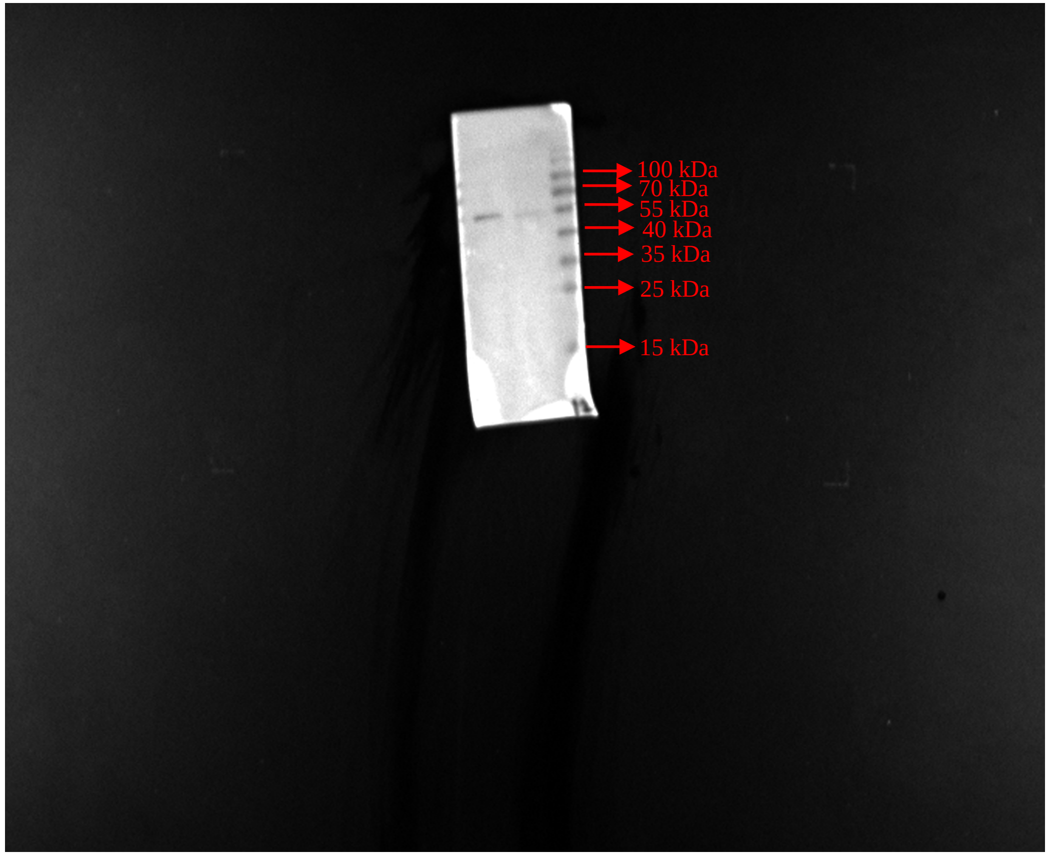

Figure S1C GAPDH

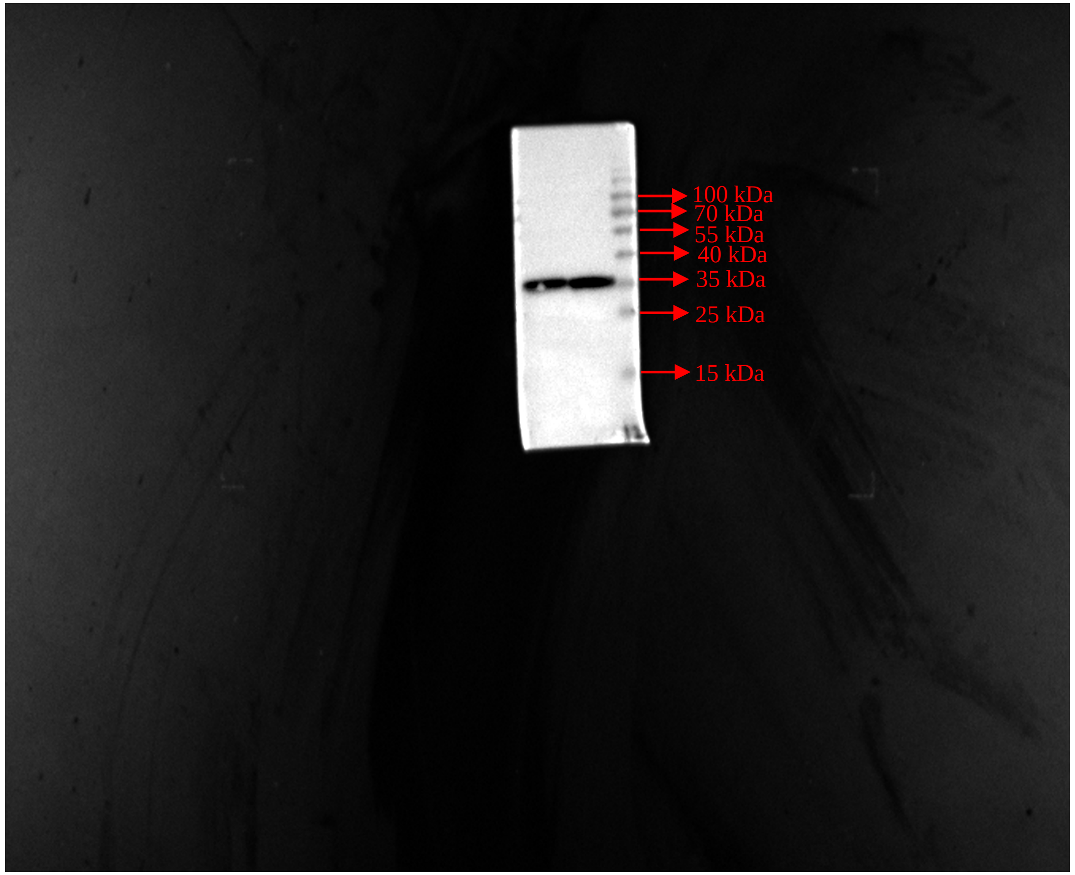

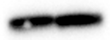

Figure S1C FOXD3

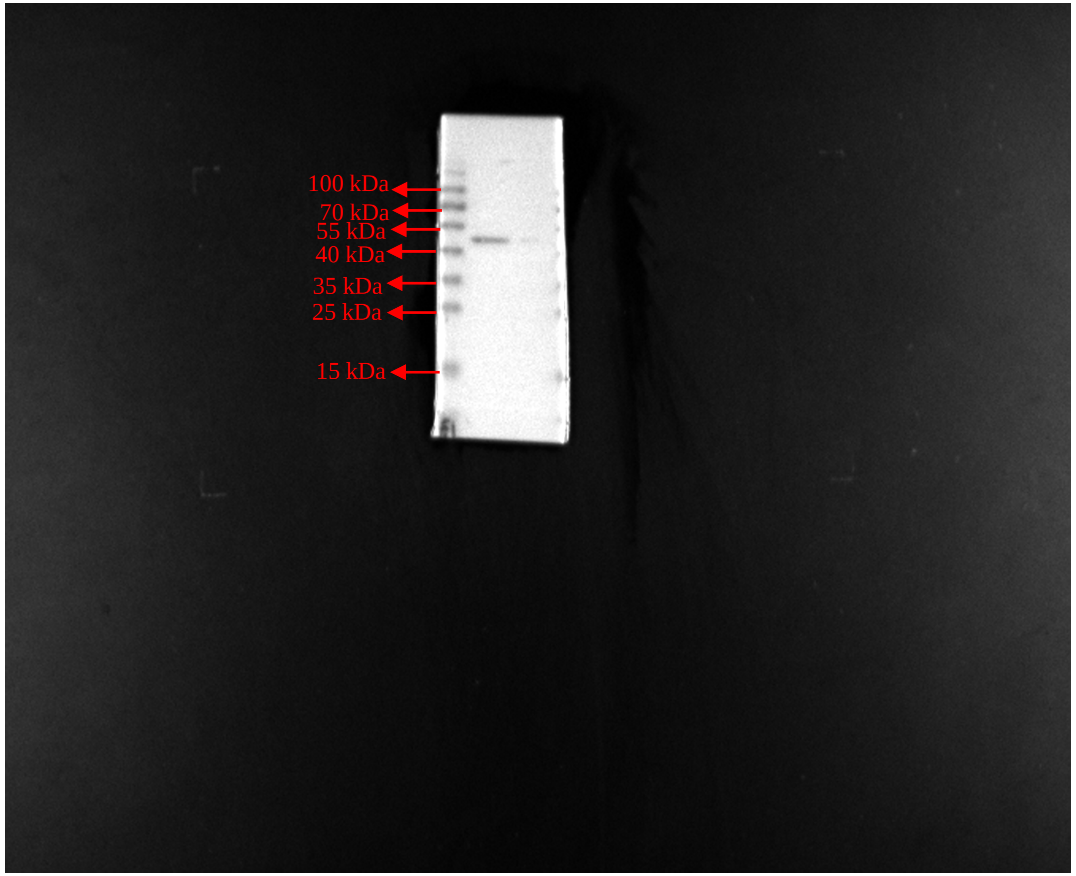

Figure S1C GAPDH

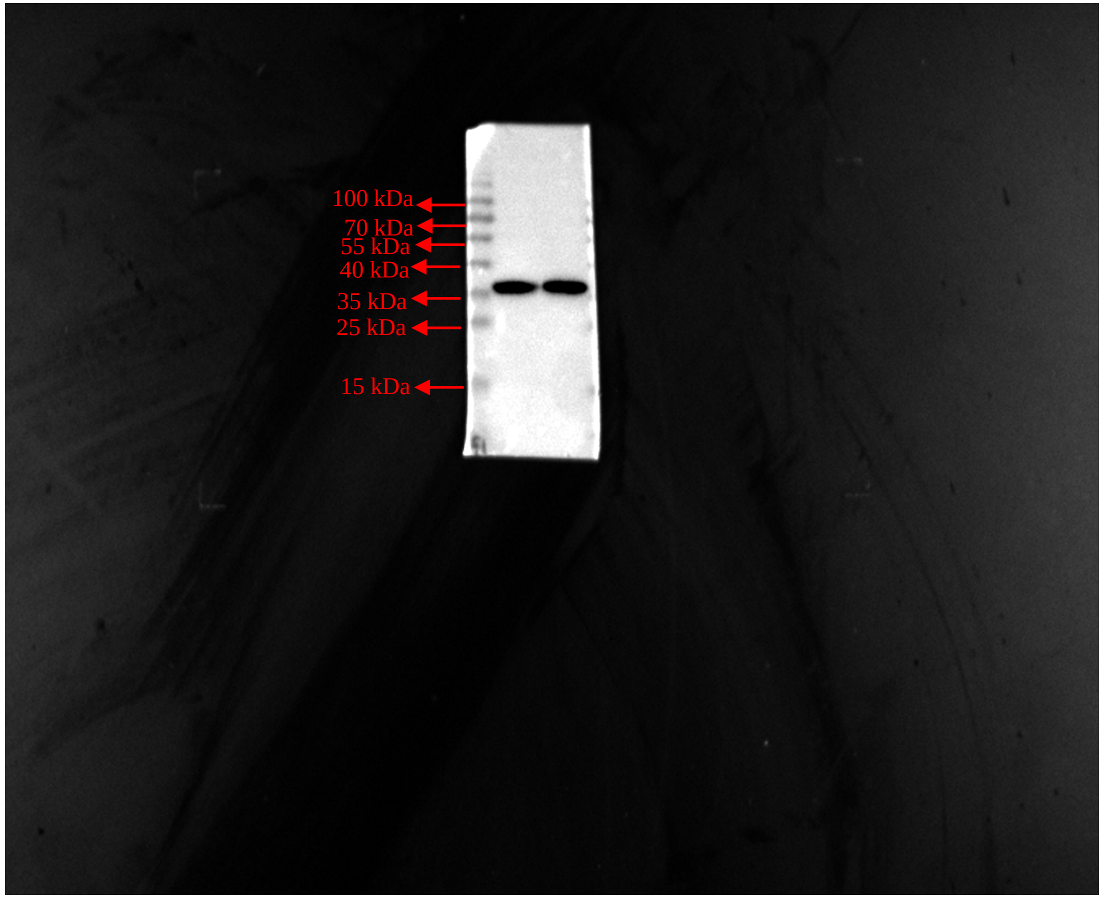

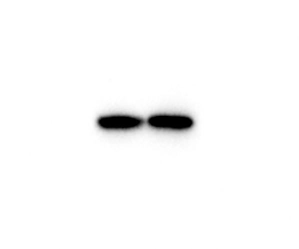

Figure S1C FOXD4

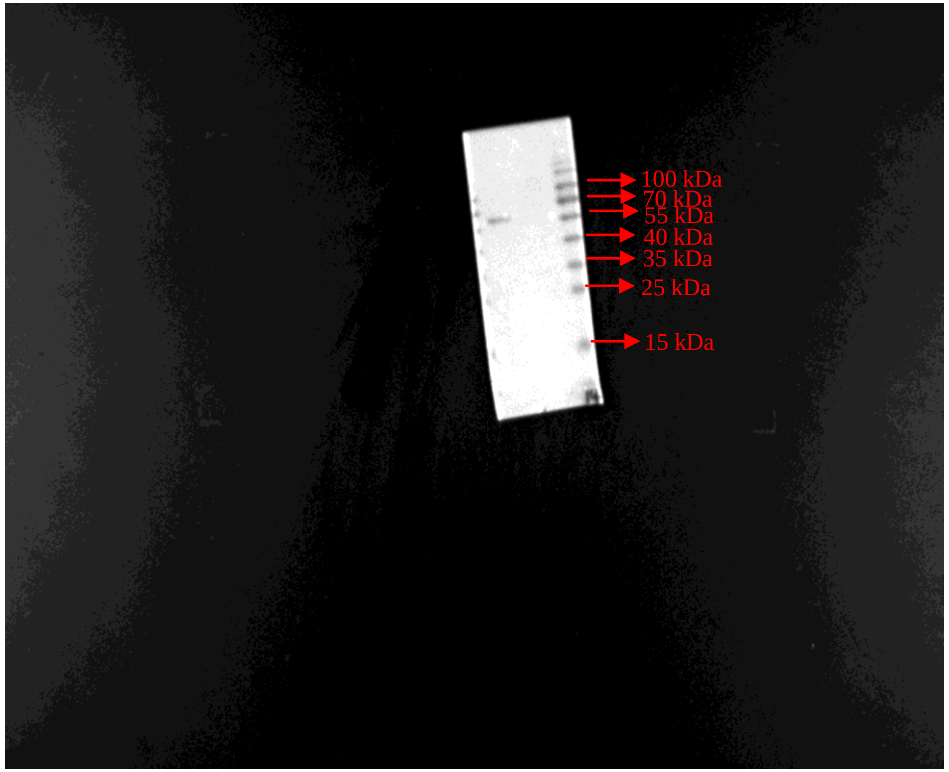

Figure S1C GAPDH

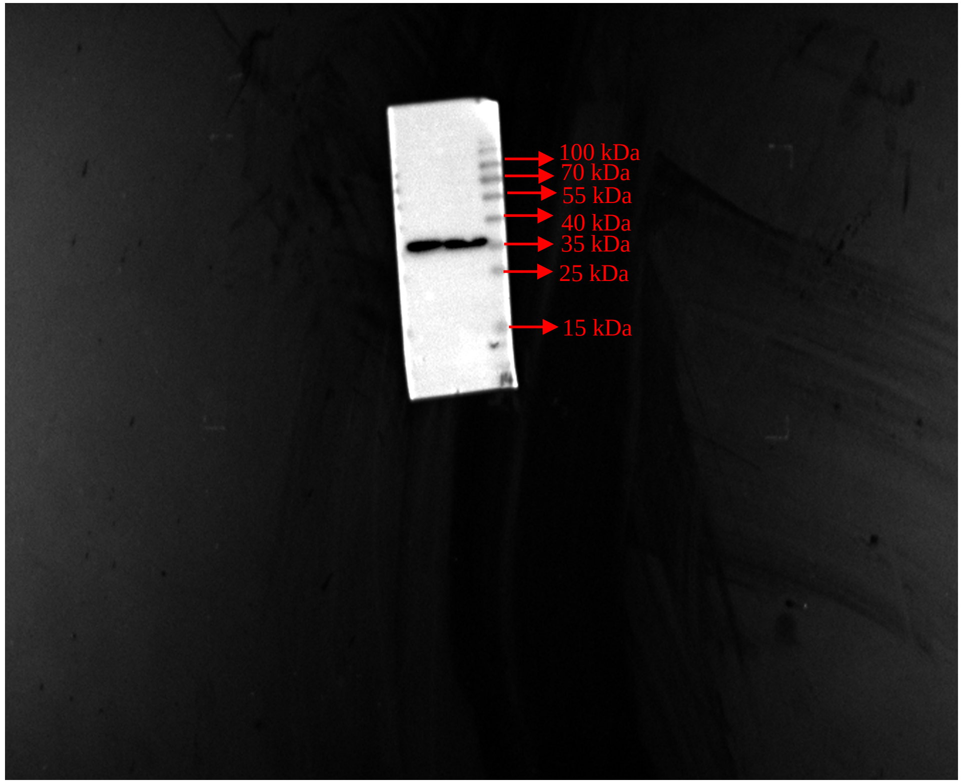

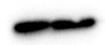

Figure S1C FOXF2

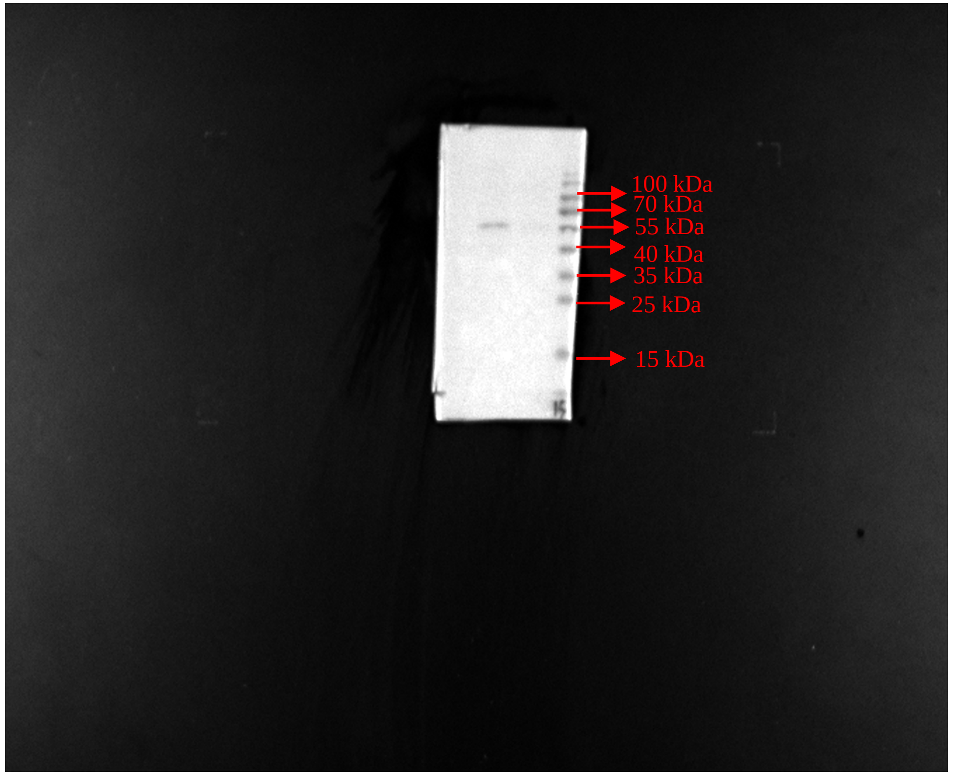

Figure S1C GAPDH

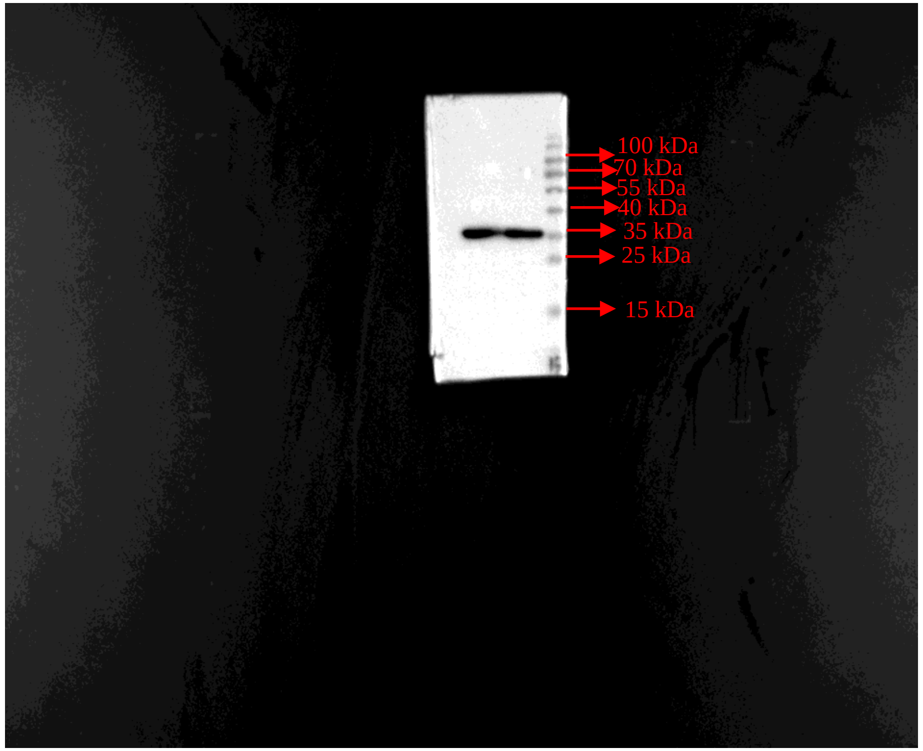

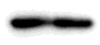

Figure S1C FOXJ1

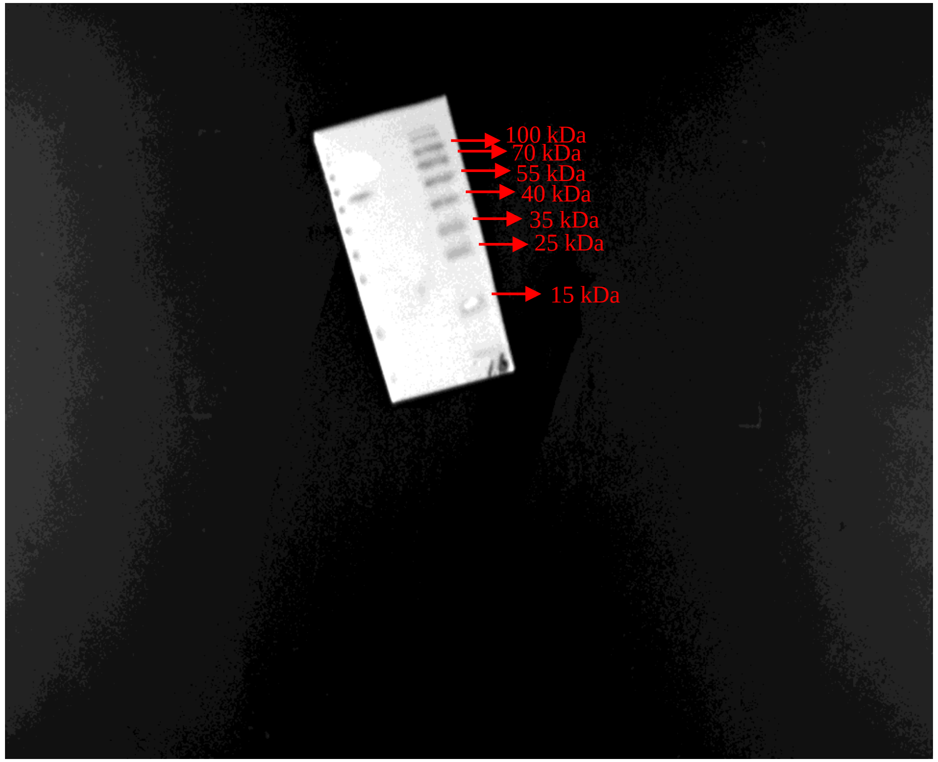

Figure S1C GAPDH

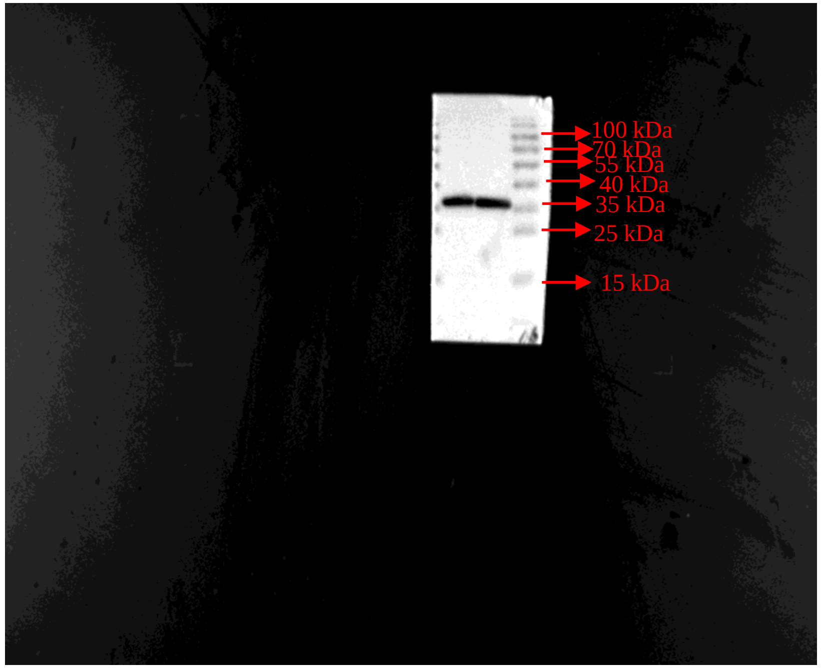

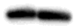

Figure S1C FOXN2

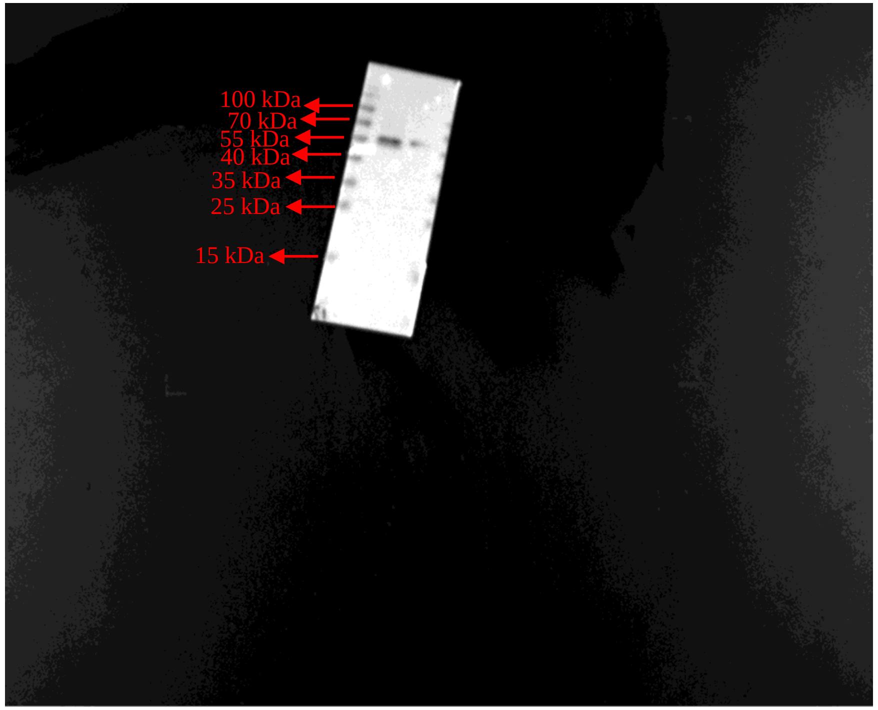

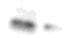

Figure S1C GAPDH

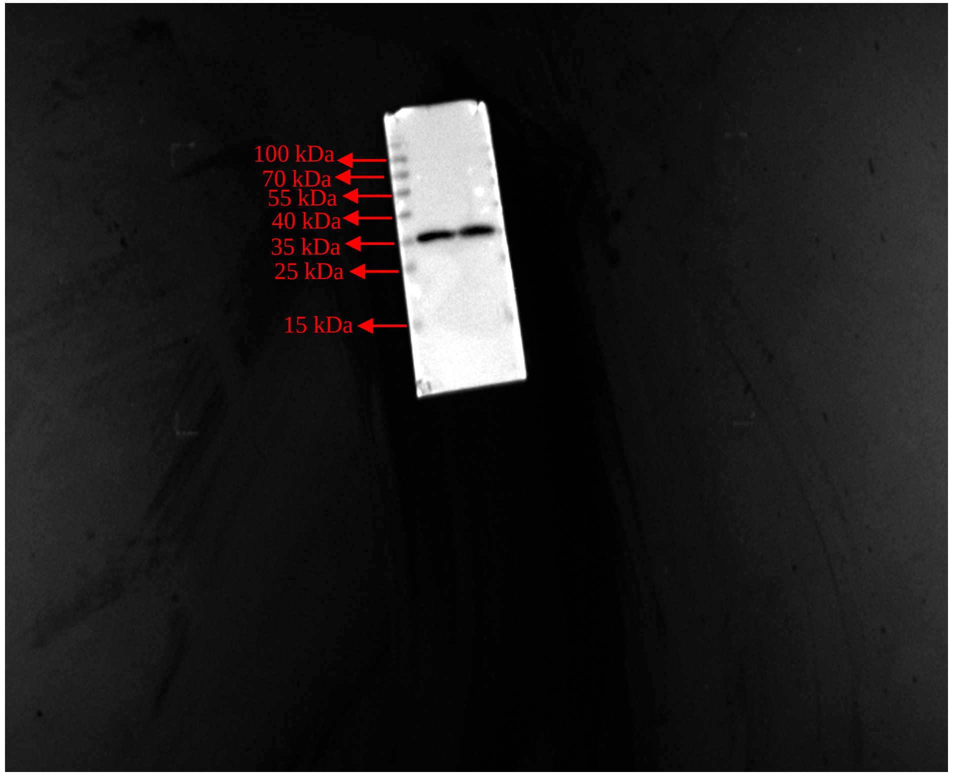

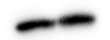

Figure S1C FOXN3

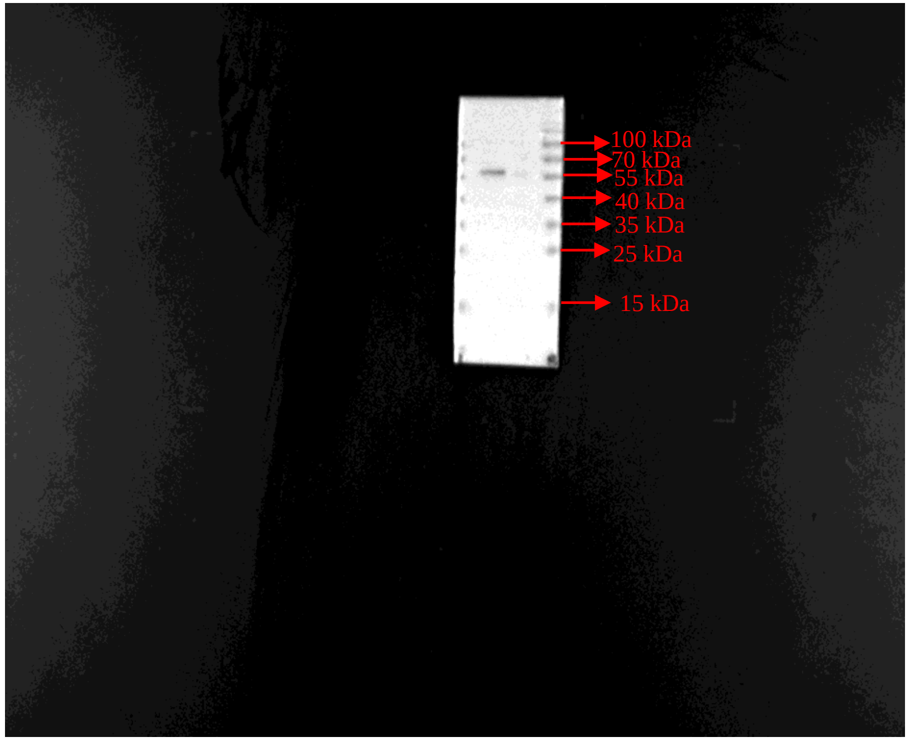

Figure S1C GAPDH

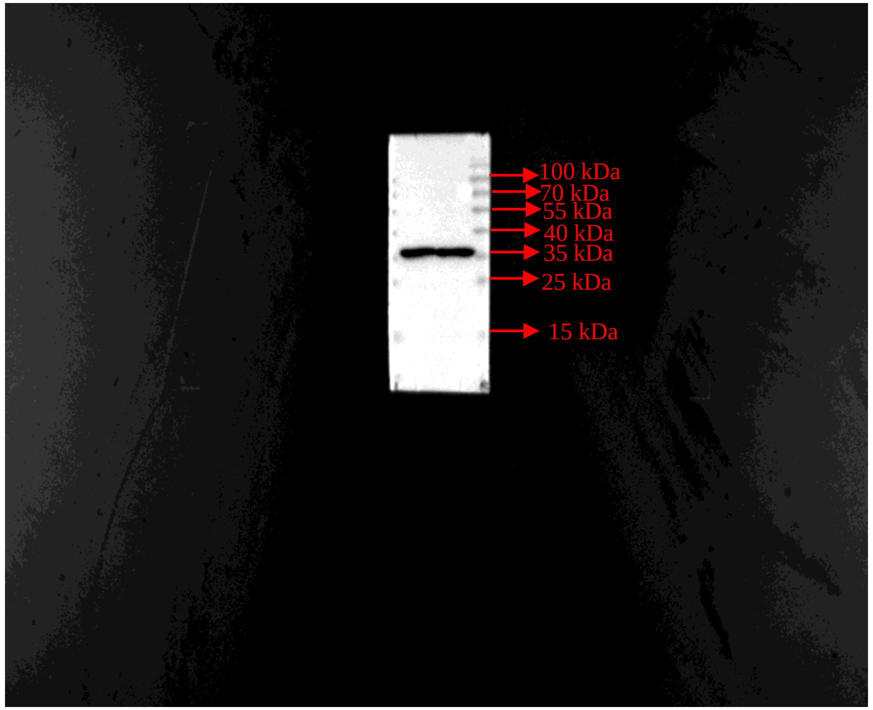

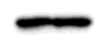

Figure S1C FOXN4

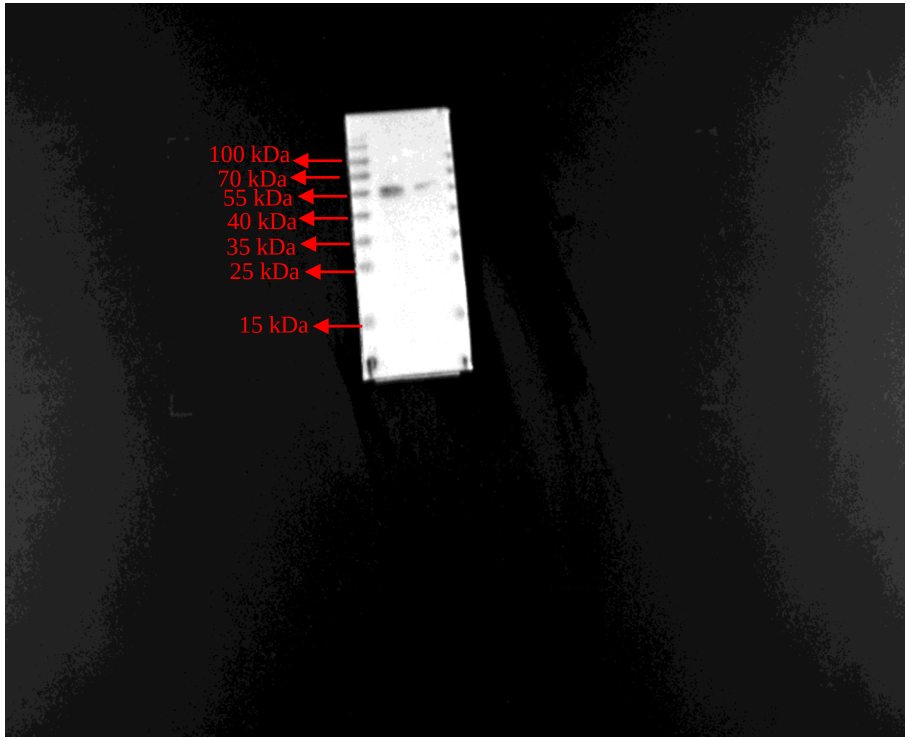

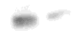

Figure S1C GAPDH

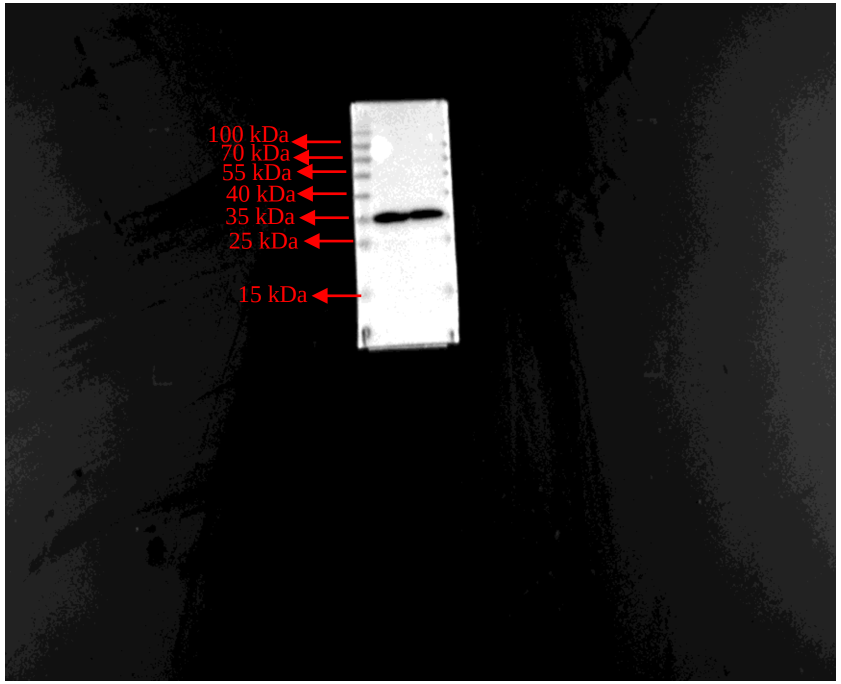

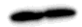

Figure S1C FOXL2

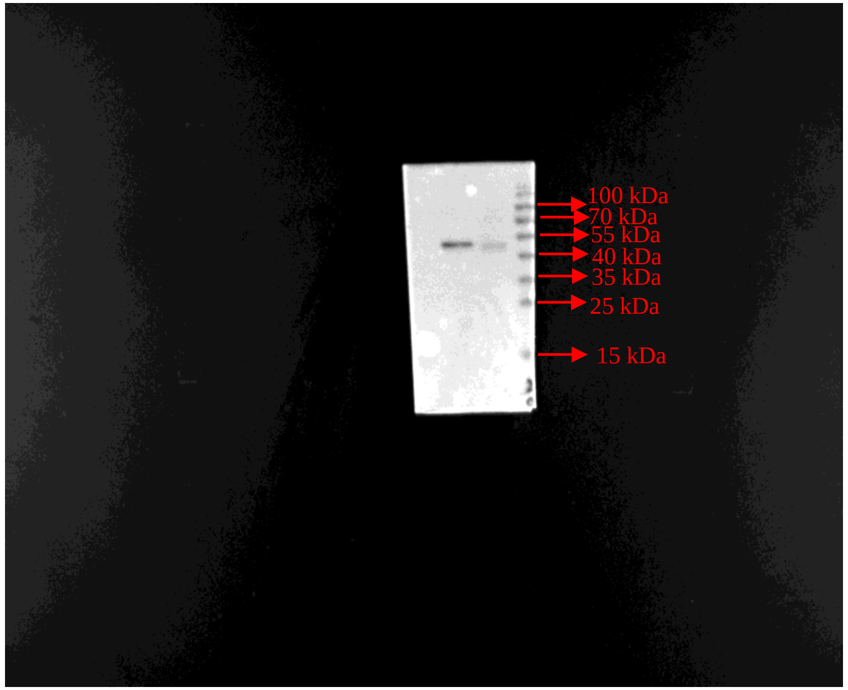

Figure S1C GAPDH

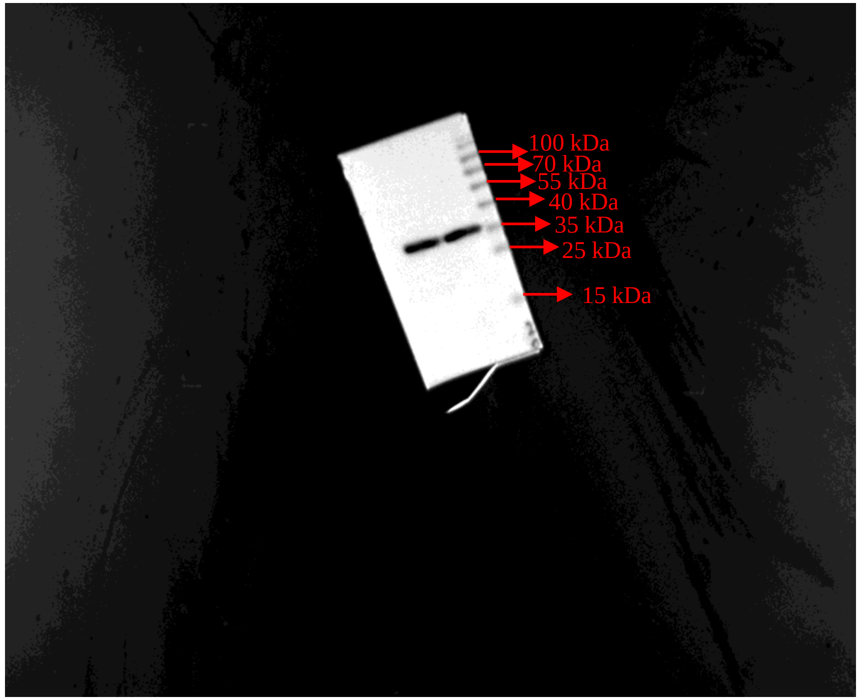

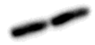

Figure S1C FOXC1

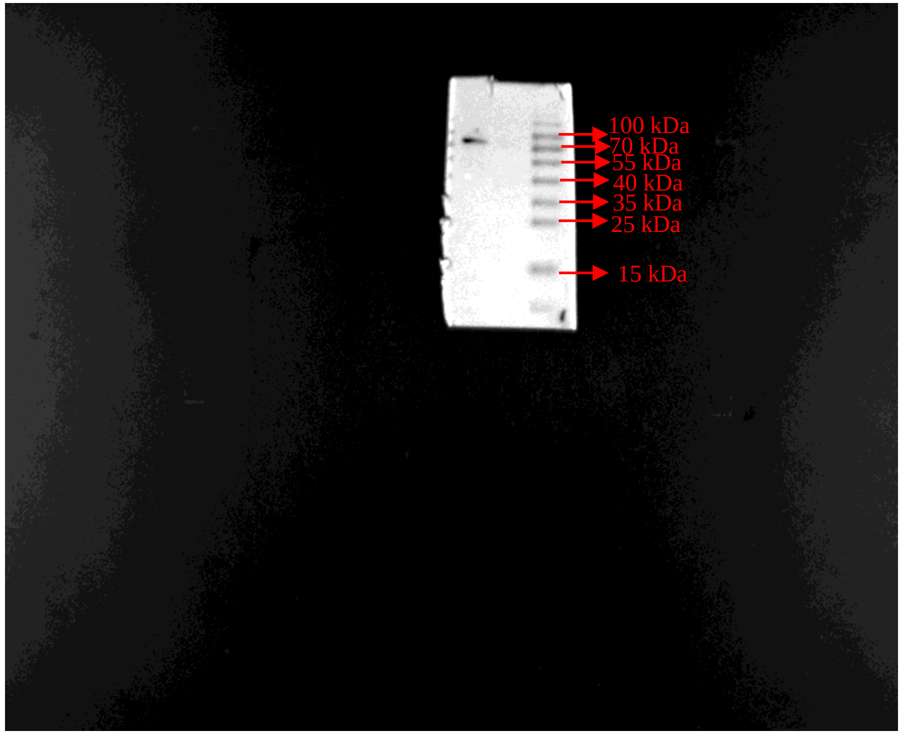

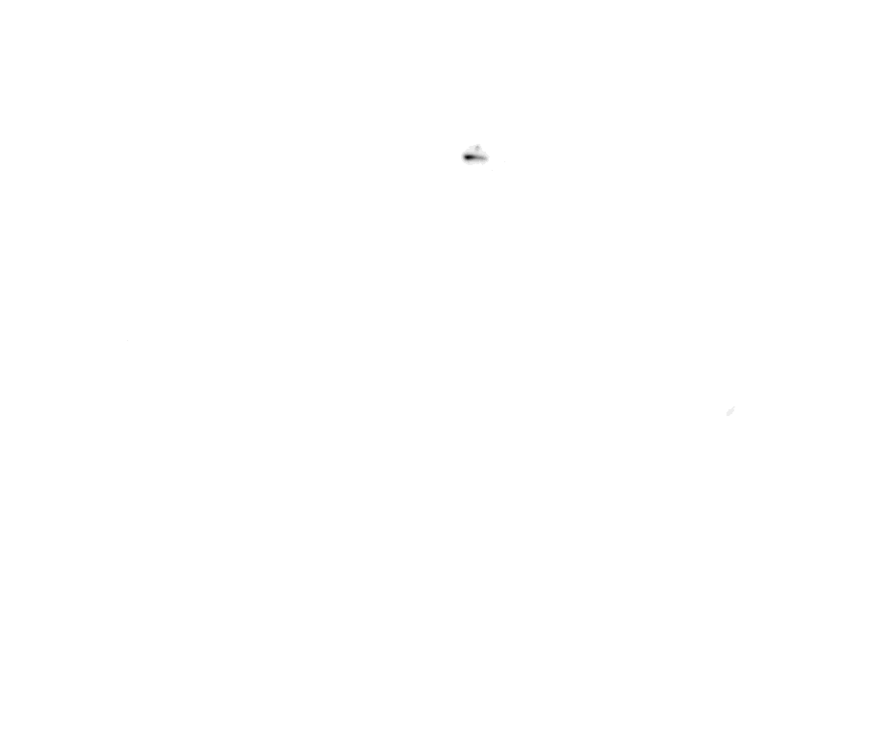

Figure S1C GAPDH

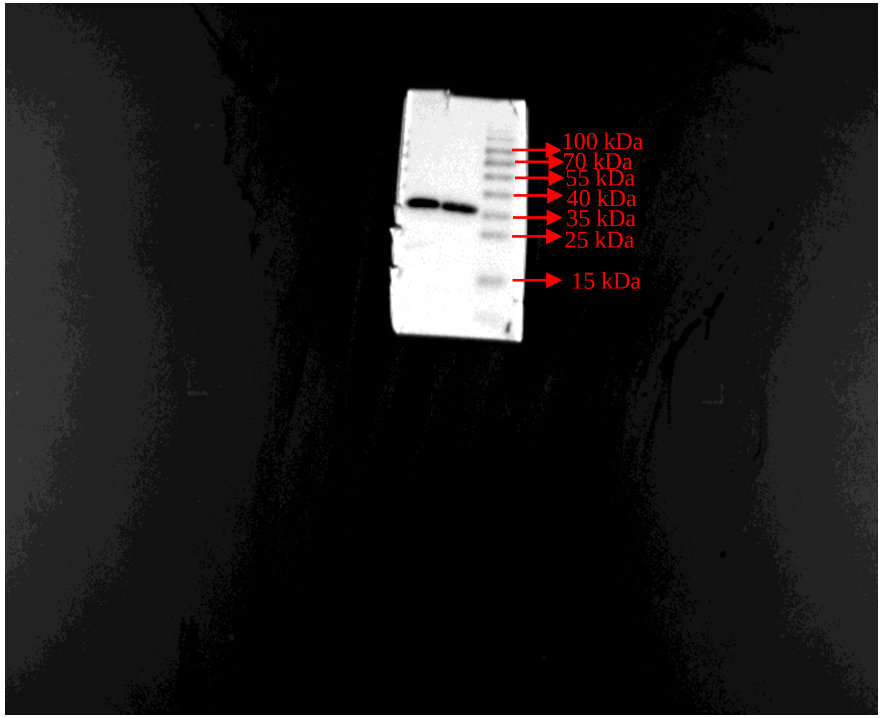

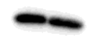

Figure S1C FOXJ2

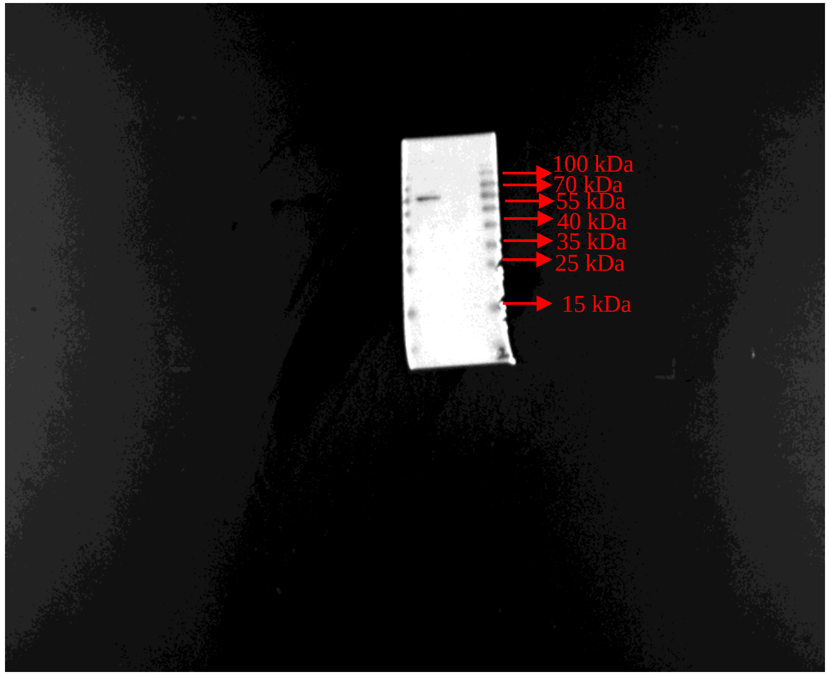

Figure S1C GAPDH

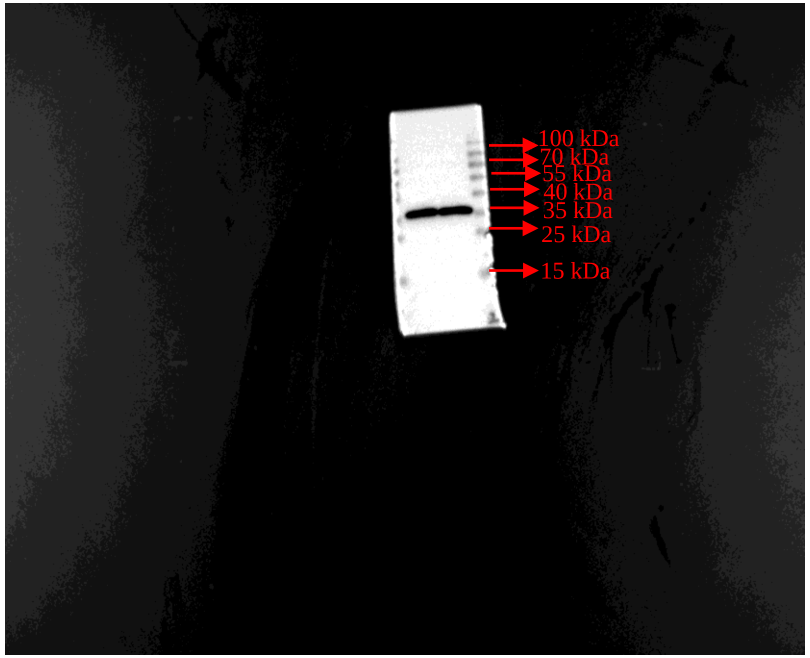

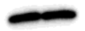

Figure S1C FOXK1

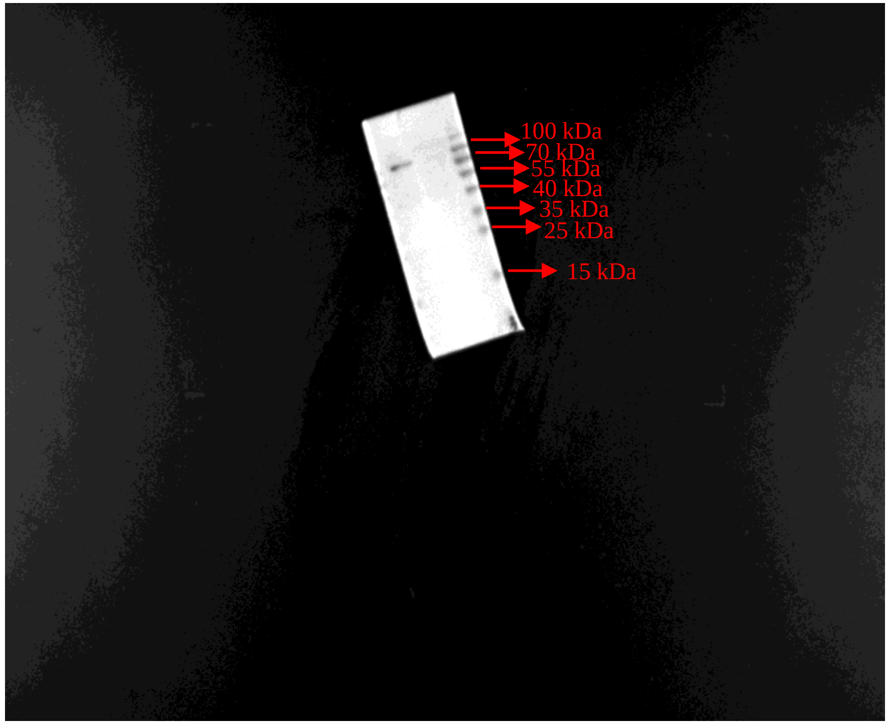

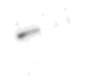

Figure S1C GAPDH

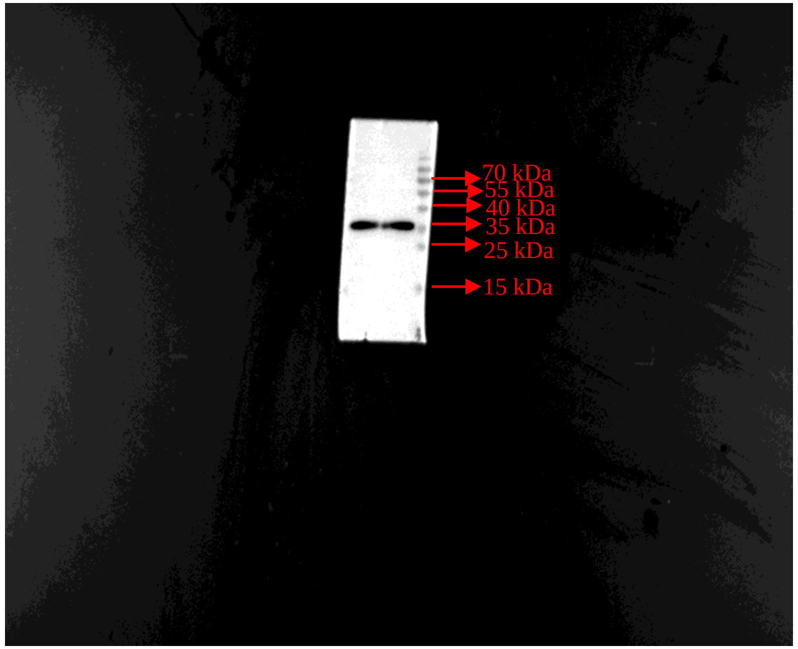

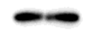

Figure S1C FOXK2

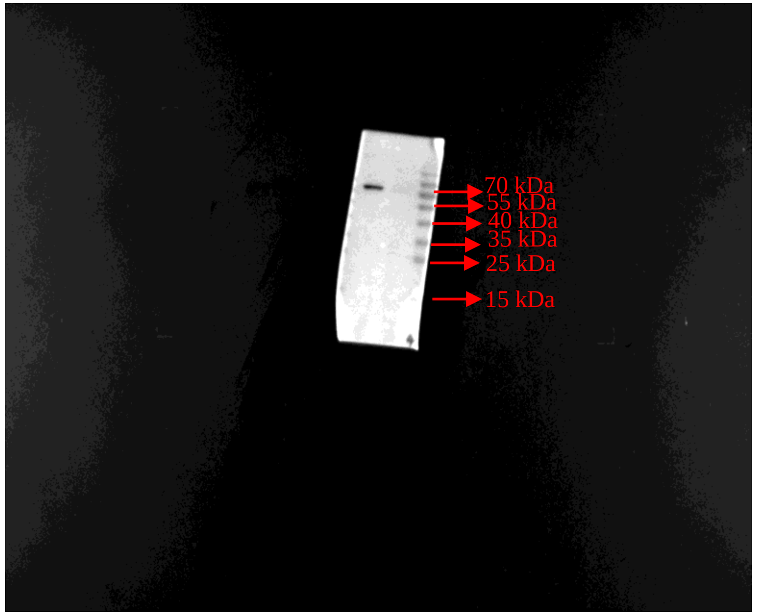

Figure S1C GAPDH

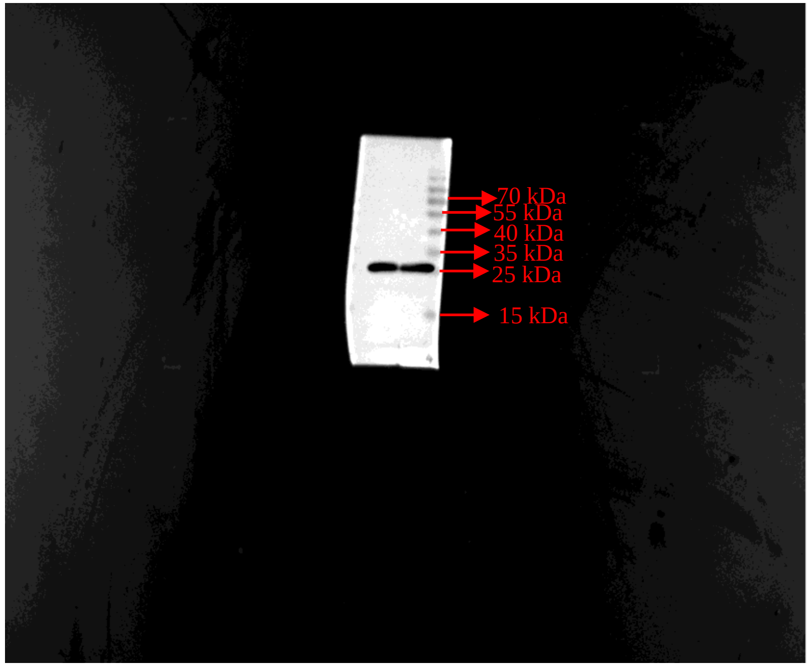

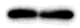

Figure S1C FOXM1

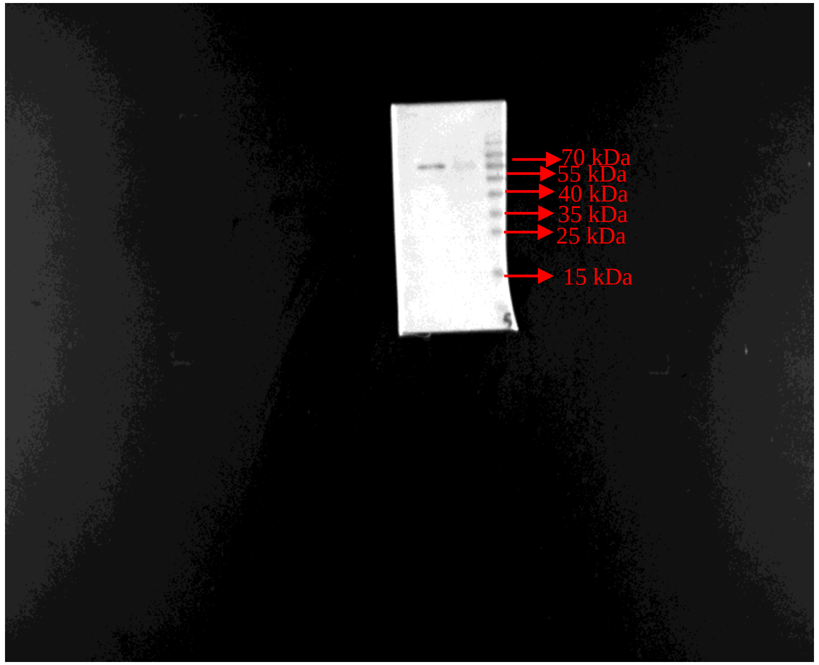

Figure S1C GAPDH

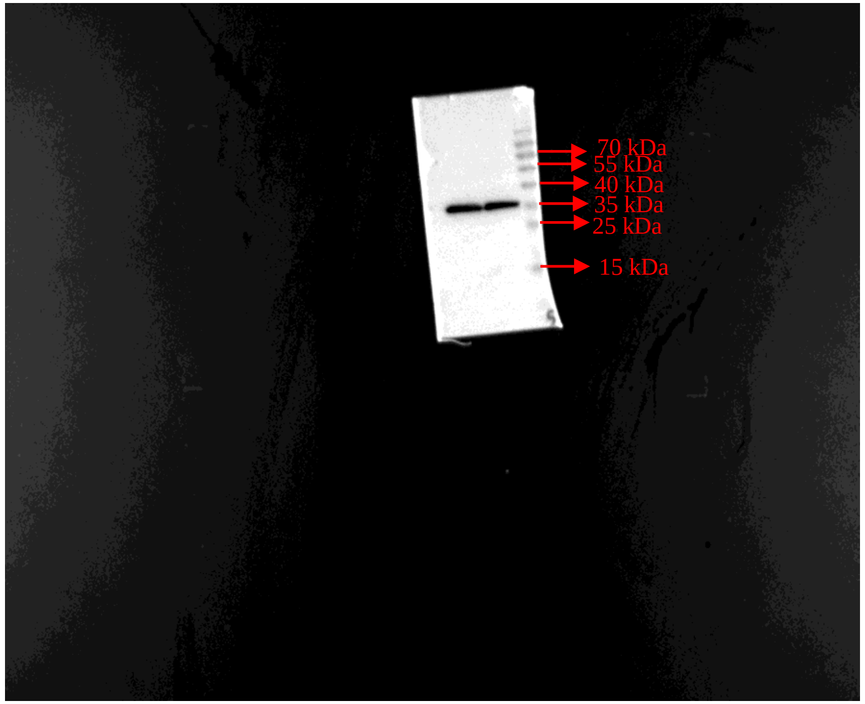

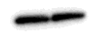

Figure S1C FOXO1

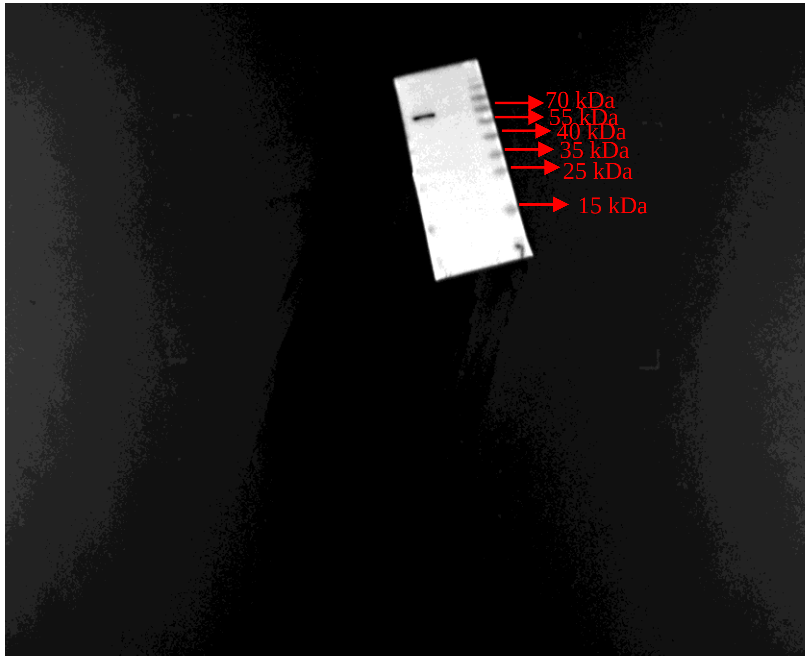

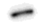

Figure S1C GAPDH

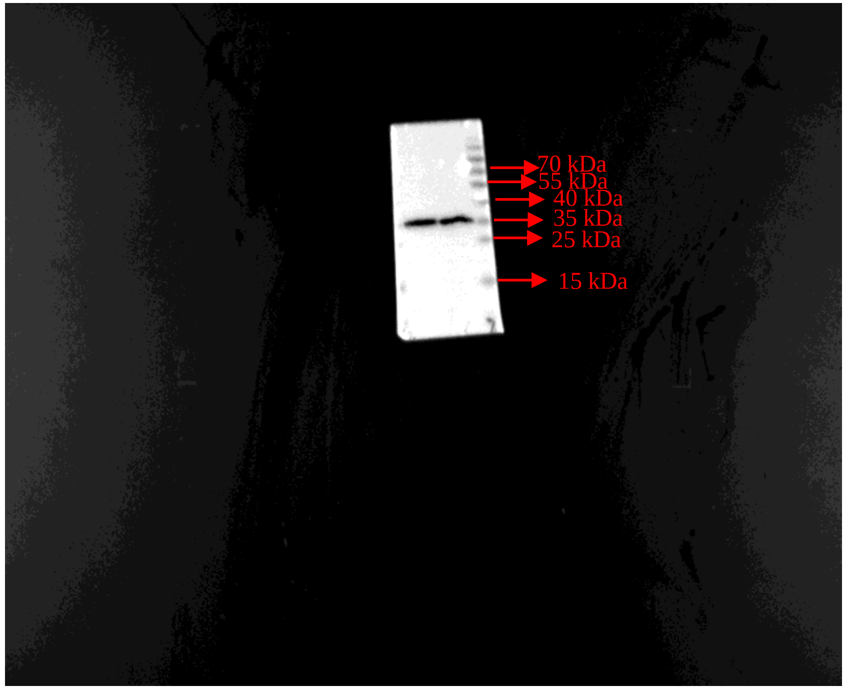

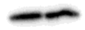

Figure S1C FOXO3

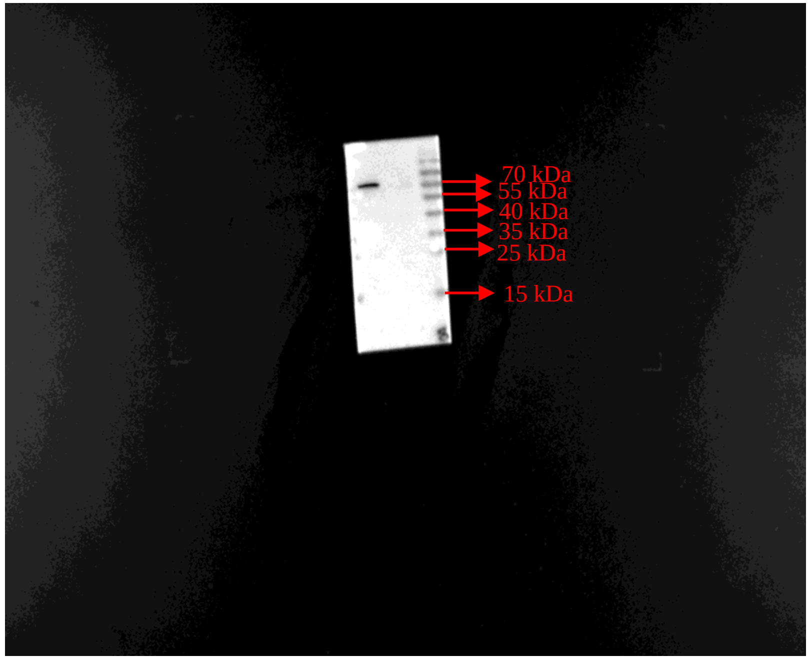

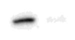

Figure S1C GAPDH

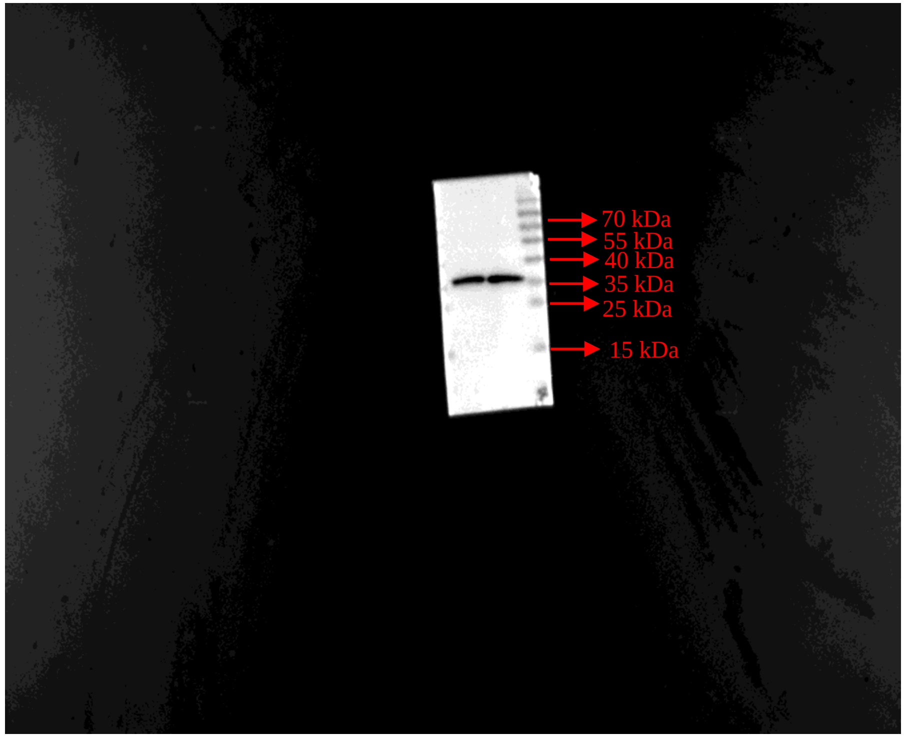

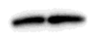

Figure S1C FOXO4

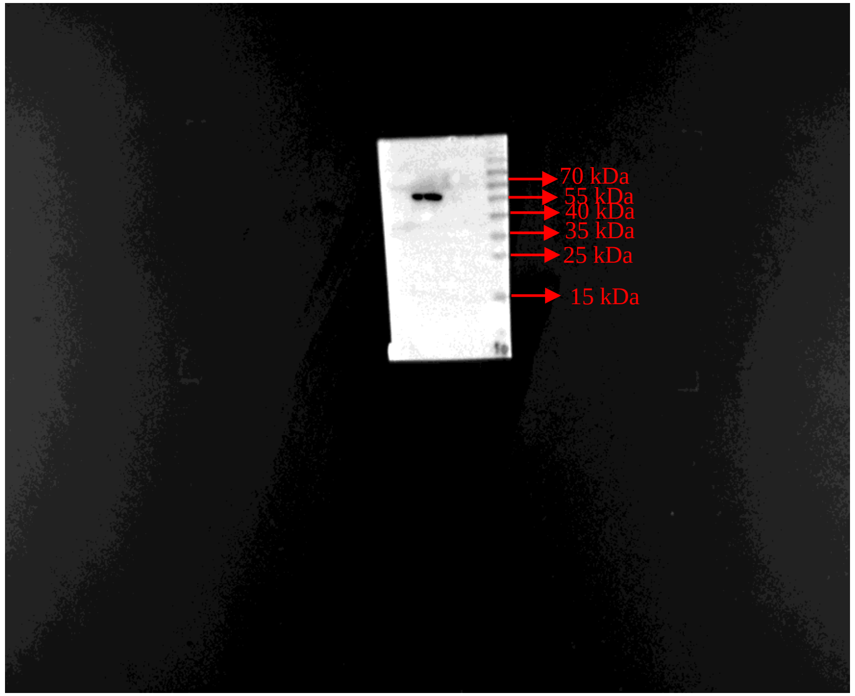

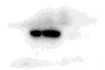

Figure S1C GAPDH

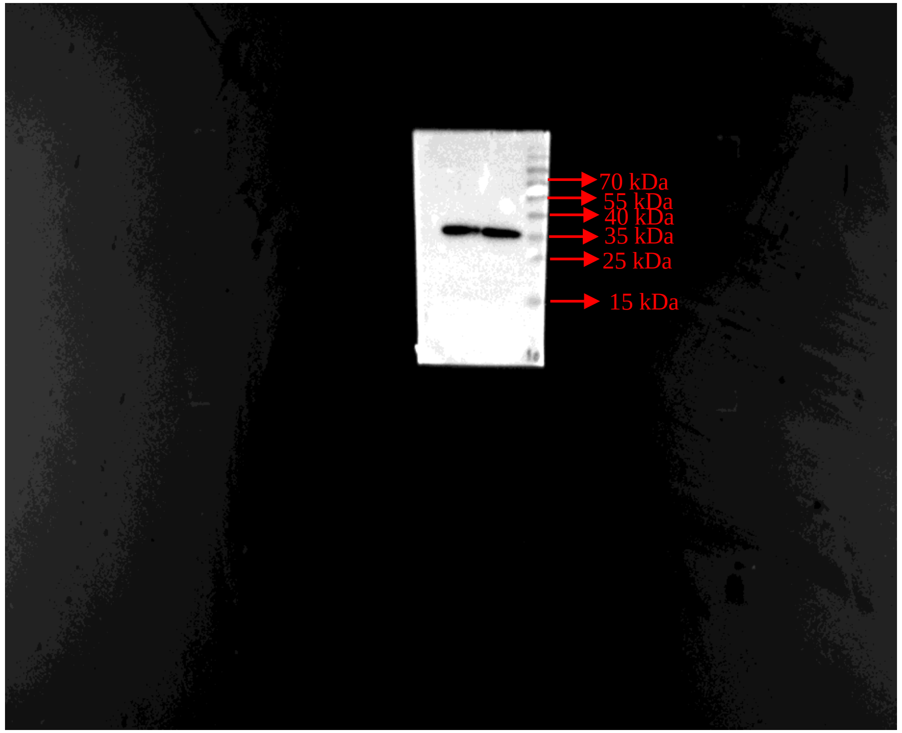

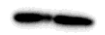

Figure S1C FOXP1

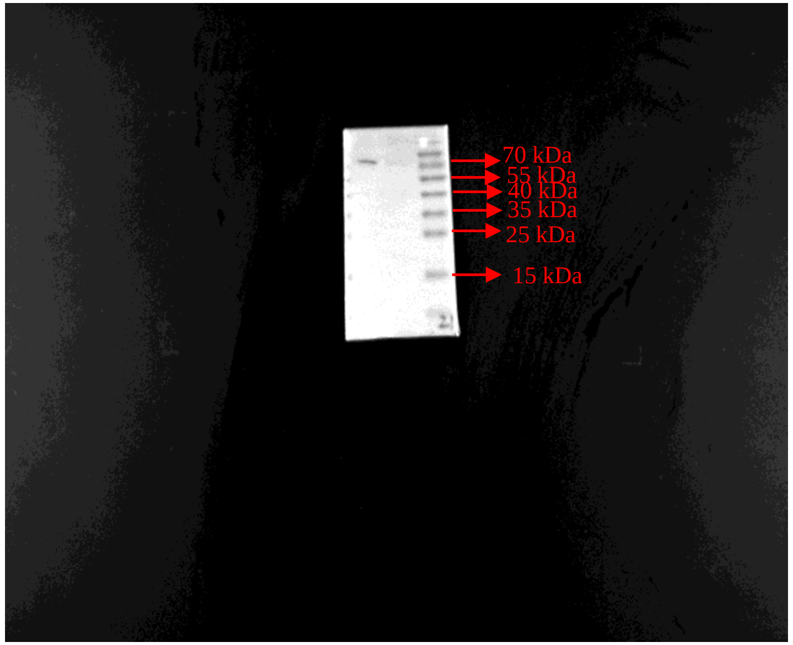

Figure S1C GAPDH

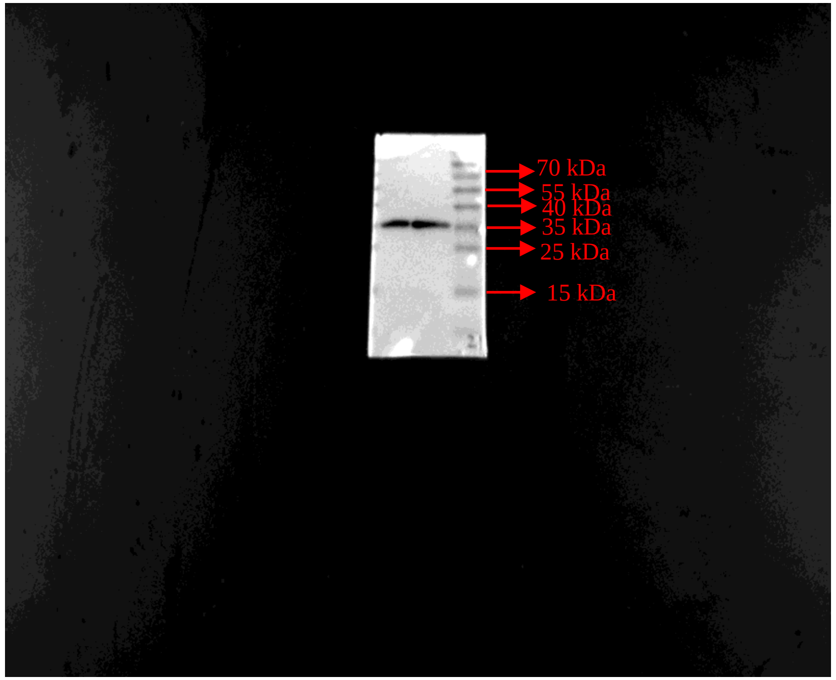

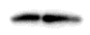

Figure 1H FOXP2

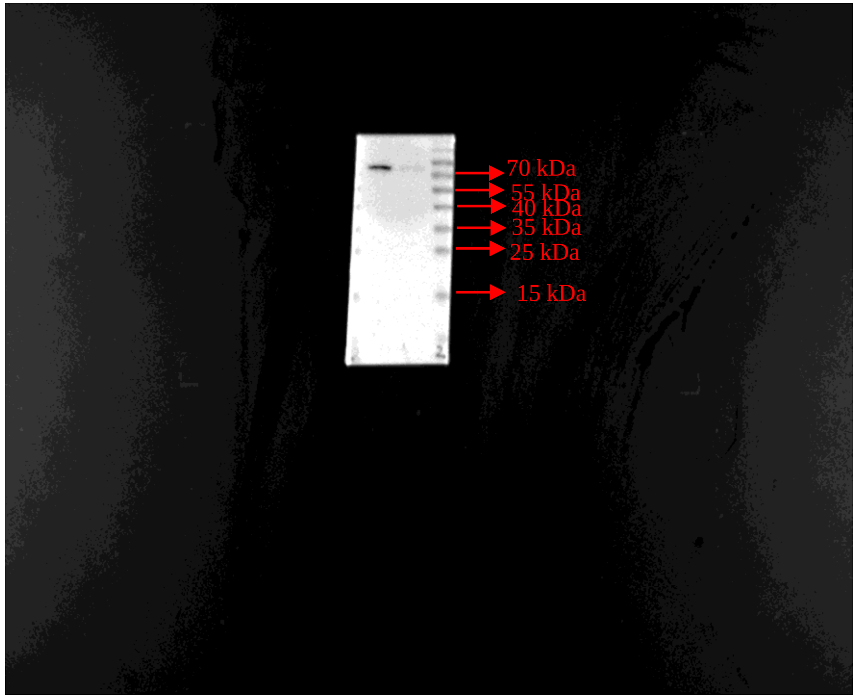

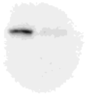

Figure S1C GAPDH

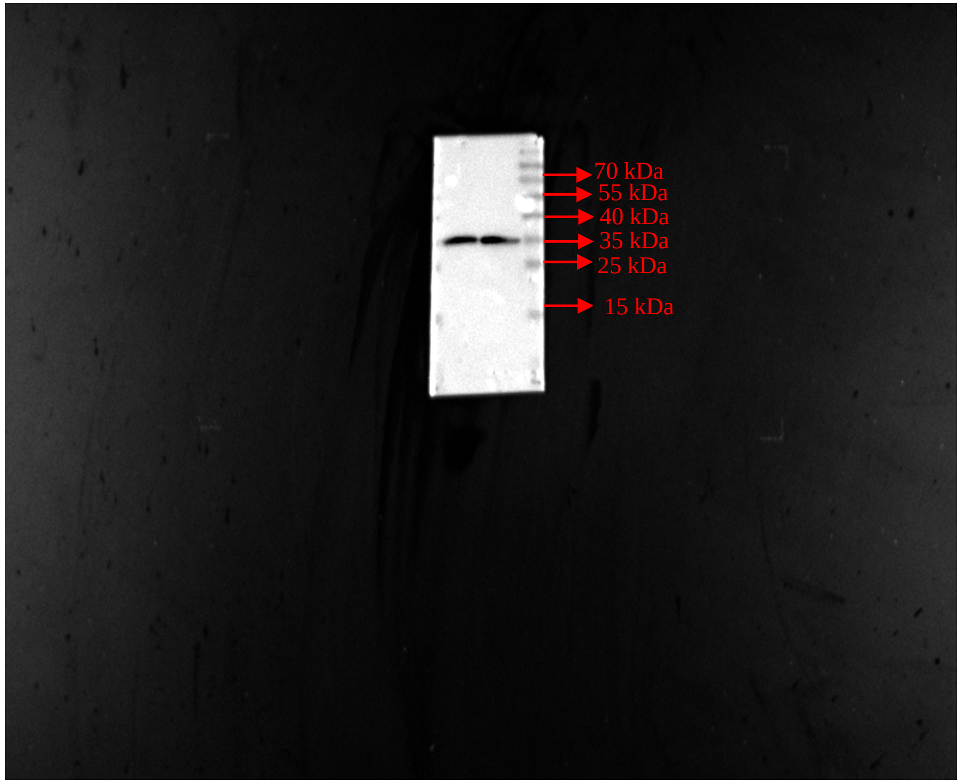

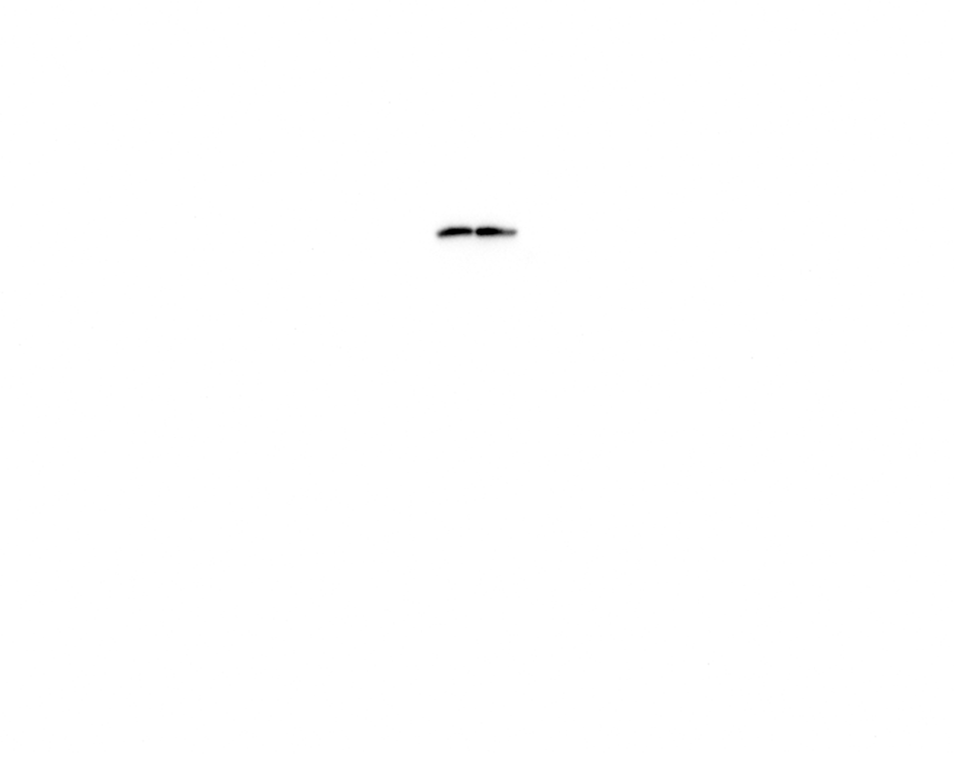

Figure S1C FOXP4

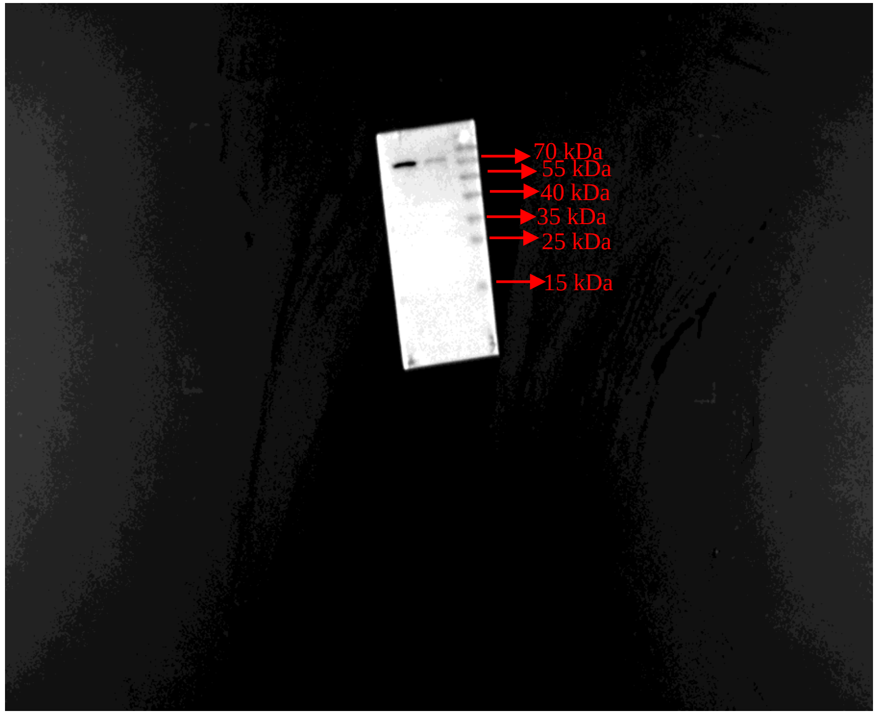

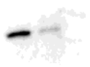

Figure S1C GAPDH

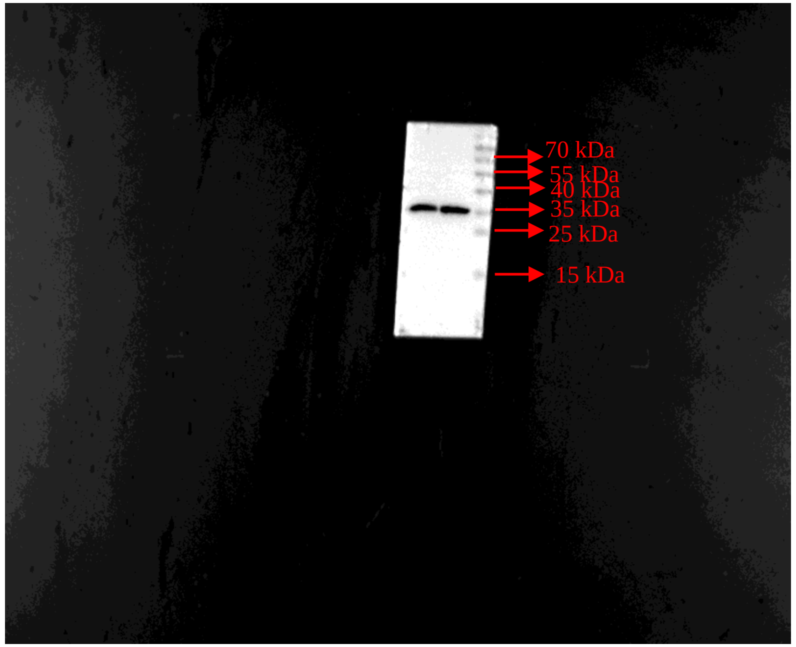

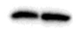

Figure S1C FOXG1

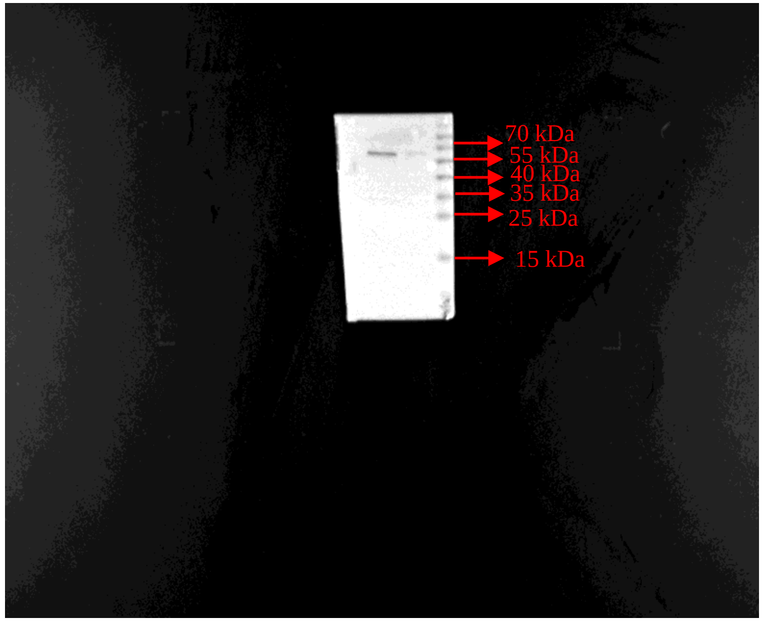

Figure S1C GAPDH

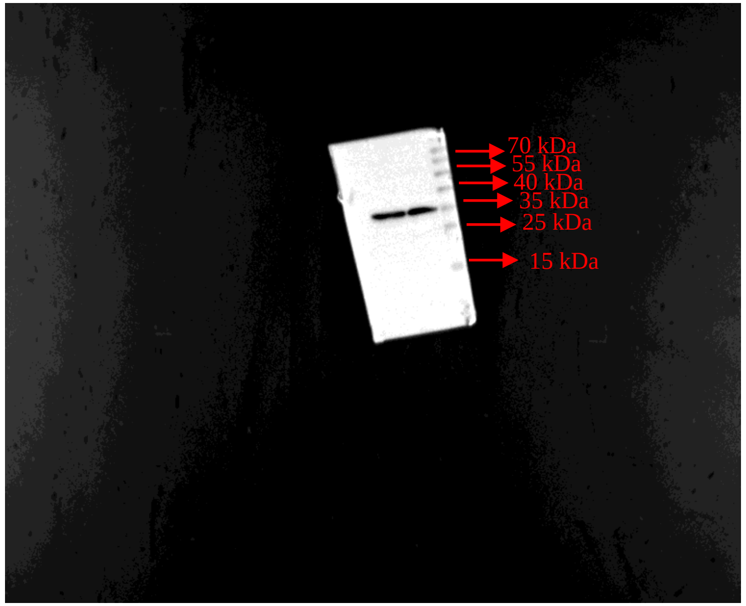

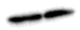

Figure S1C FOXN1

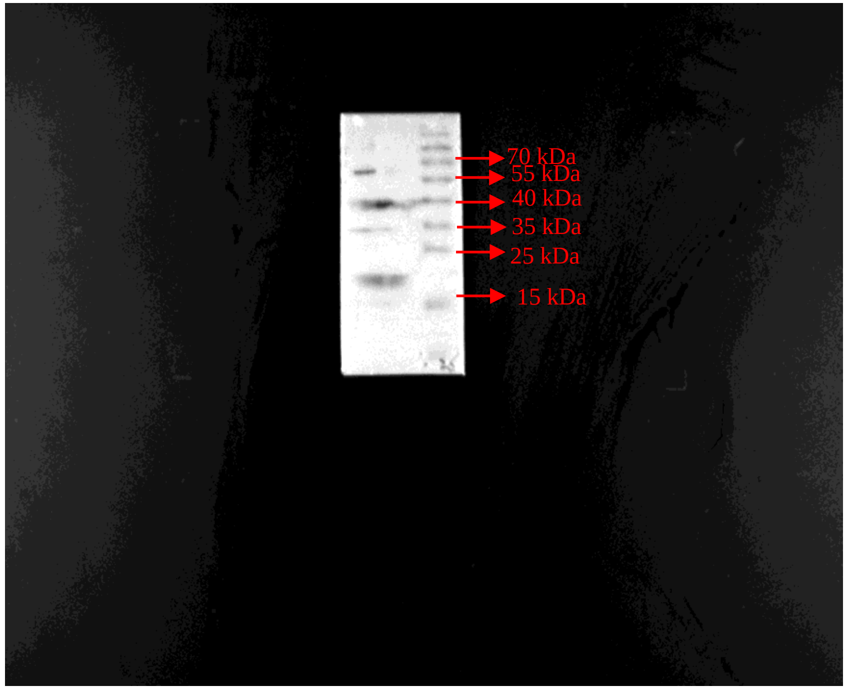

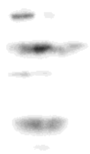

Figure S1C GAPDH

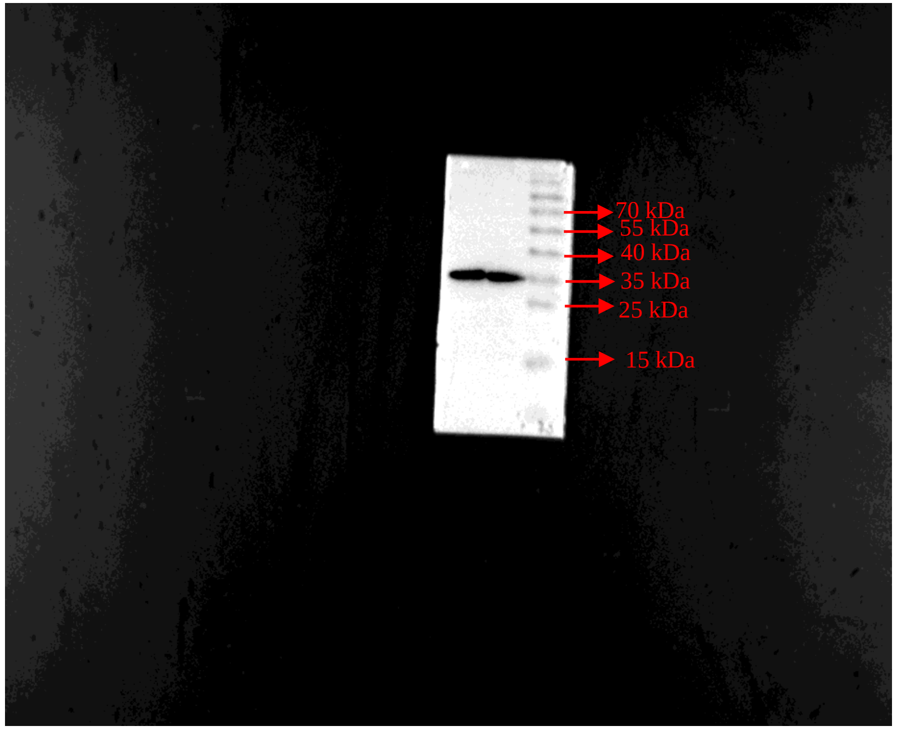

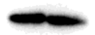

Figure S1C FOXJ3

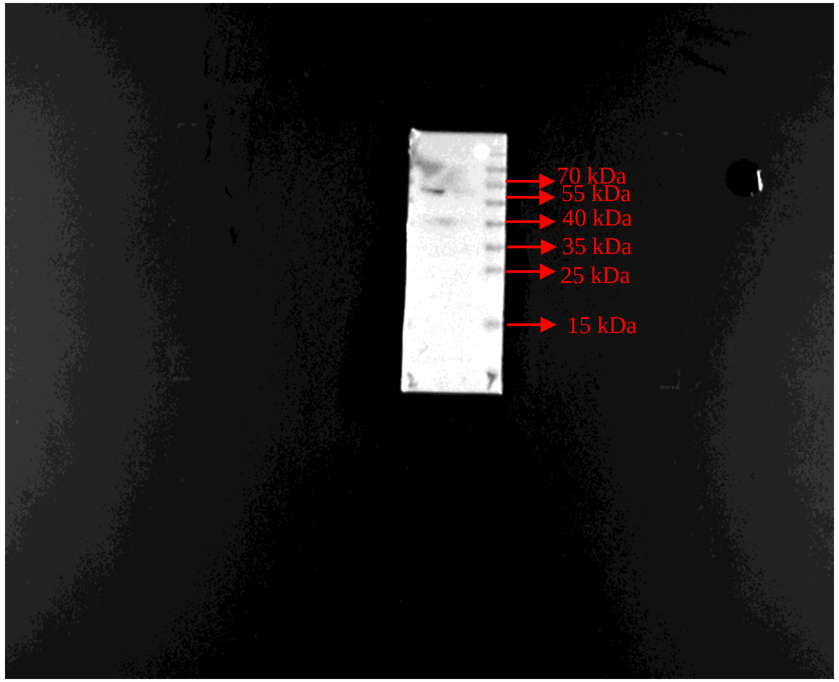

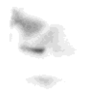

Figure S1C GAPDH

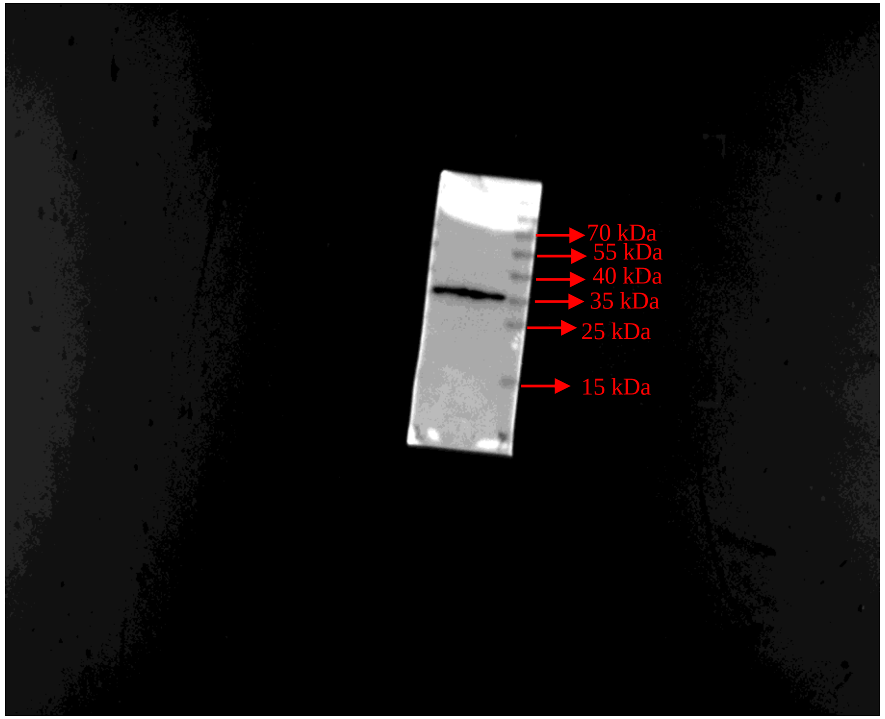

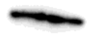

Figure S1C FOXD4L1

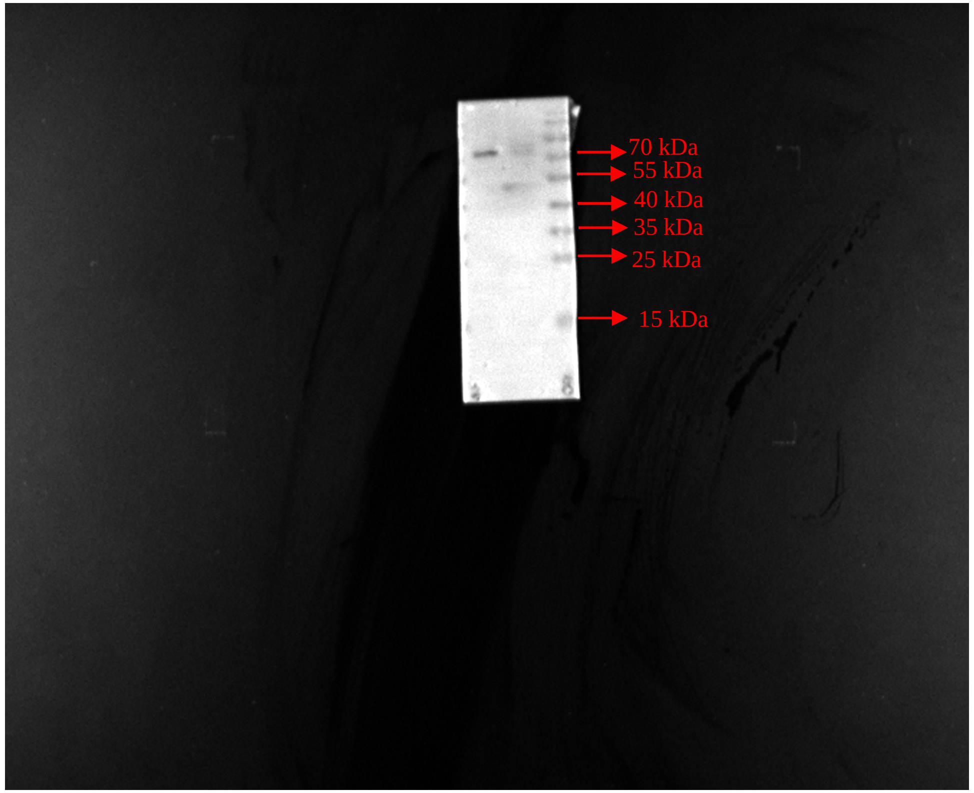

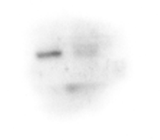

Figure S1C GAPDH

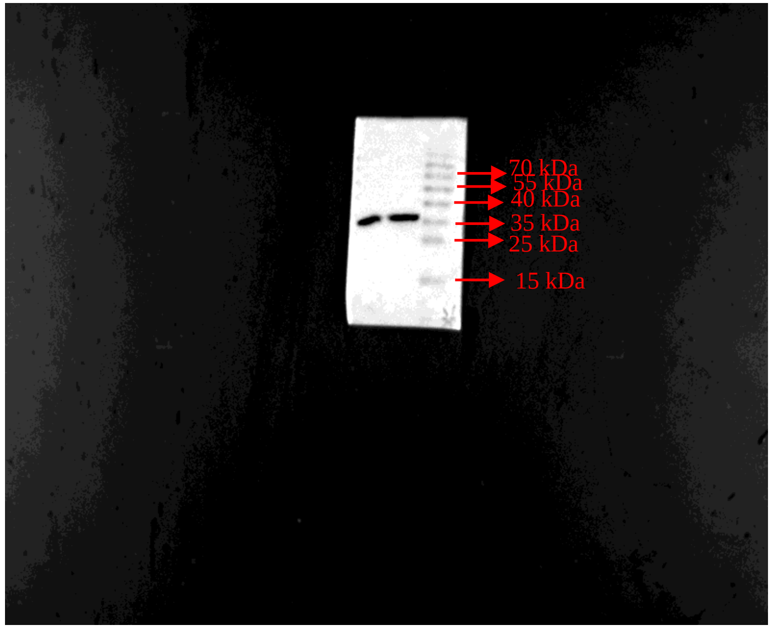

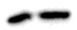

Figure S1C FOXD4L2

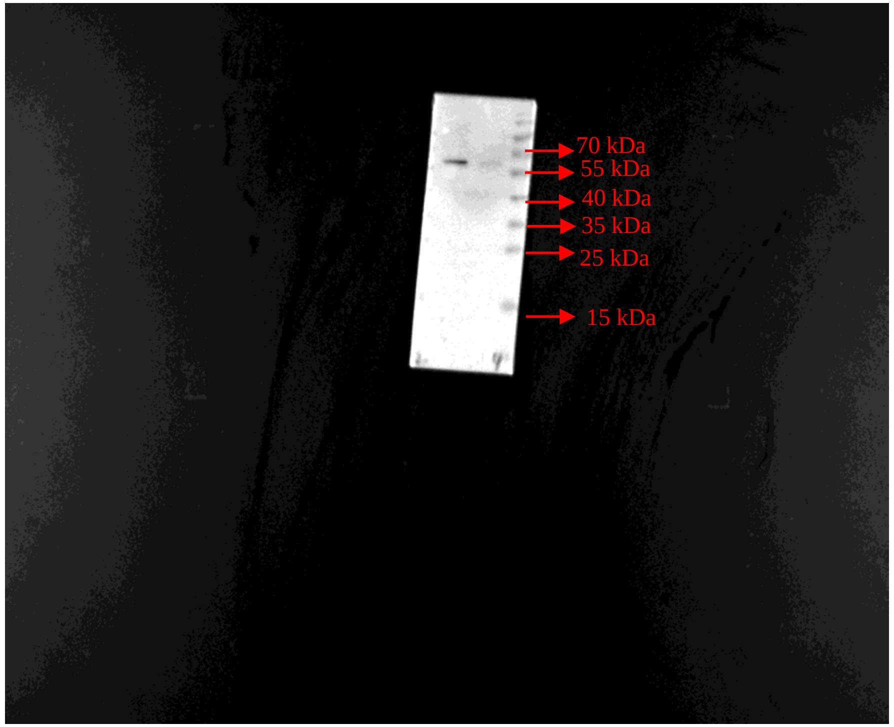

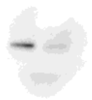

Figure S1C GAPDH

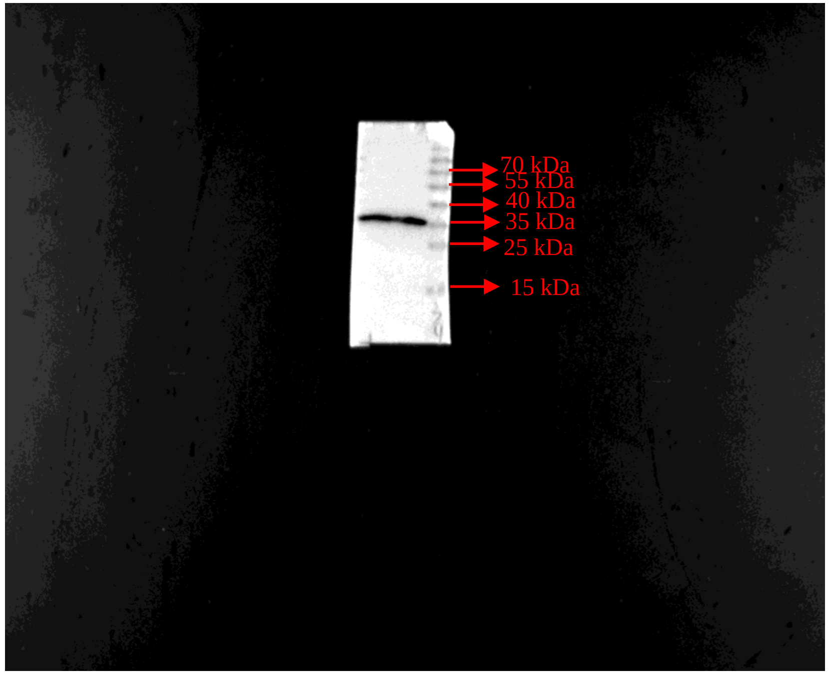

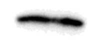

Figure S1C FOXP3

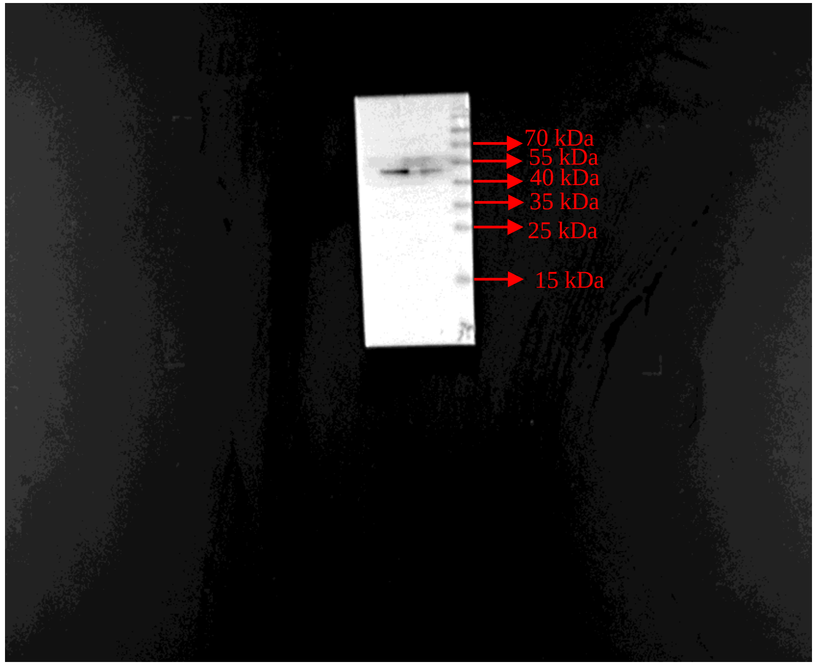

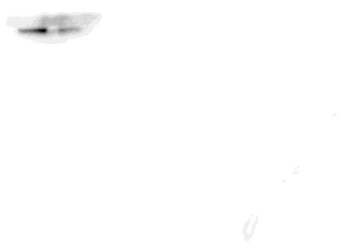

Figure S1C GAPDH

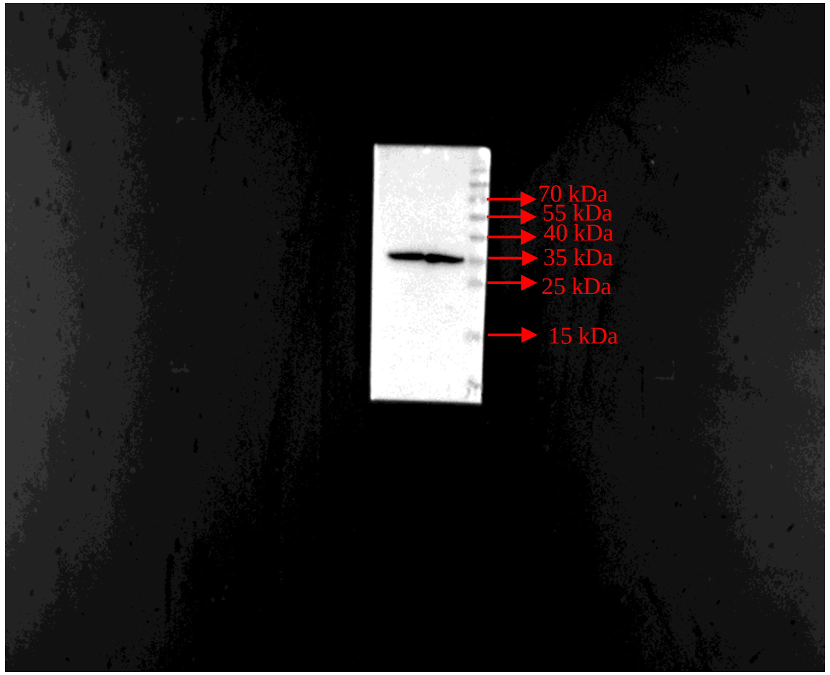

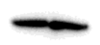

Figure S1C FOXI1

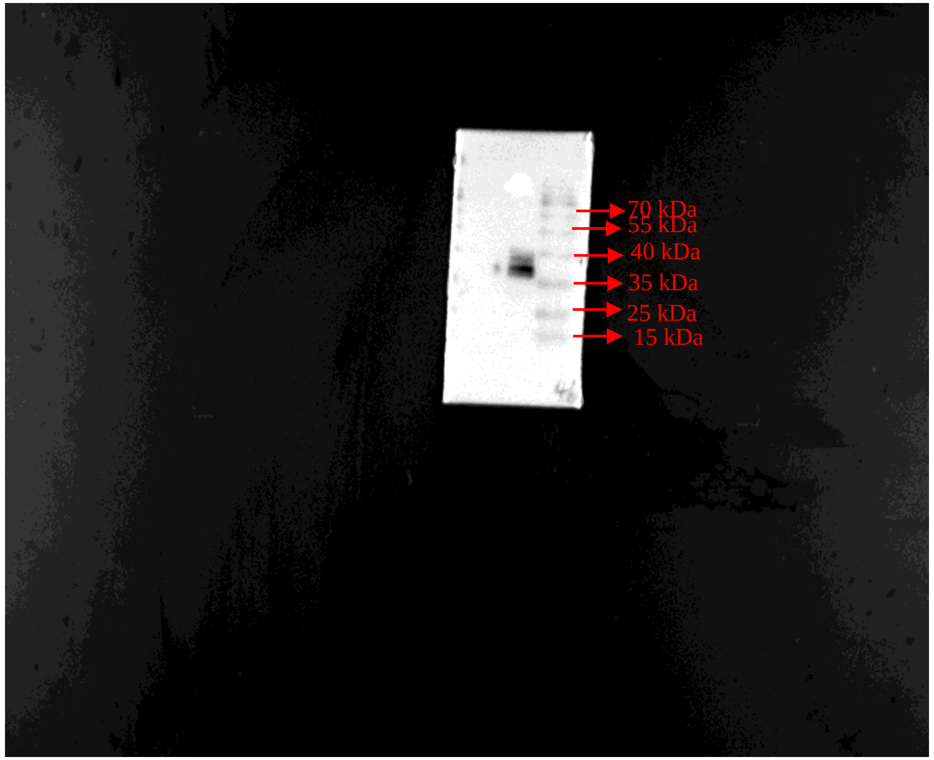

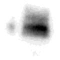

Figure S1C GAPDH

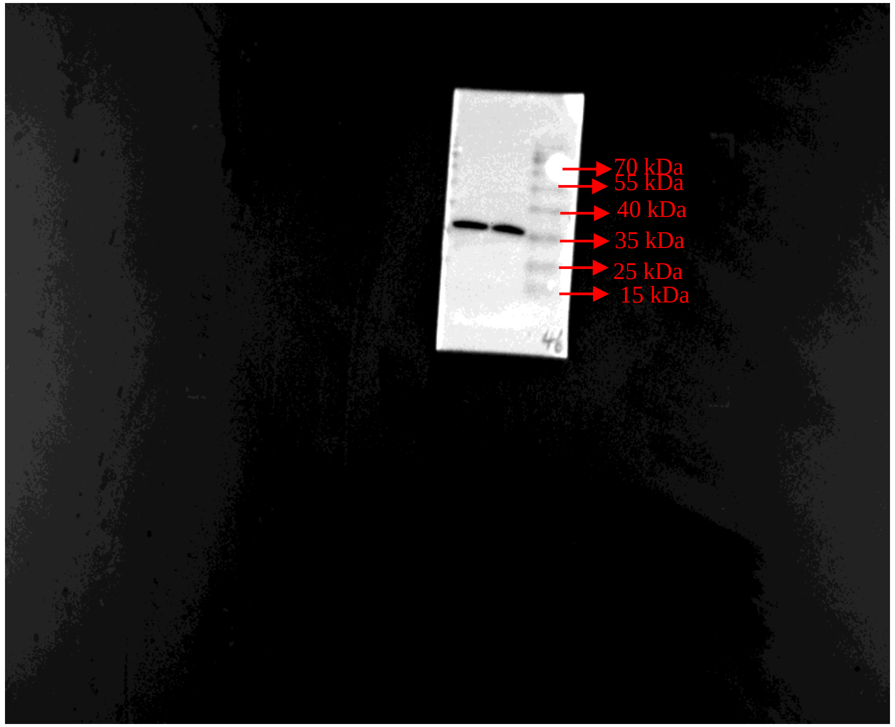

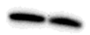

Figure S1C FOXC2

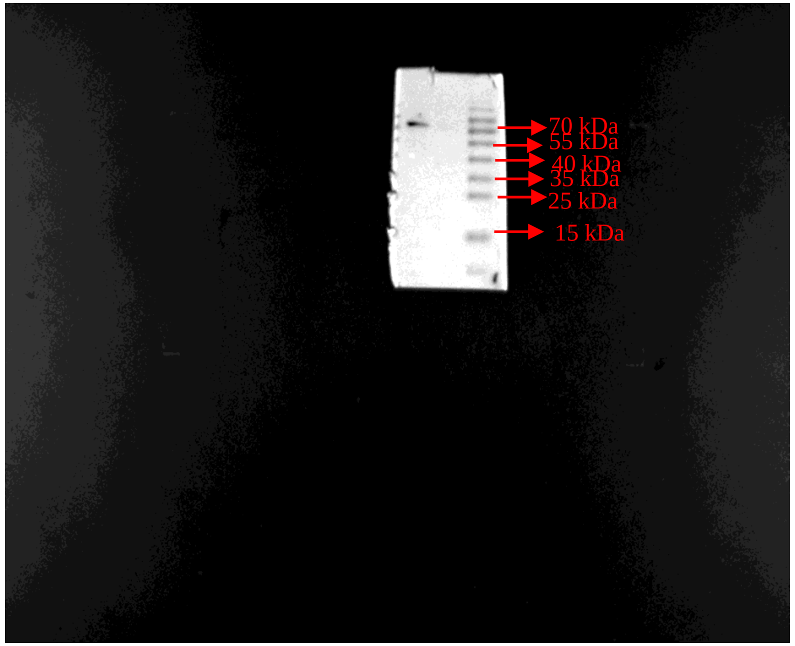

Figure S1C GAPDH

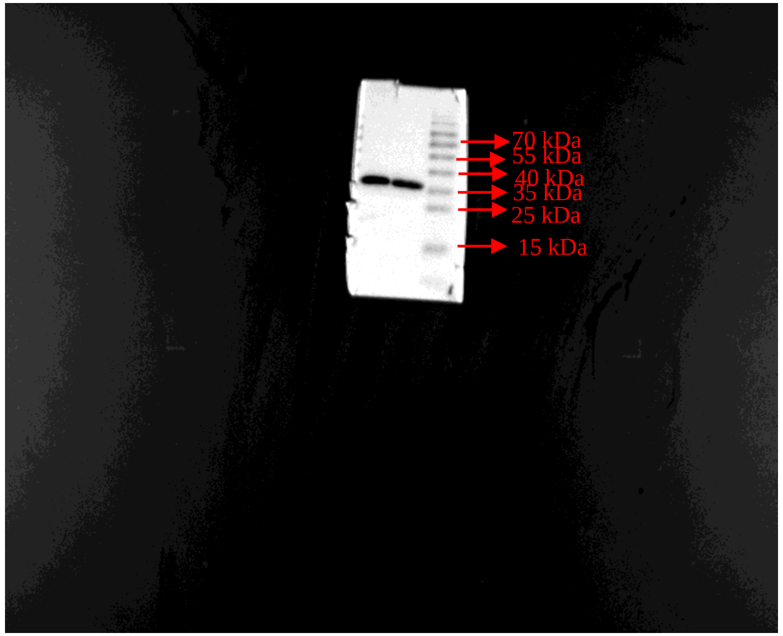

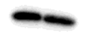

Figure 1H FOXP3

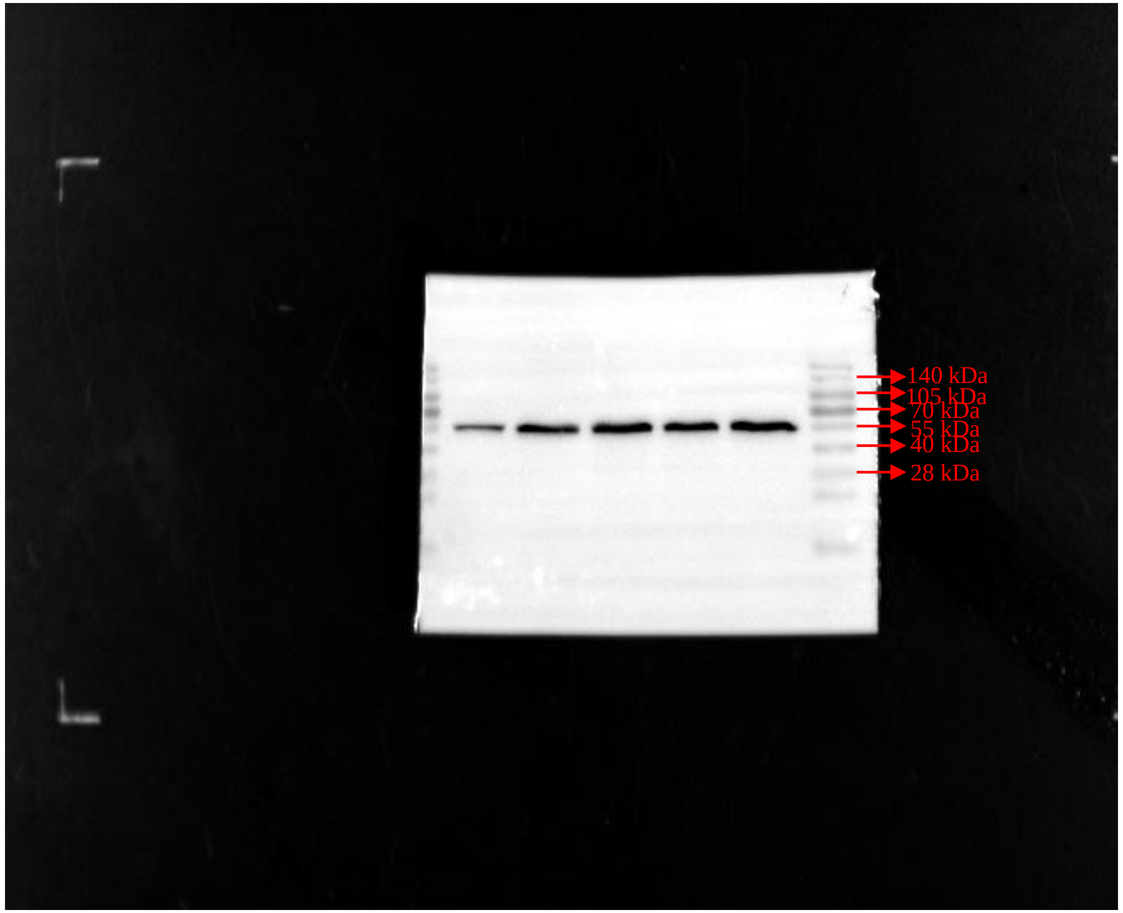

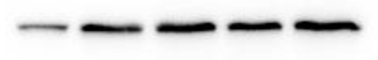

Figure 1H GAPDH

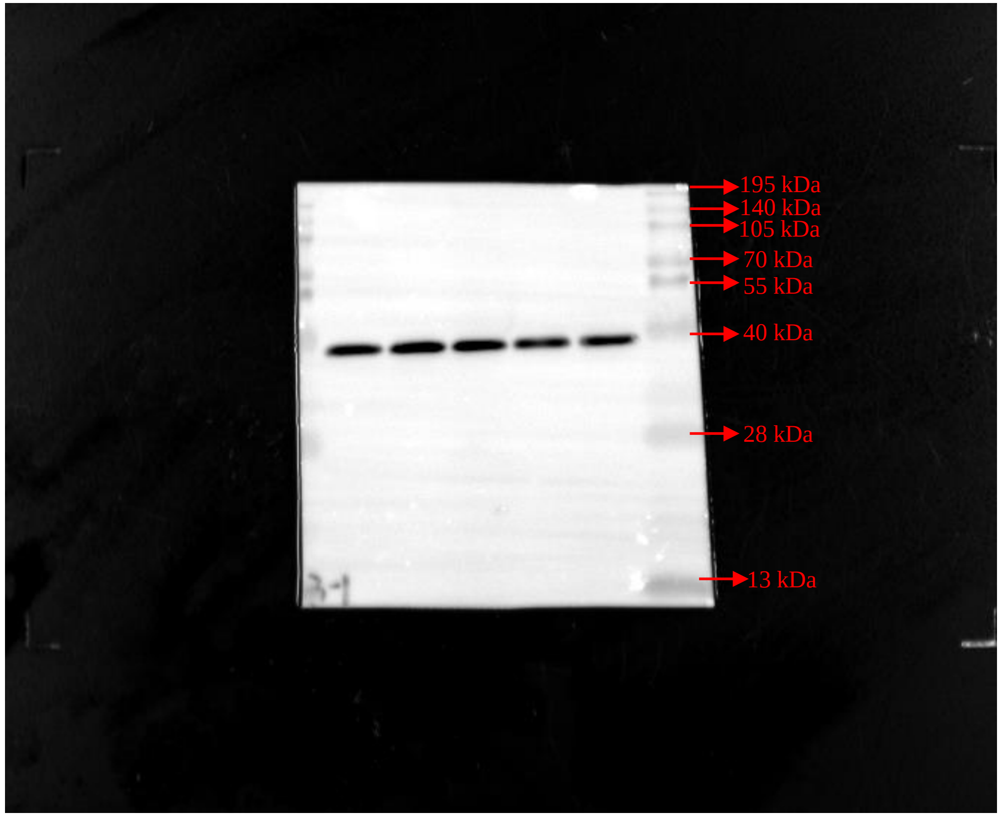

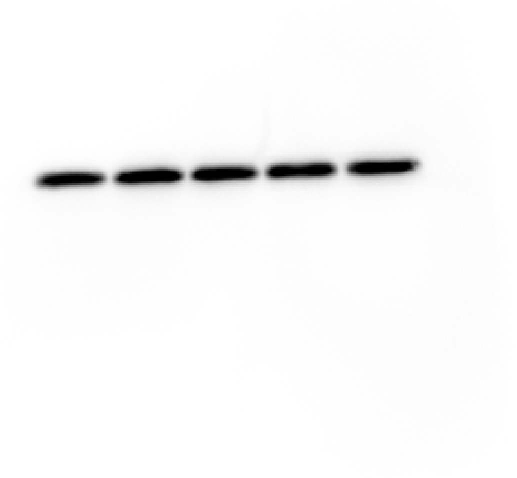

Figure2F U87 FOXP3

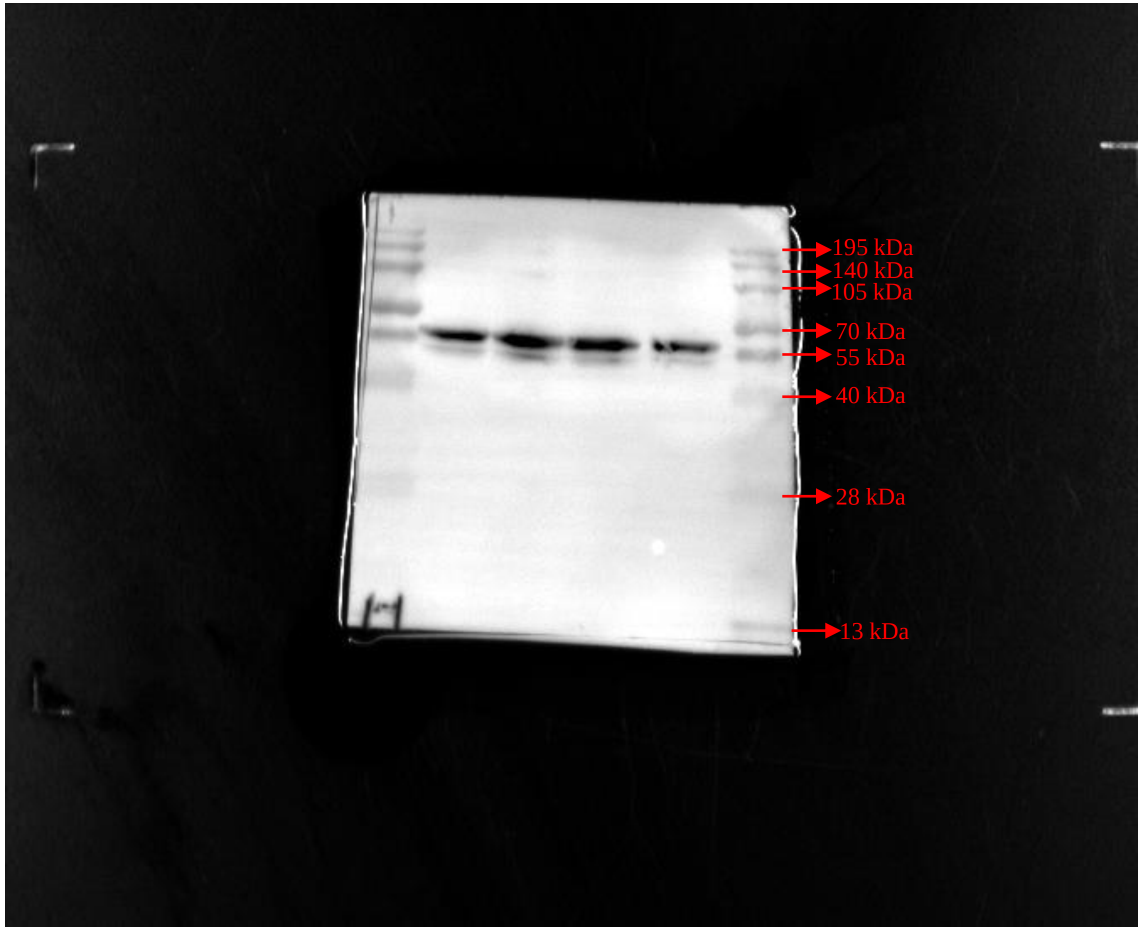

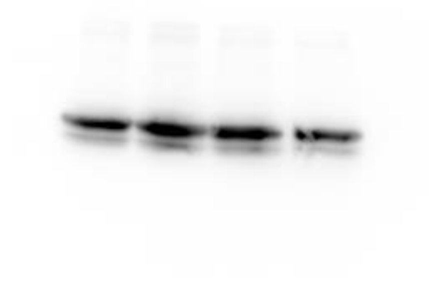

Figure2F U87 SLC40A1

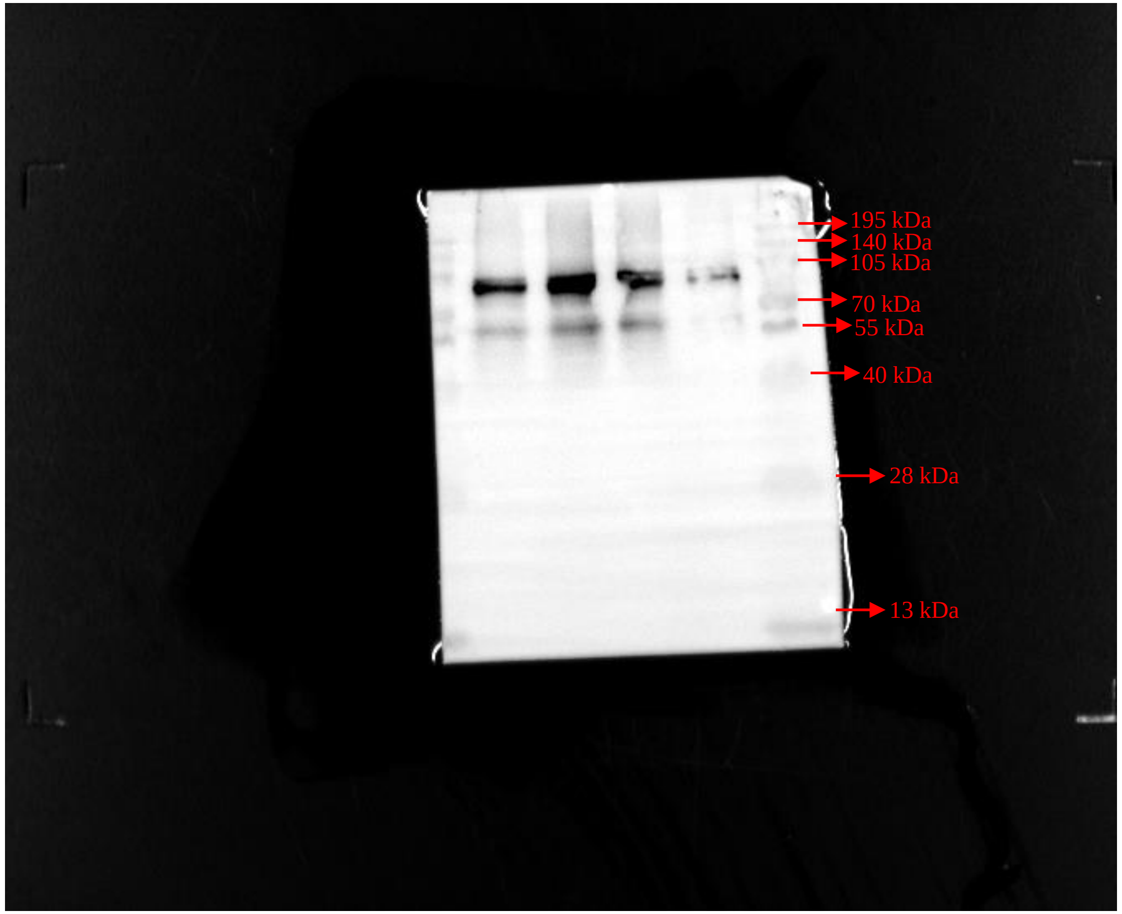

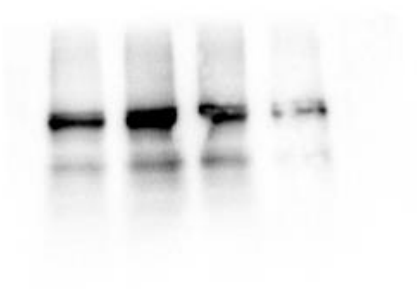

Figure2F U87 SLC7A11

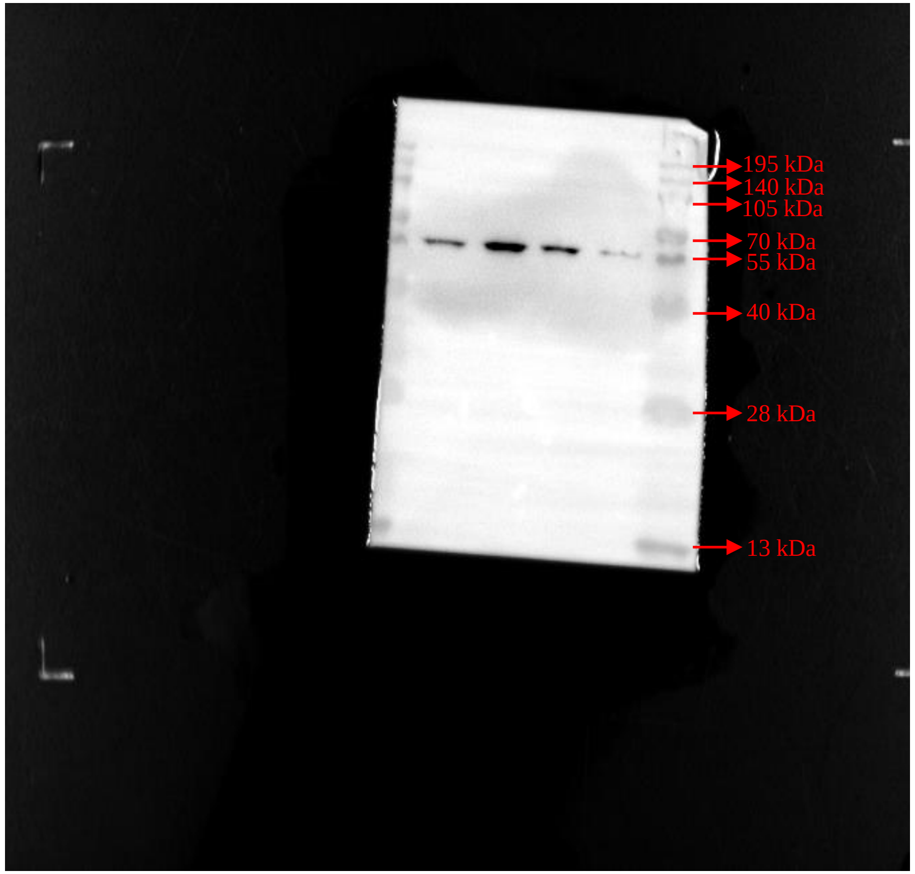

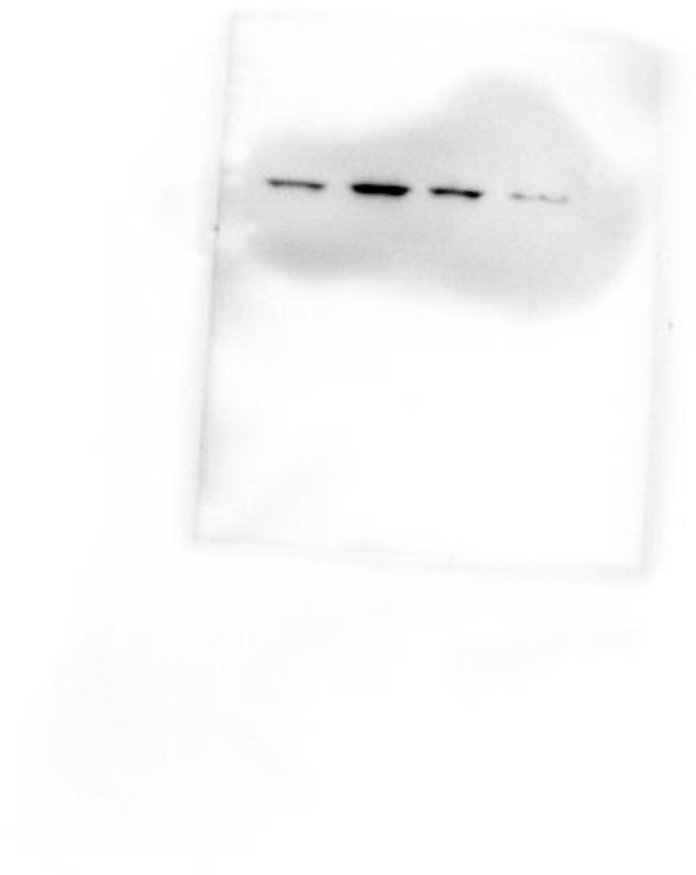

Figure2F U87 GPX4

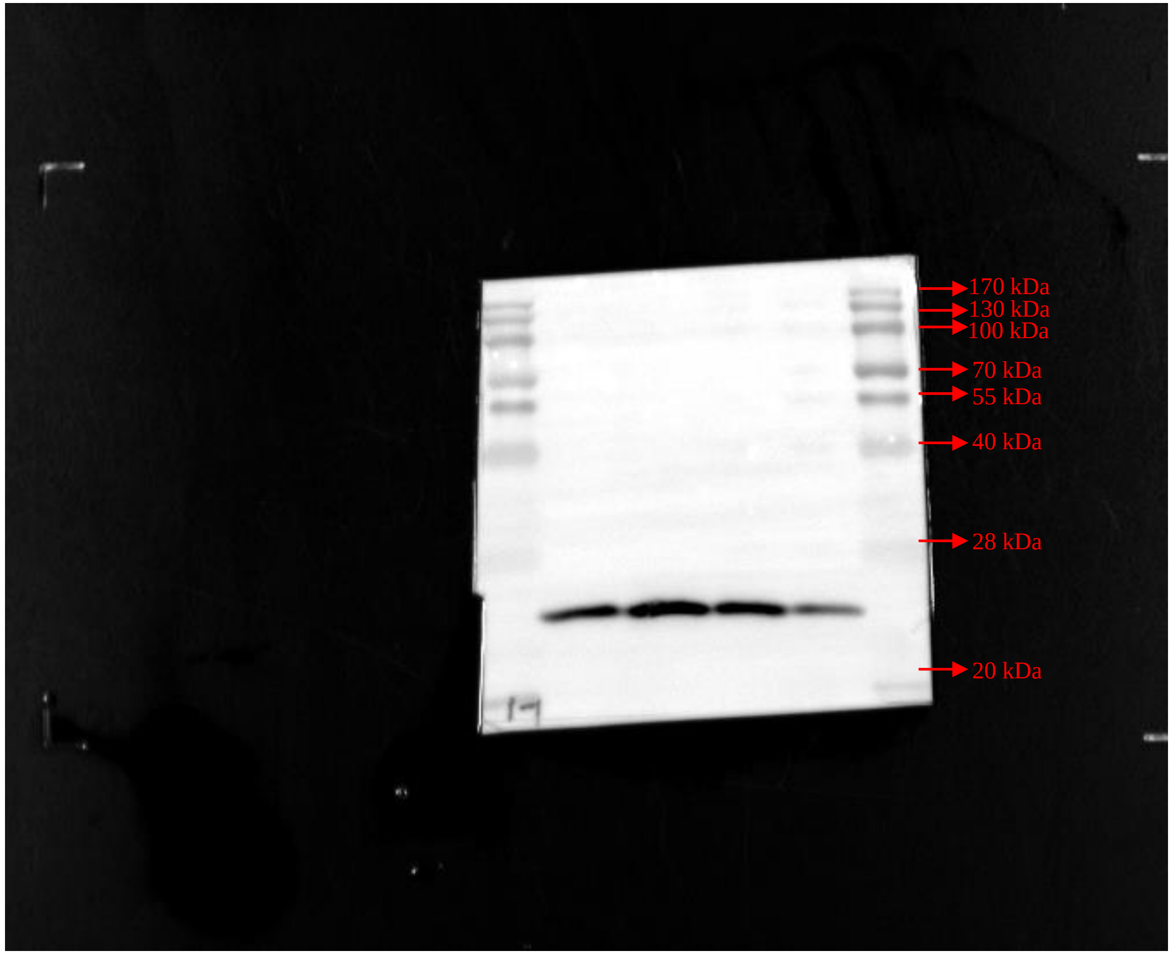

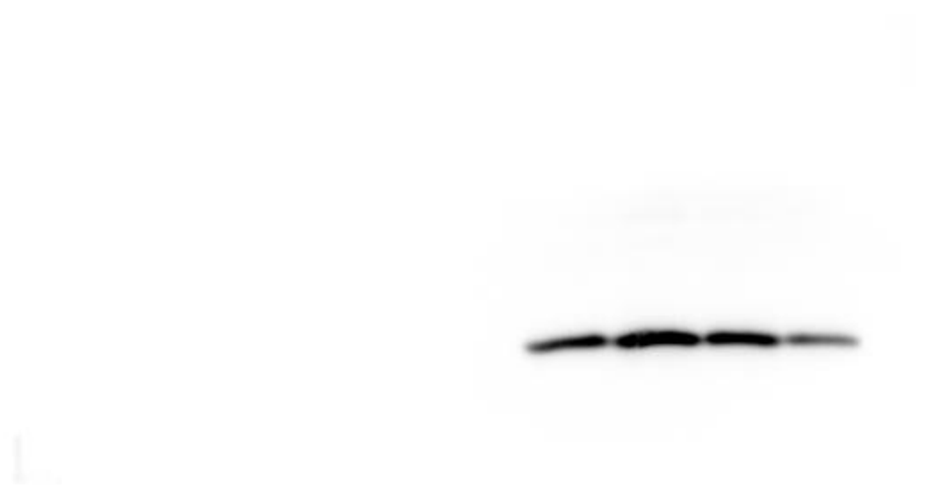

Figure2F U87 FTH1

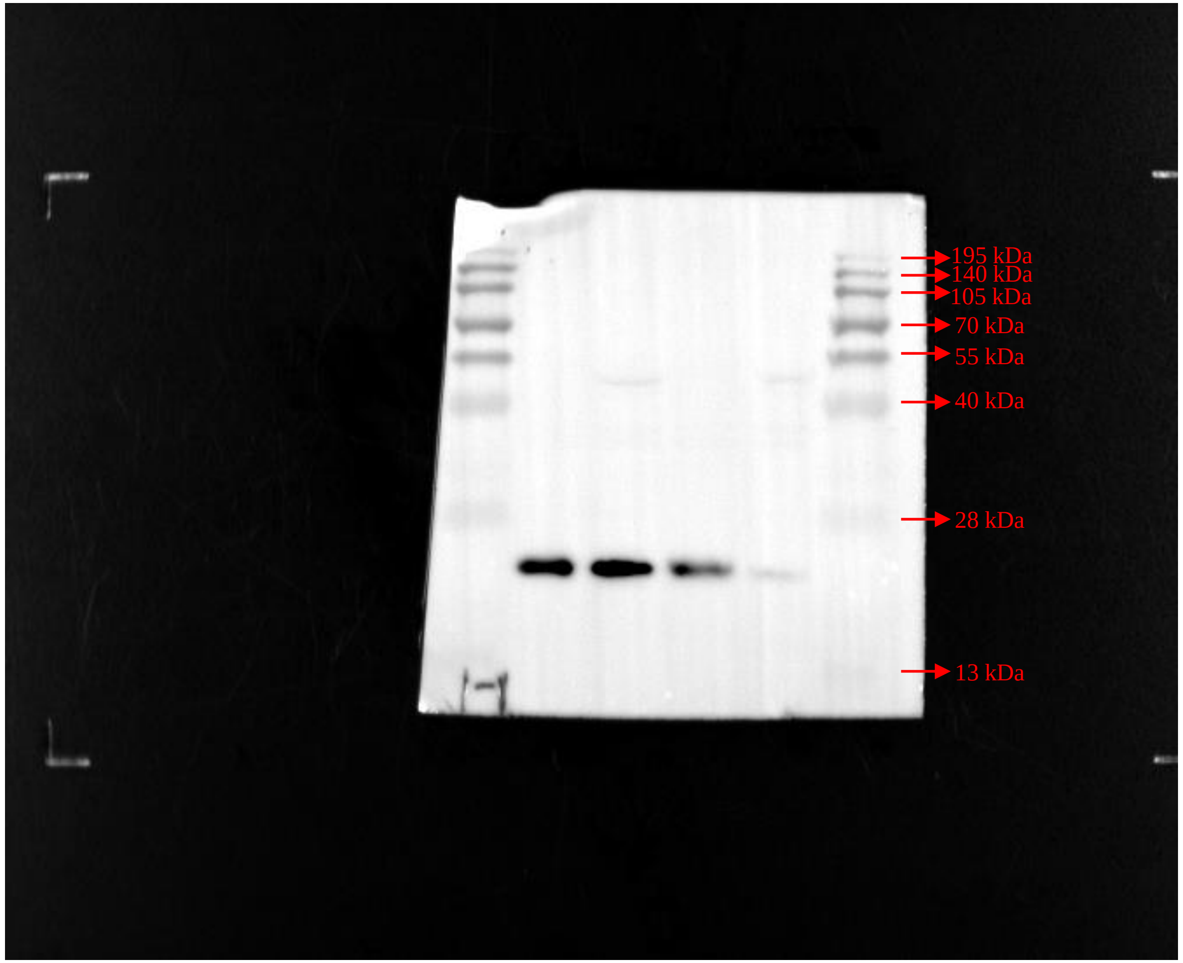

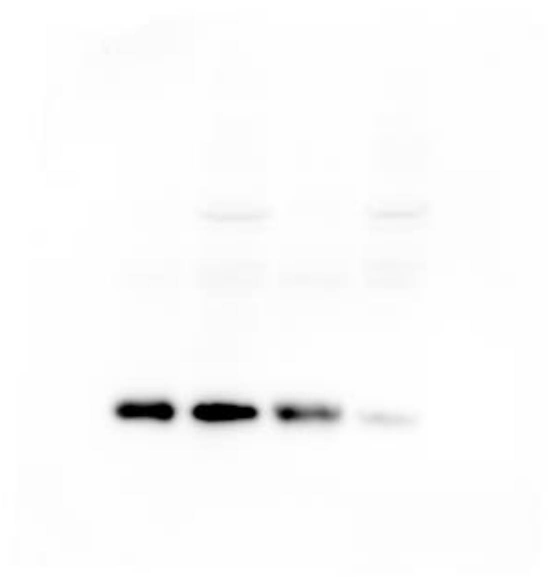

Figure2F U87 GAPDH

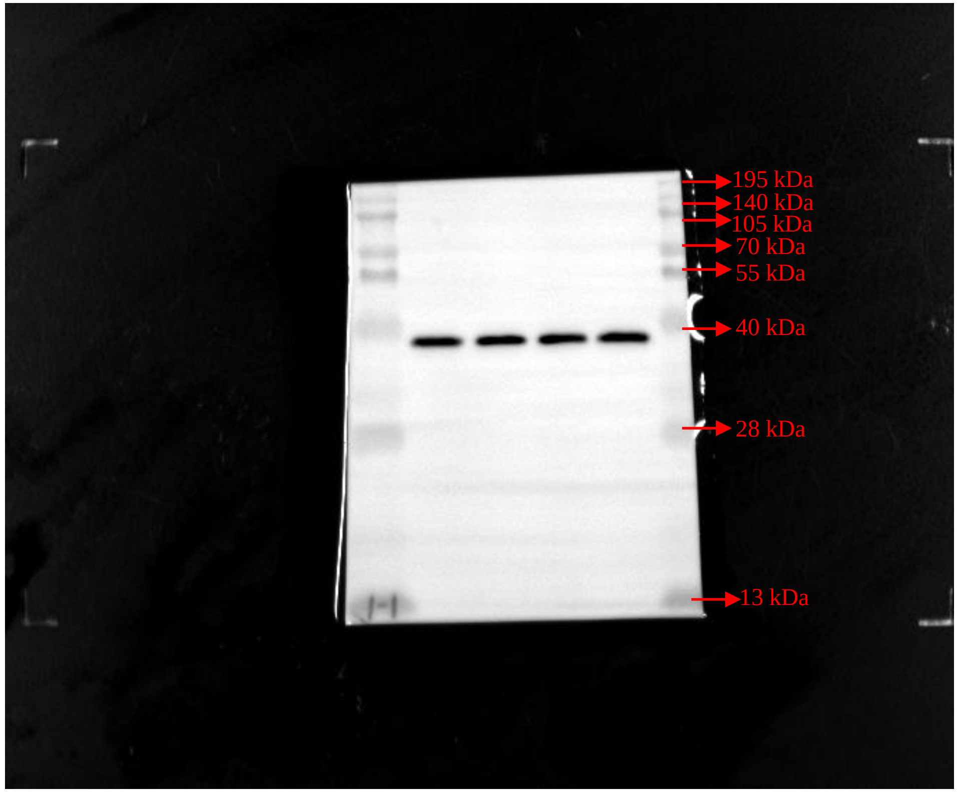

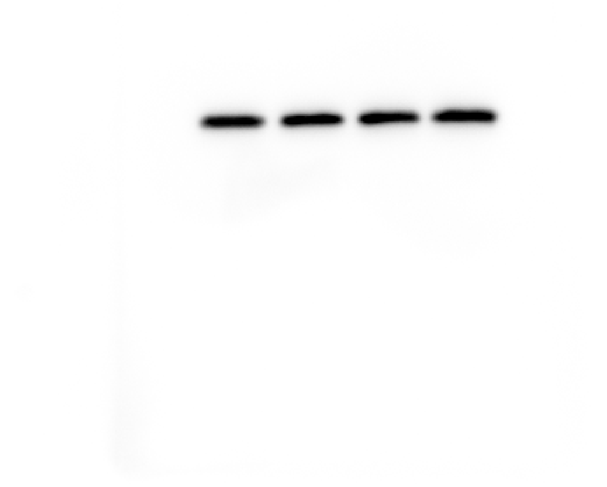

Figure2F LN229 FOXP3

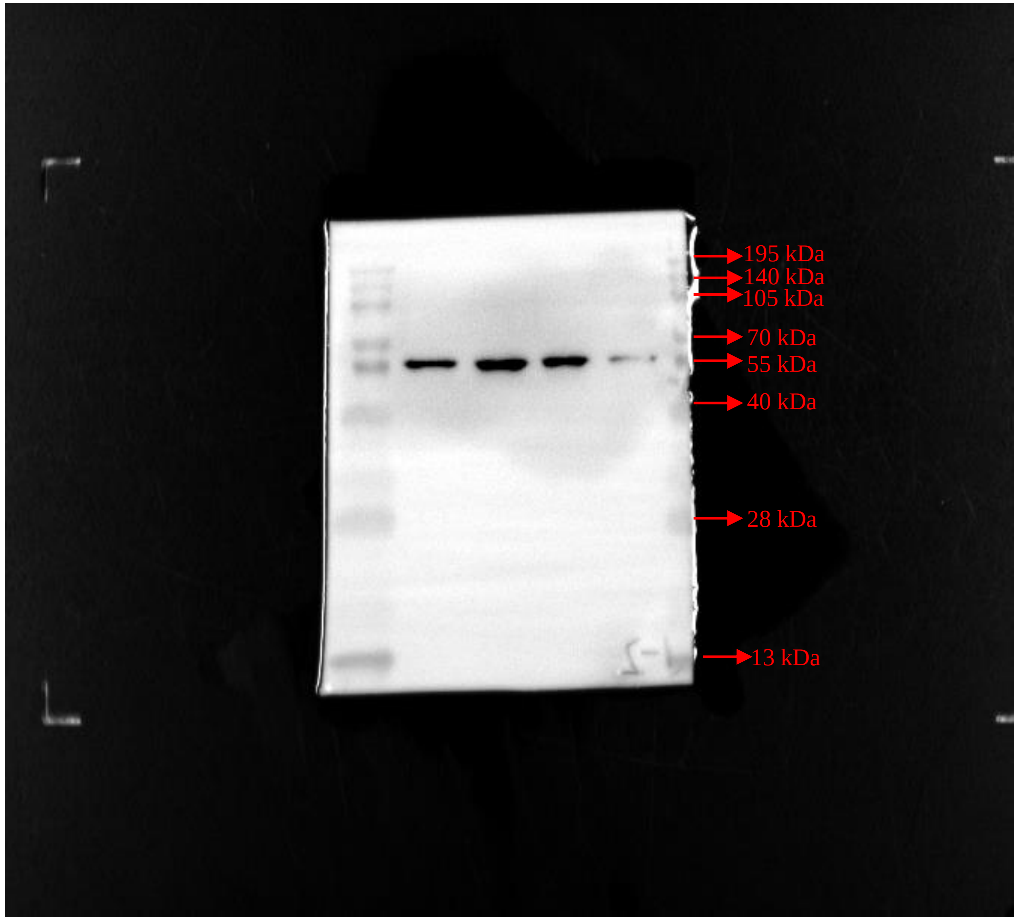

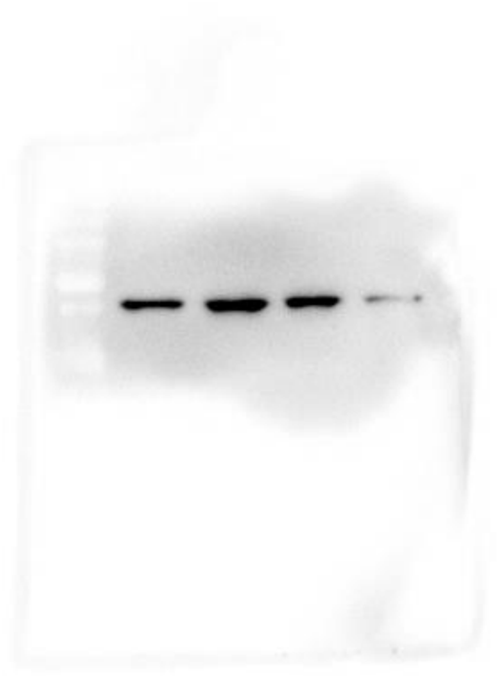

Figure2F LN229 FTH1

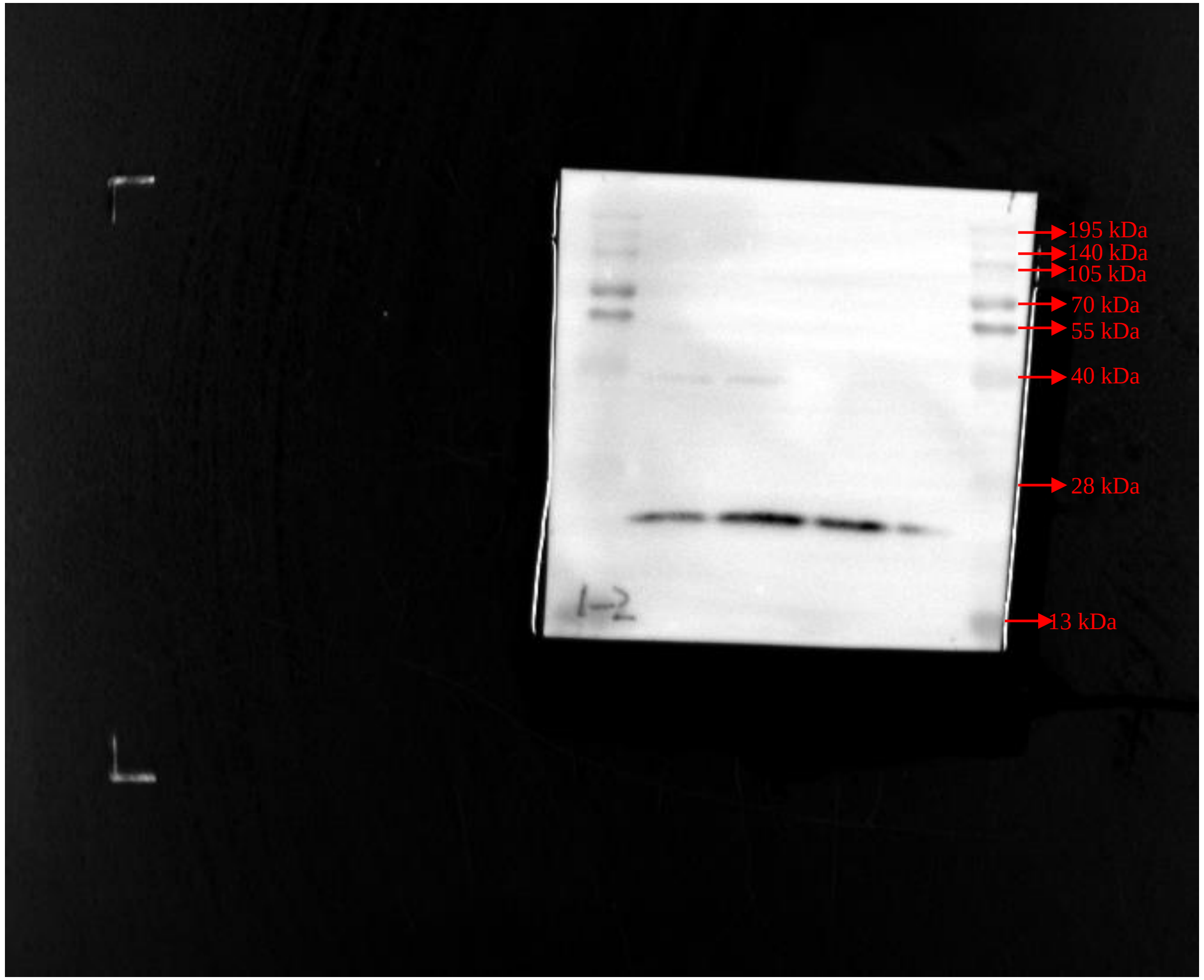

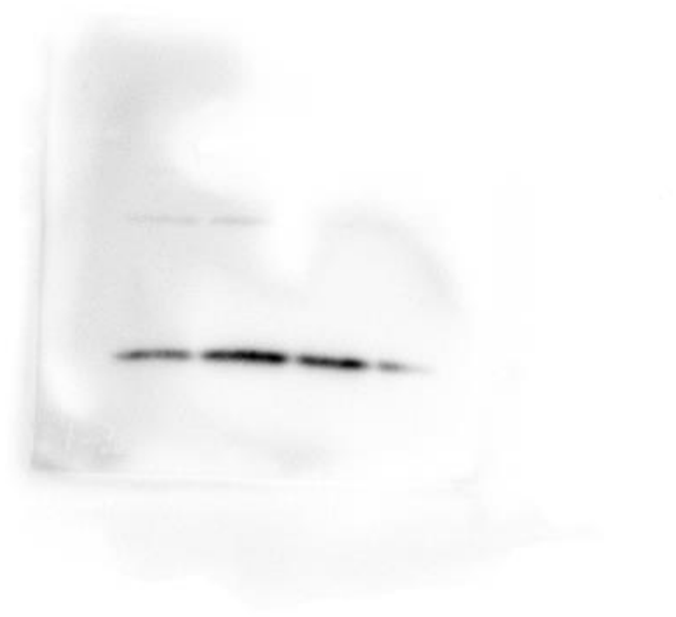

Figure2F LN229 GPX4

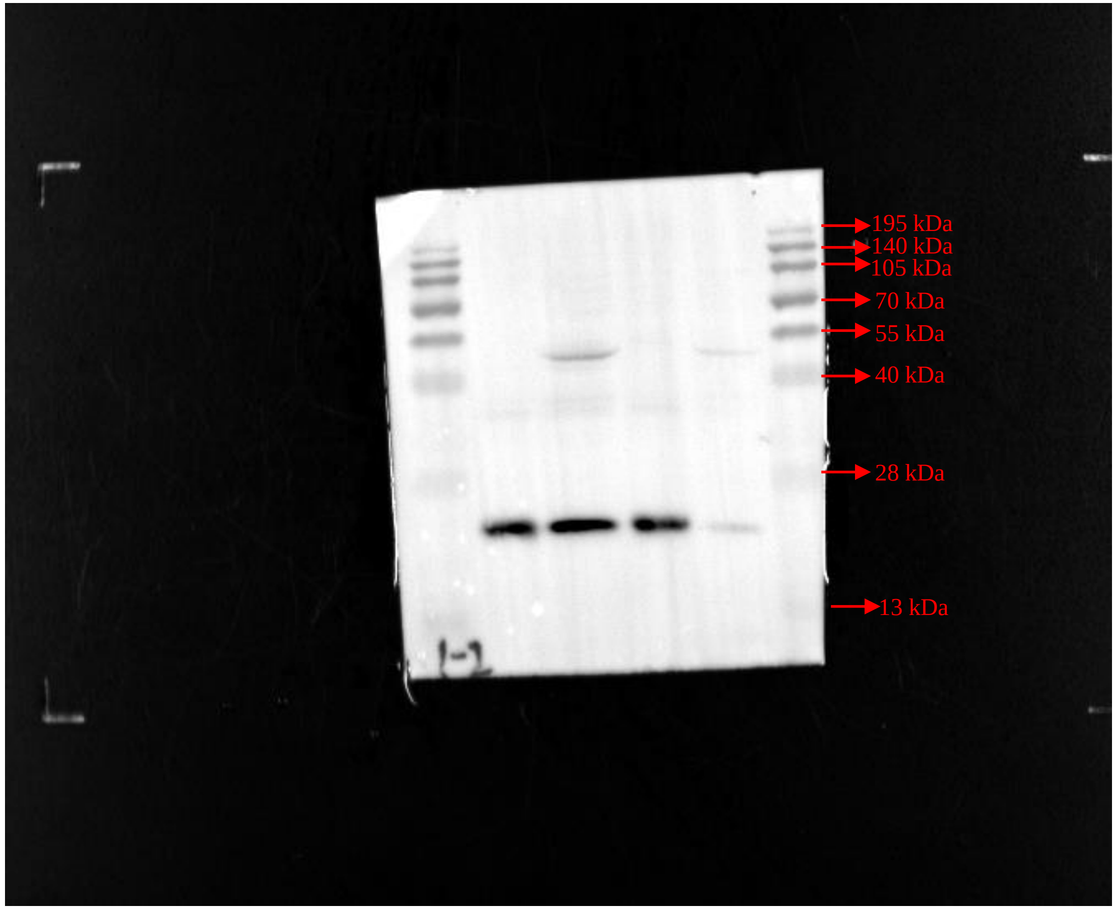

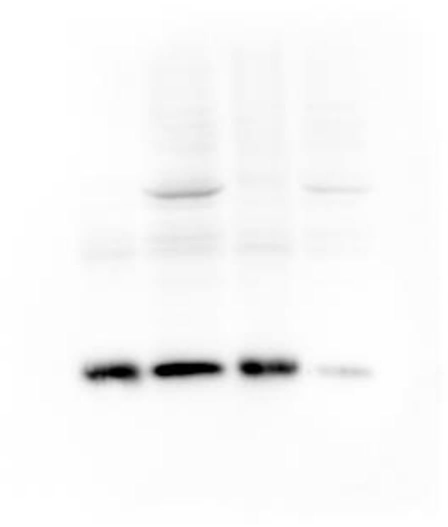

Figure2F LN229 SLC7A11

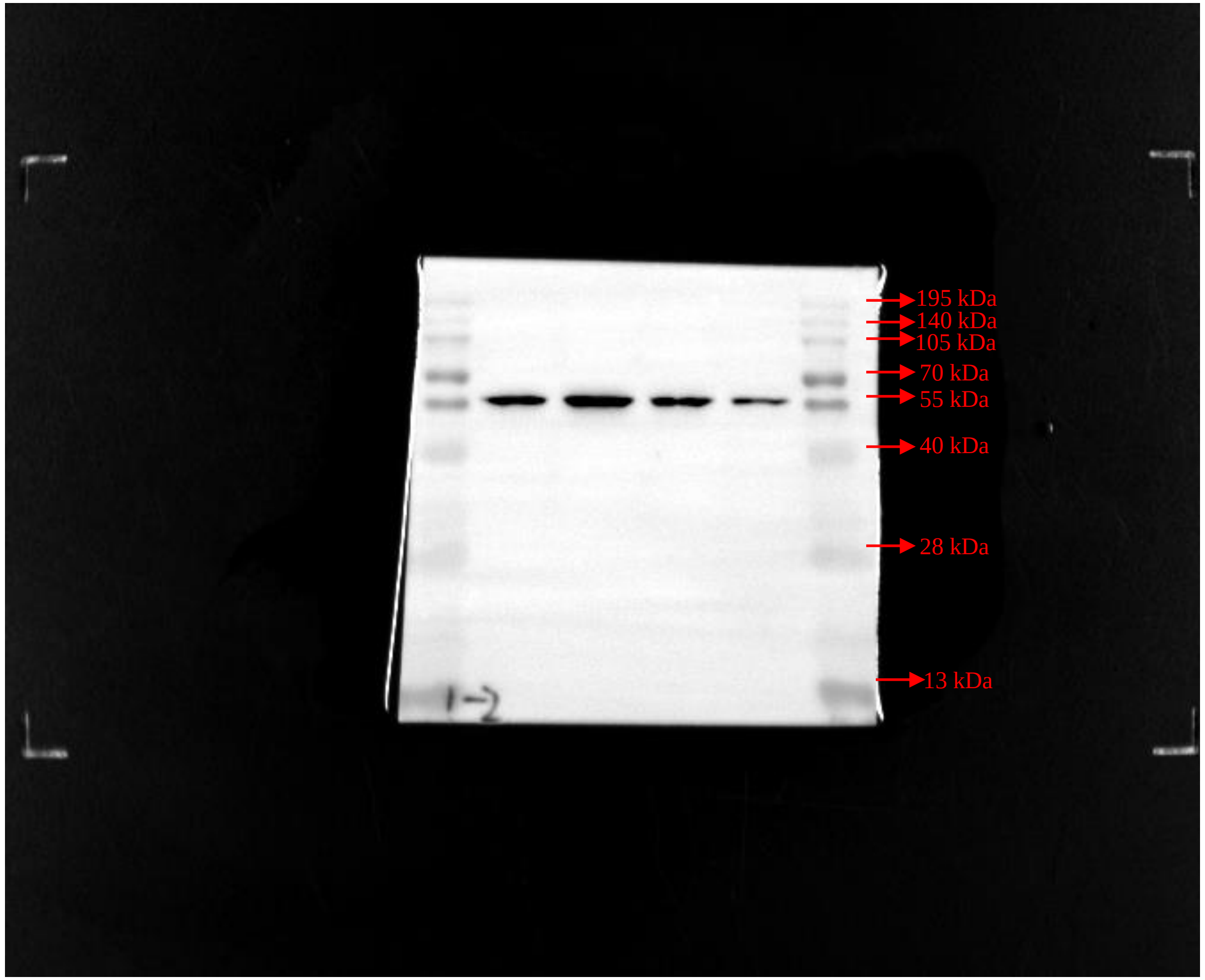

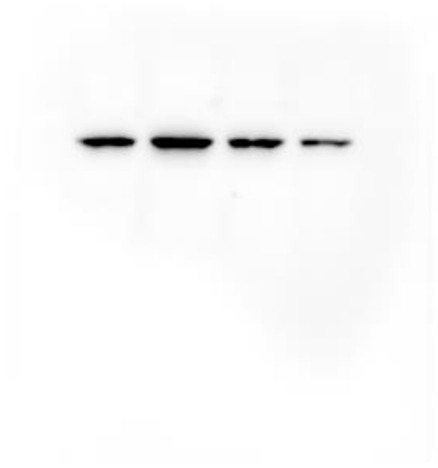

Figure2F LN229 SLC40A1

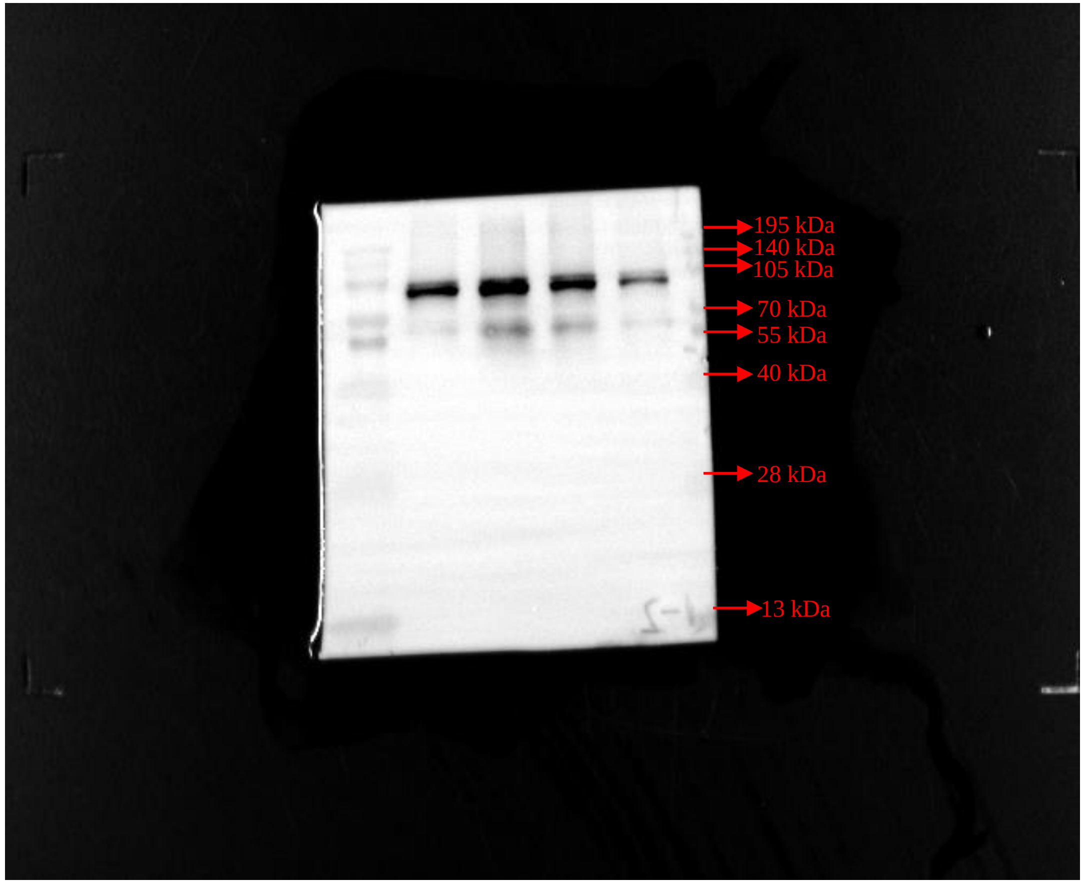

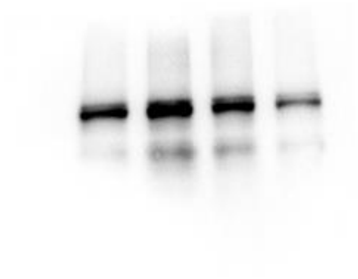

Figure2F LN229 GAPDH

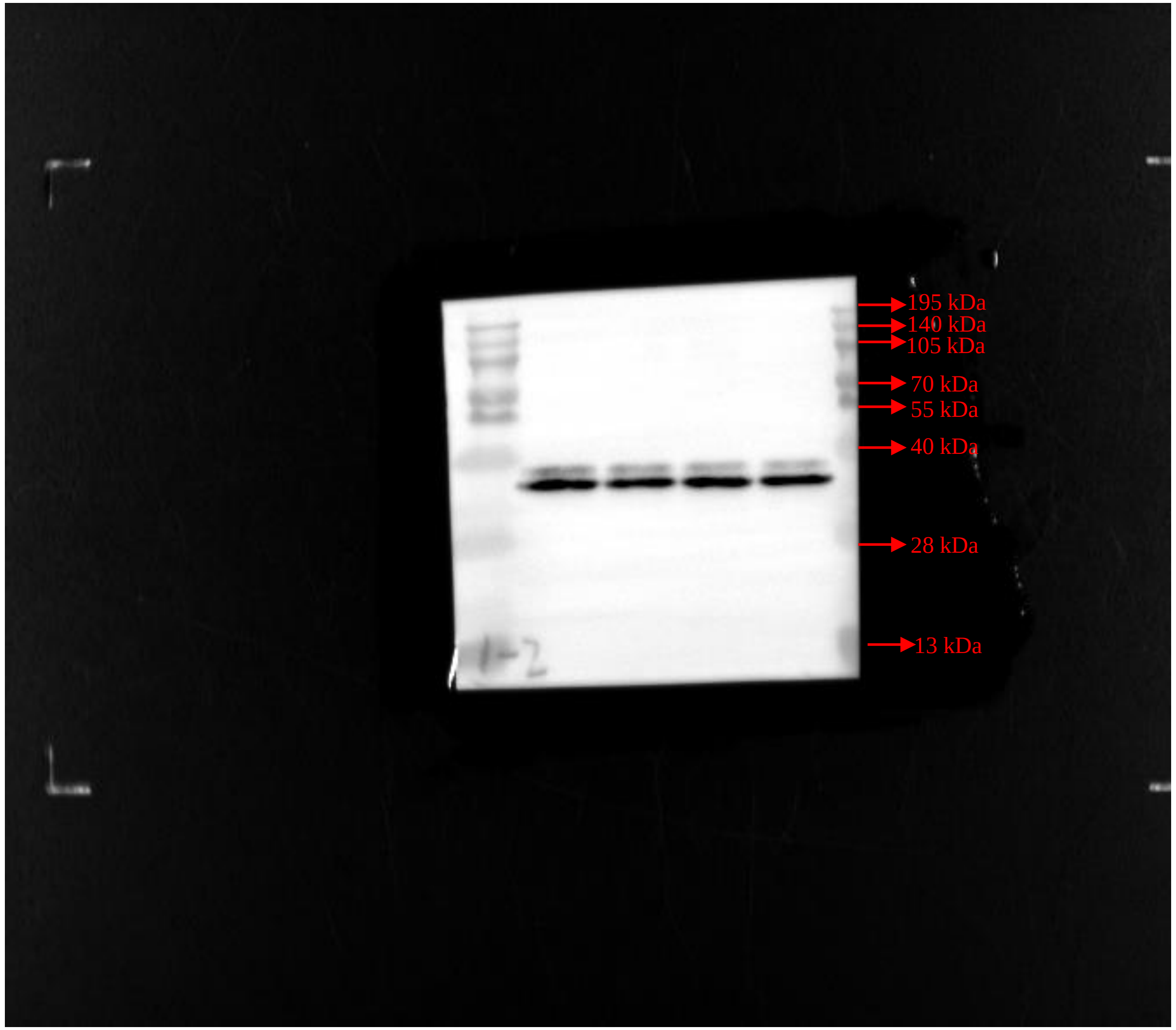

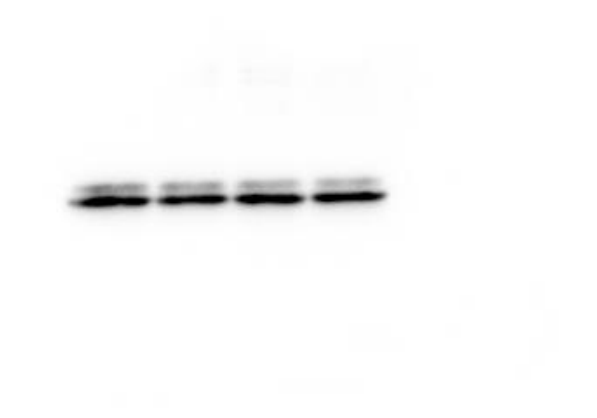

Figure 6F U87 FOXP3

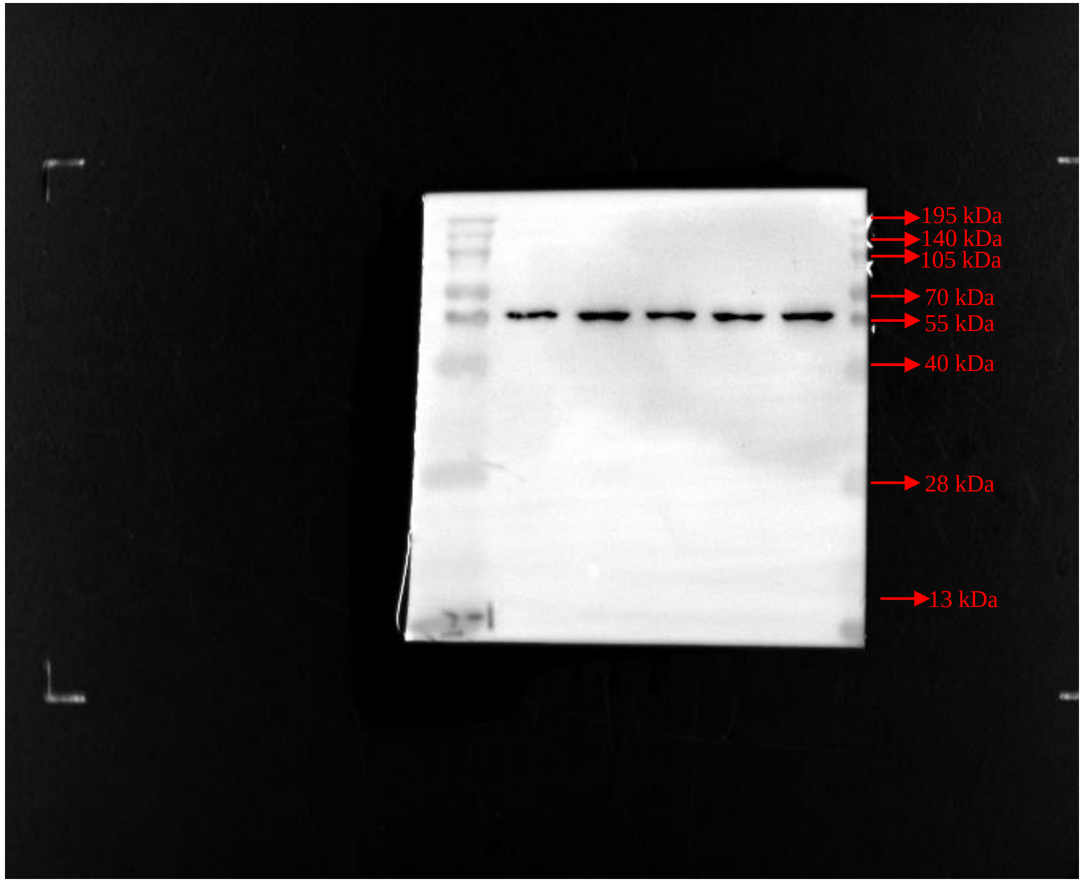

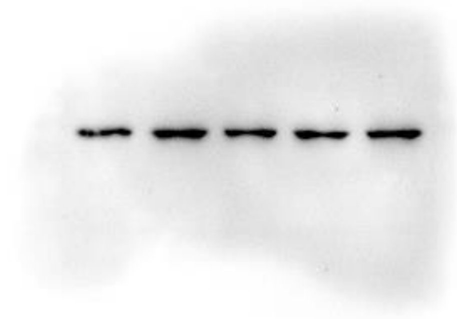

Figure 6F U87 SLC7A11

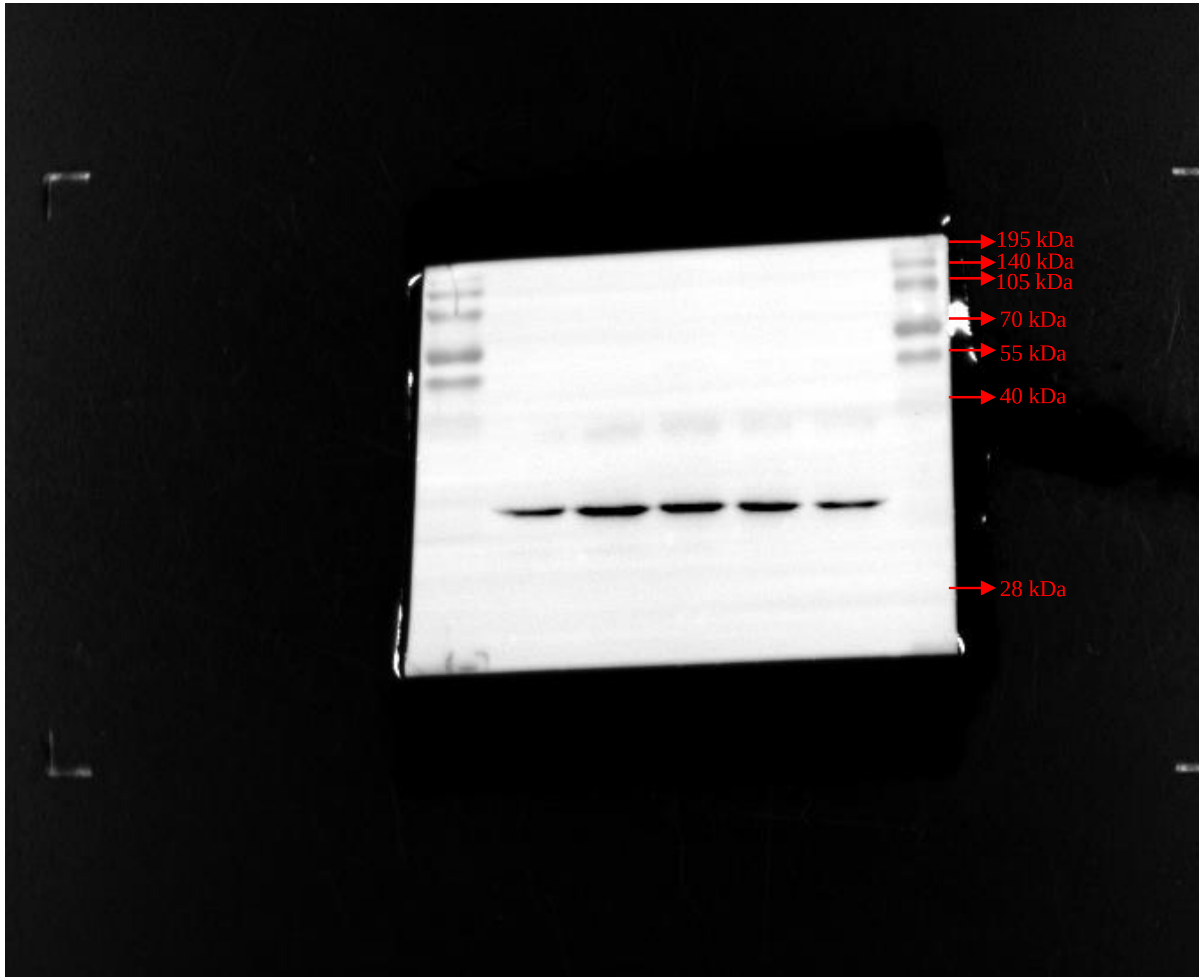

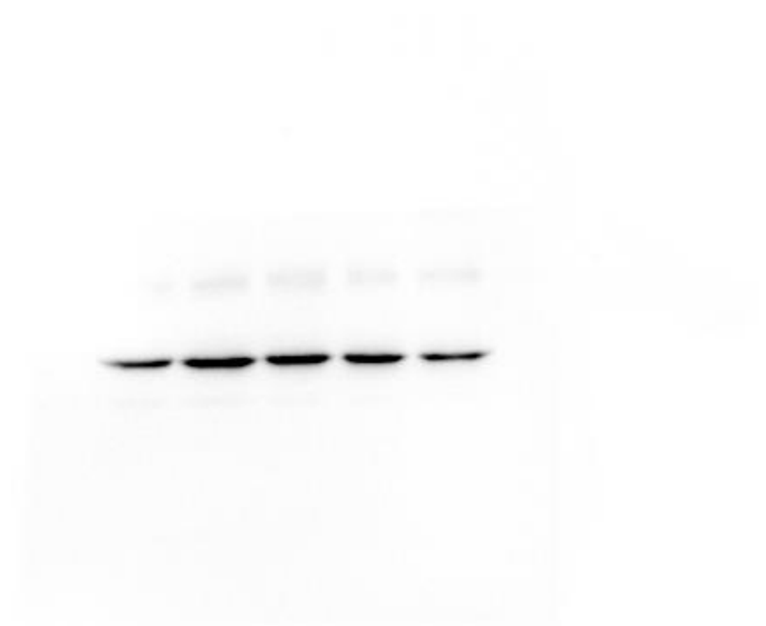

Figure 6F U87 GPX4

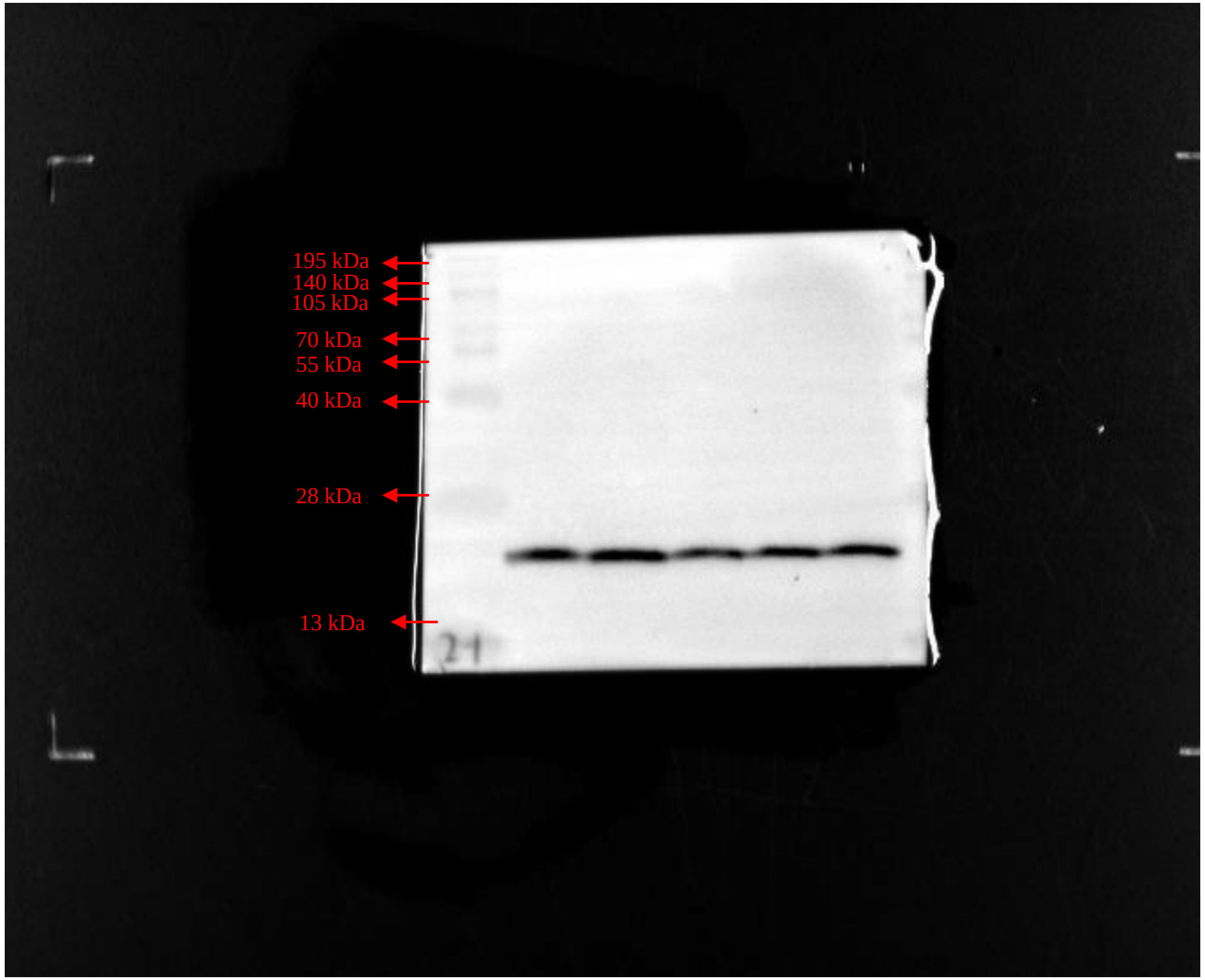

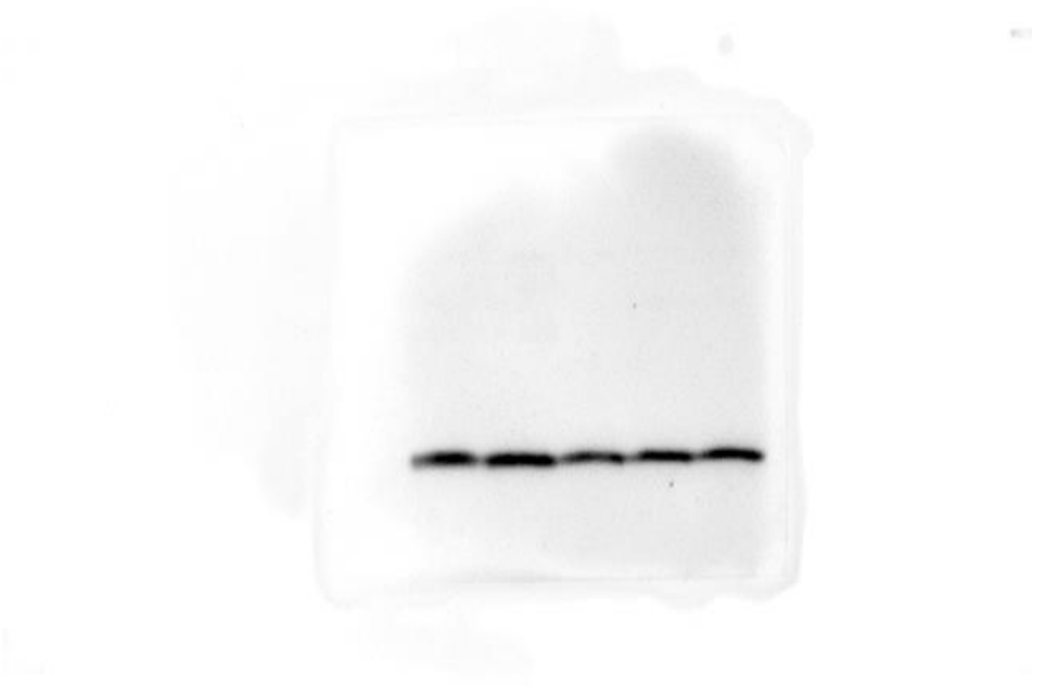

Figure 6F U87 FTH1

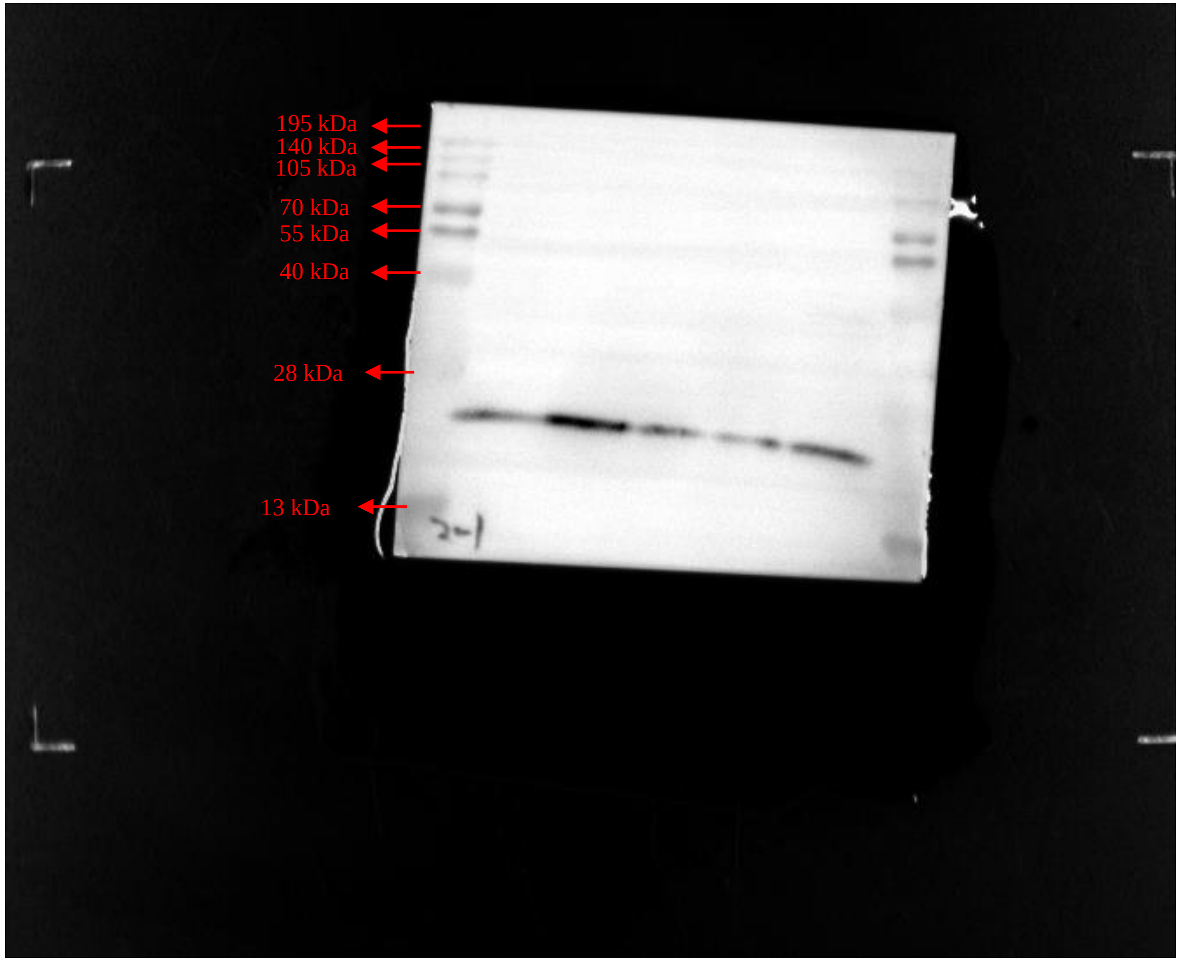

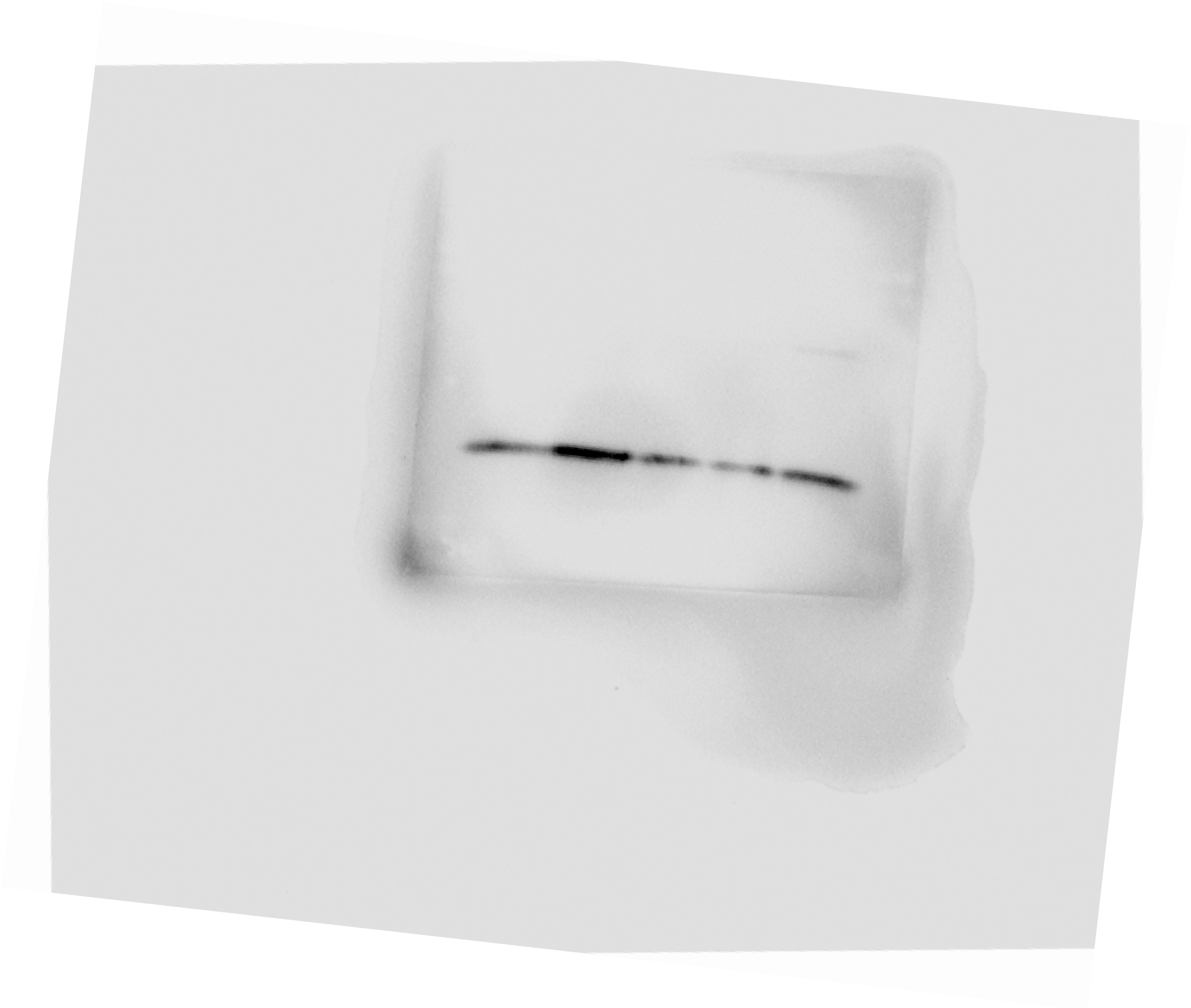

Figure 6F U87 SLC40A1

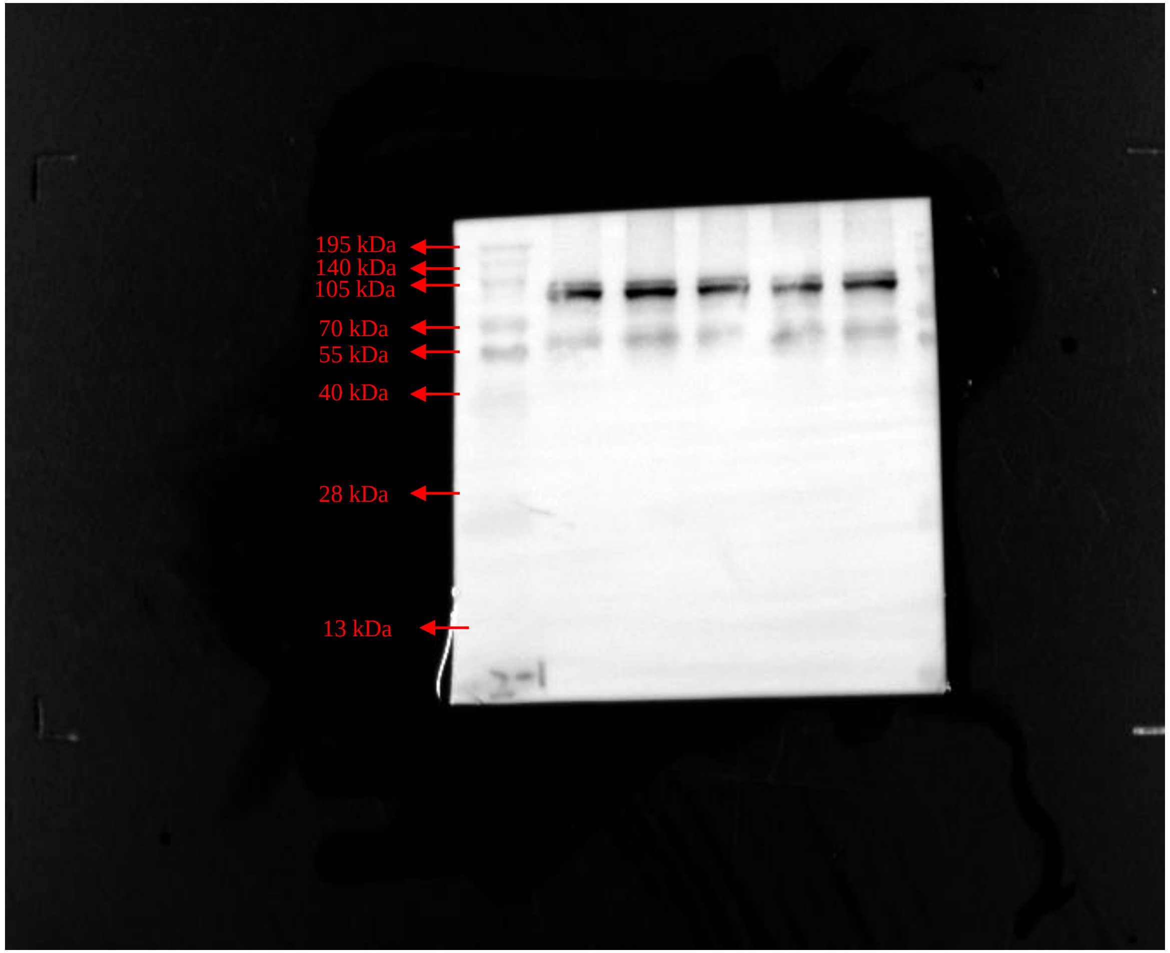

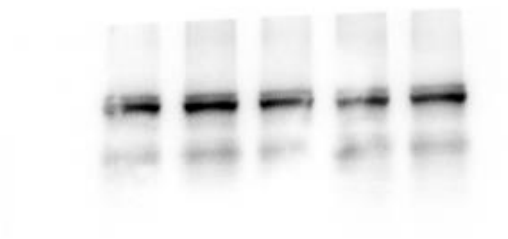

Figure 6F U87 GAPDH

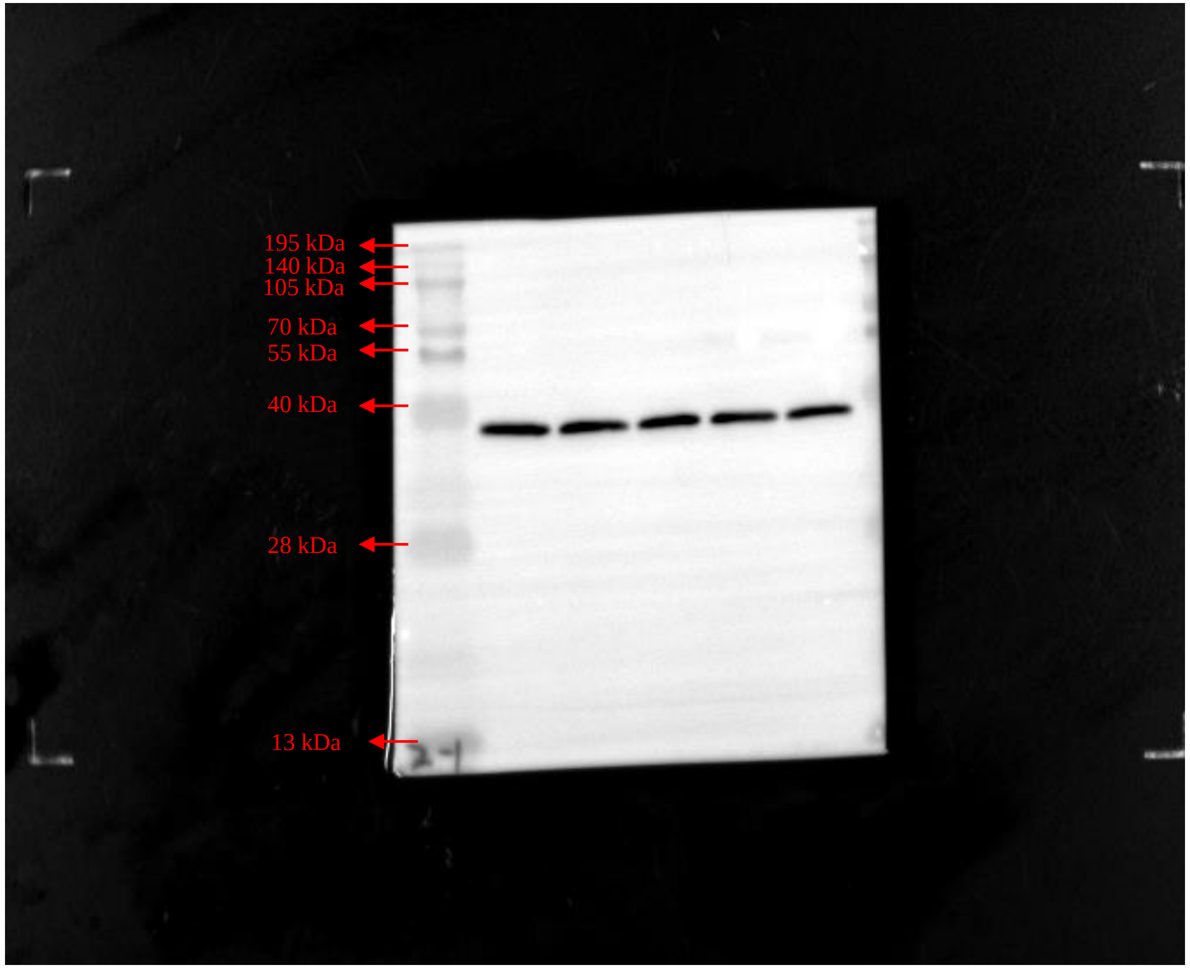

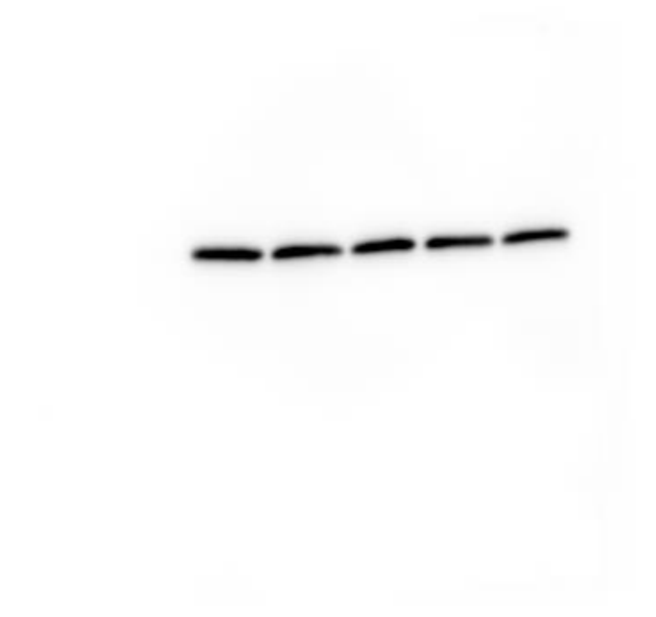

Figure 6F LN229 FOXP3

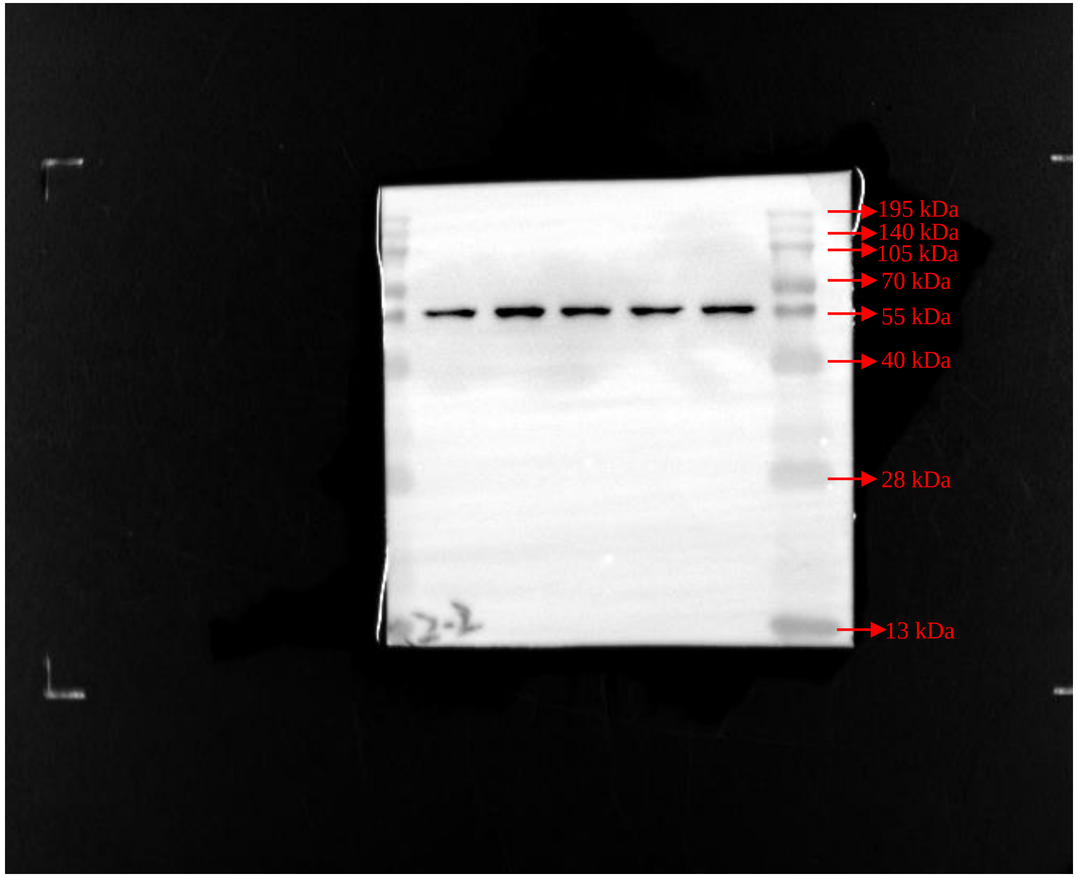

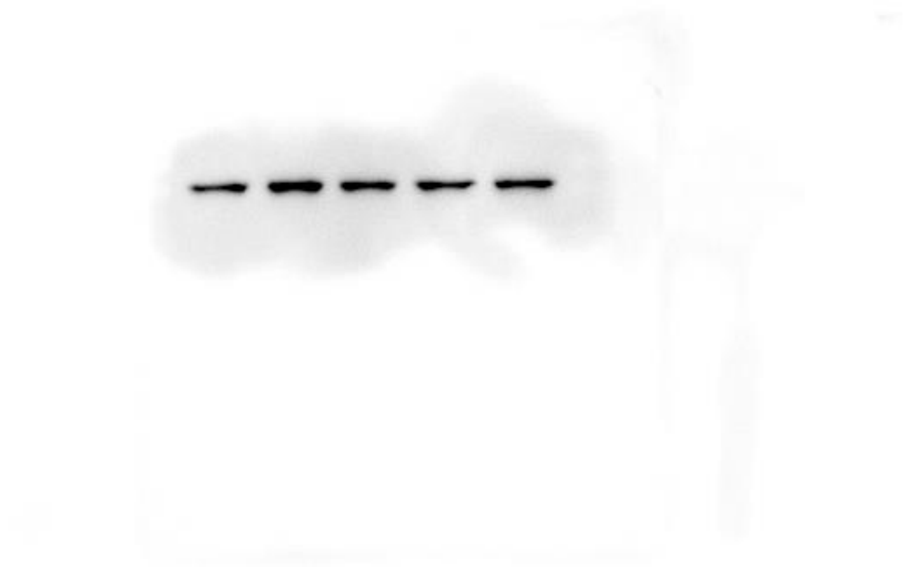

Figure 6F LN229 FTH1

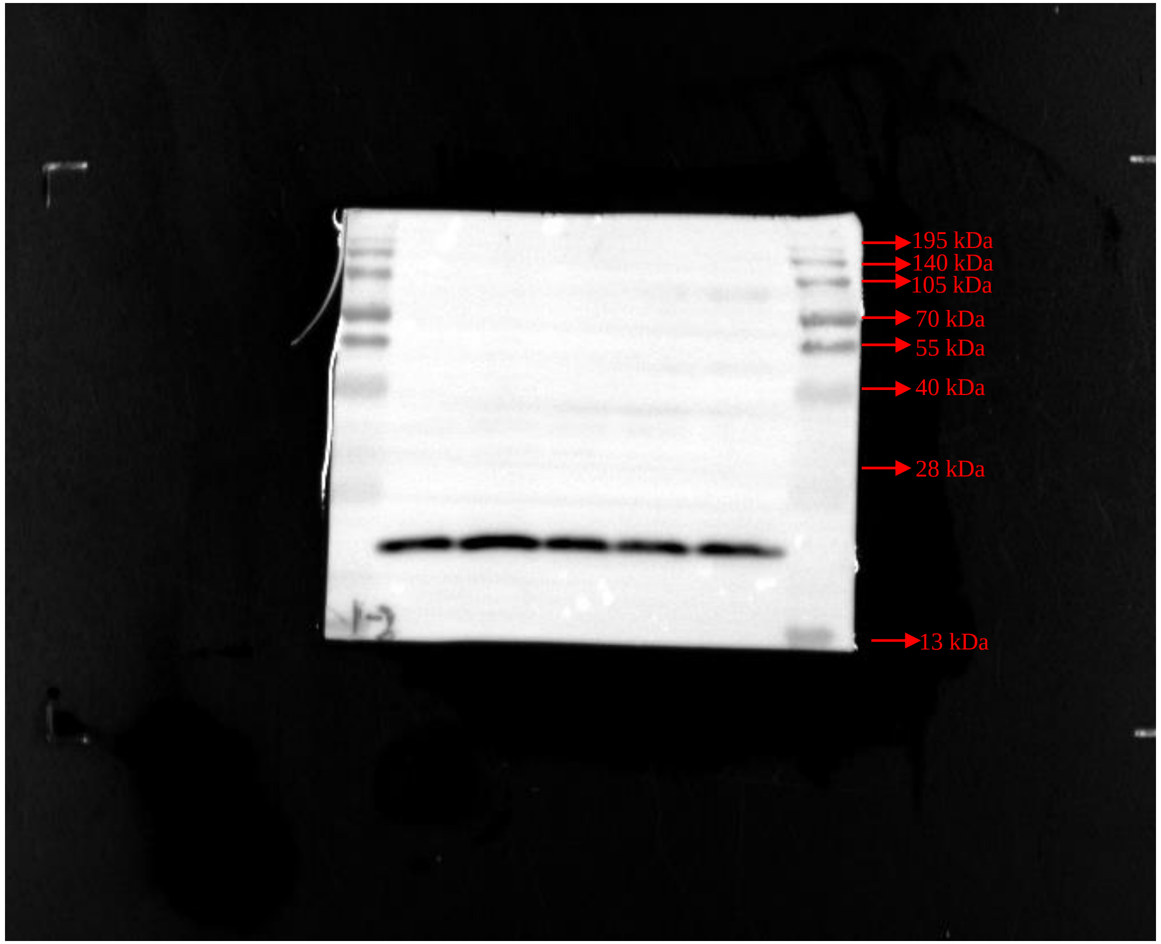

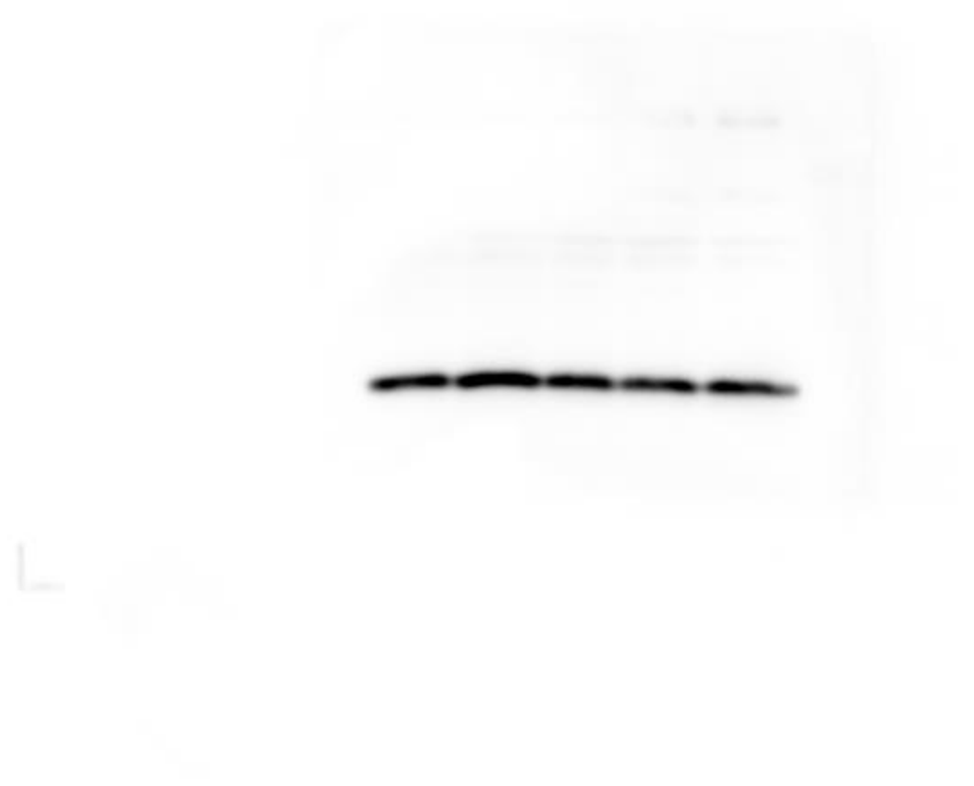

Figure 6F LN229 GPX4

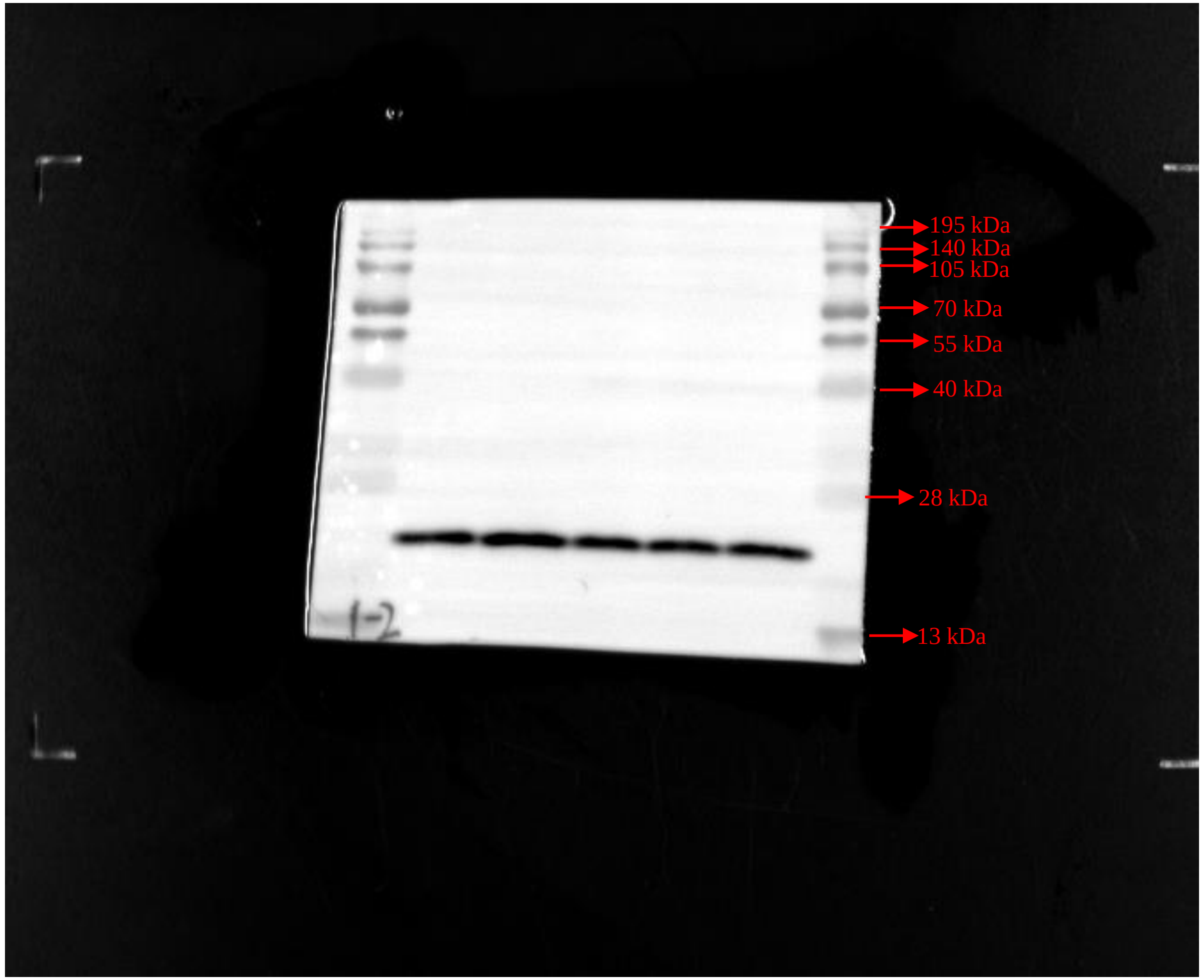

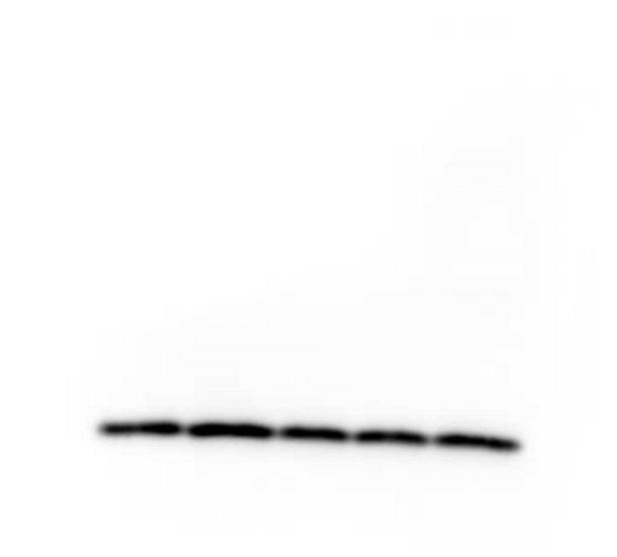

Figure 6F LN229 SLC7A11

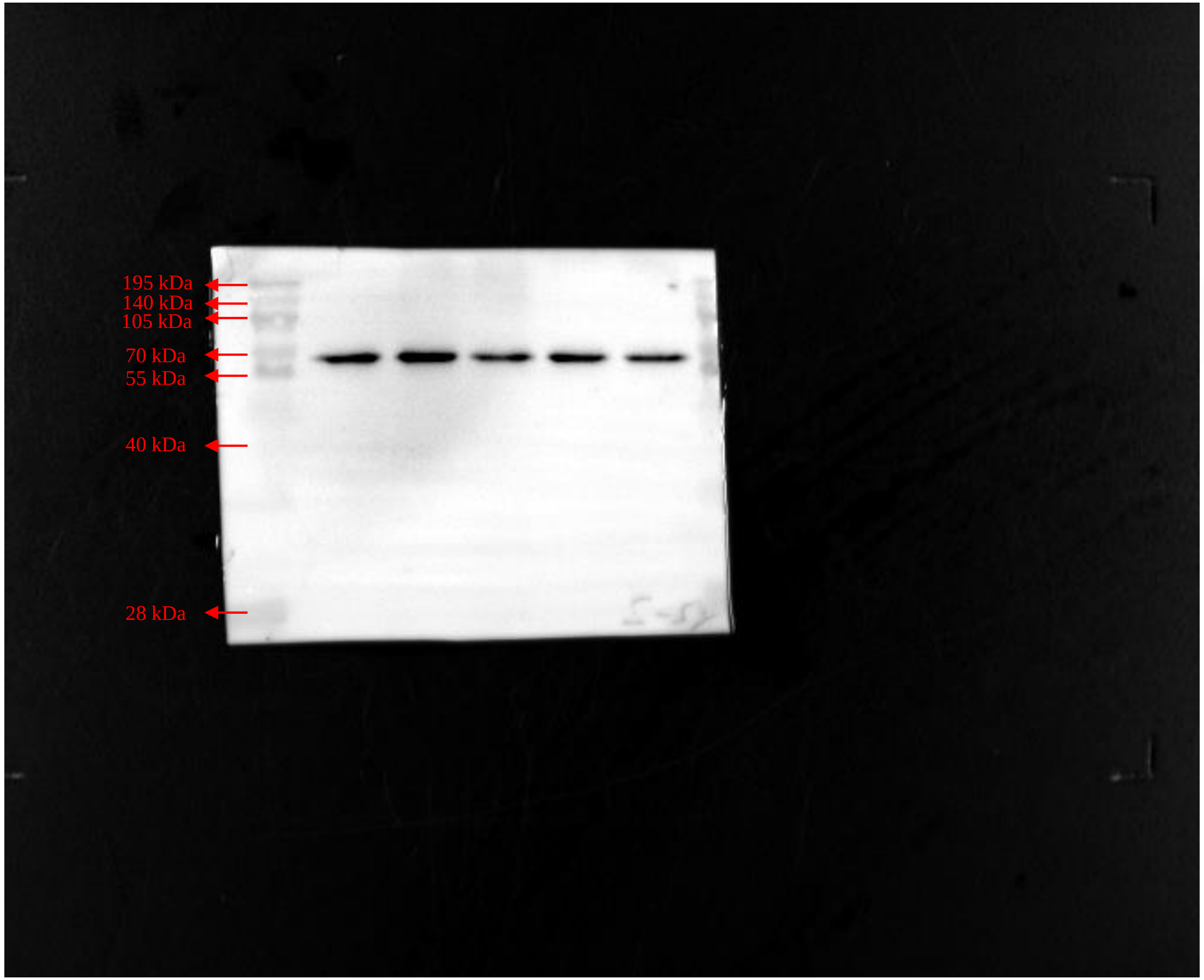

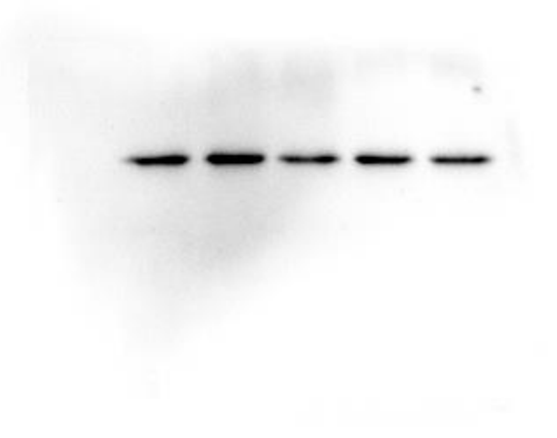

Figure 6F LN229 SLC40A1

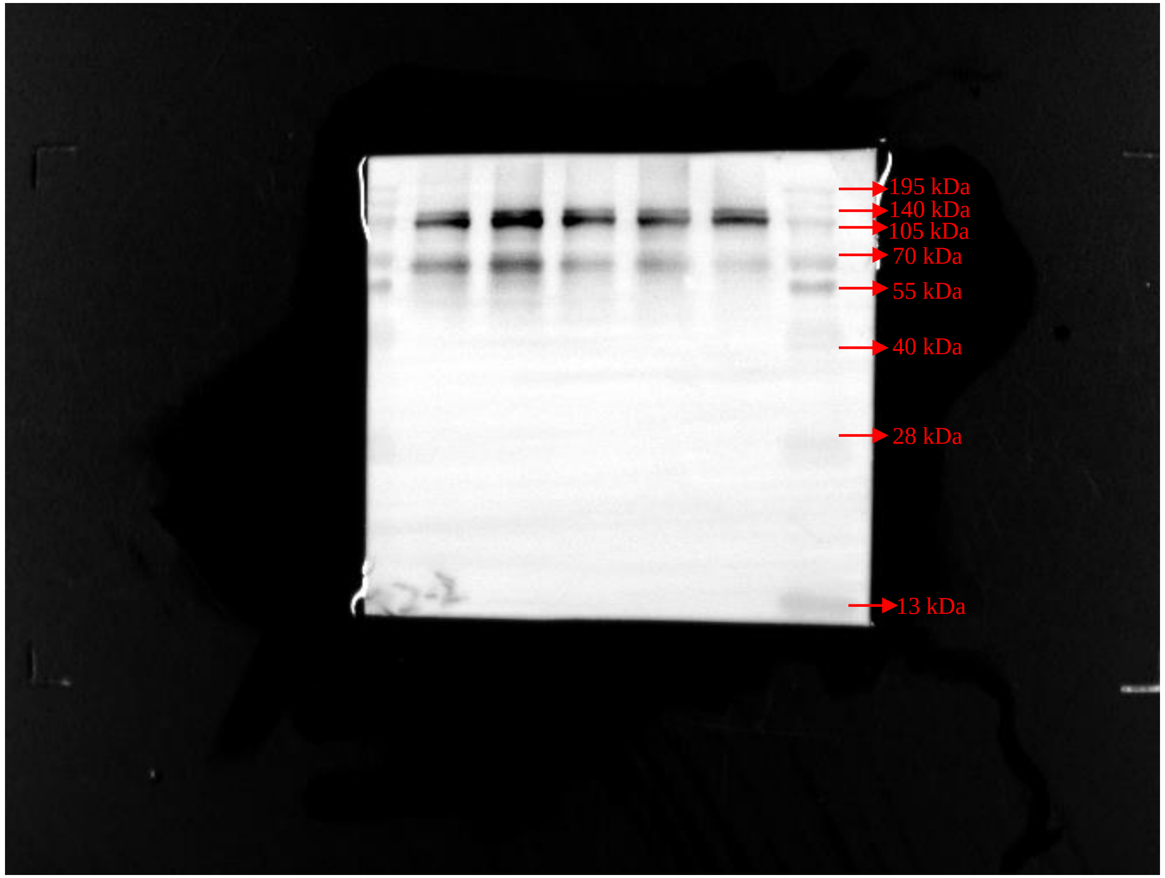

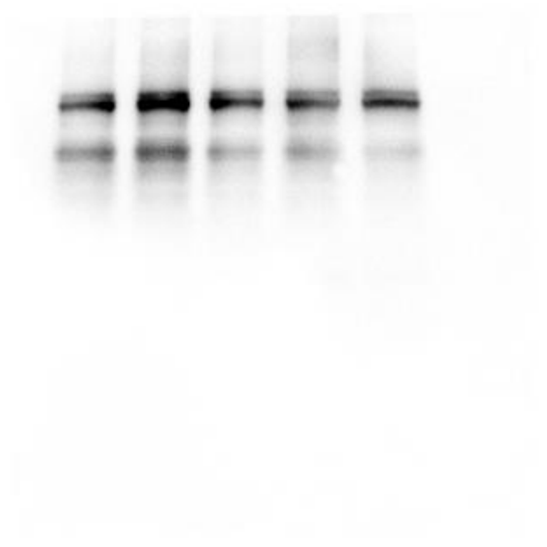

Figure 6F LN229 GAPDH

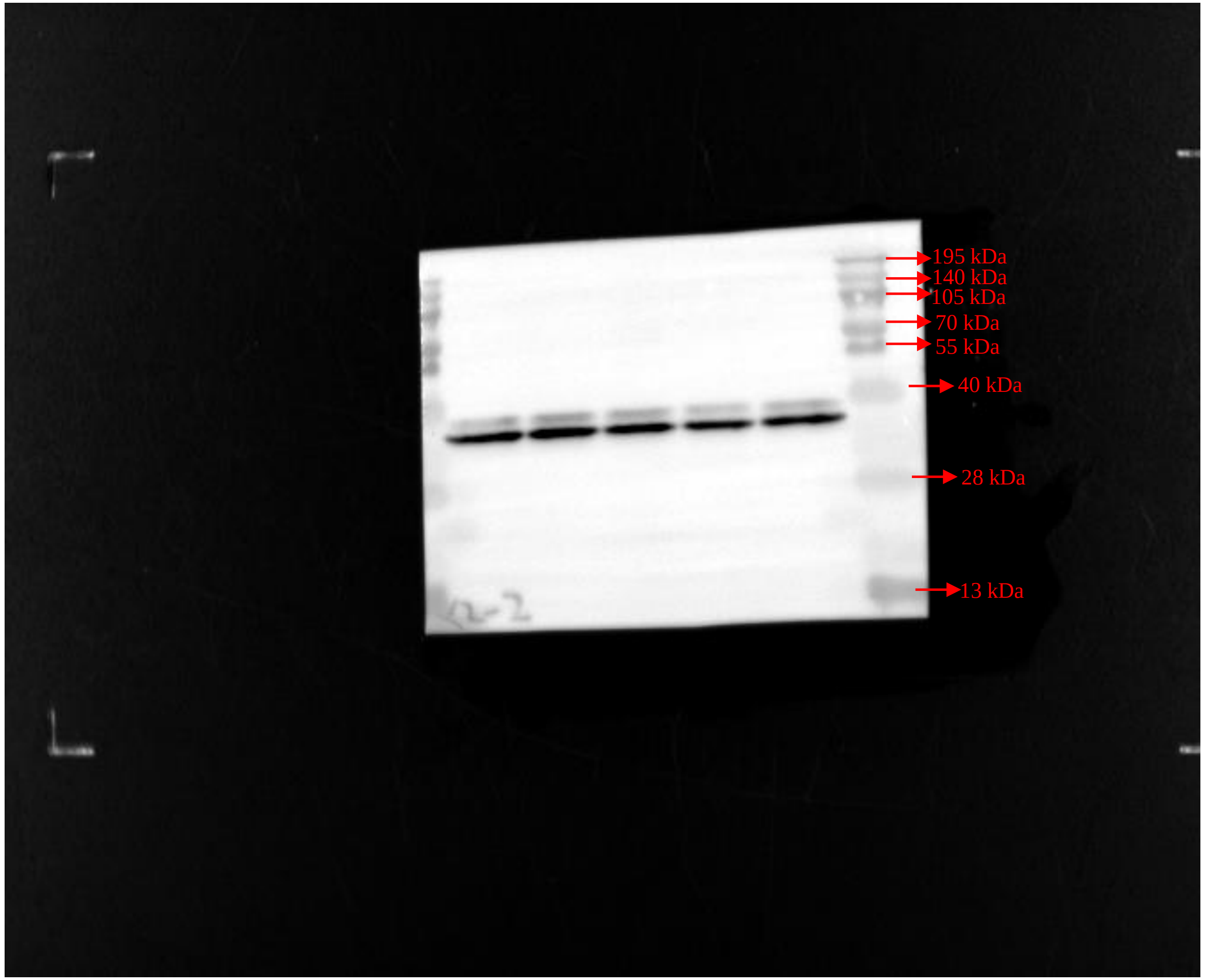

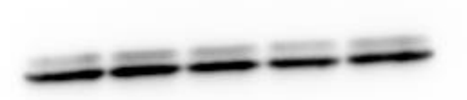

Supplement: Supplementary file 2 — Full and uncropped western blots [file 41419_2024_6619_MOESM2_ESM.pdf]
