# Supplementary figures and images for: FOXP3 promote the progression of glioblastoma via inhibiting ferroptosis mediated by linc00857/miR-1290/GPX4 axis
Source: Cell Death Dis. 2024 Apr 1;15(4):239. doi: 10.1038/s41419-024-06619-4 (PMC10984987; doi:10.1038/s41419-024-06619-4)

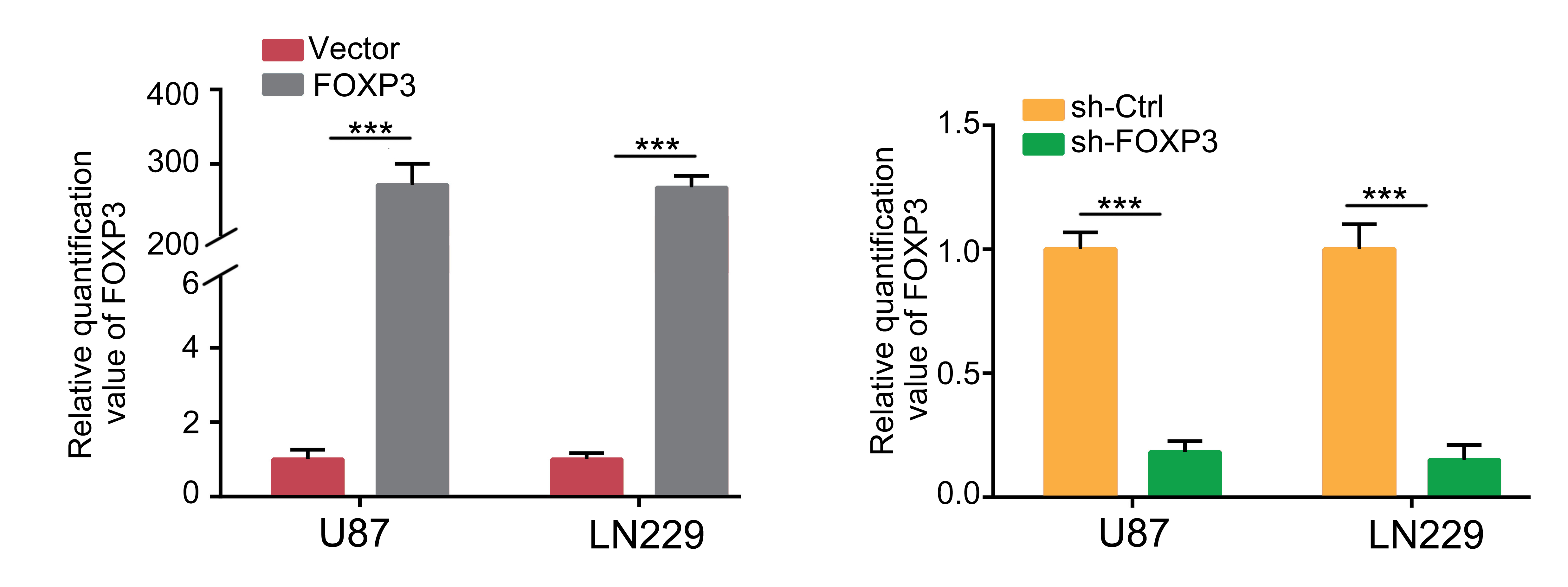

Supplement: Supplementary file 5 — Figure S3 [file 41419_2024_6619_MOESM5_ESM.tif]

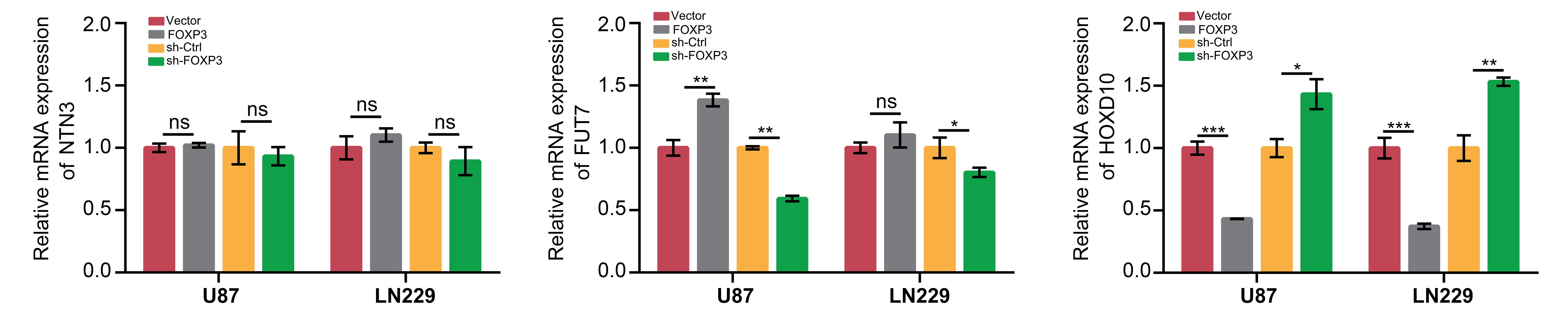

Supplement: Supplementary file 6 — Figure S4 [file 41419_2024_6619_MOESM6_ESM.tif]
